# Catalyst‐Free Deaminative Functionalizations of Primary Amines by Photoinduced Single‐Electron Transfer

**DOI:** 10.1002/anie.201814452

**Published:** 2019-03-14

**Authors:** Jingjing Wu, Phillip S. Grant, Xiabing Li, Adam Noble, Varinder K. Aggarwal

**Affiliations:** ^1^ School of Chemistry University of Bristol Cantock's Close Bristol BS8 1TS UK

**Keywords:** deamination, electron donor–acceptor complexes, Giese reactions, photochemistry, radical reactions

## Abstract

The use of pyridinium‐activated primary amines as photoactive functional groups for deaminative generation of alkyl radicals under catalyst‐free conditions is described. By taking advantage of the visible light absorptivity of electron donor–acceptor complexes between Katritzky pyridinium salts and either Hantzsch ester or Et_3_N, photoinduced single‐electron transfer could be initiated in the absence of a photocatalyst. This general reactivity platform has been applied to deaminative alkylation (Giese), allylation, vinylation, alkynylation, thioetherification, and hydrodeamination reactions. The mild conditions are amenable to a diverse range of primary and secondary alkyl pyridiniums and demonstrate broad functional group tolerance.

Visible light photochemistry in organic synthesis has witnessed a surge in research activity over the last decade.^1^ This is largely due to a growing appreciation of the synthetic utility of photoredox catalysts, which, upon photoexcitation, function as single‐electron or energy transfer catalysts to provide access to free‐radical intermediates.^2^ An alternative strategy, that circumvents the need for catalysis, is direct photoexcitation of a substrate, which has classically been performed using UV light.^3^ However, recent developments have taken advantage of the visible light absorptivity of specific functional groups that act as photoactive handles to enable photoinduced electron transfer (PET).^4^ Although direct photoexcitation is possible with a number of different functional groups,^5^ such reactions more commonly take advantage of electron donor–acceptor (EDA) complexes, whose absorption spectra display a bathochromic shift relative to their constituent parts, thus enabling photoexcitation with visible light.^6^

These strategies have enabled the development of a broad range of radical transformations that proceed through visible light‐mediated PET under catalyst‐free conditions. However, such reactions are typically limited to the generation of perfluoroalkyl or stabilized alkyl radicals.^5^, ^7^, ^8^ Access to non‐stabilized alkyl radicals under such conditions is considerably more challenging,^9^, ^10^ with only a single report by Melchiorre and co‐workers that generates secondary alkyl radicals by direct photoexcitation of 4‐alkyl‐1,4‐dihydropyridine derivatives.^11^ We sought an alternative functional group that could act as a versatile photoactive handle for catalyst‐free generation of non‐stabilized carbon‐centered radicals. One possibility was Katritzky *N*‐alkylpyridinium salts **1**, which are easily prepared from primary amines **2** by reaction with 2,4,6‐triphenylpyrylium **3**, are air and moisture stable, and allow selective deaminative transformations of abundant amino groups (Scheme 1 A).^12^ While these redox active amines have recently been applied to a number of radical‐mediated transformations, they usually rely on catalysis to promote single‐electron transfer (SET)‐induced deamination.^13^, ^14^

**Scheme 1:**
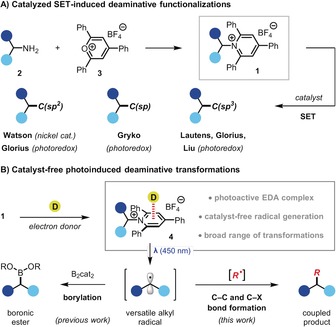
Radical‐mediated transformations of Katritzky pyridinium salts.

We recently reported a catalyst‐free deaminative borylation reaction that proceeds through EDA complex formation between **1** and bis(catecholato)diboron (B_2_cat_2_) (Scheme 1 B).^15^ Subsequent PET and fragmentation provided efficient access to non‐stabilized alkyl radicals that were intercepted by the diboron reagent. We reasoned that the 2,4,6‐triphenylpyridinium moiety in **1** could be complexed with other electron‐donors to generate EDA complex **4**,^16^ thus providing a photoactive handle capable of generating non‐stabilized alkyl radicals for application in a diverse range of C−C or C−X bond forming reactions (Scheme 1 B). Herein, we report that Katritzky pyridinium salts are versatile substrates for photoinduced deaminative functionalizations of primary amines under catalyst‐free conditions.

Our investigations began by studying the use of pyridiniums **1** in Giese reactions with electron‐deficient alkenes (Table 1). Such reactions are well‐developed using photocatalysis, but there are few reports of photoinduced reactions under catalyst‐free conditions.^17^ Given the overall transformation is reductive, a stoichiometric reductant was required. We selected Hantzsch ester (**5**) as this would act as a reductant but could also function as an electron‐donor to form the key EDA complex with **1**.10c,10d Gratifyingly, irradiation (*λ*_max_=450 nm) of a mixture of 4‐aminopiperidine‐derived pyridinium **1 a**, Hantzsch ester, and methyl acrylate in DMA yielded the desired Giese adduct **6** in 77 % yield (Table 1). Control experiments confirmed the necessity of light and **5** for successful reaction, and alternative reductants, such as Et_3_N, gave no desired product.^18^

**Table 1:** Giese reaction substrate scope.^[a]^

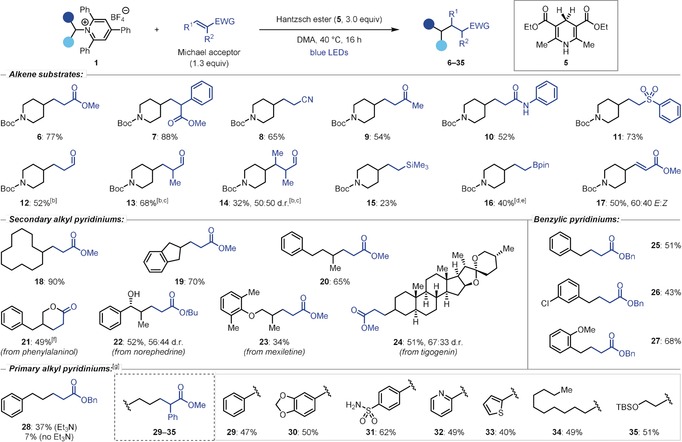

[a] General conditions: Pyridinium (0.2 mmol, 1.0 equiv), Michael acceptor (1.3 equiv) and **5** (3.0 equiv) in DMA (0.5 m) at 40 °C for 16 h. Yields are of isolated products after flash column chromatography. [b] Isolated as the alcohol after reduction with NaBH_4_. [c] Reaction performed at 60 °C for 40 h. [d] Isolated as the alcohol after oxidation with NaBO_3_. [e] Using 1.8 equiv of vinylboronic acid pinacol ester. [f] Lactonization was promoted by treatment with Amberlyst®. [g] Reactions performed at 60 °C in DMA (0.25 m) with the addition of Et_3_N (3.0 equiv). DMA=*N*,*N*‐dimethylacetamide. TBS=*tert*‐butyldimethylsilyl.

These optimized conditions were subsequently applied to a broad range of Michael acceptors (Table 1). Giese products from reactions with substituted acrylates (**7**), acrylonitrile (**8**), methyl vinyl ketone (**9**), *N*‐phenylacrylamide (**10**), and phenyl vinyl sulfone (**11**) were formed in good to excellent yields. Aldehydes were tolerated (**12**–**14**), although the substituted enals methacrolein (**13**) and tiglic aldehyde (**14**) required higher temperatures for successful reaction. Interestingly, vinyl silanes and boronic esters were also suitable substrates, providing products **15** and **16**, respectively, albeit in low yield. Finally, methyl propiolate underwent the Giese reaction to give alkene product **17** as a mixture of *E* and *Z* isomers.

With respect to the pyridinium salts, a variety of cyclic (**18** and **19**) and acyclic (**20**) secondary alkyl substrates reacted efficiently. The Giese product from a γ‐amino alcohol‐derived pyridinium could be cyclized by treatment with acid to generate lactone **21**. Alternatively, *t*‐butyl acrylate could be used in place of methyl acrylate to inhibit lactonization, allowing isolation of norephedrine‐derived alcohol **22**. Pharmaceutical and natural product derivatives were also readily accessed, as exemplified by the formation of product **23**, from the anti‐arrhythmic drug mexiletine, and **24**, from the steroid tigogenin.

While primary benzylic pyridiniums yielded products **25**–**27** in good yields, primary non‐benzylic substrates failed to undergo the deaminative Giese reaction. However, we found that adding Et_3_N to the reaction mixture and increasing the reaction temperature to 60 °C had a dramatic effect on the outcome of the reaction and enabled the isolation of adduct **28**, albeit in low yield. Switching from benzyl acrylate to the more activated alkene methyl 2‐phenylacrylate provided further improvements and enabled isolation of product **29** in 47 % yield. Despite the yield being moderate, this result is notable as it is a rare example of a photoinduced Giese reaction of a non‐stabilized primary alkyl radical under mild and catalyst‐free conditions. With these new conditions, a range of non‐benzylic primary alkyl pyridiniums reacted to give the Giese products (**29**–**35**) in moderate to good yields. Furthermore, the functional group tolerance of the methodology was highlighted by generating products bearing primary sulfonamide (**31**), pyridine (**32**), thiophene (**33**), and silyl ether (**35**) moieties.

To shed light on the mechanism of this catalyst‐free Giese reaction, we analyzed the reaction components by UV/Vis absorption spectroscopy. DMA solutions of secondary alkyl pyridinium **36** and Hantzsch ester (**5**) were both found to absorb in the visible region (>400 nm) (Figure 1 A). However, a mixture of **36** and **5** displayed a significant red‐shift in absorbance, confirming formation of the postulated EDA complex. A similar shift was observed with a mixture of primary alkyl pyridinium **37** and **5** (Figure 1 B). Interestingly, a mixture of **37**, **5**, and Et_3_N showed a further bathochromic shift, suggesting the formation of a ternary EDA complex, which could contribute to the enhanced reactivity observed with primary alkyl pyridiniums upon addition of Et_3_N. The formation of alkyl radical intermediates was confirmed by a radical clock experiment with cyclopropylmethyl pyridinium **38**, during which ring‐opening occurred to give alkene **39** as the only observable product (Figure 1 C).

**Figure 1:**
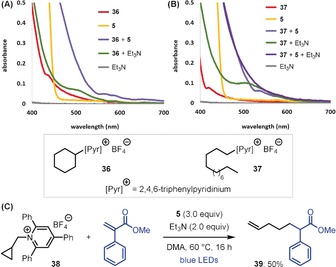
Mechanistic studies. A) Spectrophotometry of pyridinium **36**. B) Spectrophotometry of pyridinium **37**. C) Radical clock experiment.

These results suggest a mechanism comprised of initial formation of an EDA complex **40** between the electron‐deficient pyridinium **1** and electron‐rich Hantzsch ester (**5**) (Figure 2). Subsequent PET leads to dihydropyridine radical cation **41** and radical **42**, which fragments to triphenylpyridine **43** and alkyl radical **44**. Addition of **44** to methyl acrylate generates radical **45**, which undergoes hydrogen atom transfer (HAT) with dihydropyridine radical cation **41** (BDFE=31 kcal mol^−1^)^19^ or **5** (BDFE=69 kcal mol^−1^)^19^ to form Giese product **46** (BDFE≈96 kcal mol^−1^)^20^ and pyridinium **47** or dihydropyridine radical **48**, respectively.^21^

**Figure 2:**
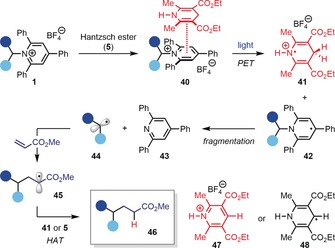
Proposed mechanism.

Encouraged by the results of the Giese reaction, we proceeded to investigate other catalyst‐free transformations. Pleasingly, with only slight modification to the reaction conditions,^18^ allylation reactions with allyl sulfone **49** were also found to be efficient (Table 2).14c A range of secondary alkyl pyridiniums underwent the catalyst‐free deaminative allylation to give products **50**–**57** in moderate to good yields. As with the Giese reaction, although primary benzylic pyridiniums yielded the allylation product **58** under these conditions, non‐benzylic primary alkyl pyridiniums **59**–**68** required the addition of Et_3_N for successful reaction. The allylation reaction was found to tolerate a diverse range of functional groups, including alcohols (**53**), nitriles (**60**), sulfonamides (**61**), unprotected indoles (**63**), olefins (**65**), and secondary carbamates (**67**), and was also applied to various pharmaceuticals (**57** and **66**) and natural product derivatives (**55**, **56**, **67**, and **68**). Furthermore, the use of other allyl sulfone reagents enabled the preparation of styrene derivative **69** and alkenylboronic ester **70**.

**Table 2:** Allylation reaction substrate scope.^[a]^

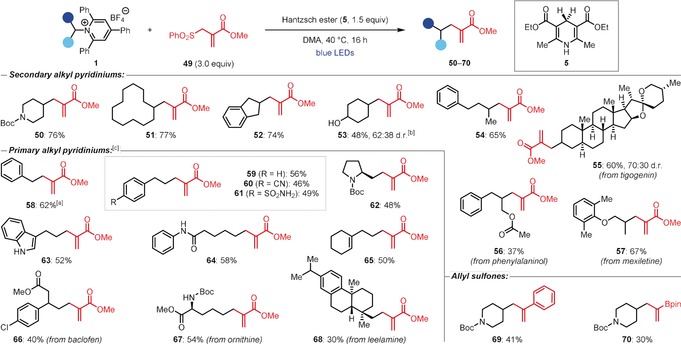

[a] General conditions: Pyridinium (1.0 equiv), allyl sulfone (3.0 equiv), and **5** (1.5 equiv) in DMA (0.4 m) at 40 °C for 16 h. Yields are of isolated products after flash column chromatography. [b] Isolated after acetyl protection of the alcohol. [c] Reactions performed at 60 °C with **5** (2.5 equiv) and Et_3_N (3.0 equiv).

During our UV/Vis absorbance studies of pyridinium **36** we found that it also forms an EDA complex with Et_3_N (Figure 1 A). Thus, we were curious as to whether these photoinduced reactions could be performed with Et_3_N in place of Hantzsch ester. While the Giese reaction proceeded with low yield, the allylation reaction proceeded smoothly to generate **50** in 71 % when using 6.0 equiv of Et_3_N in place of Hantzsch ester (Scheme 2).^18^ An identical result was also obtained when Et_3_N was replaced by *i*Pr_2_NEt. This result is intriguing given that these conditions are very similar to the photoredox‐catalyzed conditions recently reported by Liu and co‐workers, which differ only by the use of an iridium photocatalyst.14c We also investigated other addition–elimination reactions with unsaturated sulfone reagents and found that alkynylation and vinylation reactions also proceeded under our catalyst‐free conditions, generating alkyne **71** and alkene **72** in good yields. Again, these conditions are similar to previously reported photoredox‐catalyzed protocols by Gryko and co‐workers but proceed efficiently in the absence of a photocatalyst.14b Finally, we found that by replacing the unsaturated sulfones with other sulfur‐based reagents, under otherwise identical conditions, high yielding hydrodeamination and deaminative thioetherification reactions were also possible, providing good yields of *N*‐Boc‐piperidine **73** and thioether **74**, respectively.

**Scheme 2:**
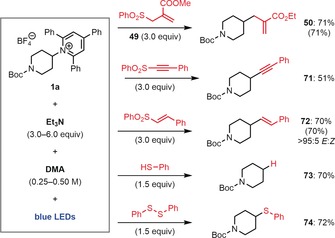
Deaminative transformations promoted by Et_3_N. Reactions performed at 60 °C. Yields in parentheses are for reactions performed using *i*Pr_2_NEt in place of Et_3_N.

In conclusion, we have described the development of a general catalyst‐free deaminative protocol for the generation of non‐stabilized alkyl radicals, proceeding through visible light photoexcitation of EDA complexes of *N*‐alkylpyridinium salts. The radicals were shown to undergo a range of transformations, including Giese, allylation, vinylation, alkynylation, HAT, and thioetherification reactions. The mild conditions, high functional group tolerance and ease of synthesis of the pyridinium substrates make this a useful catalyst‐free approach to alkyl radical formation.

## Conflict of interest

The authors declare no conflict of interest.

## Supporting information

As a service to our authors and readers, this journal provides supporting information supplied by the authors. Such materials are peer reviewed and may be re‐organized for online delivery, but are not copy‐edited or typeset. Technical support issues arising from supporting information (other than missing files) should be addressed to the authors.

Supplementary

## References

[1a] S. Protti , M. Fagnoni , Photochem. Photobiol. Sci. 2009, 8, 1499; 19862408 10.1039/b909128a

[1b] T. P. Yoon , M. A. Ischay , J. Du , Nat. Chem. 2010, 2, 527; 20571569 10.1038/nchem.687

[1c] L. Buzzetti , G. E. M. Crisenza , P. Melchiorre , Angew. Chem. Int. Ed. 2018, 10.1002/anie.201809984; 30339746

[2a] Visible Light Photocatalysis in Organic Chemistry (Eds.: C. R. J. Stephenson, T. P. Yoon, D. W. C. MacMillan), Wiley-VCH, Weinheim, 2018;

[2b] J. W. Tucker , C. R. J. Stephenson , J. Org. Chem. 2012, 77, 1617; 22283525 10.1021/jo202538x

[2c] M. H. Shaw , J. Twilton , D. W. C. MacMillan , J. Org. Chem. 2016, 81, 6898; 27477076 10.1021/acs.joc.6b01449 PMC4994065

[2d] K. L. Skubi , T. R. Blum , T. P. Yoon , Chem. Rev. 2016, 116, 10035; 27109441 10.1021/acs.chemrev.6b00018 PMC5083252

[2e] N. A. Romero , D. A. Nicewicz , Chem. Rev. 2016, 116, 10075. 27285582 10.1021/acs.chemrev.6b00057

[3a] N. Hoffmann , Chem. Rev. 2008, 108, 1052; 18302419 10.1021/cr0680336

[3b] T. Bach , J. P. Hehn , Angew. Chem. Int. Ed. 2011, 50, 1000; 10.1002/anie.201002845 21246702

[4] M. Silvi , P. Melchiorre , Nature 2018, 554, 41. 29388950 10.1038/nature25175

[5] For selected examples, see:

[5a] G. Cecere , C. M. König , J. L. Alleva , D. W. C. MacMillan , J. Am. Chem. Soc. 2013, 135, 11521; 23869694 10.1021/ja406181e PMC3786402

[5b] M. Silvi , E. Arceo , I. D. Jurberg , C. Cassani , P. Melchiorre , J. Am. Chem. Soc. 2015, 137, 6120; 25748069 10.1021/jacs.5b01662

[5c] M. Silvi , C. Sandford , V. K. Aggarwal , J. Am. Chem. Soc. 2017, 139, 5736; 28402109 10.1021/jacs.7b02569

[5d] A. Bahamonde , P. Melchiorre , J. Am. Chem. Soc. 2016, 138, 8019; 27267587 10.1021/jacs.6b04871 PMC4929524

[5e] G. Filippini , M. Silvi , P. Melchiorre , Angew. Chem. Int. Ed. 2017, 56, 4447; 10.1002/anie.201612045 PMC5396335 28323367

[5f] M. Silvi , C. Verrier , Y. P. Rey , L. Buzzetti , P. Melchiorre , Nat. Chem. 2017, 9, 868; 28837165 10.1038/nchem.2748

[5g] P. Bonilla , Y. P. Rey , C. M. Holden , P. Melchiorre , Angew. Chem. Int. Ed. 2018, 57, 12819; 10.1002/anie.201808183 PMC6175195 30098097

[5h] G. Goti , B. Bieszczad , A. Vega-Peñaloza , P. Melchiorre , Angew. Chem. Int. Ed. 2019, 58, 1213; 10.1002/anie.201810798 PMC6468318 30419156

[6a] S. V. Rosokha , J. K. Kochi , Acc. Chem. Res. 2008, 41, 641; 18380446 10.1021/ar700256a

[6b] C. G. S. Lima , T. de M. Lima , M. Duarte , I. D. Jurberg , M. W. Paixão , ACS Catal. 2016, 6, 1389;

[6c] A. Postigo , Eur. J. Org. Chem. 2018, 6391.

[7] For selected examples of perfluoroalkyl radical generation, see:

[7a] P. V. Pham , D. A. Nagib , D. W. C. MacMillan , Angew. Chem. Int. Ed. 2011, 50, 6119; 10.1002/anie.201101861 PMC3310164 21604347

[7b] M. Nappi , G. Bergonzini , P. Melchiorre , Angew. Chem. Int. Ed. 2014, 53, 4921; 10.1002/anie.201402008 24668827

[7c] Ł. Woźniak , J. J. Murphy , P. Melchiorre , J. Am. Chem. Soc. 2015, 137, 5678; 25901659 10.1021/jacs.5b03243 PMC4428001

[7d] J. W. Beatty , J. J. Douglas , R. Miller , R. C. McAtee , K. P. Cole , C. R. J. Stephenson , Chem 2016, 1, 456; 28462396 10.1016/j.chempr.2016.08.002 PMC5409161

[7e] M. L. Spell , K. Deveaux , C. G. Bresnahan , B. L. Bernard , W. Sheffield , R. Kumar , J. R. Ragains , Angew. Chem. Int. Ed. 2016, 55, 6515; 10.1002/anie.201601566 27086646

[7f] Y. Cheng , S. Yu , Org. Lett. 2016, 18, 2962; 27227431 10.1021/acs.orglett.6b01301

[7g] Y.-Y. Liu , X.-Y. Yu , J.-R. Chen , M.-M. Qiao , X. Qi , D.-Q. Shi , W.-J. Xiao , Angew. Chem. Int. Ed. 2017, 56, 9527; 10.1002/anie.201704690 28636809

[7h] H.-Y. Tu , S. Zhu , F.-L. Qing , L. Chu , Chem. Commun. 2018, 54, 12710; 10.1039/c8cc07344a 30345997

[7i] Q. Guo , M. Wang , H. Liu , R. Wang , Z. Xu , Angew. Chem. Int. Ed. 2018, 57, 4747; 10.1002/anie.201800767 29476596

[8] For stabilized alkyl radical generation, see:

[8a] E. Arceo , I. D. Jurberg , A. Álvarez-Fernández , P. Melchiorro , Nat. Chem. 2013, 5, 750; 23965676 10.1038/nchem.1727

[8b] E. Arceo , A. Bahamonde , G. Bergonzini , P. Melchiorro , Chem. Sci. 2014, 5, 2438;

[8c] S. R. Kandukuri , A. Bahamonde , I. Chatterjee , I. D. Jurberg , E. C. Escudero-Adán , P. Melchiorre , Angew. Chem. Int. Ed. 2015, 54, 1485; 10.1002/anie.201409529 25475488

[8d] Z.-Y. Cao , T. Ghosh , P. Melchiorre , Nat. Commun. 2018, 9, 3274; 30115906 10.1038/s41467-018-05375-2 PMC6095928

[8e] C.-W. Hsu , H. Sundén , Org. Lett. 2018, 20, 2051. 29561620 10.1021/acs.orglett.8b00597

[9] For aryl radical generation, see:

[9a] M. A. Fox , J. Younathan , G. E. Fryxell , J. Org. Chem. 1983, 48, 3109;

[9b] M. Tobisu , T. Furukawa , N. Chatani , Chem. Lett. 2013, 42, 1203;

[9c] L. Marzo , S. Wang , B. König , Org. Lett. 2017, 19, 5976; 29064719 10.1021/acs.orglett.7b03001

[9d] B. Liu , C.-H. Lim , G. M. Miyake , J. Am. Chem. Soc. 2017, 139, 13616. 28910097 10.1021/jacs.7b07390 PMC5920654

[10] For heteroatom-centered radical generation, see:

[10a] J. Davies , S. G. Booth , S. Essafi , R. A. W. Dryfe , D. Leonori , Angew. Chem. Int. Ed. 2015, 54, 14017; 10.1002/anie.201507641 PMC4648045 26412046

[10b] D. F. Reina , E. M. Dauncey , S. P. Morcillo , T. D. Svejstrup , M. V. Popescu , J. J. Douglas , N. S. Sheikh , D. Leonori , Eur. J. Org. Chem. 2016, 2108;

[10c] J. Zhang , Y. Li , R. Xu , Y. Chen , Angew. Chem. Int. Ed. 2017, 56, 12619; 10.1002/anie.201707171 28809077

[10d] Y. Li , J. Zhang , D. Li , Y. Chen , Org. Lett. 2018, 20, 3296. 29767989 10.1021/acs.orglett.8b01172

[11] L. Buzzetti , A. Prieto , S. R. Roy , P. Melchiorre , Angew. Chem. Int. Ed. 2017, 56, 15039; 10.1002/anie.201709571 PMC5698711 28984403

[12] J. B. Bapat , R. J. Blade , A. J. Boulton , J. Epsztajn , A. R. Katritzky , J. Lewis , P. Molina-Buendia , P.-L. Nie , C. A. Ramsden , Tetrahedron Lett. 1976, 17, 2691.

[13] For nickel-catalyzed reactions, see:

[13a] C. H. Basch , J. Liao , J. Xu , J. J. Piane , M. P. Watson , J. Am. Chem. Soc. 2017, 139, 5313; 28359153 10.1021/jacs.7b02389 PMC5480296

[13b] J. Liao , W. Guan , B. P. Boscoe , J. W. Tucker , J. W. Tomlin , M. R. Garnsey , M. P. Watson , Org. Lett. 2018, 20, 3030; 29745674 10.1021/acs.orglett.8b01062 PMC6005208

[13c] W. Guan , J. Liao , M. P. Watson , Synthesis 2018, 50, 3231. 30174353 10.1055/s-0037-1610084 PMC6112771

[14] For photoredox-catalyzed reactions, see:

[14a] F. J. R. Klauck , M. J. James , F. Glorius , Angew. Chem. Int. Ed. 2017, 56, 12336; 10.1002/anie.201706896 28762257

[14b] M. Ociepa , J. Turkowska , D. Gryko , ACS Catal. 2018, 8, 11362;

[14c] M.-M. Zhang , F. Liu , Org. Chem. Front. 2018, 5, 3443;

[14d] F. J. R. Klauck , H. Yoon , M. J. James , M. Lautens , F. Glorius , ACS Catal. 2019, 9, 236.

[15a] J. Wu , L. He , A. Noble , V. K. Aggarwal , J. Am. Chem. Soc. 2018, 140, 10700; 30091912 10.1021/jacs.8b07103

[15b] J. Hu , G. Wang , S. Li , Z. Shi , Angew. Chem. Int. Ed. 2018, 57, 15227; 10.1002/anie.201809608 30253022

[15c] F. Sandfort , F. Strieth-Kalthoff , F. J. R. Klauck , M. J. James , F. Glorius , Chem. Eur. J. 2018, 24, 17210; For a mechanistically related decarboxylative borylation, see:30290050 10.1002/chem.201804246

[15d] A. Fawcett , J. Pradeilles , Y. Wang , T. Mutsuga , E. L. Myers , V. K. Aggarwal , Science 2017, 357, 283. 28619717 10.1126/science.aan3679

[16] For EDA complexes of Katritzky pyridiniums, see:

[16a] A. R. Katritzky , G. Z. de Ville , R. C. Patel , Tetrahedron Lett. 1980, 21, 1723;

[16b] A. R. Katritzky , G. De Ville , R. C. Patel , Tetrahedron 1981, 37, 25.

[17a] K. Okada , K. Okamoto , N. Morita , K. Okubo , M. Oda , J. Am. Chem. Soc. 1991, 113, 9401;

[17b] G. L. Lackner , K. W. Quasdorf , G. Pratsch , L. E. Overman , J. Org. Chem. 2015, 80, 6012; 26030387 10.1021/acs.joc.5b00794 PMC4697963

[17c] G. Pratsch , G. L. Lackner , L. E. Overman , J. Org. Chem. 2015, 80, 6025. 26030520 10.1021/acs.joc.5b00795 PMC4699447

[18] See Supporting Information for details.

[19] X.-Q. Zhu , H.-R. Li , Q. Li , T. Ai , J.-Y. Lu , Y. Yang , J.-P. Cheng , Chem. Eur. J. 2003, 9, 871.12584702

[20] J. J. Brocks , H.-D. Beckhaus , A. L. J. Beckwith , C. Rüchardt , J. Org. Chem. 1998, 63, 1935.

[21] See Supporting Information for further mechanistic discussions and evidence for the formation of radicals **42** and **48**.

