# Supplementary material for: Catalyst‐Free Deaminative Functionalizations of Primary Amines by Photoinduced Single‐Electron Transfer
Source: Angew Chem Int Ed Engl. 2019 Mar 14;58(17):5697–701. doi: 10.1002/anie.201814452 (PMC6492299; doi:10.1002/anie.201814452)
Supplement: Supplementary file 1 — Supplementary [file ANIE-58-5697-s001.pdf]

Angewandte Chemie
Eine Zeitschrift der Gesellschaft Deutscher Chemiker

Supporting Information

Catalyst-Free Deaminative Functionalizations of Primary Amines by Photoinduced Single-Electron Transfer

*Jingjing Wu[+], Phillip S. Grant[+], Xiabing Li, Adam Noble, and Varinder K. Aggarwal**

anie_201814452_sm_miscellaneous_information.pdf

Table of Contents

1. General Information **2**

2. Optimization Studies **3**

2.1. Giese reaction of secondary alkyl pyridiniums 3

2.2. Giese reaction of primary alkyl pyridiniums 4

2.3. Allylation reaction of secondary alkyl pyridiniums 5

2.4. Allylation reaction of primary alkyl pyridiniums 5

3. General Procedures **6**

4. Reaction Setup **9**

5. Synthesis of Starting Materials **10**

5.1. Pyridinium salts 10

5.2. Radical acceptors 17

6. Product Characterization **20**

6.1. Giese reactions 20

6.2. Allylation reactions 34

6.3. By-products 44

7. Deaminative Reactions Promoted by Et_3N (or *i*Pr_2NEt) **45**

8. Mechanisitic Studies **48**

8.1. UV/Vis absorption spectroscopy 48

8.2. Radical clock experiment 50

8.3. Quantum yield 51

8.4. Side-product formation 52

8.5. Mechanism of Hantzsch ester-promoted reactions 54

8.6. Mechanism of Et_3N-promoted reactions 57

9. References **58**

10. NMR Spectra **60**

1. General Information

Water is de-ionised and brine refers to a saturated aqueous solution of NaCl. Anhydrous DMA was purchased from Sigma-Aldrich or Acros and degassed by three freeze-pump-thaw cycles before use. Absolute EtOH was purchased from Acros. All reagents were used as received unless otherwise stated.

Flash column chromatography was carried out using silica gel (Aldrich, silica gel 60, 40-63 μm). Analytical thin-layer chromatography (TLC) was performed using aluminium-backed silica plates (0.25 mm, Merck, silica gel 60 F254). Compounds were visualised under UV light and/or by staining with aqueous basic potassium permanganate or an ethanolic solution of phosphomolybdic acid (PMA).

^{1}H, ^{13}C and ^{19}F NMR spectra were acquired at various field strengths, as indicated, using Bruker 400 MHz, Varian VNMR 500 MHz, and Bruker Cryo 500 MHz spectrometers. All NMR spectra were recorder at 25 °C, unless otherwise stated. Chemical shifts (δ) are given in parts per million (ppm) and referenced to $CDCl_3$ (^{1}H: 7.26 ppm, ^{13}C: 77.16) or DMSO-d_6 (^{1}H: 2.50 ppm, ^{13}C: 39.52). Coupling constants (*J*) are given in Hertz (Hz) and refer to apparent multiplicities (s = singlet, br. s = broad singlet, d = doublet, t = triplet, q = quartet, quin = quintet, sex = sextet, h = heptet, m = multiplet, dd = doublet of doublets, etc.). The ^{1}H NMR spectra are reported as follows: chemical shift (multiplicity, coupling constants, number of protons).

Gas chromatography (GC) was performed on an Agilent Technologies 6890N Network GC System using an Agilent HP-5 column (15 m × 0.25 mm × 0.25 μm). High-resolution mass spectra (HRMS) were recorded on a Bruker micrOTOF instrument using electrospray ionisation (ESI). Low-resolution mass spectra (LRMS) were recorded on an Agilent 7820A GC-MS equipped with a HP-5MS UI column (30 m × 0.25 mm × 0.25 μm) using electron ionisation (EI). Infra-red (IR) spectra were recorded on a Perkin Elmer Spectrum One FT-IR spectrometer as a thin film. Selected absorption maxima (ν_{max}) are reported in wavenumbers (cm^{-1}). UV/Vis absorption spectra were recorded using a Agilent Technologies Cary 300 UV/Vis spectrophotometer, in quartz cuvettes with a path length of 10 mm. Optical rotations were recorded using Optical rotations ($[\alpha]_D^T$) were measured on a Bellingham and Stanley Ltd. ADP220 polarimeter and are quoted in (° mL)(g dm)$^{-1}$.

2. Optimization Studies

2.1. Giese reaction of secondary alkyl pyridiniums

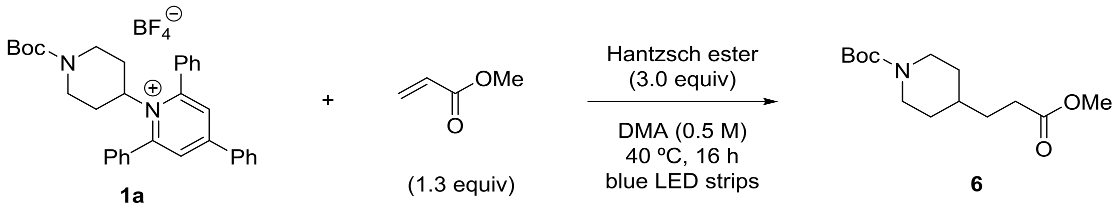

Entry	Deviation from above conditions	NMR yield of 6
**1**	none	(76% isolated yield)
**2**	dioxane as the solvent	<25%
**3**	MeCN as the solvent	<20%
**4**	DMF as the solvent	<30%
**5**	0.125 M concentration	69%
**6**	3.0 equiv of methyl acrylate	55%
**7**	no Hantzsch ester	0%
**8**	2 equiv of Hantzsch ester	44%
**9**	4.0 equiv of Hantzsch ester	76%
**10**	3.0 equiv of NEt_3 instead of Hantzsch ester	0%
**11**	no light*	0%
**12**	green LEDs	15%
**13**	open to air	<10%

Reactions were carried on a 0.1 mmol scale, yields were determined by ^{1}H NMR using 1,3,5-trimethoxybenzene as an internal standard. *The reaction was heated to 40 °C in the dark.

2.2. Giese reaction of primary alkyl pyridiniums

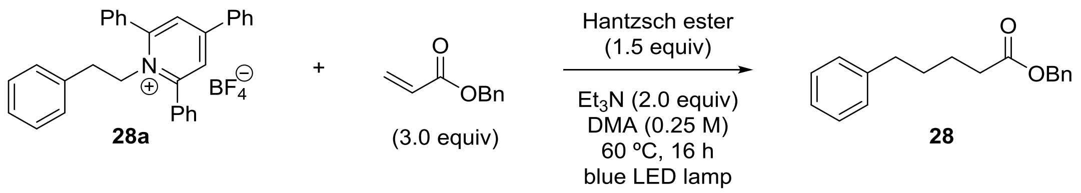

Entry	Deviation from above conditions	GC yield of 28
**1**	none	40%
**2**	no Et_3N, 2.0 equiv of benzyl acrylate	7%
**3**	1.3 equiv of benzyl acrylate	29%
**4**	2.0 equiv of benzyl acrylate	37%
**5**	0.5 M concentration	29%
**6**	0.50 M concentration with 2.5 equiv of Hantzsch ester	30%
**7**	0.10 M concentration	36%
**8**	0.10 M concentration with 2.5 equiv of Hantzsch ester	33%

Reactions were carried on a 0.1 mmol scale, yields were determined by GC using 1,3,5-trimethoxybenzene as an internal standard.

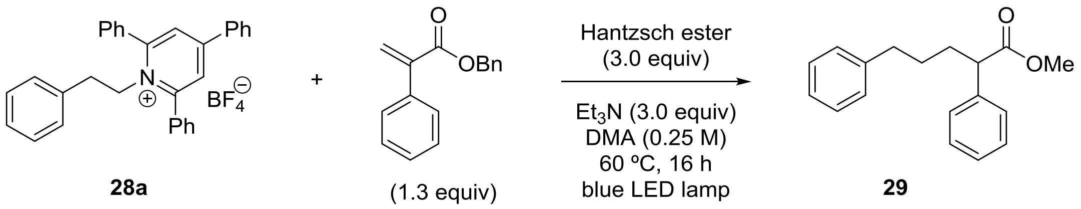

Entry	Deviation from above conditions	GC yield of 29
**1**	none	63%
**2**	2.0 equiv of Michael acceptor	62%
**3**	2.0 equiv of Michael acceptor, 2.0 equiv of Et_3N	60% (50%)
**4**	3.0 equiv of Michael acceptor	65%
**5**	2.0 equiv of Hantzsch ester	40%
**6**	DABCO (2 equiv) instead of Et_3N	54%
**7**	*i*Pr_2NEt (2 equiv) instead of Et_3N	63%

Reactions were carried on a 0.1 mmol scale, yields were determined by GC using 1,3,5-trimethoxybenzene as an internal standard. Isolated yields are in parenthesis.

2.3. Allylation reaction of secondary alkyl pyridiniums

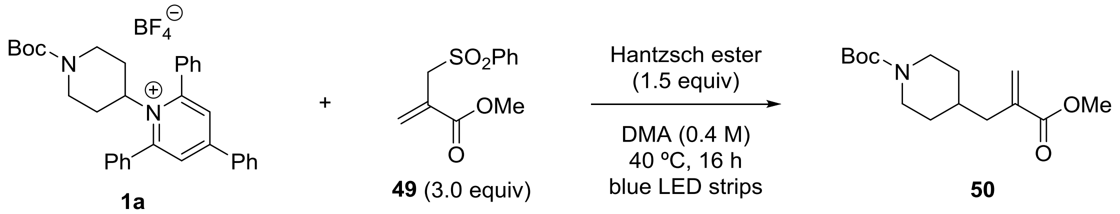

Entry	Deviation from above conditions	NMR yield of 53
**1**	none	93% (85%)
**2**	1.5 equiv of **49**	70%
**3**	2.0 equiv of **49**	78% (71%)
**4**	2.0 equiv of **49** and<br>Et_3N (3.0 equiv) instead of Hantzsch ester	40% (35%)
**5**	Et_3N (6.0 equiv) instead of Hantzsch ester	74% (71%)
**6**	no light*	0%

Reactions were carried on a 0.1 mmol scale, yields were determined by ^{1}H NMR using 1,3,5-trimethoxybenzene as an internal standard. Isolated yields are in parenthesis. *The reaction was heated to 40 °C in the dark.

2.4. Allylation reaction of primary alkyl pyridiniums

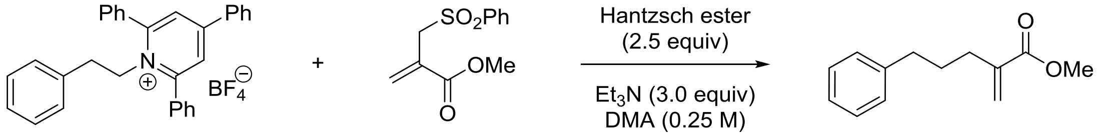

Entry	Deviation from above conditions	NMR yield of 62
**1**	none	56%
**2**	no Et_3N, 1.5 equiv of Hantzsch ester	0%
**3**	no Hantzsch ester, 6.0 equiv Et_3N	18%
**4**	1.5 equiv of Hantzsch ester	44%
**5**	3.0 equiv of Hantzsch ester	53%
**6**	quinuclidine (3 equiv) instead of Et_3N, 3.0 equiv of Hantzsch ester	36%
**7**	no light*	0%

Reactions were carried on a 0.1 mmol scale, yields were determined by ^{1}H NMR using 1,3,5-trimethoxybenzene as an internal standard. *The reaction was heated to 60 °C in the dark.

3. General Procedures

General Procedures 1A and 1B: Conversion of primary amines to pyridinium salts

Pyridinium salts were prepared by one of two procedures:

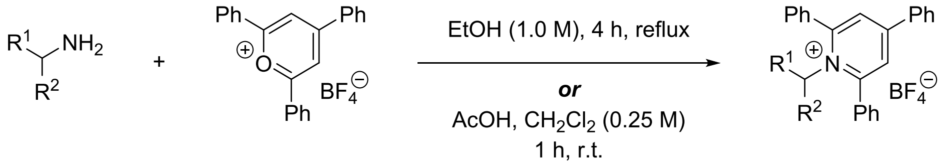

General procedure 1A (GP1A):

According to the procedure described by Watson et al.,[1] primary amine (1.2-1.5 equiv) was added to a suspension of 2,4,6-triphenylpyrylium tetrafluoroborate (1.0 equiv) and EtOH (1.0 M) in a long Schlenk tube. The mixture was stirred and heated at reflux in an oil bath at 85 ºC to 90 ºC for 4 h. The mixture was then allowed to cool to rt. If product precipitation occurred, the solid was filtered, washed with EtOH (3 × 25 mL) then Et_2O (3 × 25 mL), and dried under high vacuum. If product precipitation did not occur, the solution was diluted with Et_2O (2–3 × volume of EtOH used) and vigorously stirred for 1 h to induce trituration. The resulting solid pyridinium salt was filtered and washed with Et_2O (3 × 25 mL). If the pyridinium salt failed to precipitate at this point, it was purified by flash column chromatography, eluting with acetone/CH_2Cl_2.

Where the corresponding amine hydrochloride salt was used, a modified procedure was followed: Et_3N (1.2-1.3 equiv) was added to a mixture of the corresponding alkyl ammonium hydrochloride salt (1.2-1.3 equiv) and 2,4,6-triphenylpyrylium tetrafluoroborate (1 equiv) in EtOH (1.0 M). The mixture was stirred and heated at reflux in an oil bath at 85 ºC to 90 ºC for 4 h. The mixture was then cooled to rt and concentrated *in vacuo*. The crude residue was then purified by flash column chromatography, eluting with acetone/CH_2Cl_2.

General procedure 1B (GP1B):

According to the procedure described by Gabrielson et al.,[2] 2,4,6-triphenylpyrylium tetrafluoroborate (1.0 equiv) was added portionwise over 10 min to a solution of primary amine (1.3 equiv) in CH_2Cl_2 (0.25 M) containing 2-3 drops of acetic acid. The mixture was then stirred for 1.5 h at rt before being concentrated *in vacuo* and purified by flash column chromatography, eluting with CH_2Cl_2/acetone.

General Procedure 2 (GP2): Deaminative Giese reactions of secondary pyridinium Salts

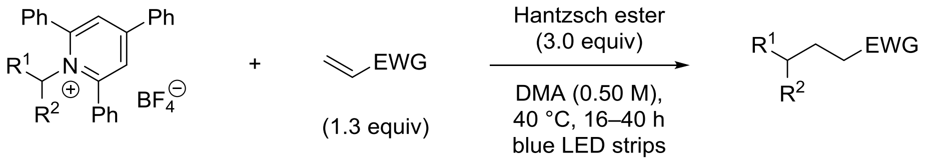

An oven-dried vial was charged with pyridinium salt (1.0 equiv) and Hantzsch ester (3.0 equiv), sealed, then evacuated and back-filled with N_2 three times. Degassed DMA (0.5 M) and the Michael acceptor (1.3 equiv), if liquid [*if the Michael acceptor was solid, it was added prior to sealing the vial*], were then added and the reaction mixture was stirred under blue LED irradiation for 16 h (unless otherwise stated). During the reaction, the reaction was covered with aluminium foil so that the heat generated by the LEDs resulted in a reaction temperature of 40–45 °C (see **Figure S1a** for experimental setup). The reaction mixture was diluted with EtOAc (25 mL), washed with H_2O (10 mL) and brine (10 mL), dried over $MgSO_4$, and concentrated *in vacuo*. The crude mixture was then purified by flash column chromatography or preparative thin layer chromatography.

General Procedure 3 (GP3): Deaminative Giese reactions of primary pyridinium salts

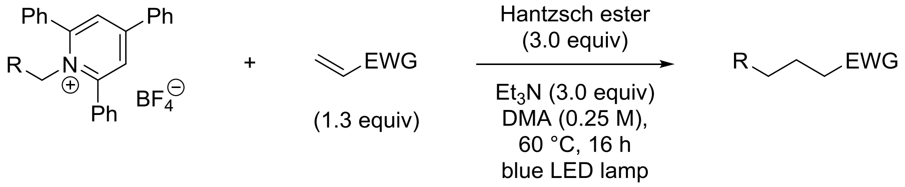

An oven-dried vial was charged with pyridinium salt (1.0 equiv), Hantzsch ester (3.0 equiv) and Michael acceptor (1.3 to 1.5 equiv), sealed, then evacuated and back-filled with N_2 three times. Degassed DMA (0.25 M) was then added, followed by freshly distilled Et_3N (3.0 equiv). The reaction mixture was stirred under blue LED irradiation for 16 h. During the reaction, the vial was placed touching the LEDs and the reaction was covered with aluminium foil so that the heat generated by the LEDs resulted in a reaction temperature of 60 °C (see **Figure S1b** for experimental setup). The reaction mixture was diluted with EtOAc (40 mL), washed with H_2O (10 mL) and brine (10 mL), dried over $MgSO_4$, and concentrated *in vacuo*. The crude mixture was then purified by flash column chromatography or preparative thin layer chromatography.

General Procedure 4 (GP4): Deaminative Allylations of secondary pyridinium salts

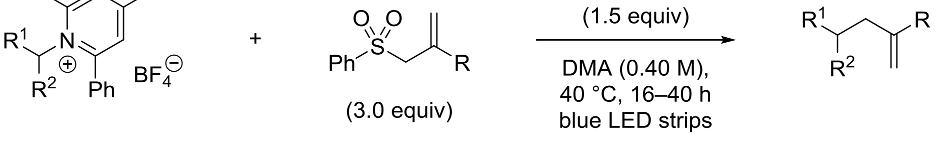

An oven-dried vial was charged with pyridinium salt (1.0 equiv), Hantzsch ester (1.5 equiv), and allyl sulfone (3.0 equiv), sealed, then evacuated and back-filled with N_2 three times. Degassed DMA (0.4 M) was then added, and reaction mixture was stirred under blue LED irradiation for 16 h (unless otherwise stated). During the reaction, the reaction was covered with aluminium foil so that the heat generated by the LEDs resulted in a reaction temperature of 40–45 °C (see **Figure S1a** for experimental setup). The reaction mixture was diluted with EtOAc (40 mL), washed with H_2O (10 mL) and brine (10 mL), dried

over $MgSO_4$, and concentrated *in vacuo*. The crude mixture was then purified by flash column chromatography or preparative thin layer chromatography.

General Procedure 5 (GP5): Deaminative Allylations of primary pyridinium salts

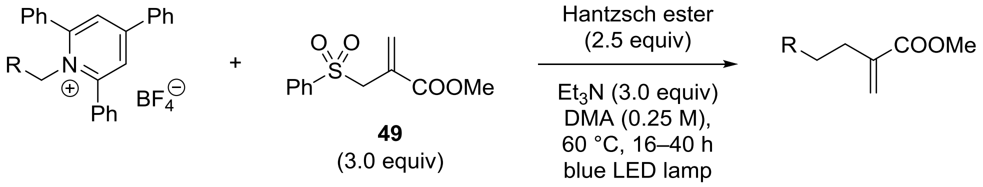

An oven-dried vial was charged with pyridinium salt (1.0 equiv), Hantzsch ester (2.5 equiv) and allyl sulfone (3.0 equiv), sealed, then evacuated and back-filled with N_2 three times. Degassed DMA (0.25 M) was then added, followed by freshly distilled Et_3N (3.0 equiv). The reaction mixture was stirred under blue LED irradiation for 16 h. During the reaction, the vial was placed touching the LEDs and the reaction was covered with aluminium foil so that the heat generated by the LEDs resulted in a reaction temperature of 60 °C (see **Figure S1b** for experimental setup). The reaction mixture was diluted with EtOAc (40 mL), washed with H_2O (10 mL) and brine (10 mL), dried over $MgSO_4$, and concentrated *in vacuo*. The crude mixture was then purified by flash column chromatography or preparative thin layer chromatography.

General Procedure 6 (GP6): Deaminative reactions promoted by Et_3N

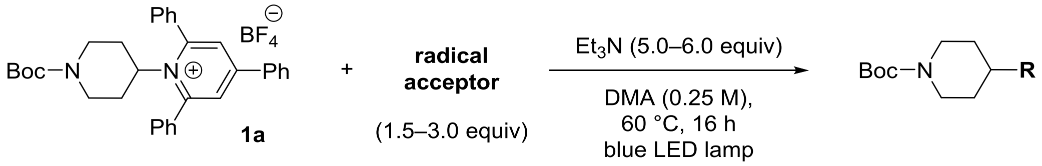

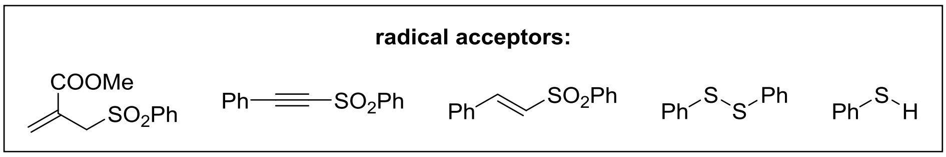

An oven-dried vial was charged with pyridinium salt (1.0 equiv), and radical acceptor (1.5–3.0 equiv), sealed, then evacuated and back-filled with N_2 three times. Degassed DMA (0.25 M) was then added as the solvent, followed by freshly distilled Et_3N (5.0–6.0 equiv). The reaction mixture was stirred under blue LED irradiation for 16 h (unless otherwise stated). During the reaction, the vial was placed touching the LEDs and the reaction was covered with aluminium foil so that the heat generated by the LEDs resulted in a reaction temperature of 60 °C (see **Figure S1b** for experimental setup). The reaction mixture was diluted with EtOAc (40 mL), washed with H_2O (10 mL) and brine (10 mL), dried over $MgSO_4$, and concentrated *in vacuo*. The crude mixture was then purified by flash column chromatography or preparative thin layer chromatography.

4. Reaction Setup

Blue LEDs, where specified, were either Fluxia blue SMD3528 LED strips, using approximately 5.0 m coiled around the inside of a 15 cm diameter crystallising dish (see Figure S1a); or a “100 W” blue/white LED fishing lamp (purchased from taobao.com, China), using only the blue LED setting (Figure S1b).

Optimum yields were observed when the reactions were heated by the heat generated by the LEDs. Therefore, the LEDs and reactions were wrapped in aluminium foil to maintain a high reaction temperature. For the blue LED strips (Figure S1a), this resulted in a reaction temperature of approximately 40 °C; for the LED lamp (Figure S1b), this resulted in a reaction temperature of approximately 60 °C

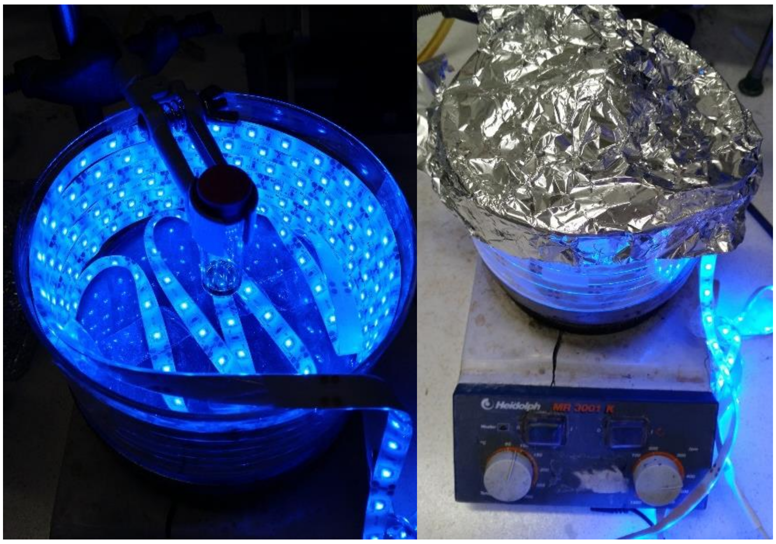

Figure S1a: Reaction setup for secondary/benzylic alkylpyridinium salts (GP2, GP4)

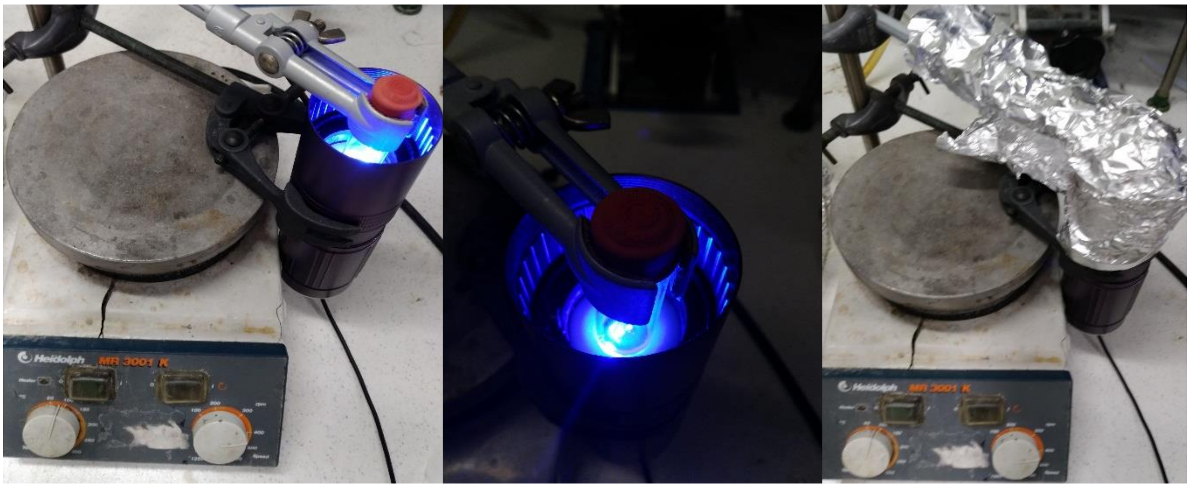

Figure S1b: Reaction setup for Primary alkylpyridinium salts (GP3, GP5 and GP6)

5. Synthesis of Starting Materials

5.1. Pyridinium salts

The pyridinium salts listed in **Table S1** were prepared according to our previous report.[1]

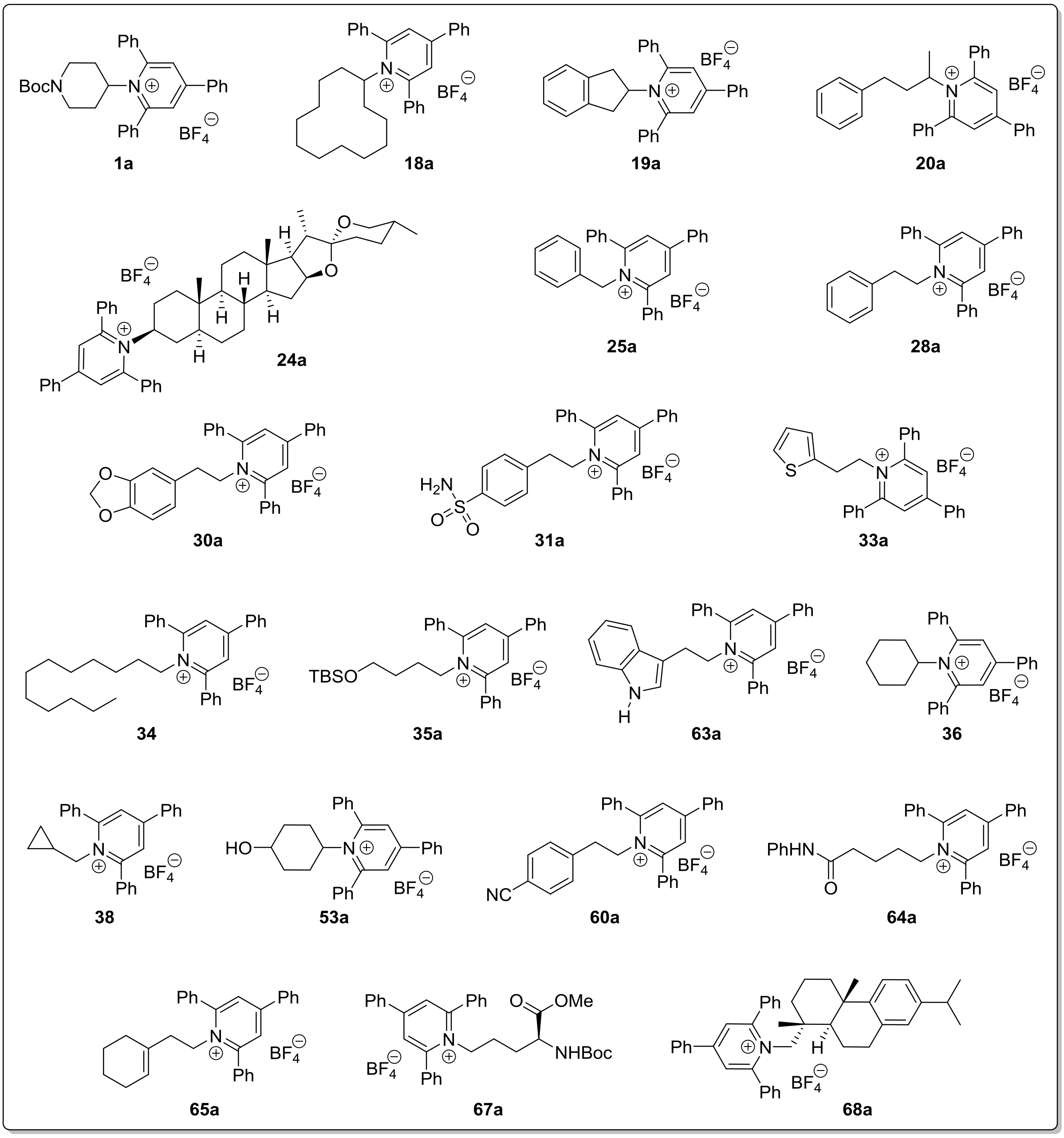

Table S1.

Synthesis of **21a**:

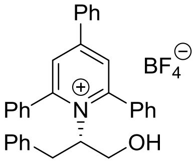

21a

Prepared following **GP1A** with 2,4,6-triphenylpyrylium tetrafluoroborate (4.00 g, 10.1 mmol), (*S*)-(–)-2-amino-3-phenyl-1-propanol (1.83 g, 12.1 mmol) and absolute EtOH (10 mL). Purification by flash column chromatography (5–60% acetone/CH_2Cl_2) gave the desired product **21a** (3.87 g, 78% yield) as a white amorphous solid.

^{1}H NMR (400 MHz, $CDCl_3$) δ 8.10 – 7.67 (m, 7H), 7.66 – 7.36 (m, 10H), 7.15 – 7.09 (m, 1H), 7.09 – 7.03 (m, 2H), 6.59 – 6.52 (m, 2H), 5.42 – 5.27 (m, 1H), 3.65 (m, 1H), 3.49 (m, 1H), 3.23 (dd, *J* = 14.1, 6.2 Hz, 1H), 2.47 (dd, *J* = 14.1, 6.2 Hz, 1H).

^{13}C NMR (100 MHz, $CDCl_3$) δ 155.1, 135.8, 133.7, 133.5, 132.3, 131.1, 130.2, 129.8, 129.1, 129.0, 128.6, 128.3, 127.5, 73.2, 62.4, 38.4 (*2 aromatic carbon signals are not observed due to signal broadening*).

^{19}F NMR (377 MHz, $CDCl_3$) δ –152.50 (minor, $^{10}BF_4$), –152.55 (major, $^{11}BF_4$).

HRMS (ESI$^+$): calculated for $C_{32}H_{28}NO$ [*M*–BF_4]$^+$ 442.2165, found 442.2157.

IR (ν_{max}/cm^{-1}, neat) 3523, 3062, 1618, 1599, 765, 702.

Synthesis of **22a**:

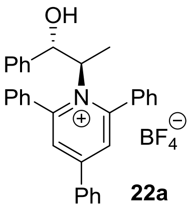

22a

Prepared following **GP1A** with 2,4,6-triphenylpyrylium tetrafluoroborate (1.40 g, 3.53 mmol), (1*S*,2*R*)-(+)-norephedrine (640 mg, 4.24 mmol) and absolute EtOH (3.5 mL). Purification by flash column chromatography (5–60% acetone/CH_2Cl_2) gave the desired product **22a** (1.02 g, 55% yield) as a yellow amorphous solid.

^{1}H NMR (400 MHz, $CDCl_3$) δ 7.65 – 7.36 (m, 17H), 7.16 – 6.99 (m, 3H), 6.60 (d, *J* = 7.0 Hz, 2H), 5.29 (p, *J* = 7.1 Hz, 1H), 4.94 (d, *J* = 5.6 Hz, 1H), 3.55 (s, 1H), 1.16 (d, *J* = 7.2 Hz, 3H).

^{13}C NMR (101 MHz, $CDCl_3$) δ 158.3, 155.1, 139.9, 134.4, 133.9, 132.1, 130.9, 130.6, 130.4, 129.6, 129.3, 129.0, 128.9, 128.7, 128.6, 128.5, 128.3, 128.1, 125.5, 74.2, 72.1, 17.3 (*2 aromatic carbon signals are not observed due to signal broadening*).

^{19}F NMR (377 MHz, $CDCl_3$) δ –152.58 (minor, $^{10}BF_4$), –152.63 (major, $^{11}BF_4$).

HRMS (ESI$^+$): calculated for $C_{32}H_{28}NO$ [*M*−BF_4]$^+$ 442.2165, found 442.2164.

IR (ν_{max}/cm^{-1}, neat) 3502 (br.), 3063, 1616, 1495, 1055, 891.

$[\alpha]_D^{25}$ = –76 (*c* 1.00, $CHCl_3$).

Synthesis of **23a**:

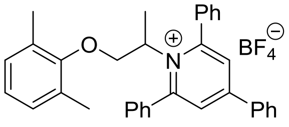

23a

Prepared following **GP1A** with 2,4,6-triphenylpyrylium tetrafluoroborate (1.19 g, 3.00 mmol), mexiletine hydrochloride (776 mg, 3.60 mmol), Et_3N (0.50 mL, 3.6 mmol) and absolute EtOH (3 mL). Purification by flash column chromatography (1–10% acetone/CH_2Cl_2) afforded the desired product **23a** (0.70 g, 42%) as a yellow amorphous solid.

^{1}H NMR (400 MHz, $CDCl_3$) δ 7.79 (br. s, 5H), 7.73 – 7.67 (m, 3H), 7.62 – 7.53 (m, 5H), 7.53 – 7.48 (m, 2H), 7.42 (d, J = 8.8 Hz, 2H), 6.96 – 6.82 (m, 3H), 5.45 (h, J = 7.1 Hz, 1H), 4.12 (dd, J = 9.8, 6.3 Hz, 1H), 3.51 (dd, J = 9.8, 7.2 Hz, 1H), 1.91 (s, 6H), 1.53 (d, J = 7.2 Hz, 3H).

^{13}C NMR (100 MHz, $CDCl_3$) δ 155.7, 154.4, 134.0, 133.7, 132.1, 131.1, 130.1, 129.7, 129.4, 129.3, 129.2, 129.1, 129.0, 129.0, 128.9, 128.7, 128.4, 124.5, 73.5, 65.3, 19.4, 16.3 (*1 aromatic carbon signal is not observed due to signal broadening*).

^{19}F NMR (377 MHz, $CDCl_3$) δ –152.79 (minor, $^{10}BF_4$), –152.84 (major, $^{11}BF_4$).

HRMS (ESI$^+$) calculated for $C_{34}H_{32}NO$ [M−BF_4]$^+$ 470.2478, found 470.2467.

IR (ν_{max}/cm^{-1}, neat) 3060, 1620, 1599, 1563, 763, 766.

Synthesis of **26a**:

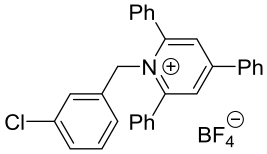

26a

Prepared following **GP1B** with 2,4,6-triphenylpyrylium tetrafluoroborate (400 mg, 1.01 mmol), 3-chlorobenzylamine (186 mg, 1.31 mmol), 2 drops of AcOH, and CH_2Cl_2 (2.2 mL). Purification by flash column chromatography (2–20% acetone/CH_2Cl_2) afforded the desired product **26a** (508 mg, 97% yield) as a white amorphous solid.

^{1}H NMR (400 MHz, $CDCl_3$) δ 7.91 (s, 2H), 7.80 – 7.75 (m, 2H), 7.67 – 7.61 (m, 4H), 7.59 – 7.42 (m, 9H), 7.12 (ddd, J = 8.1, 1.9, 1.2 Hz, 1H), 7.06 (t, J = 8.1 Hz, 1H), 6.46 – 6.40 (m, 1H), 6.33 (t, J = 1.9 Hz, 1H), 5.73 (s, 2H).

^{13}C NMR (101 MHz, CDCl3) δ 157.6, 156.7, 135.9, 134.7, 133.8, 132.7, 132.6, 131.2, 130.4, 129.9, 129.4, 129.2, 128.6, 128.3, 126.8, 126.7, 124.7, 57.7.

^{19}F NMR (377 MHz, $CDCl_3$) δ –152.76 (minor, $^{10}BF_4$), –152.82 (major, $^{11}BF_4$).

HRMS (ESI$^+$) calculated for $C_{33}H_{23}ClN$ [M−BF_4]$^+$ 432.1514, found 432.1530.

IR (ν_{max}/cm^{-1}, neat) 3062, 1620, 1594, 1495, 768, 702.

Synthesis of **27a**:

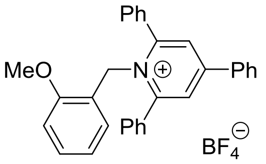

27a

Prepared following **GP1B** with 2,4,6-triphenylpyrylium tetrafluoroborate (400 mg, 1.01 mmol), 2-methoxybenzylamine (180 mg, 1.31 mmol), 2 drops of AcOH, and CH_2Cl_2 (2.2 mL). Purification by flash column chromatography (2–20% acetone/CH_2Cl_2) afforded the desired product **27a** (420 mg, 81% yield) as a white amorphous solid.

^{1}H NMR (400 MHz, $CDCl_3$) δ 7.82 (s, 2H), 7.75 (d, *J* = 8.3 Hz, 2H), 7.62 (d, *J* = 9.4 Hz, 4H), 7.55 – 7.39 (m, 9H), 7.08 (t, *J* = 8.7 Hz, 1H), 6.57 (t, *J* = 7.9 Hz, 2H), 6.23 (d, *J* = 7.4 Hz, 1H), 5.70 (s, 2H), 3.44 (s, 3H).

^{13}C NMR (101 MHz, $CDCl_3$) δ 158.1, 156.5, 155.9, 133.8, 132.3, 130.9, 129.9, 129.8, 129.2, 129.1, 128.8, 128.2, 126.1, 122.1, 120.6, 110.4, 77.4, 55.9, 55.2.

^{19}F NMR (377 MHz, $CDCl_3$) δ –152.93 (minor, $^{10}BF_4$), –152.99 (major, $^{11}BF_4$).

HRMS (ESI$^+$) calculated for $C_{33}H_{23}ClN$ [M−BF_4]$^+$ 428.2009, found 428.2004.

IR (ν_{max}/cm^{-1}, neat) 3063, 1619, 1494, 727, 699.

Synthesis of **32a**:

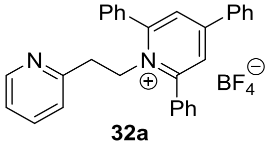

32a

Prepared following **GP1A** with triphenylpyrylium tetrafluoroborate (1.19 g, 3.00 mmol), 2-(2-pyridyl)-ethylamine (440 mg, 3.60 mmol) and absolute EtOH (3.0 mL). The resulting solid pyridinium salt was filtered, washed with Et_2O (3 mL) and dried under high vacuum to give the desired product **32a** (906 mg, 60% yield) as a white solid.

^{1}H NMR (400 MHz, $CDCl_3$) δ 8.19 (d, *J* = 4.7 Hz, 1H), 7.86 (s, 2H), 7.80 – 7.77 (m, 2H), 7.72 – 7.70 (m, 4H), 7.61 – 7.48 (m, 9H), 7.42 (td, *J* = 7.7, 1.6 Hz, 1H), 7.03 (dd, *J* = 7.3, 5.1 Hz, 1H), 6.63 (d, *J* = 7.8 Hz, 1H), 4.97 (t, *J* = 7.4 Hz, 2H), 2.89 (t, *J* = 7.4 Hz, 2H).

^{13}C NMR (125 MHz, $CDCl_3$) δ 157.2, 156.1, 156.0, 149.3, 136.8, 134.2, 133.0, 132.2, 131.2, 129.9, 129.5, 129.2, 128.3, 126.9, 123.4, 122.1, 54.3, 36.7.

^{19}F NMR (377 MHz, $CDCl_3$) δ –153.12 (minor, $^{10}BF_4$), –153.18 (dd, *J* = 2.0, 0.9 Hz, major, $^{11}BF_4$).

HRMS (ESI$^+$): calculated for $C_{30}H_{25}N_2$ [M−BF_4]$^+$ 413.2012, found 413.1998.

IR (ν_{max}/cm^{-1}, neat) 3072, 3050, 3007, 2925, 1622, 1093, 1050, 1037, 764, 700.

Synthesis of **56a**:

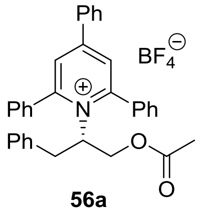

56a

To a solution of **21a** (200 mg, 0.400 mmol) and DMAP (2 mg, 0.008 mmol) in anhydrous CH_2Cl_2 (3 mL) was added Et_3N (0.1 mL, 0.8 mmol). The reaction mixture was then cooled to 0 °C. Acetyl chloride (0.04 mL, 0.6 mmol) was added dropwise to the mixture. The reaction was allowed to warm to rt and stirred overnight, before being quenched with H_2O and extracted with CH_2Cl_2 (3 × 20 mL). The combined organic layers were dried over Na_2SO_4 and concentration in vacuo. Purification by flash column chromatography (2–10% acetone/CH_2Cl_2) provided the desired product **56a** (173 mg, 80%) as a white solid.

^{1}H NMR (400 MHz, $CDCl_3$) δ 8.08 (br. s, 1H), 7.87 (br. s, 2H), 7.79 (d, *J* = 7.6 Hz, 2H), 7.76 – 7.35 (m, 12H), 7.16 – 7.07 (m, 3H), 6.62 (d, *J* = 7.3 Hz, 2H), 5.38 (tt, *J* = 9.8, 4.9 Hz, 1H), 4.18 (dd, *J* = 12.4, 9.6 Hz, 1H), 3.85 (dd, *J* = 12.4, 5.4 Hz, 1H), 3.69 (dd, *J* = 14.0, 4.4 Hz, 1H), 2.69 (dd, *J* = 14.0, 10.2 Hz, 1H), 1.95 (s, 3H).

^{13}C NMR (100 MHz, $CDCl_3$) δ 169.8, 156.2, 135.5, 133.9, 133.4, 132.4, 131.4, 129.8, 129.0, 128.7, 128.6, 127.7, 69.5, 63.9, 39.3, 20.6 (*4 aromatic carbon signals are not observed due to signal broadening*).

^{19}F NMR (377 MHz, $CDCl_3$) δ –152.76 (minor, $^{10}BF_4$), –152.82 (major, $^{11}BF_4$).

HRMS (ESI$^+$): calculated for $C_{34}H_{30}NO_2$ [*M*–BF_4]$^+$ 484.2271, found 484.2253.

IR (ν_{max}/cm^{-1}, neat) 3061,3029, 2961, 2967, 1746, 1619, 1598, 1228, 1055, 1064, 893, 703.

Synthesis of **62a**:

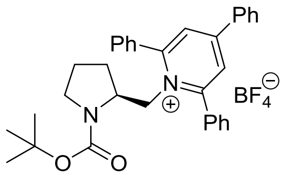

62a

Prepared following **GP1A** with 2,4,6-triphenylpyrylium tetrafluoroborate (1.63 g, 4 mmol), (*S*)-2-(aminomethyl)-1-Boc-pyrrolidine (909 mg, 4.40 mmol) and absolute EtOH (4 mL). The resulting solid pyridinium salt was filtered, washed with Et_2O (5 mL) and dried under high vacuum to give the desired product **62a** (2.29 g, 99% yield) as a white solid.

^{1}H NMR (400 MHz, $CDCl_3$) δ 8.03 – 7.91 (m, 3H), 7.80 (s, 2H), 7.72 (d, *J* = 6.9 Hz, 2H), 7.64 – 7.50 (m, 10H), 4.79 (dd, *J* = 14.2, 3.6 Hz, 1H), 4.64 (t, *J* = 12.9 Hz, 1H), 3.94 – 3.88 (m, 1H), 2.91 (dq, *J* = 10.4, 6.6 Hz, 1H), 2.66 (t, *J* = 9.3 Hz, 1H), 1.55 – 1.45 (m, 2H), 1.31 (s, 9H), 1.25 – 1.21 (m, 1H), 0.87 – 0.74 (m, 1H).

^{13}C NMR (125 MHz, DMSO-*d*$_6$, 100 °C) δ 156.8, 154.5, 154.1, 132.8, 132.7, 131.8, 130.4, 129.0, 129.0, 128.6, 128.0, 125.3, 79.1, 56.7, 54.7, 46.1, 27.5, 27.3, 22.3 (*4 aromatic carbon signals are not observed due to signal overlap*).

^{19}**F NMR** (377 MHz, $CDCl_3$) δ –153.05 (minor, $^{10}BF_4$), –153.10 (major, $^{11}BF_4$).

HRMS (ESI^+): calculated for $C_{33}H_{35}N_2O_2$ [M−BF_4]$^+$ 491.2693, found 491.2684.

IR (ν_{max}/cm^{-1}, neat) 3064, 2974, 2932, 2884, 1685, 1621, 1562, 1382, 1165, 1055, 703.

Synthesis of **66aa**:

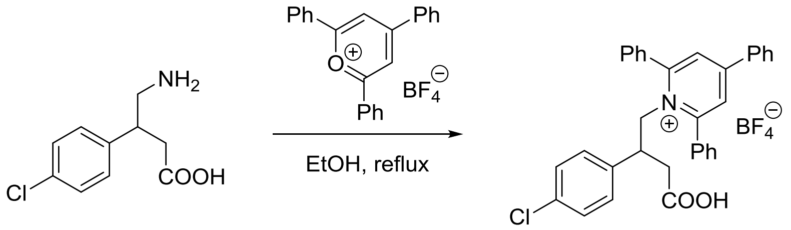

(±)-baclofen **66aa**

Prepared following **GP1A** with triphenylpyrylium tetrafluoroborate (1.19 g, 3.00 mmol), (±)-baclofen (770 mg, 3.6 mmol) and absolute EtOH (3.0 mL). The resulting solid pyridinium salt was filtered, washed with Et_2O (1 mL) and dried under high vacuum to give desired product **66aa** (965 mg, 54% yield) as a white solid.

^{1}**H NMR** (400 MHz, $CDCl_3$) δ 8.46–7.31 (m, 17H), 7.02 (d, J = 8.2 Hz, 2H), 6.30 (d, J = 8.2 Hz, 2H), 5.37 (br. s, 1H), 5.03 (dd, J = 14.5, 7.0 Hz, 1H), 4.85 (dd, J = 14.4, 7.7 Hz, 1H), 3.12 (p, J = 7.3 Hz, 1H), 2.24 – 2.09 (m, 2H).

^{13}**C NMR** (100 MHz, $CDCl_3$) δ 173.4, 157.5, 156.4, 136.7, 134.0, 133.6, 132.9, 132.6, 131.6, 129.9, 129.8, 129.4, 128.5, 128.4, 126.9, 59.4, 40.6, 37.0 (*1 aromatic carbon signal is not observed due to signal broadening*).

^{19}**F NMR** (377 MHz, $CDCl_3$) δ –151.91 (minor, $^{10}BF_4$), –151.97 (major, $^{11}BF_4$).

HRMS (ESI^+): calculated for $C_{33}H_{27}ClNO_2$ [M–BF_4]$^+$ 504.1725, found 504.1717.

IR (ν_{max}/cm^{-1}, neat) 3070, 3005, 2990, 1735, 1619, 1560, 1413, 1275, 1058, 764, 749.

Synthesis of **66a**:

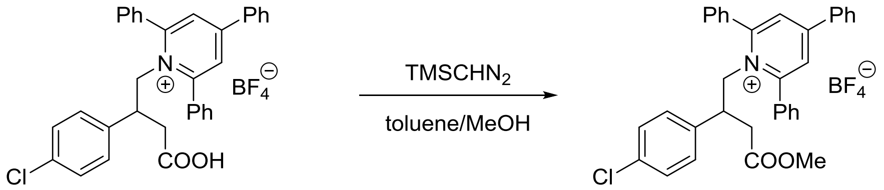

66aa **66a**

To a solution of **66aa** (296 mg, 0.500 mmol) in anhydrous toluene/MeOH (6.0 mL/1.5 mL), (trimethylsilyl)diazomethane (2.0 M in Et_2O, 1.2 mL, 2.4 mmol) was added dropwise at rt. After addition, the solution became yellow in colour. The reaction was stirred at rt for 1 h before concentration *in vacuo*. Purification by flash column chromatography (2–12% acetone/CH_2Cl_2) gave the desired product **66a** (258 mg, 86% yield) as an off-white solid.

^{1}**H NMR** (400 MHz, $CDCl_3$) δ 8.20–7.36 (m, 17H), 7.01 (d, J = 8.2 Hz, 2H), 6.34 (d, J = 8.2 Hz, 2H), 5.04 (dd, J = 14.6, 6.4 Hz, 1H), 4.92 (dd, J = 14.6, 8.1 Hz, 1H), 3.37 (s, 3H), 3.15 (p, J = 7.2 Hz, 1H), 2.24 – 2.02 (m, 2H).

^{13}**C NMR** (100 MHz, $CDCl_3$) δ 170.4, 157.5, 156.2, 136.6, 133.9, 133.6, 132.9, 132.6, 131.4, 129.9, 129.7, 129.3, 128.5, 128.2, 126.7, 59.2, 52.0, 41.0, 37.2 (*1 aromatic carbon signal is not observed due to signal broadening*).

^{19}**F NMR** (377 MHz, $CDCl_3$) δ –152.99 (minor, $^{10}BF_4$), –153.05 (major, $^{11}BF_4$).

HRMS (ESI^+): calculated for $C_{34}H_{29}ClNO_2Na$ $[M+Na]^+$ 518.1881, found 518.1872.

IR (ν_{max}/cm^{-1}, neat) 3006, 2989, 1736, 1619, 1275, 1260, 1055, 764, 760.

5.2. Radical acceptors

Synthesis of methyl 2-phenylacrylate:

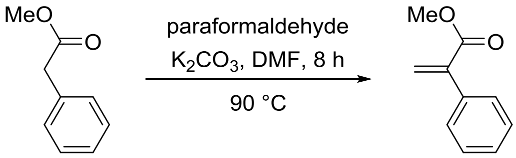

Methyl 2-phenylacrylate was prepared according to a literature procedure.[2] To a solution of methyl phenylacetate (2.0 mL, 14 mmol) and paraformaldehyde (664 mg, 22.1 mmol) in DMF (10 mL), K_2CO_3 (2.9 g, 21 mmol) was added. The solution was heated to 90 °C for 8 h, before being diluted with H_2O (25 mL), extracted with EtOAc (40 + 15 mL), washed with brine (15 mL), dried over $MgSO_4$, and concentrated *in vacuo*. The crude mixture was then purified by flash column chromatography (1–10% Et_2O/pentane) to give methyl 2-phenylacrylate (1.22g, 53% yield) as a colourless oil.

^{1}H NMR (400 MHz, $CDCl_3$) δ 7.46 – 7.31 (m, 5H), 6.37 (d, *J* = 1.2 Hz, 1H), 5.90 (d, *J* = 1.2 Hz, 1H), 3.83 (s, 3H).

^{13}C NMR (100 MHz, $CDCl_3$) δ 167.4, 141.5, 136.8, 128.4, 128.3, 128.3, 127.0, 52.3.

Spectroscopic data were consistent with those previously reported.[2]

Synthesis of allyl sulfone radical acceptor **52**:

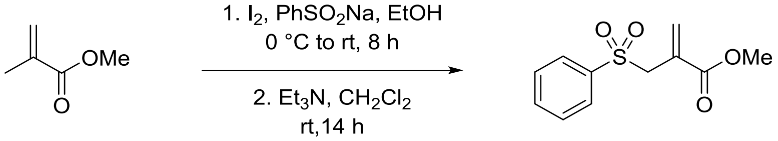

49a **49**

Following a literature procedure,[3] methyl methacrylate (3.08 mL, 29.0 mmol), I_2 (8.6 g, 34 mmol) and $PhSO_2Na$ (10 g, 61 mmol) in absolute EtOH (60 mL) were stirred at rt overnight. EtOH was removed *in vacuo* and the crude mixture was diluted with H_2O (40 mL) extracted with EtOAc (100 + 45 mL), washed with brine (60 mL), dried over $MgSO_4$, and concentrated *in vacuo* to give the crude product, which was used for the next step without further purification.

To the crude mixture, CH_2Cl_2 (30 mL) was added, followed by Et_3N (8.4 mL, 60 mmol), and the reaction was stirred at rt for 14 h before being concentrated *in vacuo*. The crude mixture was then purified by flash column chromatography (2–30% EtOAc/petroleum ether) to give desired product **49** (5.53g, 79% yield) as a white solid.

^{1}H NMR (500 MHz, $CDCl_3$) δ 7.89 – 7.84 (m, 2H), 7.67 – 7.63 (m, 1H), 7.57 – 7.52 (m, 2H), 6.51 (d, *J* = 0.6 Hz, 1H), 5.91 (q, *J* = 0.7 Hz, 1H), 4.16 (d, *J* = 0.8 Hz, 2H), 3.58 (s, 3H).

^{13}C NMR (125 MHz, $CDCl_3$) δ 165.4, 138.5, 134.0, 133.8, 129.2, 129.0, 128.9, 57.8, 52.5.

Spectroscopic data were consistent with those previously reported.[3]

Synthesis of radical acceptor **69a.**

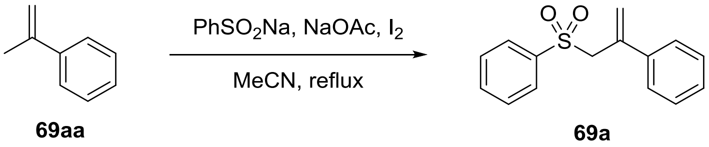

Following a literature procedure:[8] To a suspension of $PhSO_2Na$ (3.79 g, 23.1 mmol) and NaOAc (947 mg, 11.6 mmol) in MeCN (30 mL), 2-phenylpropene (1.00 mL, 7.70 mmol) was added followed by I_2 (2.93 g, 11.6 mmol). The mixture was heated at reflux for 3 h before allowing to cool to rt and quenching with saturated aq. sodium thiosulfate solution (5 mL). The mixture was basified with saturated aq. $NaHCO_3$ and extracted with EtOAc (3 × 20 mL). The combined organic phases were washed with H_2O, brine, dried over anhydrous Na_2SO_4, and concentrated *in vacuo*. The residue was purified by flash column chromatography (5–10% EtOAc/pentane) to give **69a** (1.61 g, 81% yield) as a white solid.

^{1}H NMR (400 MHz, $CDCl_3$) δ 7.81 (d, *J* = 7.4 Hz, 2H), 7.57 (t, *J* = 7.5 Hz, 1H), 7.45 (t, *J* = 7.7 Hz, 2H), 7.31 – 7.23 (m, 5H), 5.62 (s, 1H), 5.25 (s, 1H), 4.30 (s, 2H).

Spectroscopic data were consistent with those previously reported.[8]

Synthesis of radical acceptor **70a**:

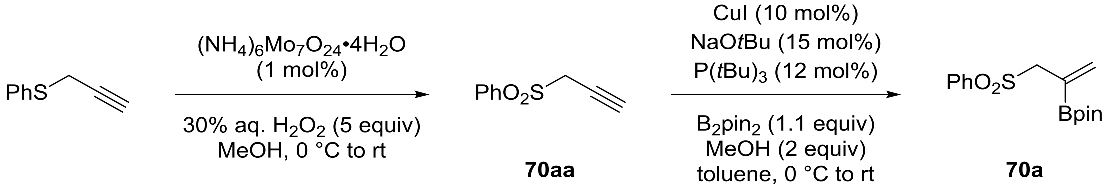

Following a literature procedure:[6] To a solution of phenyl propargyl sulfide (0.46 mL, 3.4 mmol) and $(NH_4)_6Mo_7O_{24}•4H_2O$ (42 mg, 1.0 mol%) in MeOH (5.0 mL) was added dropwise 30% aq. H_2O_2 (1.74 mL, 17.0 mmol) at 0 °C under an N_2 atmosphere. The reaction mixture was stirred at rt for 15 h before being extracted with $CHCl_3$ (2 × 20 mL) and combined organic layers were washed with H_2O (4 × 10 mL) and brine, dried over anhydrous Na_2SO_4 and concentrated to give **70aa** (600 mg, 99% yield) as a colourless solid, which was used in the next step without further purification.

^{1}H NMR (400 MHz, $CDCl_3$) δ 8.00 (d, *J* = 7.6 Hz, 2H), 7.71 (t, *J* = 7.5 Hz, 1H), 7.60 (t, *J* = 7.7 Hz, 2H), 3.96 (d, *J* = 2.7 Hz, 2H), 2.36 (t, *J* = 2.7 Hz, 1H).

Spectroscopic data were consistent with those previously reported.[6]

Following a literature procedure:[7] An oven dried Schlenk tube was charged with CuCl (17 mg, 0.17 mmol, 10 mol%), NaO*t*Bu (24 mg, 0.25 mmol, 15 mol%), bis(pinacolato)diboron (464 mg, 1.83 mmol) and **70aa** (300 mg, 1.66 mmol). The flask was evacuated and backfilled with N_2 three times. In another vial P(*t*Bu)$_3$ (40 mg, 0.20 mmol, 12 mol%) was dissolved in dry toluene (4 mL) under N_2. The mixture obtained was then transferred to the first Schlenk tube. Finally, MeOH (0.134 mL, 3.32 mmol) was added to the solution and the resulting mixture was stirred at rt for 1 h. The reaction was quenched with MeOH (2 mL), filtered through a pad of Celite, eluting with CH_2Cl_2, and the filtrate concentrated to *in vacuo*. Purification by flash column chromatography (20% EtOAc/petroleum ether) gave **70a** (262 mg, 50% yield) as a white solid.

^{1}H NMR (400 MHz, $CDCl_3$) δ 7.84 (d, *J* = 7.6 Hz, 2H), 7.61 (t, *J* = 7.4 Hz, 1H), 7.51 (t, *J* = 7.6 Hz, 2H), 6.09 (d, *J* = 2.5 Hz, 1H), 5.78 (s, 1H), 3.93 (s, 2H), 1.15 (s, 12H).

Spectroscopic data were consistent with those previously reported.[7]

Synthesis of alkynyl sulfone radical acceptor **71a**:

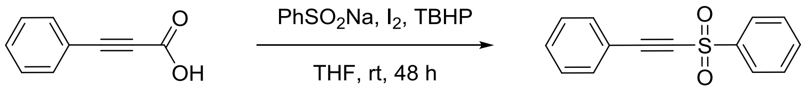

71aa **71a**

Following a literature procedure,[4] with phenylpropiolic acid **71aa** (597 mg, 4.08 mmol), $PhSO_2Na$ (1.34 g, 8.16 mmol), I_2 (508 mg, 2.00 mmol), TBHP (70% in H_2O, 1.66 mL, 12.0 mmol) and THF (16 mL). Purification by flash column chromatography (10–30% EtOAc/petroleum ether) gave **71a** (740 mg, 75% yield) as a white solid.

^{1}H NMR (400 MHz, $CDCl_3$) δ 8.09 (dt, *J* = 8.7, 1.9 Hz, 2H), 7.72 – 7.66 (m, 1H), 7.64 – 7.57 (m, 2H), 7.53 (dd, *J* = 8.3, 1.2 Hz, 2H), 7.50 – 7.45 (m, 1H), 7.37 (t, *J* = 7.6 Hz, 2H).

^{13}C NMR (100 MHz, $CDCl_3$) δ 142.0, 134.3, 132.9, 131.7, 129.5, 128.8, 127.5, 118.0, 93.6, 85.5.

Spectroscopic data were consistent with those previously reported.[4]

Synthesis of alkenyl sulfone radical acceptor **72a**:

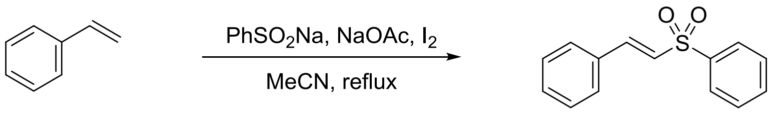

72a

Following a literature procedure,[5] with styrene (1.16 mL, 10.0 mmol), $PhSO_2Na$ (4.92 g, 30.0 mmol), NaOAc (1.23 g, 15.0 mmol), I_2 (3.81 g, 15.0 mmol) and MeCN (40 mL). Purification by flash column chromatography (10–30% EtOAc/petroleum ether) gave vinyl sulfone **72a** (2.02 g, 81% yield) as a white solid.

^{1}H NMR (400 MHz, $CDCl_3$) δ 7.96 (d, *J* = 7.7 Hz, 2H), 7.69 (d, *J* = 15.4 Hz, 1H), 7.62 (t, *J* = 7.4 Hz, 1H), 7.55 (t, *J* = 7.5 Hz, 2H), 7.51 – 7.45 (m, 2H), 7.44 – 7.35 (m, 3H), 6.87 (d, *J* = 15.4 Hz, 1H).

^{13}C NMR (126 MHz, $CDCl_3$) δ 142.6, 140.9, 133.5, 132.5, 131.4, 129.5, 129.2, 128.7, 127.8, 127.4.

Spectroscopic data were consistent with those previously reported.[5]

6. Product Characterization

6.1. Giese reactions

Synthesis of **6**:

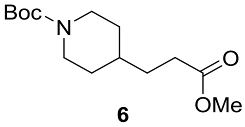

6

Prepared following **GP2**, with pyridinium **1a** (116 mg, 0.200 mmol), Hantzsch ester (152 mg, 0.600 mmol), methyl acrylate (24 µL, 0.26 mmol) and DMA (0.4 mL). Purification by flash column chromatography (4–40% EtOAc/hexane) gave **6** (42 mg, 77% yield) as a colourless oil.

^{1}H NMR (400 MHz, $CDCl_3$) δ 4.07 (br. s, 2H), 3.67 (s, 3H), 2.66 (t, 2H), 2.33 (t, 2H), 1.71 – 1.53 (m, 5H), 1.44 (s, 9H), 1.08 (qd, *J* = 12.5, 4.4 Hz, 2H).

^{13}C NMR (100 MHz, $CDCl_3$) δ 174.2, 155.0, 79.4, 51.7, 44.1 (br.), 35.6, 32.0, 31.5, 31.4, 28.6.

Spectroscopic data were consistent with those previously reported.[9]

Synthesis of **7**:

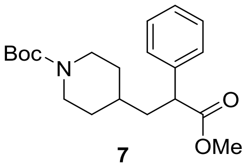

7

Prepared following **GP2**, with pyridinium **1a** (116 mg, 0.200 mmol), Hantzsch ester (152 mg, 0.600 mmol), methyl 2-phenylacrylate (42 mg, 0.26 mmol) and DMA (0.4 mL). Purification by flash column chromatography (1–35% Et_2O/pentane) gave **7** (61 mg, 88% yield) as a colourless oil.

^{1}H NMR (400 MHz, $CDCl_3$) δ 7.30 – 7.19 (m, 5H), 4.00 (br. s, 2H), 3.64 (t, *J* = 7.8 Hz, 1H), 3.61 (s, 3H), 2.56 (t, *J* = 12.6 Hz, 2H), 1.99 (dt, *J* = 14.9, 7.6 Hz, 1H), 1.73 – 1.56 (m, 3H), 1.40 (s, 9H), 1.30 – 1.23 (m, 1H), 1.06 (qd, *J* = 12.3, 4.4 Hz, 2H).

^{13}C NMR (100 MHz, $CDCl_3$) δ 174.5, 154.9, 139.1, 128.8, 128.0, 127.4, 79.4, 52.2, 48.7, 43.9, 40.2 (br.), 33.8, 32.2, 31.9, 28.6.

HRMS (ESI^+) calculated for $C_{20}H_{30}NO_4$ $[M+H]^+$ 348.2169, found 348.2172;

calculated for $C_{20}H_{29}NO_4Na$ $[M+Na]^+$ 370.1989, found 370.1998.

IR (ν_{max}/cm^{-1}, neat) 3086, 3061, 3029, 3004, 2975, 2932, 2849, 1735, 1689, 1423, 1365, 1276, 1161.

Synthesis of **7** (1 mmol scale):

Prepared following **GP2**, with pyridinium **1a** (578 mg, 1.00 mmol), Hantzsch ester (760 mg, 3.00 mmol), methyl 2-phenylacrylate (211 mg, 1.30 mmol) and DMA (2 mL). The reaction was stirred and irradiated with a blue LED lamp (under conditions shown in **GP3**) for 24 h. Purification by flash column chromatography (2–35% Et_2O/pentane) gave **7** (301 mg, 87% yield) as a colourless oil.

Synthesis of **8**:

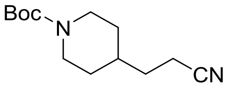

8

Prepared following **GP2**, with pyridinium **1a** (116 mg, 0.200 mmol), Hantzsch ester (152 mg, 0.600 mmol), acrylonitrile (17 µL, 0.26 mmol) and DMA (0.4 mL). Purification by flash column chromatography (5–30% EtOAc/toluene) gave **8** (31 mg, 65% yield) as a colourless oil.

^{1}H NMR (400 MHz, $CDCl_3$) δ 4.11 (br. s, 2H), 2.70 (t, *J* = 12.2 Hz, 2H), 2.38 (t, *J* = 7.1 Hz, 2H), 1.74 – 1.58 (m, 5H), 1.45 (s, 9H), 1.11 (qd, *J* = 12.8, 4.4 Hz, 2H).

^{13}C NMR (100 MHz, $CDCl_3$) δ 154.9, 119.7, 79.6, 43.6 (br.), 35.1, 31.9, 31.6, 28.6, 14.7.

HRMS (ESI$^+$) calculated for $C_{13}H_{22}N_2NaO_2$ [M+Na]$^+$ 261.1573, found 261.1582.

IR (ν_{max}/cm^{-1}, neat) 2927, 1689, 1247, 1166, 1066.

Synthesis of **9**:

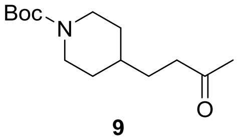

9

Prepared following **GP2**, with pyridinium **1a** (116 mg, 0.200 mmol), Hantzsch ester (152 mg, 0.600 mmol), methyl vinyl ketone (22 µL, 0.26 mmol) and DMA (0.4 mL). Purification by flash column chromatography (6–60% acetone/hexane) gave **9** (27 mg, 54% yield) as a colourless oil.

^{1}H NMR (400 MHz, $CDCl_3$) δ 4.06 (br. s, 2H), 2.64 (t, *J* = 12.7 Hz, 2H), 2.44 (t, *J* = 7.6 Hz, 2H), 2.13 (s, 3H), 1.62 (d, *J* = 12.9 Hz, 2H), 1.52 (q, *J* = 7.6 Hz, 2H), 1.44 (s, 9H), 1.41 – 1.30 (m, 1H), 1.07 (qd, *J* = 12.6, 4.4 Hz, 2H).

^{13}C NMR (100 MHz, $CDCl_3$) δ 208.9, 155.0, 79.4, 44.0 (br.), 40.9, 35.6, 32.1, 30.3, 30.0, 28.6;

HRMS (ESI$^+$) calculated for $C_{14}H_{25}NNaO_3$ [M+Na]$^+$ 278.1727, found 278.1719;

IR (ν_{max}/cm^{-1}, neat) 2927, 1689, 1365, 1160, 867.

Synthesis of **10**:

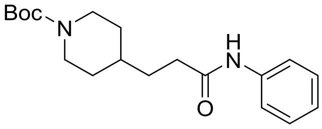

10

Prepared following **GP2**, with pyridinium **1a** (116 mg, 0.200 mmol), Hantzsch ester (152 mg, 0.600 mmol), *N*-phenylacrylamide (39 mg, 0.26 mmol) and DMA (0.4 mL). Purification by flash column chromatography (2–35% EtOAc/pentane) gave **10** (34 mg, 52% yield) as a white solid.

^{1}H NMR (400 MHz, $CDCl_3$) δ 7.70 (br. s, 1H), 7.55 – 7.47 (m, 2H), 7.34 – 7.27 (m, 2H), 7.08 (t, *J* = 7.3 Hz, 1H), 4.06 (br. s, 2H), 2.75 – 2.57 (m, 2H), 2.37 (t, *J* = 7.7 Hz, 2H), 1.65 – 1.64 (m, 4H), 1.50-1.39 (m, 1H), 1.45 (s, 9H), 1.09 (qd, *J* = 12.3, 4.1 Hz, 2H).

^{13}C NMR (100 MHz, $CDCl_3$) δ 171.4, 155.0, 138.2, 129.1, 124.3, 119.9, 79.5, 44.0 (br.), 35.6, 34.7, 32.0, 28.6.

HRMS (ESI$^+$) calculated for $C_{19}H_{29}N_2O_3$ [M+H]$^+$ 333.2173, found 333.2180;

calculated for $C_{19}H_{28}N_2O_3Na$ [M+Na]$^+$ 355.1992, found 355.2006.

IR (ν_{max}/cm^{-1}, neat) 3309 (br.), 3198, 3137, 3058, 2975, 2925, 2853, 1690, 1660, 1599, 1542, 1440, 1425, 1160.

Synthesis of **11**:

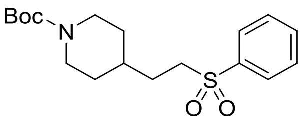

11

Prepared following **GP2**, with pyridinium **1a** (116 mg, 0.200 mmol), Hantzsch ester (152 mg, 0.600 mmol), phenyl vinyl sulfone (40 mg, 0.26 mmol) and DMA (0.4 mL). Purification by flash column chromatography (6–60% acetone/hexane) gave **11** (52 mg, 73% yield) as a colourless oil.

^{1}H NMR (400 MHz, $CDCl_3$) δ 7.93 – 7.87 (m, 2H), 7.69 – 7.64 (m, 1H), 7.61 – 7.54 (m, 2H), 4.05 (br. s, 2H), 3.14 – 3.05 (m, 2H), 2.62 (t, J = 12.8 Hz, 2H), 1.73 – 1.63 (m, 2H), 1.62 – 1.59 (m, 2H), 1.53 – 1.45 (m, 1H), 1.43 (s, 9H), 1.06 (qd, J = 12.5, 4.4 Hz, 2H).

^{13}C NMR (100 MHz, $CDCl_3$) δ 154.9, 139.3, 133.9, 129.5, 128.1, 79.6, 54.1, 43.8 (br.), 35.1, 31.8, 29.1, 28.6.

HRMS (ESI$^+$) calculated for $C_{18}H_{27}NNaO_4S$ [M+Na]$^+$ 376.1553, found 376.1600.

IR (ν_{max}/cm^{-1}, neat) 2930, 1588, 1423, 1245, 1087.

Synthesis of **12**:

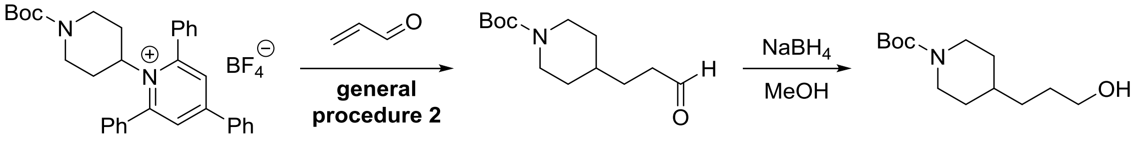

1a **12** **12b**

Prepared following **GP2**, with pyridinium **1a** (116 mg, 0.200 mmol), Hantzsch ester (152 mg, 0.600 mmol), acrolein (15 µL, 0.26 mmol) and DMA (0.4 mL). The crude mixture was then dissolved in MeOH (3 mL), cooled to 0 °C and sodium borohydride (76 mg, 10 equiv) was added. The reaction mixture was stirred for 1 h, then allowed to warm to rt. EtOAc (20 mL) was added and the organic phase was washed with H_2O (10 mL) and brine (10 mL), then dried over $MgSO_4$ and concentrated *in vacuo*. Purification by flash column chromatography (15–100% EtOAc/hexane) gave **12b** (26 mg, 52% yield) as a colourless oil.

^{1}H NMR (400 MHz, $CDCl_3$) δ 4.07 (br. s, 2H), 3.63 (t, J = 6.6 Hz, 2H), 2.67 (t, J = 12.3 Hz, 2H), 1.72 – 1.52 (m, 5H), 1.45 (s, 9H), 1.42 – 1.23 (m, 3H), 1.08 (qd, J = 12.6, 4.4 Hz, 2H).

^{13}C NMR (100 MHz, $CDCl_3$) δ 154.9, 119.7, 79.6, 43.9 (br.), 35.1, 31.9, 31.6, 28.6, 14.7.

Spectroscopic data were consistent with those previously reported.[10]

Synthesis of **13**:

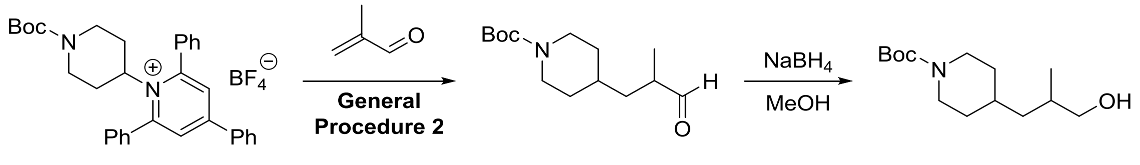

1a **13** **13b**

Prepared following **GP2**, with pyridinium **1a** (116 mg, 0.200 mmol), Hantzsch ester (152 mg, 0.600 mmol), methacrolein (30 µL, 0.36 mmol), DMA (0.4 mL), and stirring under irradiation with the fishing lamp at 60 °C for 40 h. The crude mixture was then dissolved in MeOH (3 mL), cooled to 0 °C and sodium borohydride (76 mg, 10 equiv) was added. The reaction mixture was stirred for 1 h, then allowed to warm to rt. EtOAc (20 mL) was added and the organic phases was washed with H_2O (10 mL) then brine (10 mL). The organic phases were then dried over $MgSO_4$ and concentrated *in vacuo*. Purification by flash column chromatography (15–100% EtOAc/hexane) gave **13b** (37 mg, 68% yield) as a colourless oil.

^{1}**H NMR** (400 MHz, $CDCl_3$) δ 4.07 (br. s, 2H), 3.55 – 3.36 (m, 2H), 2.71 – 2.64 (m, 2H), 1.79 – 1.69 (m, 1H), 1.69 – 1.58 (m, 3H), 1.45 (s, 9H), 1.33 – 1.22 (m, 2H), 1.15 – 0.96 (m, 3H), 0.92 (d, *J* = 6.7 Hz, 3H).

^{13}**C NMR** (100 MHz, $CDCl_3$) 155.0, 79.3, 68.7, 44.3 (br.), 40.3, 33.4, 33.1, 32.6, 32.0, 28.6, 16.9.

HRMS (ESI^+) calculated for $C_{14}H_{27}NNaO_3$ $[M+Na]^+$ 280.1883, found 280.1886.

IR (ν_{max}/cm^{-1}, neat) 3444, 2923, 1694, 1425, 1366.

Synthesis of **14**:

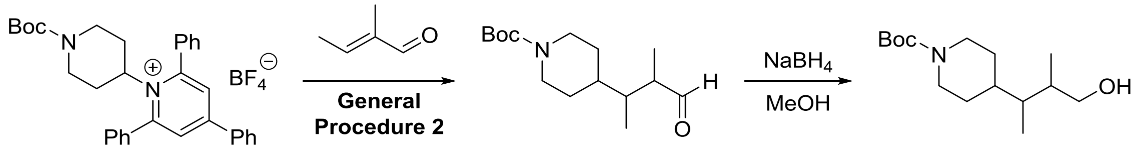

1a **14** **14b**

Prepared following **GP2**, with pyridinium **1a** (116 mg, 0.200 mmol), Hantzsch ester (152 mg, 0.600 mmol), tiglic aldehyde (35 µL, 0.36 mmol), DMA (0.4 mL), and stirring under irradiation with the fishing lamp at 60 °C for 40 h. The crude mixture was then dissolved in MeOH (3 mL), cooled to 0 °C and sodium borohydride (76 mg, 10 equiv) was added. The reaction mixture was stirred for 1 h, then allowed to warm to rt. EtOAc (20 mL) was added and the organic phases was washed with H_2O (10 mL) then brine (10 mL). The organic phases were then dried over $MgSO_4$ and concentrated *in vacuo*. Purification by flash column chromatography (1–10% acetone/CH_2Cl_2) gave **14b** (17 mg, 32% yield, 50:50 d.r.) as a colourless oil.

^{1}**H NMR** (400 MHz, $CDCl_3$) δ (*50:50 mixture of diastereomers*) 4.25 – 3.95 (m, 2H), 3.68 (dd, J = 10.5, 4.1 Hz, 0.5H), 3.57 – 3.42 (m, 1.5H), 2.64 (br. s, 2H), 1.88 – 1.80 (m, 0.5H), 1.72 – 1.67 (m, 1.5H), 1.53 – 1.50 (m, 2H), 1.45 (s, 9H), 1.45 – 1.38 (m, 0.5H), 1.34 – 1.24 (m, 2H), 1.17 – 1.08 (s, 1.5H), 1.00 (d, J = 6.8 Hz, 1.5H), 0.81 (m, 3H), 0.76 (d, J = 6.9 Hz, 1.5H).

^{13}**C NMR** (126 MHz, $CDCl_3$) δ (*50:50 mixture of diastereomers*) 155.0, 79.4, 67.2, 65.8, 40.4, 39.1, 38.4, 37.8, 37.4, 36.3, 28.6, 16.2, 12.4, 11.4, 11.2 (*7 carbon signals are unresolved from the corresponding signals in the other diastereomer*).

HRMS (ESI$^+$): calculated for $C_{15}H_{29}NNaO_3$ [M+Na]$^+$ 294.2040, found 294.2038.

IR (ν_{max}/cm^{-1}, neat) 2927, 1694, 1428, 1172, 1028.

Synthesis of **15**:

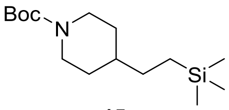

15

Prepared following **GP2**, with pyridinium **1a** (116 mg, 0.200 mmol), Hantzsch ester (203 mg, 0.800 mmol), vinyl trimethylsilane (88 µL, 0.60 mmol) and DMA (0.4 mL), stirring for 40 h. Purification by flash column chromatography (1–15% EtOAc/toluene) gave **15** (13 mg, 23% yield) as a colourless oil.

^{1}H NMR (400 MHz, $CDCl_3$) δ 4.07 (br. s, 2H), 2.65 (t, J = 12.8 Hz, 2H), 1.67 (d, J = 13.0 Hz, 2H), 1.45 (s, 9H), 1.24 – 1.18 (m, 3H), 1.03 (qd, J = 12.7, 4.4 Hz, 2H), 0.55 – 0.38 (m, 2H), -0.03 (s, 9H).

^{13}C NMR (100 MHz, $CDCl_3$) δ 155.1, 79.3, 44.3 (br.), 39.2, 32.1, 30.7, 28.6, 13.5, -1.6.

HRMS (ESI$^+$): calculated for $C_{15}H_{31}NNaO_2Si$ [M+Na]$^+$ 308.2016, found 308.2017.

IR (ν_{max}/cm^{-1}, neat) 2918, 1696, 1422, 1237, 1156.

Synthesis of **16**:

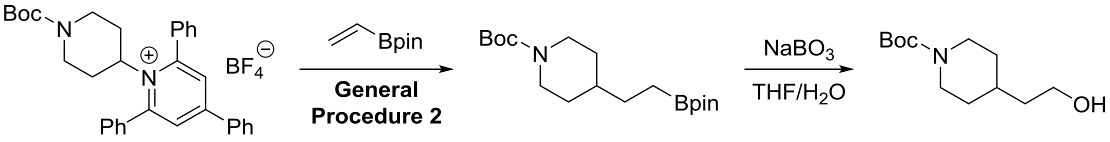

1a **16** **16b**

Prepared following **GP2**, with pyridinium **1a** (116 mg, 0.200 mmol), Hantzsch ester (152 mg, 0.600 mmol), vinylboronic acid pinacol ester (61 µL, 0.26 mmol) and DMA (0.4 mL), stirring for 40 h. The crude mixture was then dissolved in THF/H_2O (1:1, 3 mL), sodium perborate (40 mg, 0.40 mmol) was added and the suspension was stirred overnight at rt. The reaction mixture was diluted with H_2O (7 mL), extracted with EtOAc (3 × 10 mL), then the combined organic extracts were dried over $MgSO_4$ and concentration *in vacuo*. Purification by flash column chromatography (15–100% EtOAc/hexane) gave **16b** (20 mg, 40% yield) as a colourless oil.

^{1}H NMR (400 MHz, $CDCl_3$) δ 4.06 (br. s, 2H), 3.70 (t, J = 6.5 Hz, 2H), 2.69 (t, J = 12.8 Hz, 2H), 1.71 – 1.55 (m, 4H), 1.51 (q, J = 6.7 Hz, 2H), 1.45 (s, 9H), 1.12 (qd, J = 12.7, 4.4 Hz, 2H).

^{13}C NMR (100 MHz, $CDCl_3$) δ 155.0, 79.4, 60.4, 44.1 (br.), 39.4, 32.7, 32.3, 28.6.

Spectroscopic data were consistent with those previously reported.[11]

Synthesis of **17**:

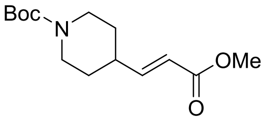

17

Prepared following **GP2**, with pyridinium **1a** (116 mg, 0.200 mmol), Hantzsch ester (156 mg, 0.200 mmol), methyl propiolate (41 µL, 0.46 mmol) and DMA (0.4 mL), stirring for 40 h. Purification by flash column chromatography (1–15% EtOAc/pentane) gave *E*-**17** (17 mg, 30% yield) as a colourless oil. The isomer *Z*-**17** could not be separated from the oxidised Hantzsch ester by-product, however, ^{1}H NMR analysis of the mixture established the yield to be 20%.

^{1}H NMR (400 MHz, $CDCl_3$) δ 6.90 (dd, *J* = 15.8, 6.7 Hz, 1H), 5.80 (dd, *J* = 15.8, 1.5 Hz, 1H), 4.11 (br. s, 2H), 3.73 (s, 3H), 2.76 (t, *J* = 12.8 Hz, 2H), 2.36 – 2.21 (m, 1H), 1.72 (d, *J* = 12.2 Hz, 2H), 1.45 (s, 9H), 1.34 (qd, *J* = 12.3, 4.3 Hz, 2H).

^{13}C NMR (100 MHz, $CDCl_3$) δ 167.2, 154.9, 152.1, 119.8, 79.7, 51.7, 43.5 (br.), 38.8, 30.8, 28.6.

HRMS (ESI^+): calculated for $C_{14}H_{23}NO_4Na$ $[M+Na]^+$ 292.1519, found 292.1529.

IR (ν_{max}/cm^{-1}, neat) 3007, 2975, 2928, 2856, 1725, 1692, 1423, 1365, 1276, 1166.

Synthesis of **18**:

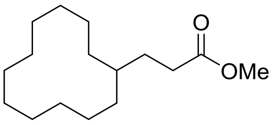

18

Prepared following **GP2**, with pyridinium **18a** (111 mg, 0.200 mmol), Hantzsch ester (152 mg, 0.600 mmol), methyl acrylate (24 µL, 0.26 mmol) and DMA (0.4 mL). Purification by flash column chromatography (2–15% EtOAc/toluene) gave **18** (45 mg, 90% yield) as a colourless oil.

^{1}H NMR (400 MHz, $CDCl_3$) δ 3.66 (s, 3H), 2.36 – 2.30 (m, 2H), 1.58 – 1.49 (m, 2H), 1.42 – 1.19 (m, 23H).

^{13}C NMR (100 MHz, $CDCl_3$) δ 174.8, 51.6, 33.8, 32.4, 30.1, 28.9, 24.9, 24.3, 23.5, 23.4, 21.7.

HRMS (ESI^+): calculated for $C_{16}H_{30}NaO_2$ $[M+Na]^+$ 277.2138, found 277.2139.

IR (ν_{max}/cm^{-1}, neat) 2930, 2862, 1741, 1470, 1167.

Synthesis of **19**:

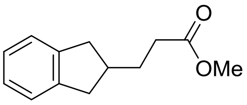

19

Prepared following **GP2**, with pyridinium **19a** (104 mg, 0.200 mmol), Hantzsch ester (152 mg, 0.600 mmol), methyl acrylate (24 µL, 0.26 mmol) and DMA (0.4 mL). Purification by flash column chromatography (5–40% EtOAc/toluene) gave **19** (29 mg, 70% yield) as a purple oil.

^{1}H NMR (400 MHz, $CDCl_3$) δ 7.19 (dd, *J* = 5.4, 3.4 Hz, 2H), 7.13 (dt, *J* = 5.2, 3.6 Hz, 2H), 3.69 (s, 3H), 3.06 (dd, *J* = 15.4, 8.1 Hz, 2H), 2.61 (dd, *J* = 15.4, 8.1 Hz, 2H), 2.53 – 2.37 (m, 3H), 1.86 (q, *J* = 7.5 Hz, 2H).

^{13}C NMR (100 MHz, $CDCl_3$) δ 174.2, 143.2, 126.3, 124.5, 51.7, 39.8, 39.1, 33.1, 30.9.

HRMS (ESI^+): calculated for $C_{13}H_{16}NaO_2$ [*M*+Na]$^+$ 227.1043, found 227.1046.

IR (ν_{max}/cm^{-1}, neat) 2928, 1691, 1425, 1246, 1066.

Synthesis of **20**:

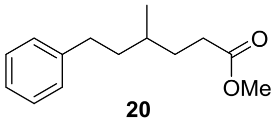

20

Prepared following **GP2**, with pyridinium **20a** (108 mg, 0.210 mmol), Hantzsch ester (156 mg, 0.620 mmol), methyl acrylate (24 µL, 0.26 mmol) and DMA (0.4 mL). Purification by flash column chromatography (1–15% EtOAc/toluene) gave **20** (29 mg, 65% yield) as a colourless oil.

^{1}H NMR (400 MHz, $CDCl_3$) δ 7.30 – 7.26 (m, 2H), 7.20 – 7.15 (m, 3H), 3.67 (s, 3H), 2.74 – 2.55 (m, 2H), 2.40 – 2.26 (m, 2H), 1.78 – 1.61 (m, 2H), 1.55 – 1.42 (m, 3H), 0.95 (d, *J* = 6.1 Hz, 3H).

^{13}C NMR (100 MHz, $CDCl_3$) δ 174.6, 142.9, 128.5, 128.4, 125.8, 51.6, 38.7, 33.4, 32.2, 31.9, 31.9, 19.3.

HRMS (ESI^+): calculated for $C_{14}H_{20}NaO_2$ [*M*+Na]$^+$ 243.1356, found 243.1357.

IR (ν_{max}/cm^{-1}, neat) 3026, 2952, 2861, 1738, 1603, 1253;

Synthesis of **21**:

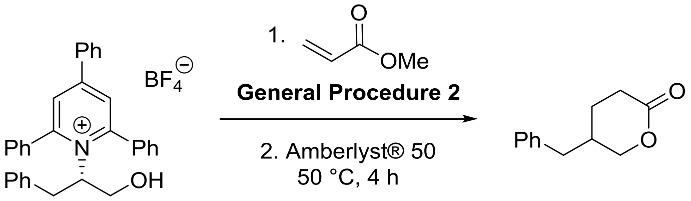

21a **21**

Prepared following **GP2**, with pyridinium **21a** (109 mg, 0.210 mmol), Hantzsch ester (156 mg, 0.620 mmol), methyl acrylate (24 µL, 0.26 mmol) and DMA (0.4 mL). Before work-up, DMA (2 mL) and Amberlyst® 50 were added and the reaction mixture was stirred for 4 h at 50 °C. Purification by flash column chromatography (20% EtOAc/pentane) gave **21** (19 mg, 49% yield) as a colourless oil.

^{1}H NMR (400 MHz, $CDCl_3$) δ 7.38 – 7.29 (m, 2H), 7.29 – 7.20 (m, 1H), 7.20 – 7.08 (m, 2H), 4.31 (ddd, *J* = 11.2, 4.4, 1.9 Hz, 1H), 4.02 (dd, *J* = 11.2, 9.3 Hz, 1H), 2.72 – 2.56 (m, 3H), 2.49 (ddd, *J* = 17.7, 9.7, 7.4 Hz, 1H), 2.30 – 2.15 (m, 1H), 2.09 – 1.91 (m, 1H), 1.67 – 1.59 (m, 1H).

^{13}C NMR (100 MHz, $CDCl_3$) δ 171.4, 138.5, 129.0, 128.8, 126.8, 73.2, 38.1, 34.8, 29.1, 25.4.

Spectroscopic data were consistent with those previously reported.[12]

Synthesis of **22**:

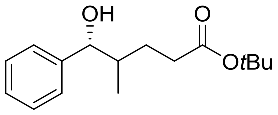

22

Prepared following **GP2**, with pyridinium **22a** (106 mg, 0.200 mmol), Hantzsch ester (152 mg, 0.600 mmol), *tert*-butyl acrylate (38 µL, 0.26 mmol) and DMA (0.4 mL). Purification by preparative TLC (60% EtOAc/hexane) gave **22** (27 mg, 52% yield, 56:44 d.r.) as a colourless oil.

^{1}H NMR (400 MHz, $CDCl_3$) δ (*56:44 mixture of diastereomers, asterisk denotes signals arising from minor diastereomer*) 7.40 – 7.20 (m, 11H), 4.57 (d, *J* = 5.0 Hz, 0.8H)*, 4.40 (d, *J* = 7.3 Hz, 1H), 2.47 – 1.91 (m, 7H), 1.91 – 1.50 (m, 6H), 1.44 (s, 9H), 1.43 (s, 7.7H)*, 0.89 (d, *J* = 6.7 Hz, 3H), 0.75 (d, *J* = 6.8 Hz, 2.8H)*.

^{13}C NMR (101 MHz, $CDCl_3$) δ (*56:44 mixture of diastereomers, asterisk denotes signals arising from minor diastereomer*) 173.5*, 173.4, 143.5, 143.4*, 128.34, 128.28*, 127.6, 127.3*, 126.8, 126.3*, 80.3, 80.2*, 78.9*, 77.3*, 39.9*, 39.8, 33.5*, 33.2, 29.8*, 28.3*, 28.2, 27.6, 15.8*, 14.1.

HRMS (ESI$^+$): calculated for $C_{16}H_{24}NaO_3$ [*M*+Na]$^+$ 287.1618, found 287.1622.

IR (ν_{max}/cm^{-1}, neat) 2976, 2927, 1725, 1622, 1454, 1058.

Synthesis of **23**:

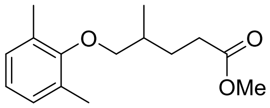

23

Prepared following **GP2**, with pyridinium **23a** (112 mg, 0.200 mmol), Hantzsch ester (152 mg, 0.600 mmol), methyl acrylate (24 µL, 0.26 mmol) and DMA (0.4 mL). Purification by flash column chromatography (0–40% EtOAc/toluene) gave **23** (17 mg, 34% yield) as a colourless oil.

^{1}H NMR (400 MHz, $CDCl_3$) δ 7.00 (d, *J* = 7.4 Hz, 2H), 6.90 (dd, *J* = 8.2, 6.7 Hz, 1H), 3.69 (s, 3H), 3.66 – 3.50 (m, 2H), 2.55 – 2.37 (m, 2H), 2.27 (s, 6H), 2.03 – 1.93 (m, 2H), 1.73 – 1.59 (m, 1H), 1.10 (d, *J* = 6.5 Hz, 3H).

^{13}C NMR (100 MHz, $CDCl_3$) δ 174.3, 155.9, 131.0, 129.0, 123.8, 76.7, 51.7, 34.1, 32.1, 28.9, 16.9, 16.4.

HRMS (ESI$^+$): calculated for $C_{15}H_{22}NaO_3$ [*M*+Na]$^+$ 251.1642, found 251.1642.

IR (ν_{max}/cm^{-1}, neat) 2984, 1739, 1436, 1384, 1092.

Synthesis of **24**:

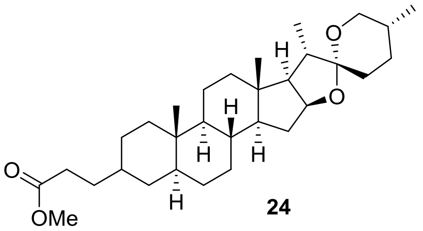

24

Prepared following **GP2**, with pyridinium **24a** (159 mg, 0.200 mmol), Hantzsch ester (152 mg, 0.600 mmol), methyl acrylate (24 µL, 0.26 mmol) and DMA (0.4 mL). Purification by flash column chromatography (2–15% EtOAc/hexane) gave **24** (50 mg, 51% yield, 67:33 d.r.) as a white solid.

^{1}H NMR (500 MHz, $CDCl_3$) δ (*67:33 mixture of diastereomers*) 4.40 (q, *J* = 7.3 Hz, 1H), 3.68 (s, 3H), 3.47 (dd, *J* = 4.6, 1.8 Hz, 1H), 3.39 (t, *J* = 10.9 Hz, 1H), 2.30 (dd, *J* = 8.8, 6.8 Hz, 2H), 1.99 (ddd, *J* = 12.4, 7.5, 5.4 Hz, 1H), 1.87 (p, *J* = 7.5, 6.9 Hz, 1H), 1.79 – 1.34 (m, 17H), 1.30 – 1.06 (m, 9H), 0.97 (d, *J* = 6.9 Hz, 3H), 0.95 – 0.89 (m, 1H), 0.81 (s, 3H), 0.80 (d, *J* = 6.9 Hz, 3H), 0.77 (s, 3H), 0.71 (td, *J* = 12.2, 4.0 Hz, 1H).

^{13}C NMR (125 MHz, $CDCl_3$) δ (*67:33 mixture of diastereomers, asterisk denotes signals arising from minor diastereomer*) 174.7, 109.4, 81.0, 67.0, 62.3, 56.6, 56.5*, 54.83, 54.75* 51.6, 46.6*, 41.7, 40.7, 40.4, 40.3, 38.5*, 37.7*, 36.7, 36.3*, 35.4*, 35.28*, 35.26*, 32.4* 33.2, 33.0, 32.9, 32.7, 32.4, 31.9, 31.5, 30.4, 29.00*, 28.95, 28.7*, 27.3, 25.4, 21.0*, 20.7, 17.3, 16.7, 14.6, 12.5*, 11.9 (*all remaining signals are unresolved of the corresponding signals in the other diastereomer*).

HRMS (ESI^+): calculated for $C_{31}H_{52}NaO_4$ $[M+Na]^+$ 487.3782, found 487.3780.

IR (ν_{max}/cm^{-1}, neat) 2992, 2855, 1740, 1652, 981, 898, 732.

Synthesis of **25**:

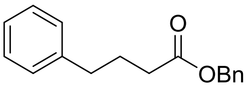

25

Prepared following **GP2**, with pyridinium **25a** (101 mg, 0.208 mmol), Hantzsch ester (158 mg, 0.624 mmol), benzyl acrylate (42 µL, 0.26 mmol) and DMA (0.4 mL). Purification by flash column chromatography (100% toluene) gave **25** (27 mg, 51% yield) as a colourless oil.

^{1}H NMR (400 MHz, $CDCl_3$) δ 7.39 – 7.31 (m, 5H), 7.30 – 7.24 (m, 2H), 7.21 – 7.12 (m, 3H), 5.11 (s, 2H), 2.71 – 2.58 (m, 2H), 2.37 (t, *J* = 7.5 Hz, 2H), 1.97 (p, *J* = 7.5 Hz, 2H).

^{13}C NMR (100 MHz, $CDCl_3$) δ 173.4, 141.5, 136.2, 128.7, 128.6, 128.5, 128.4, 128.4, 126.1, 66.3, 35.3, 33.8, 26.7 (*1 aromatic carbon signal is not observed due to overlap).*

Spectroscopic data were consistent with those previously reported.[13]

Synthesis of **26**:

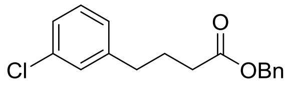

26

Prepared following **GP2**, with pyridinium **26a** (104 mg, 0.200 mmol), Hantzsch ester (152 mg, 0.600 mmol), benzyl acrylate (40 µL, 0.26 mmol) and DMA (0.4 mL). Purification by flash column chromatography (100% toluene) gave **26** (25 mg, 43% yield) as a colourless oil.

^{1}H NMR (400 MHz, $CDCl_3$) δ 7.42 – 7.29 (m, 5H), 7.25 – 7.11 (m, 3H), 7.03 (d, *J* = 7.0 Hz, 1H), 5.12 (s, 2H), 2.70 – 2.58 (m, 2H), 2.38 (t, *J* = 7.4 Hz, 2H), 2.03 – 1.90 (m, 2H).

^{13}C NMR (101 MHz, $CDCl_3$) δ 173.2, 143.5, 136.1, 134.3, 129.8, 128.8, 128.7, 128.4, 126.8, 126.4, 66.4, 34.9, 33.6, 26.4 (*1 aromatic carbon signal is not observed due to overlap*).

HRMS (ESI^+): calculated for $C_{17}H_{17}ClNaO_2$ $[M+Na]^+$ 311.0809, found 311.0822.

IR (ν_{max}/cm^{-1}, neat) 3033, 2928, 1733, 1455, 1143, 697.

Synthesis of **27**:

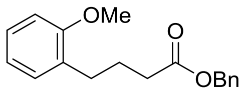

27

Prepared following **GP2**, with pyridinium **27a** (103 mg, 0.200 mmol), Hantzsch ester (152 mg, 0.600 mmol), benzyl acrylate (40 µL, 0.26 mmol) and DMA (0.4 mL). Purification by flash column chromatography (100% toluene) gave **27** (35 mg, 68% yield) as a colourless oil.

^{1}H NMR (400 MHz, $CDCl_3$) δ 7.45 – 7.31 (m, 5H), 7.19 (td, *J* = 7.9, 1.7 Hz, 1H), 7.11 (dd, *J* = 7.4, 1.7 Hz, 1H), 6.88 (td, *J* = 7.4, 1.0 Hz, 1H), 6.84 (d, *J* = 8.2 Hz, 1H), 5.12 (s, 2H), 3.80 (s, 3H), 2.75 – 2.59 (m, 2H), 2.40 (t, *J* = 7.6 Hz, 2H), 1.97 (p, *J* = 7.6 Hz, 2H).

^{13}C NMR (100 MHz, $CDCl_3$) δ 173.7, 157.6, 136.3, 130.2, 129.9, 128.7, 128.3, 128.3, 127.4, 120.5, 110.3, 66.2, 55.3, 34.0, 29.7, 25.1.

HRMS (ESI^+): calculated for $C_{18}H_{20}NaO_3$ $[M+Na]^+$ 307.1305, found 307.1315.

IR (ν_{max}/cm^{-1}, neat) 2936, 1732, 1493, 1029, 751.

Synthesis of **28**:

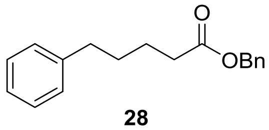

28

Prepared following **GP3**, with pyridinium **28a** (100 mg, 0.200 mmol), Hantzsch ester (152 mg, 0.600 mmol), benzyl acrylate (64 µL, 0.4 mmol), Et_3N (84 µL, 0.60 mmol) and DMA (0.4 mL). Purification by flash column chromatography (1–8% Et_2O/pentane) gave **28** (20 mg, 37% yield) as a colourless oil.

^{1}H NMR (400 MHz, $CDCl_3$) δ 7.48 – 7.39 (m, 5H), 7.38 – 7.32 (m, 2H), 7.29 – 7.21 (m, 3H), 5.20 (s, 2H), 2.71 (t, *J* = 7.2 Hz, 2H), 2.47 (t, *J* = 7.1 Hz, 2H), 1.83 – 1.70 (m, 4H).

^{13}C NMR (100 MHz, $CDCl_3$) δ 173.5, 142.2, 136.2, 128.7, 128.5, 128.4, 128.3, 125.9, 66.3, 35.7, 34.3, 31.0, 24.7.

Spectroscopic data were consistent with those previously reported.[14]

Synthesis of **29**:

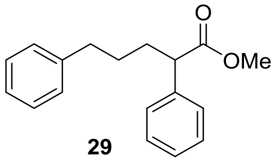

29

Prepared following **GP3**, with pyridinium **28a** (100 mg, 0.200 mmol), Hantzsch ester (152 mg, 0.600 mmol), methyl 2-phenylacrylate (42 mg, 0.26 mmol), Et_3N (84 µL, 0.60 mmol) and DMA (0.5 mL). Purification by flash column chromatography (1–5% Et_2O/pentane) gave **29** (25 mg, 47% yield) as a colourless oil.

^{1}H NMR (400 MHz, $CDCl_3$) δ 7.34 – 7.26 (m, 5H), 7.26 – 7.22 (m, 2H), 7.20 – 7.10 (m, 3H), 3.64 (s, 3H), 3.56 (t, *J* = 7.7 Hz, 1H), 2.69 – 2.55 (m, 2H), 2.17 – 2.06 (m, 1H), 1.87 – 1.78 (m, 1H), 1.67 – 1.49 (m, 2H).

^{13}C NMR (125 MHz $CDCl_3$) δ 174.6, 142.1, 139.2, 128.8, 128.5, 128.4, 128.0, 128.0, 127.4, 125.9, 52.1, 51.7, 35.8, 33.2, 29.5.

HRMS (ESI^+): calculated for $C_{18}H_{21}O_2$ $[M+H]^+$ 269.1536, found 269.1549.

IR (ν_{max}/cm^{-1}, neat) 3083, 3061, 3029, 2946, 2860, 1733, 1495, 1453, 1209, 1164.

Synthesis of **30**:

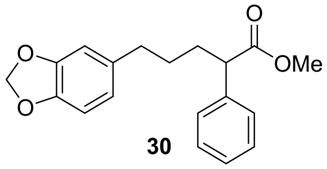

30

Prepared following **GP3**, with pyridinium **30a** (109 mg, 0.200 mmol), Hantzsch ester (152 mg, 0.600 mmol), methyl 2-phenylacrylate (42 mg, 0.26 mmol), Et_3N (84 µL, 0.60 mmol) and DMA (0.8 mL). Purification by flash column chromatography (1–10% Et_2O/pentane) gave **30** (31mg, 50% yield) as a colourless oil.

^{1}H NMR (400 MHz, $CDCl_3$) δ 7.33 – 7.21 (m, 5H), 6.69 (d, *J* = 7.8 Hz, 1H), 6.63 – 6.54 (m, 2H), 5.89 (s, 2H), 3.64 (s, 3H), 3.54 (t, *J* = 7.7 Hz, 1H), 2.60 – 2.46 (m, 2H), 2.08 (dddd, *J* = 13.4, 10.3, 7.9, 5.6 Hz, 1H), 1.79 (dddd, *J* = 13.4, 10.1, 7.5, 5.6 Hz, 1H), 1.59 – 1.45 (m, 2H).

^{13}C NMR (100 MHz, $CDCl_3$) δ 174.6, 147.6, 145.7, 139.1, 136.0, 128.8, 128.0, 127.4, 121.2, 108.9, 108.2, 100.8, 52.1, 51.6, 35.5, 33.1, 29.7.

HRMS (ESI^+): calculated for $C_{19}H_{21}O_4$ $[M+H]^+$ 313.1434, found 313.1435;

calculated for $C_{19}H_{20}O_4Na$ $[M+Na]^+$ 335.1254, found 335.1249.

IR (ν_{max}/cm^{-1}, neat) 3083, 3061, 3029, 3004, 2946, 2860, 1733, 1502, 1488, 1441, 1243, 1166, 1039.

Synthesis of **31**:

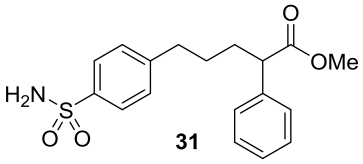

31

Prepared following **GP3**, with pyridinium **31a** (116 mg, 0.200 mmol), Hantzsch ester (152 mg, 0.600 mmol), methyl 2-phenylacrylate (42 mg, 0.26 mmol), Et_3N (84 µL, 0.60 mmol) and DMA (0.8 mL). Purification by flash column chromatography (5–40% EtOAc/petroleum ether) gave **31** (43 mg, 62% yield) as a white solid.

^{1}H NMR (400 MHz, $CDCl_3$) δ 7.86 – 7.77 (m, 2H), 7.36 – 7.23 (m, 7H), 4.94 (br. s, 2H), 3.64 (s, 3H), 3.55 (t, *J* = 7.7 Hz, 1H), 2.68 (td, *J* = 8.0, 3.2 Hz, 2H), 2.10 (dddd, *J* = 13.3, 10.2, 7.9, 5.5 Hz, 1H), 1.80 (dddd, *J* = 13.3, 10.2, 7.5, 5.5 Hz, 1H), 1.68 – 1.50 (m, 2H).

^{13}C NMR (125 MHz, $CDCl_3$) δ 174.4, 147.6, 139.6, 138.9, 129.2, 128.8, 128.0, 127.5, 126.7, 52.2, 51.5, 35.6, 33.0, 29.0.

HRMS (ESI$^+$): calculated for $C_{18}H_{21}NO_4Na$ [M+Na]$^+$ 370.1083, found 370.1087.

IR (ν_{max}/cm^{-1}, neat) 3352 (br.), 3263(br.), 3100, 3090, 3065, 3029, 2946, 2860, 1720, 1333, 1159, 1096.

Synthesis of **32**:

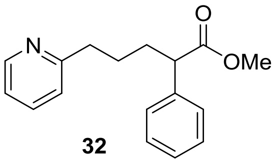

32

Prepared following **GP3**, with pyridinium **32a** (100 mg, 0.200 mmol), Hantzsch ester (152 mg, 0.600 mmol), methyl 2-phenylacrylate (42 mg, 0.26 mmol), Et_3N (84 µL, 0.60 mmol) and DMA (0.5 mL). Purification by flash column chromatography (10–50% Et_2O/pentane) gave **32** (26 mg, 49% yield) as a colourless oil.

^{1}H NMR (400 MHz, $CDCl_3$) δ 8.49 (ddd, *J* = 4.8, 1.8, 1.0 Hz, 1H), 7.56 (td, *J* = 7.7, 1.9 Hz, 1H), 7.33 – 7.26 (m, 4H), 7.26 – 7.21 (m, 1H), 7.11 – 7.04 (m, 2H), 3.64 (s, 3H), 3.58 (t, *J* = 7.6 Hz, 1H), 2.85 – 2.73 (m, 2H), 2.18 – 2.09 (m, 1H), 1.89 – 1.81 (m, 1H), 1.79 – 1.60 (m, 2H).

^{13}C NMR (100 MHz, $CDCl_3$) δ 174.5, 161.7, 149.4, 139.1, 136.4, 128.7, 128.0, 127.4, 122.8, 121.2, 52.1, 51.7, 38.2, 33.3, 27.8.

HRMS (ESI$^+$): calculated for $C_{17}H_{20}NO_2$ [M+H]$^+$ 270.1489, found 270.1501.

IR (ν_{max}/cm^{-1}, neat) 3083, 3061, 3029, 3007, 2950, 2860, 1733, 1590, 1474, 1434, 1165.

Synthesis of **33**:

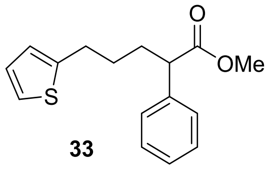

33

Prepared following **GP3**, with pyridinium **33a** (101 mg, 0.200 mmol), Hantzsch ester (152 mg, 0.600 mmol), methyl 2-phenylacrylate (42 mg, 0.26 mmol), Et_3N (84 µL, 0.60 mmol) and DMA (0.8 mL). Purification by flash column chromatography (1–10% Et_2O/pentane) gave **33** (22 mg, 40% yield) as a colourless oil.

^{1}H NMR (400 MHz, $CDCl_3$) δ 7.35 – 7.23 (m, 5H), 7.10 (dd, *J* = 5.1, 1.2 Hz, 1H), 6.90 (dd, *J* = 5.1, 3.4 Hz, 1H), 6.77 – 6.73 (m, 1H), 3.66 (s, 3H), 3.57 (t, *J* = 7.7 Hz, 1H), 2.84 (td, *J* = 7.2, 2.8 Hz, 2H), 2.15 (dddd, *J* = 13.3, 10.3, 7.9, 5.5 Hz, 1H), 1.86 (dddd, *J* = 13.3, 10.0, 7.5, 5.5 Hz, 1H), 1.74 – 1.57 (m, 2H).

^{13}C NMR (100 MHz, $CDCl_3$) δ 174.5, 144.9, 139.1, 128.8, 128.0, 127.4, 126.8, 124.3, 123.1, 52.1, 51.5, 33.0, 29.8, 29.7.

Spectroscopic data were consistent with those previously reported.[15]

Synthesis of **34**:

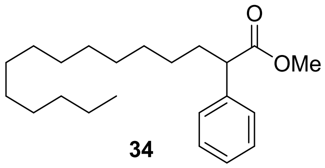

34

Prepared following **GP3**, with pyridinium **34a** (113 mg, 0.200 mmol), Hantzsch ester (152 mg, 0.600 mmol), methyl 2-phenylacrylate (42 mg, 0.26 mmol), Et_3N (84 µL, 0.60 mmol) and DMA (0.8 mL). Purification by flash column chromatography (100% toluene) gave a mixture of **34** and 2,4,6-triphenylpridine. The mixture was then purified by preparative TLC (petroleum ether/acetone = 30:1) to give pure **34** (31 mg, 49% yield) as a colourless oil.

^{1}H NMR (400 MHz, $CDCl_3$) δ 7.35 – 7.29 (m, 4H), 7.27 – 7.23 (m, 1H), 3.65 (s, 3H), 3.53 (t, *J* = 7.7 Hz, 1H), 2.11 – 2.02 (m, 1H), 1.80 – 1.72 (m, 1H), 1.32 – 1.24 (m, 22H), 0.88 (t, *J* = 6.8 Hz, 3H).

^{13}C NMR (100 MHz, $CDCl_3$) δ 174.8, 139.5, 128.7, 128.1, 127.3, 52.0, 51.8, 33.7, 32.1, 29.8, 29.8, 29.8, 29.7, 29.6, 29.5, 27.7, 22.8, 14.3 (*2 aliphatic carbon signals are not observed due to overlap*).

HRMS (ESI^+): calculated for $C_{22}H_{37}O_2$ $[M+H]^+$ 333.2788, found 333.2801.

IR (ν_{max}/cm^{-1}, neat) 3091, 3065, 3029, 2922, 2853, 1738, 1455, 1274, 1261, 1160.

Synthesis of **35**:

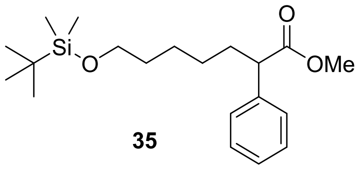

35

Prepared following **GP3**, with pyridinium **35a** (116 mg, 0.200 mmol), Hantzsch ester (152 mg, 0.600 mmol), methyl 2-phenylacrylate (42 mg, 0.26 mmol), Et_3N (84 µL, 0.60 mmol) and DMA (0.8 mL). Purification by flash column chromatography (1–8% Et_2O/pentane) gave **35** (36 mg, 51% yield) as a colourless oil.

^{1}H NMR (400 MHz, $CDCl_3$) δ 7.34 – 7.28 (m, 4H), 7.27 – 7.22 (m, 1H), 3.65 (s, 3H), 3.57 (t, *J* = 6.3 Hz, 2H), 3.54 (t, *J* = 7.7 Hz, 1H), 2.13 – 2.02 (m, 1H), 1.83 – 1.72 (m, 1H), 1.48 (p, *J* = 6.7 Hz, 2H), 1.39 – 1.20 (m, 4H), 0.88 (s, 9H), 0.03 (s, 6H).

^{13}C NMR (125 MHz, $CDCl_3$) δ 174.7, 139.4, 128.7, 128.0, 127.3, 63.2, 52.1, 51.7, 33.7, 32.7, 27.5, 26.1, 25.7, 18.5, –5.1.

HRMS (ESI$^+$): calculated for $C_{20}H_{35}SiO_3$ [M+H]$^+$ 351.2350, found 351.2348.

IR (ν_{max}/cm^{-1}, neat) 3086, 3065, 3029, 3004, 2946, 2929, 2856, 1738, 1275, 1256, 1156, 1099, 835.

6.2. Allylation reactions

Synthesis of **50**:

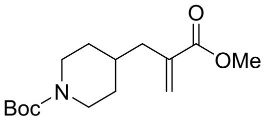

50

Prepared following **GP4**, with pyridinium **1a** (116 mg, 0.200 mmol), Hantzsch ester (76 mg, 0.30 mmol), allyl sulfone **49** (144 mg, 0.600 mmol) and DMA (0.5 mL). Purification by flash column chromatography (1–25% Et_2O/pentane) gave **50** (43 mg, 76% yield) as a yellow solid.

^{1}H NMR (400 MHz, $CDCl_3$) δ 6.18 (d, *J* = 1.6 Hz, 1H), 5.51 (q, *J* = 1.2 Hz, 1H), 4.06 (br. s, 2H), 3.75 (s, 3H), 2.65 (t, *J* = 12.3 Hz, 2H), 2.23 (d, *J* = 6.4 Hz, 2H), 1.68 – 1.55 (m, 4H), 1.44 (s, 9H), 1.06 (qd, *J* = 13.9, 13.4, 4.2 Hz, 2H).

^{13}C NMR (100 MHz, $CDCl_3$) δ 167.6, 154.8, 138.3, 126.5, 79.2, 51.9, 43.8, 39.2, 34.9, 31.9, 28.5.

HRMS (ESI^+): calculated for $C_{15}H_{25}NO_4Na$ $[M+Na]^+$ 306.1676, found 306.1691.

IR (ν_{max}/cm^{-1}, neat) 3007, 2975, 2928, 2852, 1720, 1688, 1420, 1276, 1163, 1113.

Synthesis of **51**:

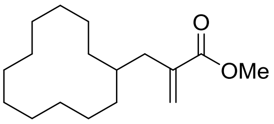

51

Prepared following **GP4**, with pyridinium **18a** (112 mg, 0.200 mmol), Hantzsch ester (76 mg, 0.30 mmol), allyl sulfone **49** (144 mg, 0.600 mmol) and DMA (0.8 mL). Purification by preparative TLC (5% Et_2O/pentane) gave **51** (41 mg, 77% yield) as a colourless oil.

^{1}H NMR (400 MHz, $CDCl_3$) δ 6.15 (d, *J* = 1.6 Hz, 1H), 5.49 (q, *J* = 1.3 Hz, 1H), 3.75 (s, 3H), 2.21 (dd, *J* = 7.0, 1.2 Hz, 2H), 1.71 – 1.62 (m, 1H), 1.40 – 1.27 (m, 20H), 1.25 – 1.17 (m, 2H).

^{13}C NMR (125 MHz, $CDCl_3$) δ 168.1, 140.0, 125.8, 51.9, 37.9, 32.9, 29.0, 24.8, 24.2, 23.6, 23.5, 21.8.

HRMS (ESI^+): calculated for $C_{17}H_{30}O_2Na$ $[M+Na]^+$ 289.2138, found 289.2140.

IR (ν_{max}/cm^{-1}, neat) 3007, 2989, 2929, 2860, 2849, 1722, 1471, 1439, 1276, 1261, 1199, 1158.

Synthesis of **52**:

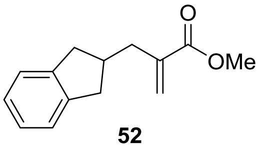

52

Prepared following **GP4**, with pyridinium **19a** (103 mg, 0.200 mmol), Hantzsch ester (76 mg, 0.30 mmol), allyl sulfone **49** (144 mg, 0.600 mmol) and DMA (0.8 mL). Purification by flash column chromatography (100% toluene) gave **52** (32 mg, 74% yield) as a colourless oil.

^{1}H NMR (400 MHz, $CDCl_3$) δ 7.21 – 7.17 (m, 2H), 7.16 – 7.11 (m, 2H), 6.22 (d, *J* = 1.5 Hz, 1H), 5.59 (q, *J* = 1.3 Hz, 1H), 3.78 (s, 3H), 3.05 (dd, *J* = 14.9, 7.4 Hz, 2H), 2.78 – 2.67 (m, 1H), 2.63 (dd, *J* = 15.4, 6.7 Hz, 2H), 2.49 (dd, *J* = 7.2, 0.9 Hz, 2H).

^{13}C NMR (100 MHz, $CDCl_3$) δ 167.9, 143.1, 139.7, 126.3, 125.9, 124.6, 52.0, 39.0, 38.3, 37.9.

Spectroscopic data were consistent with those previously reported.[16]

Synthesis of **53**:

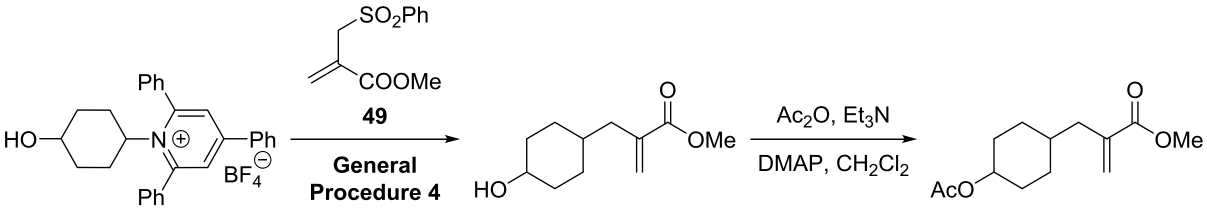

53a **53** **53b**

Prepared following **GP4**, with pyridinium **56a** (116 mg, 0.200 mmol), Hantzsch ester (76 mg, 0.30 mmol), allyl sulfone **49** (144 mg, 0.600 mmol) and DMA (0.5 mL). To a solution of crude product **53** in CH_2Cl_2 (8 mL), DMAP (3 mg, 0.02 mmol) and Et_3N (0.56 mL, 4.0 mmol) were added. The reaction mixture was cooled to 0 °C, then Ac_2O (0.38 mL, 4.0 mmol) was added dropwise over 5 min. The reaction was stirred at rt for 4 h, then quenched with saturated aq. $NaHCO_3$ (30 mL), extracted with EtOAc (30 + 15 mL), washed with brine (20 mL), dried over $MgSO_4$, and concentrated *in vacuo*. Purification by flash column chromatography (1–20% Et_2O/pentane) gave **53b** (23 mg, 48% yield, 62:38 d.r.) as a colourless oil.

^{1}H NMR (400 MHz, $CDCl_3$) δ (*62:38 mixture of diastereomers*) 6.16 (dd, *J* = 3.0, 1.6 Hz, 1H), 5.54 – 5.46 (m, 1H), 4.96 (p, *J* = 3.5 Hz, 0.62H, major isomer), 4.63 (tt, *J* = 11.1, 4.4 Hz, 0.38H, minor isomer), 3.74 (s, 3H), 2.24 (dd, *J* = 6.6, 1.1 Hz, 1.24H, major isomer), 2.19 (dd, *J* = 7.0, 1.1 Hz, 0.76H, minor isomer), 2.04 (s, 1.86H, major isomer), 2.01 (s, 1.14H, minor isomer), 1.98 – 1.91 (m, 0.76H, minor isomer), 1.87 – 1.73 (m, 2H), 1.59 – 1.43 (m, 3H), 1.36 – 1.18 (m, 2.48H, major isomer), 1.01 (td, *J* = 12.6, 11.7, 3.1 Hz, 0.76H, minor isomer).

^{13}C NMR (100 MHz, $CDCl_3$) δ (*62:38 mixture of diastereomers*)170.76 (minor isomer), 170.73 (major isomer), 167.92 (major isomer), 167.84 (minor isomer), 139.0, 126.33 (minor isomer), 126.22 (major isomer), 73.5 (minor isomer), 70.0 (major isomer), 51.9, 39.27 (minor isomer), 39.15 (major isomer), 35.61 (minor isomer), 35.35 (major isomer), 31.5, 30.8, 29.5, 27.3, 21.6.

HRMS (ESI^+): calculated for $C_{13}H_{20}O_4Na$ $[M+Na]^+$ 263.1254, found 263.1265.

IR (ν_{max}/cm^{-1}, neat) 3007, 2989, 2935, 2856, 1722, 1275, 1258, 1239, 1199.

Synthesis of **54**:

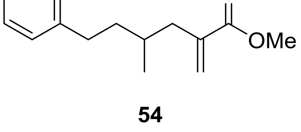

54

Prepared following **GP4**, with pyridinium **20a** (105 mg, 0.200 mmol), Hantzsch ester (76 mg, 0.30 mmol), allyl sulfone **49** (144 mg, 0.600 mmol) and DMA (0.8 mL). Purification by flash column

chromatography (100% toluene: 60 mL to remove 2,4,6-triphenylpyrdine; then 1–5% Et_2O/pentane gave **54** (30 mg, 65% yield) as a colourless oil.

^{1}H NMR (400 MHz, $CDCl_3$) δ 7.30 – 7.25 (m, 2H), 7.20 – 7.15 (m, 3H), 6.17 (d, *J* = 1.6 Hz, 1H), 5.49 (q, *J* = 1.3 Hz, 1H), 3.74 (s, 3H), 2.70 (ddd, *J* = 13.5, 10.3, 5.6 Hz, 1H), 2.59 (ddd, *J* = 13.5, 10.3, 5.7 Hz, 1H), 2.42 (ddd, *J* = 13.8, 5.8, 1.3 Hz, 1H), 2.12 (ddd, *J* = 13.8, 7.9, 1.1 Hz, 1H), 1.77 – 1.62 (m, 2H), 1.50 – 1.40 (m, 1H), 0.93 (d, *J* = 6.5 Hz, 3H).

^{13}C NMR (100 MHz, $CDCl_3$) δ 168.0, 142.9, 139.5, 128.5, 128.4, 126.2, 125.7, 51.9, 39.7, 38.7, 33.5, 31.7, 19.4.

HRMS (ESI$^+$): calculated for $C_{15}H_{20}O_2Na$ [*M*+Na]$^+$ 255.1356, found 255.1360.

IR (ν_{max}/cm^{-1}, neat) 3086, 3061, 3026, 2952, 2928, 2854, 1721, 1439, 1275, 1194, 1153, 749.

Synthesis of **55**:

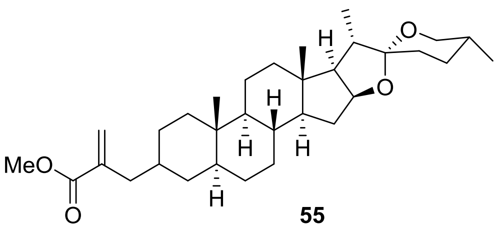

55

Prepared following **GP4**, with pyridinium **24a** (95 mg, 0.12 mmol), Hantzsch ester (46 mg, 0.18 mmol), allyl sulfone **49** (86 mg, 0.36 mmol) and DMA (0.4 mL). Purification by flash column chromatography (1–8% Et_2O/pentane) gave **55** (36 mg, 60% yield, 70:30 d.r.) as a white solid.

^{1}H NMR (400 MHz, $CDCl_3$) δ (*70:30 mixture of diastereomers*) 6.15 (d, *J* = 1.6 Hz, 0.7 H, major isomer), 6.13 (d, *J* = 1.7 Hz, 0.3 H, minor isomer), 5.47 (s, 1H), 4.43 – 4.33 (m, 1H), 3.73 (d, *J* = 1.2 Hz, 3H), 3.49 – 3.43 (m, 1H), 3.39 – 3.33 (m, 1H), 2.43 – 2.33 (m, 1.4H, major isomer), 2.23 – 2.13 (m, 0.6H, minor isomer), 1.97 (ddd, *J* = 11.6, 7.6, 5.3 Hz, 1H), 1.92 – 1.80 (m, 2H), 1.76 – 1.45 (m, 12H), 1.43 – 1.33 (m, 2H), 1.29 – 1.20 (m, 4H), 1.16 – 1.04 (m, 5H), 0.95 (d, *J* = 7.0 Hz, 3H), 0.92 – 0.87 (m, 1H), 0.79 (s, 3H), 0.76 (d, *J* = 9.7 Hz 3H), 0.75 (s, 3H), 0.72 – 0.59 (m, 1H).

^{13}C NMR (125 MHz, $CDCl_3$) δ (*70:30 mixture of diastereomers*) 168.1, 140.2 (major isomer), 139.3 (minor isomer), 125.9 (minor isomer), 125.7 (major isomer), 109.4, 81.0, 67.0, 62.3, 56.6 (major isomer), 56.5 (minor isomer), 54.9 (major isomer), 54.8 (minor isomer), 51.9, 46.6, 41.7, 40.7, 40.4, 40.3 (major isomer), 40.1 (minor isomer), 38.6, 37.1, 36.6, 36.3, 35.4, 35.3, 34.6, 33.2, 32.5, 32.5, 31.9, 31.5, 30.4, 28.9 (major isomer), 28.8 (minor isomer), 24.9, 21.0 (minor isomer), 20.7 (major isomer), 17.3, 16.7, 14.6, 12.5 (minor isomer), 12.0 (major isomer). (*all other peaks are unresolved from the corresponding signals of the other diastereomer*).

HRMS (MALDI$^+$): calculated for $C_{32}H_{50}O_4Na$ [*M*+Na]$^+$ 521.3601, found 521.3608.

IR (ν_{max}/cm^{-1}, neat) 3007, 2950, 2922, 2852, 1722, 1446, 1275, 1260, 1171, 1052, 981.

Synthesis of **56**:

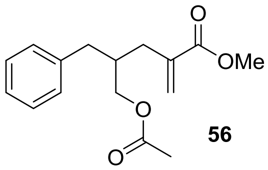

56

Prepared following **GP4**, with pyridinium **56a** (114 mg, 0.200 mmol), Hantzsch ester (76 mg, 0.30 mmol), allyl sulfone **49** (144 mg, 0.600 mmol) and DMA (0.8 mL). Purification by flash column chromatography (20% acetone/petroleum ether) gave **56** (18 mg, 37%) as a colourless oil.

^{1}H NMR (400 MHz, $CDCl_3$) δ 7.30–7.26(m, 2H), 7.24–7.11 (m, 3H), 6.21 (s, 1H), 5.56 (s, 1H), 3.97–3.89 (m, 2H), 3.73 (s, 3H), 2.70 – 2.60 (m, 2H), 2.43 – 2.32 (m, 2H), 2.30 – 2.25 (m, 1H), 2.04 (s, 3H).

^{13}C NMR (100 MHz, $CDCl_3$) δ 171.2, 167.6, 139.7, 138.6, 129.2, 128.5, 127.0, 126.3, 65.8, 52.0, 38.4, 37.9, 34.6, 21.0.

HRMS (ESI^+): calculated for $C_{16}H_{20}O_4Na$ [M+Na]$^+$ 299.1254, found 299.1250.

IR (ν_{max}/cm^{-1}, neat) 3006, 2990, 2961, 2972, 2847, 1739, 1727, 1275, 1238, 1147, 762, 749.

Synthesis of **57**:

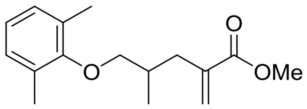

57

Prepared following **GP4**, with pyridinium **23a** (112 mg, 0.200 mmol), Hantzsch ester (76 mg, 0.30 mmol), allyl sulfone **49** (144 mg, 0.600 mmol) and DMA (0.8 mL). Purification by flash column chromatography (100% toluene: 60 mL to remove 2,4,6-triphenylpyrdine; then 1–5–10% Et_2O/pentane) gave **57** (35 mg, 67% yield) as a colourless oil.

^{1}H NMR (400 MHz, $CDCl_3$) δ 7.00 (d, J = 7.4 Hz, 2H), 6.91 (dd, J = 8.1, 6.8 Hz, 1H), 6.23 (d, J = 1.4 Hz, 1H), 5.60 (q, J = 1.3 Hz, 1H), 3.77 (s, 3H), 3.68 – 3.56 (m, 2H), 2.70 – 2.60 (m, 1H), 2.30 – 2.19 (m, 2H), 2.27 (s, 6H), 1.10 (d, J = 6.5 Hz, 3H).

^{13}C NMR (100 MHz, $CDCl_3$) δ 167.9, 155.9, 139.1, 131.0, 128.9, 126.5, 123.8, 76.6, 52.0, 36.3, 33.5, 16.9, 16.5.

HRMS (ESI^+): calculated for $C_{16}H_{23}O_3$ [M+H]$^+$ 263.1642, found 263.1644;

calculated for $C_{16}H_{22}O_3Na$ [M+Na]$^+$ 285.1461, found 285.1477.

IR (ν_{max}/cm^{-1}, neat) 3023, 2954, 2924, 2868, 1722, 1477, 1440, 1263, 1202, 1160, 1091, 1020.

Synthesis of **58**:

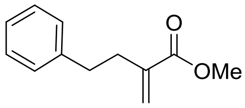

58

Prepared following **GP4**, with pyridinium **25a** (97 mg, 0.20 mmol), Hantzsch ester (76 mg, 0.30 mmol), allyl sulfone **49** (144 mg, 0.600 mmol) and DMA (0.8 mL). Purification by flash column chromatography (100% toluene) gave **58** (24 mg, 62% yield) as a colourless oil.

^{1}H NMR (400 MHz, $CDCl_3$) δ 7.31 – 7.26 (m, 2H), 7.21 – 7.15 (m, 3H), 6.15 (dd, *J* = 1.3, 0.7 Hz, 1H), 5.51 (q, *J* = 1.3 Hz, 1H), 3.77 (s, 3H), 2.79 (dd, *J* = 9.4, 6.3 Hz, 2H), 2.65 – 2.59 (m, 2H).

^{13}C NMR (100 MHz, $CDCl_3$) δ 167.6, 141.4, 139.8, 128.5, 128.3, 126.0, 125.4, 51.8, 34.9, 33.9.

Spectroscopic data were consistent with those previously reported.[17]

Synthesis of **59**:

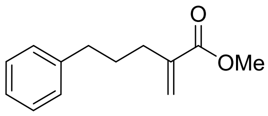

59

Prepared following **GP5**, with pyridinium **28a** (100 mg, 0.200 mmol), Hantzsch ester (126 mg, 0.500 mmol), allyl sulfone **49** (144 mg, 0.600 mmol), Et_3N (84 µL, 0.60 mmol) and DMA (0.8 mL). Purification by flash column chromatography (100% toluene: 60 mL to remove 2,4,6-triphenylpyrdine; then 5% Et_2O/pentane) gave **59** (23 mg, 56% yield) as a colourless oil.

^{1}H NMR (400 MHz, $CDCl_3$) δ 7.31 – 7.26 (m, 2H), 7.21 – 7.15 (m, 3H), 6.16 (dt, *J* = 1.5, 0.7 Hz, 1H), 5.54 (q, *J* = 1.4 Hz, 1H), 3.75 (s, 3H), 2.68 – 2.62 (m, 2H), 2.39 – 2.33 (m, 2H), 1.86 – 1.77 (m, 2H).

^{13}C NMR (100 MHz, $CDCl_3$) δ 167.9, 142.2, 140.5, 128.6, 128.5, 125.9, 125.0, 51.9, 35.5, 31.7, 30.2.

Spectroscopic data were consistent with those previously reported.[17]

Synthesis of **60**:

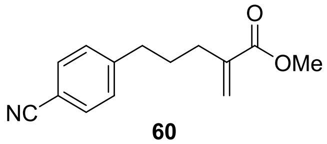

60

Prepared following **GP5**, with pyridinium **60a** (105 mg, 0.200 mmol), Hantzsch ester (126 mg, 0.500 mmol), allyl sulfone **49** (144 mg, 0.600 mmol), Et_3N (84 µL, 0.60 mmol) and DMA (0.8 mL). Purification by flash column chromatography (1–15% Et_2O/pentane) gave **60** (21 mg, 46% yield) as a white solid.

^{1}H NMR (500 MHz, $CDCl_3$) δ 7.59 – 7.54 (m, 2H), 7.30 – 7.27 (m, 2H), 6.17 (dd, *J* = 1.4, 0.6 Hz, 1H), 5.54 (q, *J* = 1.3 Hz, 1H), 3.75 (s, 3H), 2.72 – 2.67 (m, 2H), 2.37 – 2.31 (m, 2H), 1.86 – 1.78 (m, 2H).

^{13}C NMR (125 MHz, $CDCl_3$) δ 167.6, 147.8, 140.1, 132.3, 129.3, 125.4, 119.2, 109.9, 52.0, 35.6, 31.6, 29.7.

HRMS (ESI$^+$): calculated for $C_{14}H_{15}NO_2Na$ [M+Na]$^+$ 252.0995, found 252.1007.

IR (ν_{max}/cm^{-1}, neat) 3004, 2989, 2953, 2928, 2860, 2227, 1718, 1438, 1196, 1170, 1135.

Synthesis of **61**:

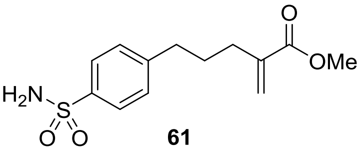

61

Prepared following **GP5**, with pyridinium **31a** (113 mg, 0.200 mmol), Hantzsch ester (127 mg, 0.500 mmol), allyl sulfone **49** (144 mg, 0.600 mmol), Et_3N (84 µL, 0.60 mmol) and DMA (0.8 mL). Purification by flash column chromatography (20% acetone/pentane) gave **61** (28 mg, 49%) as a yellow oil.

^{1}H NMR (400 MHz, $CDCl_3$) δ 7.83 (d, J = 8.1 Hz, 2H), 7.32 (d, J = 8.1 Hz, 2H), 6.16 (s, 1H), 5.55 (s, 1H), 5.01 (br. s, 2H), 3.74 (s, 3H), 2.71 (t, J = 7.7 Hz, 2H), 2.34 (t, J = 7.7 Hz, 2H), 1.82 (p, J = 7.7 Hz, 2H).

^{13}C NMR (100 MHz, $CDCl_3$) δ 167.7, 147.7, 140.1, 139.7, 129.2, 126.7, 125.4, 52.0, 35.4, 31.6, 29.8.

HRMS (ESI$^+$): calculated for $C_{13}H_{17}NO_4SNa$ [M+Na]$^+$ 306.0771, found 306.0760.

IR (ν_{max}/cm^{-1}, neat) 3356 (br.), 3261 (br.), 2947, 2923, 2856, 1707, 1629, 1596, 1401, 1333, 1160, 948, 749.

Synthesis of **62**:

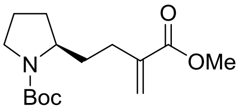

62

Prepared following **GP5**, with pyridinium **62a** (116 mg, 0.200 mmol), Hantzsch ester (126 mg, 0.500 mmol), allyl sulfone **49** (144 mg, 0.600 mmol), Et_3N (84 µL, 0.60 mmol) and DMA (0.8 mL). Purification by flash column chromatography (1–10% EtOAc/pentane) gave **62** (27 mg, 48% yield) as a colourless oil.

^{1}H NMR (500 MHz, DMSO-d_6, 100 °C) δ 6.05 (dd, J = 1.4, 0.7 Hz, 1H), 5.64 (q, J = 1.4 Hz, 1H), 3.73 – 3.68 (m, 1H), 3.70 (s, 3H), 3.32 (dt, J = 10.6, 7.4 Hz, 1H), 3.20 (ddd, J = 10.6, 7.7, 5.1 Hz, 1H), 2.32 – 2.20 (m, 2H), 1.93 (dq, J = 12.1, 7.9 Hz, 1H), 1.86 – 1.78 (m, 2H), 1.78 – 1.71 (m, 1H), 1.65 (ddt, J = 11.8, 7.2, 4.0 Hz, 1H), 1.48 (ddt, J = 12.7, 8.6, 4.5 Hz, 1H), 1.41 (s, 9H).

^{13}C NMR (125 MHz, DMSO-d_6, 100 °C) δ 166.4, 153.2, 139.8, 123.8, 77.6, 56.0, 51.0, 45.5, 32.4, 29.4, 27.8, 27.6, 22.4.

HRMS (ESI$^+$): calculated for $C_{15}H_{26}NO_4$ [M+H]$^+$ 284.1856, found 284.1857.

IR (ν_{max}/cm^{-1}, neat) 3007, 2976, 2928, 2874, 1722, 1693, 1393, 1275, 1260, 1167.

Synthesis of **63**:

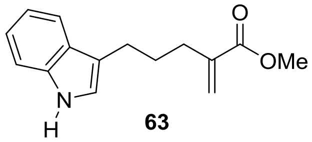

63

Prepared following **GP5**, with pyridinium **63a** (108 mg, 0.200 mmol), Hantzsch ester (127 mg, 0.500 mmol), allyl sulfone **49** (144 mg, 0.600 mmol), Et_3N (84 µL, 0.60 mmol) and DMA (0.8 mL). Purification by flash column chromatography (10% acetone/pentane) gave **63** (25 mg, 52%) as a yellow oil.

^{1}H NMR (400 MHz, $CDCl_3$) δ 7.94 (br. s, 1H), 7.61 (d, *J* = 8.3 Hz, 1H), 7.35 (d, *J* = 8.1, 0.9 Hz, 1H), 7.22 – 7.17 (m, 1H), 7.12 (ddd, *J* = 7.9, 7.0, 1.1 Hz, 1H), 7.02 – 6.97 (m, 1H), 6.17 (s, 1H), 5.56 (d, *J* = 1.4 Hz, 1H), 3.76 (s, 3H), 2.81 (t, *J* = 7.6 Hz, 2H), 2.43 (t, *J* = 7.7 Hz, 2H), 1.92 (p, *J* = 7.6 Hz, 2H).

^{13}C NMR (100 MHz, $CDCl_3$) δ 168.0, 140.7, 136.5, 127.7, 124.9, 122.0, 121.3, 119.2, 119.0, 116.5, 111.2, 51.9, 31.9, 28.8, 24.8.

HRMS (ESI^+): calculated for $C_{15}H_{17}NO_2Na$ $[M+Na]^+$ 266.1151, found 266.1156.

IR (ν_{max}/cm^{-1}, neat) 3413, 3007, 2927, 2852, 1716, 1626, 1457, 1275, 1199, 764, 748.

Synthesis of **64**:

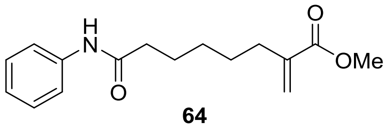

64

Prepared following **GP5**, with pyridinium **64a** (114 mg, 0.200 mmol), Hantzsch ester (126 mg, 0.500 mmol), allyl sulfone **49** (144 mg, 0.600 mmol), Et_3N (84 µL, 0.60 mmol) and DMA (0.8 mL). Purification by flash column chromatography (1–15% acetone/pentane) gave **64** (32 mg, 58% yield) as a colourless oil.

^{1}H NMR (500 MHz, $CDCl_3$) δ 7.52 (d, *J* = 8.0 Hz, 2H), 7.51 (br. s, 1H), 7.29 (t, *J* = 7.9 Hz, 2H), 7.08 (t, *J* = 7.4 Hz, 1H), 6.12 (dd, *J* = 1.5, 0.7 Hz, 1H), 5.52 (q, *J* = 1.4 Hz, 1H), 3.74 (s, 3H), 2.34 (t, *J* = 7.5 Hz, 2H), 2.30 (t, *J* = 7.5 Hz, 2H), 1.74 (p, *J* = 7.5 Hz, 2H), 1.50 (p, *J* = 7.4 Hz, 2H), 1.42 – 1.36 (m, 2H).

^{13}C NMR (125 MHz, $CDCl_3$) δ 171.5, 167.9, 140.6, 138.2, 129.0, 125.0, 124.2, 119.9, 51.9, 37.6, 31.8, 28.8, 28.2, 25.4.

HRMS (ESI^+): calculated for $C_{16}H_{22}NO_3$ $[M+H]^+$ 276.1594, found 276.1594.

IR (ν_{max}/cm^{-1}, neat) 3298 (br.), 3198, 3140, 3007, 2989, 2946, 2932, 2860, 1718, 1660, 1599, 1538, 1499, 1440, 1275, 1260.

Synthesis of **65**:

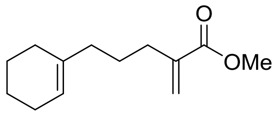

65

Prepared following **GP5**, with pyridinium **65a** (101 mg, 0.200 mmol), Hantzsch ester (126 mg, 0.500 mmol), allyl sulfone **49** (144 mg, 0.600 mmol), Et_3N (84 µL, 0.60 mmol) and DMA (0.8 mL). Purification by flash column chromatography (100% toluene) gave **65** (21 mg, 50% yield) as a colourless oil.

^{1}H NMR (500 MHz, $CDCl_3$) δ 6.13 (dt, *J* = 1.5, 0.7 Hz, 1H), 5.52 (q, *J* = 1.4 Hz, 1H), 5.42 – 5.38 (m, 1H), 3.75 (s, 3H), 2.30 – 2.24 (m, 2H), 2.00 – 1.93 (m, 4H), 1.92 – 1.88 (m, 2H), 1.64 – 1.57 (m, 3H), 1.56 – 1.52 (m, 3H).

^{13}C NMR (125 MHz, $CDCl_3$) δ 168.0, 140.9, 137.4, 124.7, 121.3, 51.9, 37.7, 31.7, 28.4, 26.5, 25.4, 23.2, 22.7.

HRMS (ESI$^+$): calculated for $C_{13}H_{20}O_2Na$ [M+Na]$^+$ 231.1356, found 231.1355.

IR (ν_{max}/cm^{-1}, neat) 3004, 2989, 2926, 2860, 2838, 1721, 1437, 1275, 1260, 1195, 1169.

Synthesis of **66**:

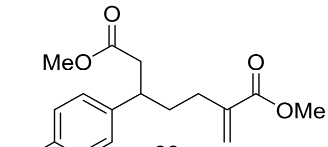

66

Prepared following **GP5**, with pyridinium **66a** (121 mg, 0.200 mmol), Hantzsch ester (126 mg, 0.500 mmol), allyl sulfone **49** (144 mg, 0.600 mmol), Et_3N (84 µL, 0.60 mmol) and DMA (0.8 mL). Purification by flash column chromatography (10% acetone/petroleum ether) gave **66** (25 mg, 40% yield) as a colourless oil.

^{1}H NMR (400 MHz, $CDCl_3$) δ 7.27 (d, *J* = 8.3 Hz, 2H), 7.13 (d, *J* = 8.3 Hz, 2H), 6.11 (s, 1H), 5.46 (s, 1H), 3.72 (s, 3H), 3.57 (s, 3H), 3.18 – 3.03 (m, 1H), 2.67–2.50 (m, 2H), 2.14 (t, *J* = 7.8 Hz, 2H), 1.93 – 1.68 (m, 2H).

^{13}C NMR (100 MHz, $CDCl_3$) δ 172.4, 167.5, 141.9, 140.0, 132.5, 129.0, 128.8, 125.3, 51.9, 51.7, 41.7, 41.3, 34.7, 30.0.

HRMS (ESI$^+$): calculated for $C_{16}H_{19}ClO_4Na$ [M+Na]$^+$ 333.0864, found 333.0873.

IR (ν_{max}/cm^{-1}, neat) 3029, 2993, 2951, 2982, 2852, 1735, 1721, 1630, 1437, 1275, 1259, 1137, 829, 764, 750.

Synthesis of **67**:

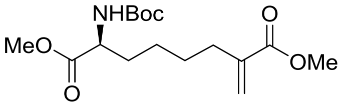

67

Prepared following **GP5**, with pyridinium **67a** (125 mg, 0.200 mmol), Hantzsch ester (126 mg, 0.500 mmol), allyl sulfone **49** (144 mg, 0.600 mmol), Et_3N (84 µL, 0.60 mmol) and DMA (0.8 mL). Purification by flash column chromatography (15% EtOAc/petroleum ether) gave **67** (36 mg, 54% yield) as a colourless oil.

^{1}H NMR (400 MHz, $CDCl_3$) δ 6.13 (s, 1H), 5.51 (s, 1H), 4.99 (d, J = 8.4 Hz, 1H), 4.32 – 4.24 (m, 1H), 3.74 (s, 3H), 3.73 (s, 3H), 2.29 (t, J = 7.5 Hz, 2H), 1.87 – 1.72 (m, 1H), 1.68 – 1.59 (m, 1H), 1.55 – 1.45 (m, 2H), 1.43 (s, 9H), 1.40 – 1.30 (m, 2H).

^{13}C NMR (100 MHz, $CDCl_3$) δ 173.5, 167.8, 155.5, 140.4, 125.0, 80.0, 53.5, 52.4, 51.9, 32.7, 31.8, 28.5, 28.1, 25.0.

HRMS (ESI$^+$): calculated for $C_{16}H_{27}NO_6Na$ [M+Na]$^+$ 352.1731, found 352.1743.

IR (ν_{max}/cm^{-1}, neat) 3370, 2987, 2946, 1748, 1718, 1513, 1439, 1275, 1260, 1167, 764, 750.

Synthesis of **68**:

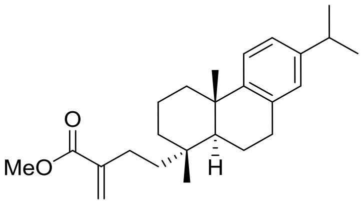

68

Prepared following **GP5**, with pyridinium **68a** (133 mg, 0.200 mmol), Hantzsch ester (126 mg, 0.500 mmol), allyl sulfone **49** (144 mg, 0.600 mmol), Et_3N (84 µL, 0.60 mmol) and DMA (0.8 mL). Purification by flash column chromatography (100% toluene) gave **68** (22 mg, 30% yield) as a colourless oil.

^{1}H NMR (400 MHz, $CDCl_3$) δ 7.18 (d, J = 8.1 Hz, 1H), 6.99 (dd, J = 8.2, 2.0 Hz, 1H), 6.89 (d, J = 1.9 Hz, 1H), 6.11 (d, J = 1.4 Hz, 1H), 5.52 (d, J = 1.4 Hz, 1H), 3.75 (s, 3H), 2.98 – 2.75 (m, 3H), 2.33 – 2.14 (m, 3H), 1.87 – 1.60 (m, 4H), 1.52 – 1.31 (m, 6H), 1.23 (d, J = 6.6 Hz, 6H), 1.22 (s, 3H), 0.95 (s, 3H).

^{13}C NMR (125 MHz, $CDCl_3$) δ 168.0, 147.8, 145.6, 141.7, 135.0, 127.0, 124.5, 124.0, 51.9, 47.4, 43.3, 38.8, 37.7, 37.1, 36.0, 33.6, 30.5, 26.2, 25.5, 24.1, 24.1, 21.1, 19.2, 18.8 (*1 aromatic carbon signal is not observed due to signal overlap*).

HRMS (ESI$^+$): calculated for $C_{25}H_{36}O_2Na$ [M+Na]$^+$ 391.2608, found 391.2605.

IR (ν_{max}/cm^{-1}, neat) 3004, 2989, 2956, 2925, 2867, 1722, 1275, 1260.

Synthesis of **69**:

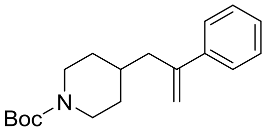

69

Prepared following **GP4**, with pyridinium **1a** (116 mg, 0.200 mmol), Hantzsch ester (76 mg, 0.30 mmol), allyl sulfone **69a** (155 mg, 0.600 mmol), and DMA (0.8 mL). Purification by flash column chromatography (1–10% EtOAc/pentane) gave **69** (25 mg, 41%) as a colourless oil.

^{1}H NMR (400 MHz, $CDCl_3$) δ 7.27 (d, *J* = 7.1 Hz, 2H), 7.21 (t, *J* = 7.1 Hz, 2H), 7.18 – 7.13 (m, 1H), 5.18 (d, *J* = 1.6 Hz, 1H), 4.92 (s, 1H), 3.91 (br. s, 2H), 2.46 (t, *J* = 12.9 Hz, 2H), 2.33 (d, *J* = 7.1 Hz, 2H), 1.52 (d, *J* = 13.5 Hz, 2H), 1.39 – 1.35(m, 1H), 1.33 (s, 9H), 0.98 (qd, *J* = 12.2, 4.4 Hz, 2H).

^{13}C NMR (100 MHz, $CDCl_3$) δ 155.0, 146.5, 141.2, 128.5, 127.6, 126.3, 114.2, 79.3, 44.0, 42.9, 34.4, 32.2, 28.6.

HRMS (ESI$^+$): calculated for $C_{19}H_{27}NO_2Na$ [*M*+Na]$^+$ 324.1934, found 324.1940.

IR (ν_{max}/cm^{-1}, neat) 3086, 3050, 3007, 2976, 1925, 2845, 1692, 1422, 1364, 1276, 1260, 1688, 971, 896, 764, 750.

Synthesis of **70**:

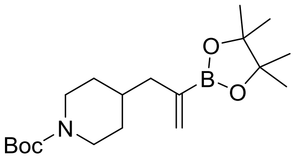

70

Prepared following **GP4**, with pyridinium **1a** (116 mg, 0.200 mmol), Hantzsch ester (76 mg, 0.30 mmol), allyl sulfone **70a** (185 mg, 0.600 mmol), and DMA (0.8 mL). Purification by flash column chromatography (2% acetone/petroleum ether) gave **70** (28 mg, 30%) as a white solid.

^{1}H NMR (400 MHz, $CDCl_3$) δ 5.82 (d, *J* = 3.6 Hz, 1H), 5.57 (d, *J* = 3.5 Hz, 1H), 4.04 (br. s, 2H), 2.65 (t, *J* = 12.6 Hz, 2H), 2.08 (d, *J* = 6.8 Hz, 2H), 1.65–1.59 (m, 2H), 1.56–1.50 (m, 1H), 1.44 (s, 9H), 1.25 (s, 12H), 1.04 (qd, *J* = 12.4, 4.3 Hz, 2H).

^{13}C NMR (100 MHz, $CDCl_3$) δ 155.1, 131.0, 83.5, 79.2, 44.2, 42.4, 36.1, 32.1, 28.6, 24.9 (t*he carbon attached to boron was not observed due to quadrupolar relaxation*).

HRMS (ESI$^+$): calculated for $C_{19}H_{34}BNO_4Na$ [*M*+Na]$^+$ 374.2477, found 374.2472.

IR (ν_{max}/cm^{-1}, neat) 2977, 2923, 2859, 1710, 1420, 1276, 1143, 967, 862, 750.

6.3. By-products

2,4,6-Triphenylpyridine (43)

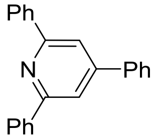

43

^{1}H NMR (500 MHz, $CDCl_3$) δ 8.23 – 8.19 (m, 4H), 7.90 (s, 2H), 7.78 – 7.74 (m, 2H), 7.57 – 7.43 (m, 9H).

^{13}C NMR (125 MHz, $CDCl_3$) δ 157.6, 150.3, 139.7, 139.2, 129.3, 129.2, 129.1, 128.8, 127.3, 127.3, 117.3.

Spectroscopic data were consistent with those previously reported.[22]

Diethyl 2,6-dimethylpyridine-3,5-dicarboxylate (47)

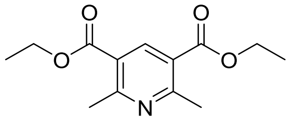

47 (free base)

^{1}H NMR (400 MHz, $CDCl_3$) δ 8.67 (s, 1H), 4.39 (q, *J* = 7.1 Hz, 4H), 2.84 (s, 6H), 1.41 (t, *J* = 7.1 Hz, 6H).

Spectroscopic data were consistent with those previously reported.[23]

7. Deaminative Reactions Promoted by Et_3N (or $i Pr_2NEt$)

Synthesis of **6** (without Hantzsch ester)**:**

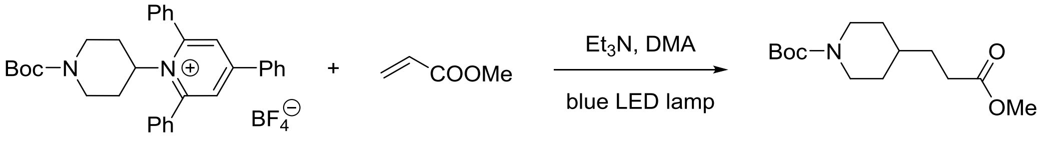

1a **6**

Prepared following **GP6**, with pyridinium **1a** (58 mg, 0.10 mmol), Et_3N (84 µL, 0.60 mmol), methyl acrylate (17 µL, 0.18 mmol) and DMA (0.4 mL). Purification by flash column chromatography (4–40% EtOAc/hexane) gave **6** (7 mg, 26% yield) as a colourless oil.

Synthesis of **50** (without Hantzsch ester)**:**

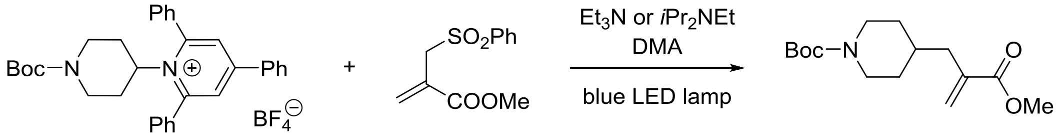

1a **49** **50**

71% with Et_3N
71% with $i Pr_2NEt$

Prepared following **GP6**, with pyridinium **1a** (58 mg, 0.10 mmol), allyl sulfone **49** (72 mg, 0.30 mmol), Et_3N (84 µL, 0.60 mmol) and DMA (0.4 mL). Purification by flash column chromatography (8% EtOAc/pentane) gave **50** (20 mg, 71%) as a colourless oil.

Using $i Pr_2NEt$ (104 µL, 0.60 mmol) instead of Et_3N gave **50** in 71% yield (20 mg).

Synthesis of **71**:

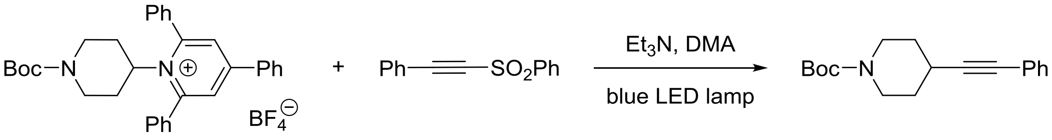

1a **71a** **71**

Prepared following **GP6**, with pyridinium **1a** (116 mg, 0.200 mmol), alkynyl sulfone **71a** (145 mg, 0.600 mmol), Et_3N (0.17 mL, 1.2 mmol) and DMA (0.8 mL). Purification by flash column chromatography (10% Et_2O/pentane) gave **71** (29 mg, 51%) as a colourless oil.

^{1}H NMR (400 MHz, $CDCl_3$) δ 7.42 – 7.37 (m, 2H), 7.30 – 7.26 (m, 3H), 3.74 (ddd, J = 13.2, 6.6, 3.6 Hz, 2H), 3.25 (ddd, J = 13.2, 8.4, 3.4 Hz, 2H), 2.80 (tt, J = 8.0, 4.0 Hz, 1H), 1.89 – 1.82 (m, 2H), 1.71 – 1.65 (m, 2H), 1.47 (s, 9H).

^{13}C NMR (100 MHz, $CDCl_3$) δ 154.9, 131.7, 128.4, 127.9, 123.7, 91.9, 82.1, 79.6, 42.4, 31.6, 28.6, 27.7.

Spectroscopic data were consistent with those previously reported.[18]

Synthesis of **72**:

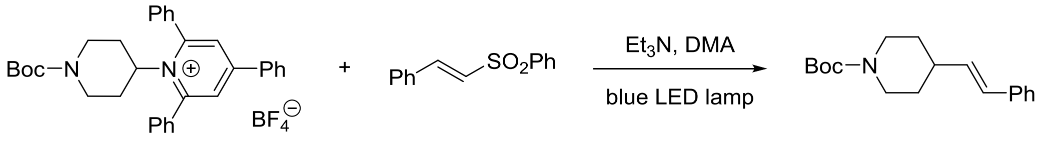

1a **72a** **72**
70% with Et_3N
70% with $i$$Pr_2NEt$

Prepared following **GP6**, with pyridinium **1a** (116 mg, 0.200 mmol), vinyl sulfone **72a** (147 mg, 0.600 mmol), Et_3N (0.17 mL, 1.2 mmol), and DMA (0.8 mL). Purification by flash column chromatography (20% EtOAc/petroleum ether) gave **72** (40 mg, 70%) as a colourless oil.

Using $i$$Pr_2NEt$ (0.21 mL, 1.2 mmol) instead of Et_3N gave **72** in 70% yield (40 mg).

^{1}H NMR (500 MHz, $CDCl_3$) δ 7.34 (dd, J = 8.3, 1.1 Hz, 2H), 7.32 – 7.28 (m, 2H), 7.23 – 7.18 (m, 1H), 6.39 (d, J = 16.0 Hz, 1H), 6.15 (dd, J = 16.0, 6.9 Hz, 1H), 4.13 (br. s, 2H), 2.78 (t, J = 10.0 Hz, 2H), 2.32 – 2.25 (m, 1H), 1.76 (d, J = 12.6 Hz, 2H), 1.47 (s, 9H), 1.38 (qd, J = 12.7, 4.4 Hz, 2H).

^{13}C NMR (125 MHz, $CDCl_3$) δ 155.0, 137.6, 134.5, 128.7, 128.6, 127.2, 126.2, 79.5, 44.1 (br.), 43.5 (br.), 39.5, 32.0, 28.6.

Spectroscopic data were consistent with those previously reported.[19]

Synthesis of **73**:

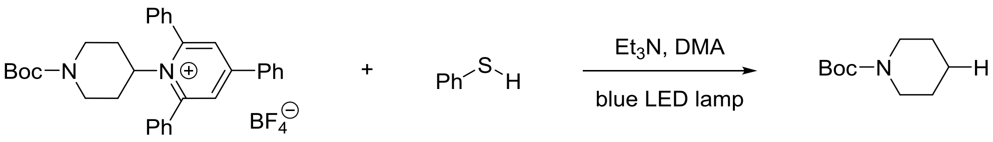

1a **73**

Prepared following **GP6**, with pyridinium **1a** (116 mg, 0.20 mmol), thiophenol (32 μL, 0.3 mmol), Et_3N (84 μL, 1.0 mmol), and DMA (0.5 mL), stirring for 14 h. Purification by flash column chromatography (1–20% Et_2O/pentane) gave **73** (26 mg, 70%) as a colourless oil.

^{1}H NMR (400 MHz, $CDCl_3$) δ 3.40 – 3.29 (m, 4H), 1.62 – 1.47 (m, 6H), 1.45 (s, 9H).

Spectroscopic data were consistent with those previously reported.[20]

Synthesis of **74**:

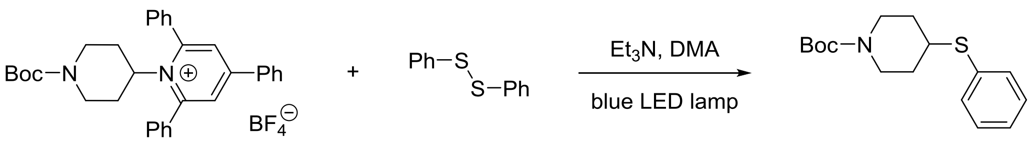

1a **74**

Prepared following **GP6**, with pyridinium **1a** (116 mg, 0.20 mmol), diphenyl disulfide (66 mg, 0.30 mmol), Et_3N (0.14 mL, 1.0 mmol), and DMA (0.5 mL), stirring for 14 h. Purification by flash column chromatography (1–10% EtOAc/pentane) gave **74** (42 mg, 72%) as a colourless oil.

^{1}H NMR (400 MHz, $CDCl_3$) δ 7.44 – 7.39 (m, 2H), 7.32 – 7.28 (m, 2H), 7.28 – 7.26 (m, 1H), 3.96 (d, *J* = 11.7 Hz, 2H), 3.21 (tt, *J* = 10.2, 3.9 Hz, 1H), 2.98 – 2.86 (m, 2H), 1.95 – 1.88 (m, 2H), 1.57 – 1.48 (m, 2H), 1.44 (s, 9H).

^{13}C NMR (100 MHz, $CDCl_3$) δ 154.8, 134.0, 132.9, 129.1, 127.5, 79.7, 44.7, 43.4 (br.), 32.3, 28.6.

Spectroscopic data were consistent with those previously reported.[21]

8. Mechanisitic Studies

8.1. UV/Vis absorption spectroscopy

Figure S2a show the UV/Vis absorption spectra of DMA solutions of secondary alkyl pyridinium **36**.

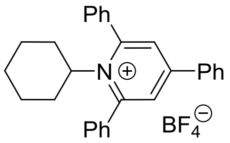

36

A) Pyridinium **36** (0.20 M)
B) Hantzsch ester (0.60 M)
C) Mixture of pyridinium **36** (0.20 M) and Hantzsch ester (0.60 M)
D) Mixture of pyridinium **36** (0.20 M) and Et_3N (0.60 M)
E) Et_3N (0.60 M)

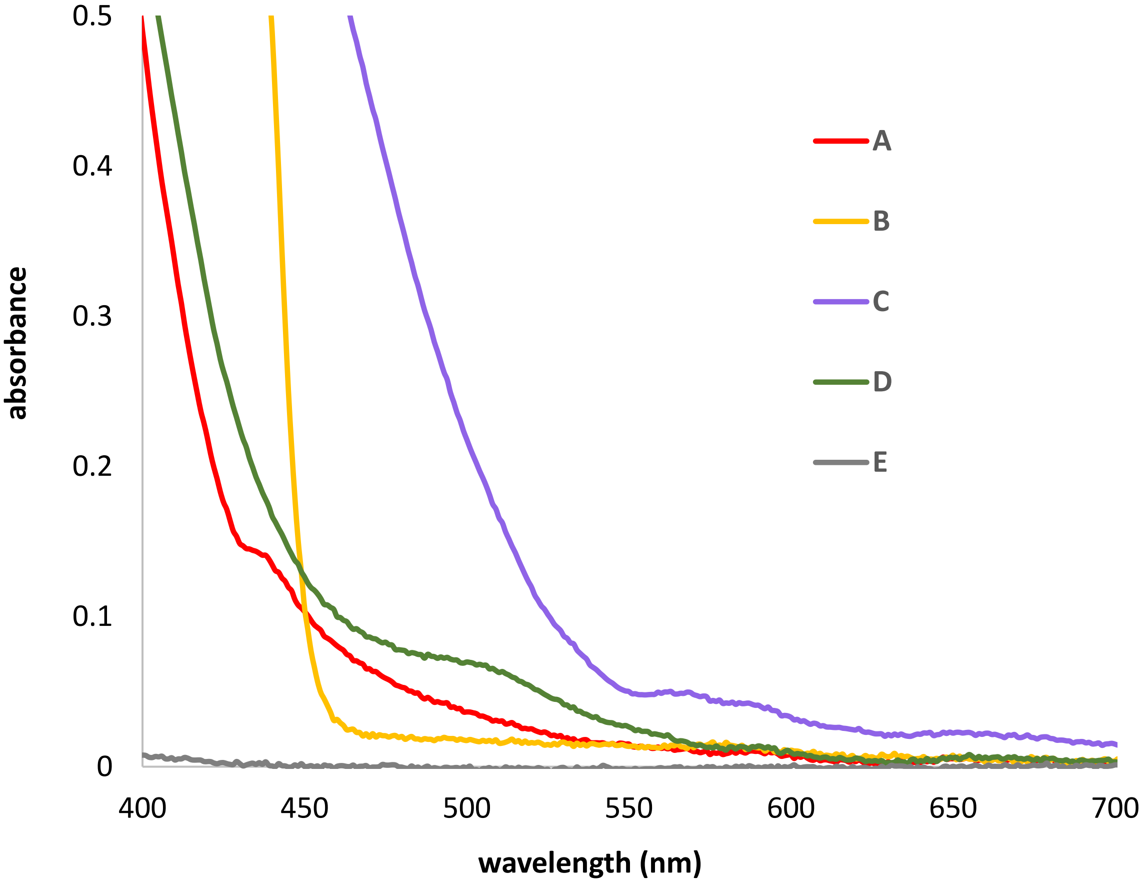

Figure S2a. UV/Vis absorption spectra of **36**

Figure S2b show the UV/Vis absorption spectra of DMA solutions of primary alkyl pyridinium **37**.

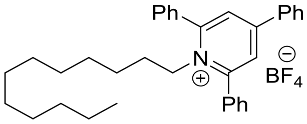

37

A) Pyridinium **37** (0.20 M)
B) Hantzsch ester (0.50 M)
C) Mixture of pyridinium **37** (0.20 M) and Hantzsch ester (0.50 M)
D) Mixture of pyridinium **37** (0.20 M) and Et_3N (0.60 M)
E) Mixture of pyridinium **37** (0.20 M), Hantzsch ester (0.50 M), and Et_3N (0.60 M)
F) Et_3N (0.60 M)

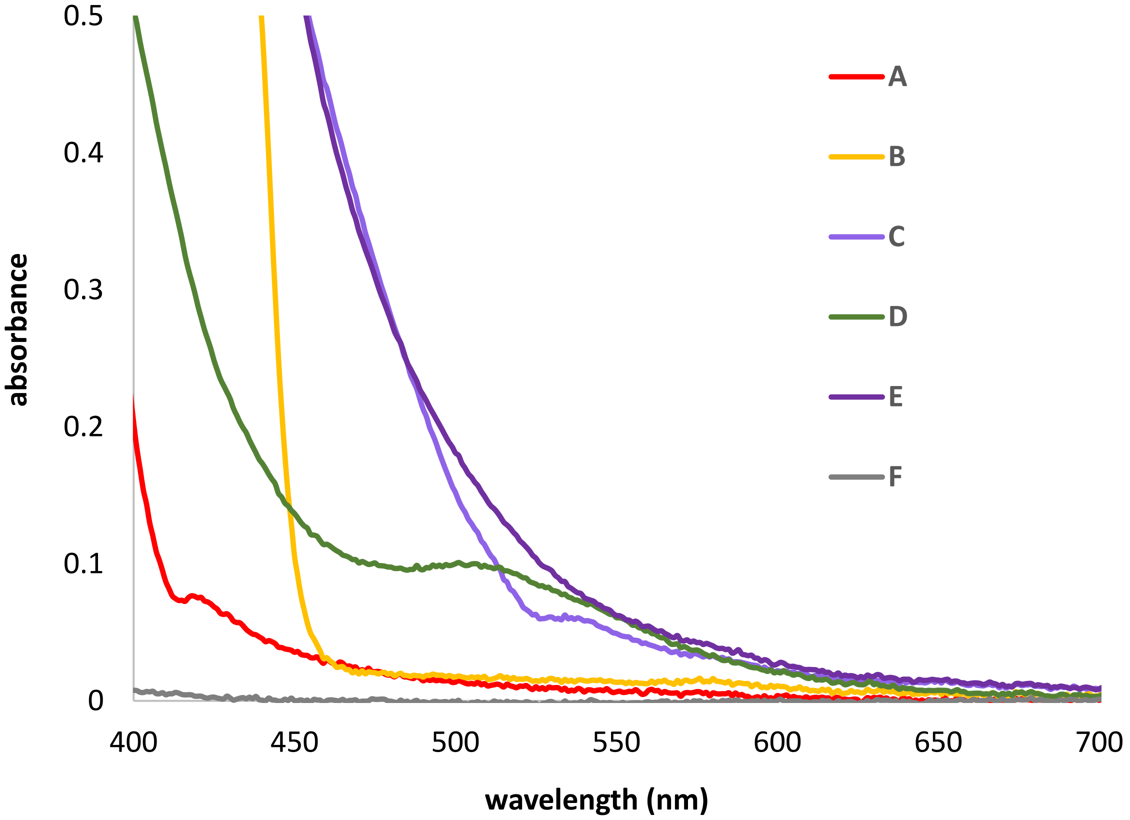

Figure S2b. UV/Vis absorption spectra of **37**

8.2. Radical clock experiment

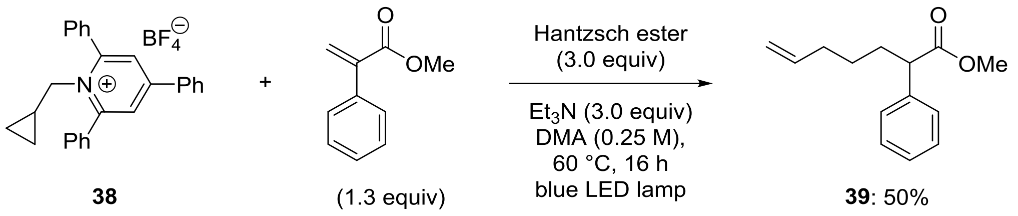

Prepared following **GP3**, with pyridinium **38** (90 mg, 0.20 mmol), Hantzsch ester (152 mg, 0.600 mmol), methyl 2-phenylacrylate (42 mg, 0.26 mmol), Et_3N (84 µL, 0.60 mmol) and DMA (0.8 mL). Purification by flash column chromatography (100% toluene) gave **39** (22 mg, 50% yield) as a colourless oil.

^{1}H NMR (500 MHz, $CDCl_3$) δ 7.34 – 7.28 (m, 4H), 7.27 – 7.24 (m, 1H), 5.76 (ddt, *J* = 16.9, 10.2, 6.7 Hz, 1H), 5.02 – 4.92 (m, 2H), 3.66 (s, 3H), 3.55 (t, *J* = 7.7 Hz, 1H), 2.12 – 2.03 (m, 3H), 1.79 (dddd, *J* = 13.4, 10.3, 7.4, 5.6 Hz, 1H), 1.43 – 1.26 (m, 2H).

^{13}C NMR (125 MHz, $CDCl_3$) δ 174.6, 139.3, 138.4, 128.7, 128.0, 127.3, 114.9, 52.1, 51.7, 33.6, 33.1, 27.0.

Spectroscopic data were consistent with those previously reported.[24]

8.3. Quantum yield

The quantum yield was measured for the reaction of pyridinium **1a** with methyl 2-phenylacrylate, generating Giese product **7**. The yield of the reaction was determined by GC analysis using biphenyl as an internal standard. The reaction was performed in a quartz cuvette (path length: l = 1.0 cm) positioned 5 cm away from a single 0.1 W blue LED (λ_{max} = 450 nm).

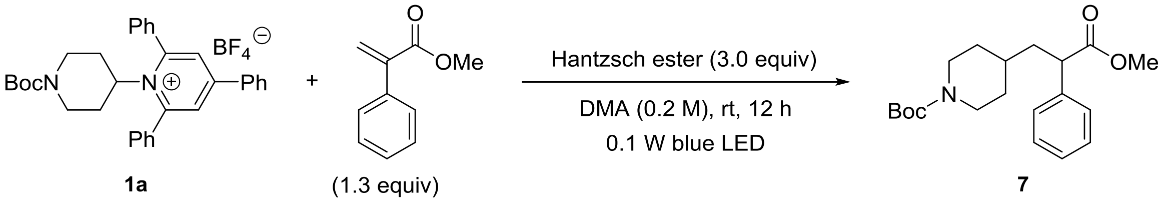

To a quartz cuvette (path length, l = 1 cm) equipped with a magnetic stir bar was added pyridinium **1a** (116 mg, 0.20 mmol), Hantzsch ester (152 mg, 0.60 mmol), methyl 2-phenylacrylate (42 mg, 0.26 mmol). The cuvette was purged with N_2 before being sealed with a septum. Degassed DMA (1.0 mL) was added and the cuvette was further sealed with parafilm. The cuvette was positioned 5 cm away from a single 0.1 W blue LED and the reaction was stirred and irradiated for 12 h. The yield was determined by GC analysis, using 1,3,5-trimethoxybenzene as an internal standard, to be 0.7% (1.40×10^{-6} mol).

The quantum yield (Φ) was then calculated using:

$$\Phi = \frac{\text{moles of product}}{\text{photon flux} \bullet \text{t} \bullet \text{f}}$$

Where t is the time (43200 s) and f is the fraction of light absorbed by the reaction mixture at λ = 450 nm, where $f = 1 - 10^{-A}$ (the absorbance of the reaction mixture (A) was determined by UV/Vis spectroscopy to be 1.12, thus f = 0.924). The photon flux of the LED setup was determined using standard ferrioxalate actinometry to be 4.31×10^{-10} einstein s^{-1}.[25-26]

$$\Phi = \frac{1.40 \times 10^{-6}}{4.31 \times 10^{-10} \bullet 43200 \bullet 0.924} = 0.0814$$

8.4. Side-product formation

1,4-Diphenethyl-2,4,6-triphenyl-1,4-dihydropyridine (**S1**)

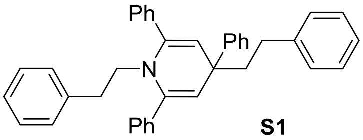

S1

^{1}H NMR (400 MHz, $CDCl_3$) δ 7.57 – 7.55 (m, 6H), 7.45 – 7.33 (m, 9H), 7.26 – 7.10 (m, 9H), 7.01 – 6.96 (m, 3H), 6.50 – 6.44 (m, 2H), 5.17 (s, 2H), 3.28 – 3.20 (m, 2H), 2.72 – 2.64 (m, 2H), 2.32 (dd, *J* = 6.7, 4.2 Hz, 2H), 2.20 – 2.12 (m, 2H).

^{13}C NMR (125 MHz, $CDCl_3$) δ 151.5, 143.2, 143.2, 139.3, 138.2, 129.2, 128.5, 128.5, 128.5, 128.5, 128.4, 128.4, 128.2, 128.2, 128.1, 126.5, 125.9, 125.7, 125.6, 125.4, 113.4, 50.1, 46.8, 44.0, 34.9, 32.8.

HRMS ($APCI^+$): calculated for $C_{39}H_{35}NO$ $[M+H]^+$ 518.2842, found 518.2841.

IR (ν_{max}/cm^{-1}, neat) 3079, 3058, 3025, 2925, 2856, 1662, 1600, 1494, 1453, 1445, 1387, 1276, 1261.

Methyl 2-((1-phenethyl-2,4,6-triphenyl-1,4-dihydropyridin-4-yl)methyl)acrylate (**S2**)

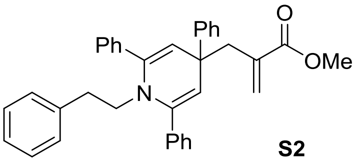

S2

^{1}H NMR (400 MHz, $CDCl_3$) δ 7.54 – 7.47 (m, 6H), 7.42 – 7.30 (m, 8H), 7.18 (tt, *J* = 7.8, 1.2 Hz, 2H), 7.02 – 6.95 (m, 3H), 6.49 – 6.42 (m, 2H), 6.27 (d, *J* = 1.7 Hz, 1H), 5.55 (d, *J* = 1.6 Hz, 1H), 5.02 (s, 2H), 3.55 (s, 3H), 3.18 – 3.10 (m, 2H), 2.96 (s, 2H), 2.36 – 2.28 (m, 2H).

^{13}C NMR (125 MHz, $CDCl_3$) δ 168.5, 150.9, 142.9, 139.4, 138.3, 138.3, 128.5, 128.5, 128.4, 128.4, 128.2, 128.1, 128.0, 126.7, 125.9, 112.4, 51.8, 49.9, 45.3, 44.7, 34.4.

HRMS (ESI^+): calculated for $C_{36}H_{34}NO_2$ $[M+H]^+$ 512.2584, found 512.2562.

IR (ν_{max}/cm^{-1}, neat) 3079, 3058, 3025, 2950, 2925, 2853, 1719, 1493, 1445, 1387, 1275, 1195, 1169.

Diethyl 4-(3-methoxy-3-oxo-2-phenylpropyl)-2,6-dimethyl-1,4-dihydropyridine-3,5-dicarboxylate (**S3**)

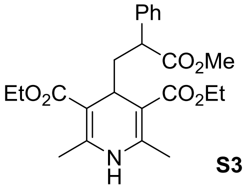

S3

^{1}H NMR (400 MHz, $CDCl_3$) δ 7.31 – 7.18 (m, 5H), 5.95 (br. s, 1H), 4.25 – 4.07 (m, 5H), 3.65 (dd, *J* = 9.0, 5.1 Hz, 1H), 3.61 (s, 3H), 2.37 (ddd, *J* = 14.0, 9.0, 6.4 Hz, 1H), 2.27 (s, 3H), 2.26 (s, 3H), 1.81 – 1.75 (m, 1H), 1.31 (t, *J* = 7.1 Hz, 3H), 1.26 (t, *J* = 7.1 Hz, 3H).

^{13}C NMR (100 MHz, $CDCl_3$) δ 174.7, 167.7, 167.6, 146.0, 145.9, 140.2, 128.5, 127.9, 127.0, 102.3, 102.2, 59.8, 52.1, 47.9, 39.1, 32.2, 19.6, 19.5, 14.5, 14.4.

HRMS (ESI^+): calculated for $C_{23}H_{30}NO_6$ $[M+H]^+$ 416.2068, found 416.2060;

calculated for $C_{23}H_{29}NO_6Na$ $[M+Na]^+$ 438.1887, found 438.1866.

IR (ν_{max}/cm^{-1}, neat) 3342 (br.), 3090, 3061, 2982, 2953, 1735, 1693, 1680, 1656, 1619, 1487, 1274, 1214, 1160, 1099.

Diethyl 4-(2-(methoxycarbonyl)allyl)-2,6-dimethyl-1,4-dihydropyridine-3,5-dicarboxylate (**S4**)

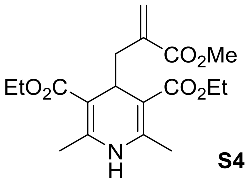

S4

^{1}H NMR (400 MHz, $CDCl_3$) δ 6.03 (d, J = 1.9 Hz, 1H), 5.63 (br. s, 1H), 5.32 – 5.25 (m, 1H), 4.16 (q, J = 7.0 Hz, 5H), 3.72 (s, 3H), 2.35 (d, J = 5.8 Hz, 2H), 2.26 (s, 6H), 1.29 (t, J = 7.1 Hz, 6H).

^{13}C NMR (125 MHz, $CDCl_3$) δ 168.1, 167.8, 145.5, 138.0, 127.3, 102.1, 59.7, 51.8, 38.1, 33.4, 19.5, 14.5.

HRMS (ESI^+): calculated for $C_{18}H_{26}NO_6$ $[M+H]^+$ 352.1755, found 352.1745;

calculated for $C_{18}H_{25}NO_6Na$ $[M+Na]^+$ 374.1574, found 374.1560.

IR (ν_{max}/cm^{-1}, neat) 3345 (br.), 3097, 2982, 2950, 1722, 1694, 1488, 1305, 1276, 1212, 1101.

8.5. Mechanism of Hantzsch ester-promoted reactions

The proposed mechanism for the Hantzsch ester-promoted Giese reaction is shown in Scheme S1.

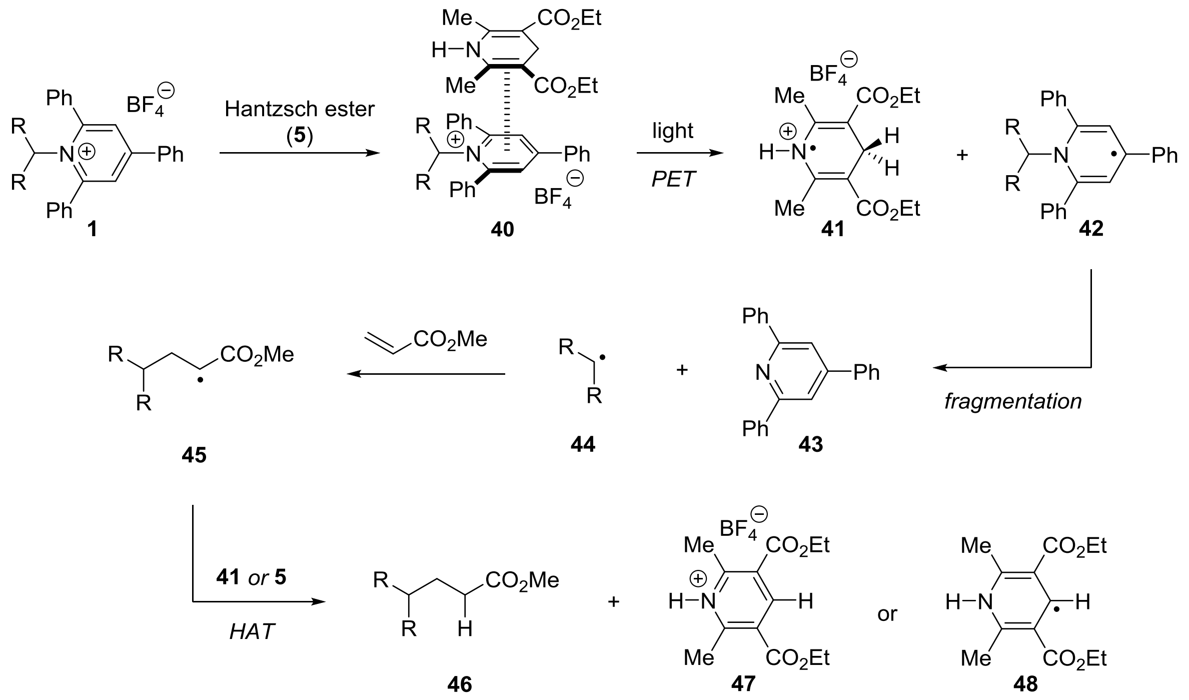

Scheme S1

This proposed mechanism is based on the observation of EDA complex (**40**) by UV/Vis spectroscopy, and the isolation of side-products **S1**, **S2**, **S3** and **S4**, which provide evidence for the formation of radical intermediates **42** and **48**.

Side-product **S1** is likely formed via a radical–radical coupling between dihydropyridine radical **S5** and primary alkyl radical **S6** (Scheme S2). This process becomes competitive due to the slow fragmentation of dihydropyridine radical **S5** when forming non-stabilized primary alkyl radicals. This results in a persistent radical,[27] which can react with the transient alkyl radical **S6**.

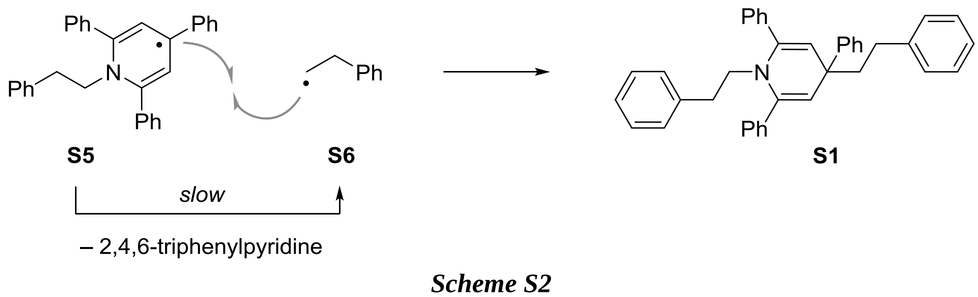

Scheme S2

Evidence for the slow fragmentation of dihydropyridine radical **S5** to form primary alkyl radical **S6** was further provided by the isolation of side-product **S2**, which is generated by addition of **S5** to allyl sulfone **49** (Scheme S3).

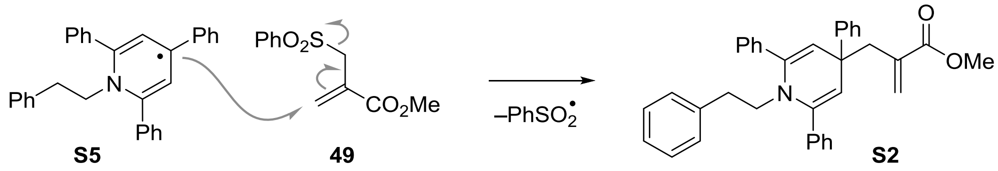

Scheme S3

Side-products **S3** and **S4** are formed by radical addition of dihydropyridine radical **48** to methyl 2-phenylacrylate and allyl sulfone **49**, respectively (Scheme S4).

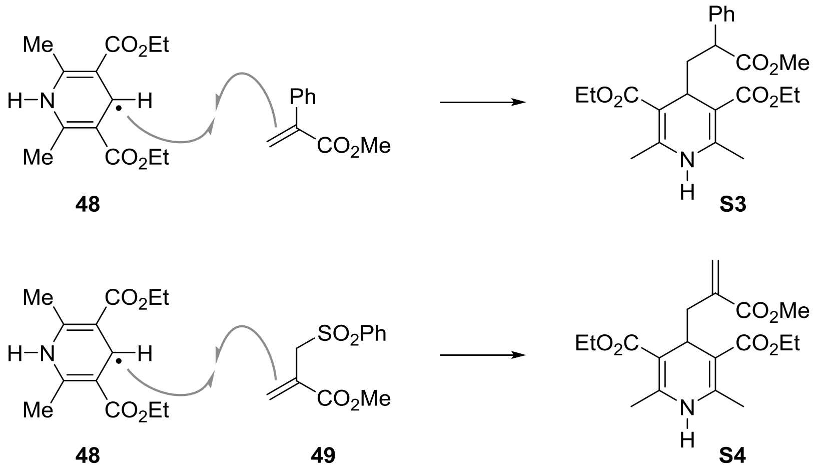

Scheme S4

The formation of radical **48** suggests that the HAT process to convert α-carbonyl radical **45** to Giese product **46** involves Hantzsch ester (Scheme S5). Based on the bond dissociation free energies (BDFE), HAT is thermodynamically favourable with both Hantzsch ester and the radical cation of Hantzsch ester (**41**).[28,29] However, due to the relatively low concentration of radical cation **41**, it is likely that the predominant pathway involves HAT from Hantzsch ester, generating dihydropyridine radical **48**.

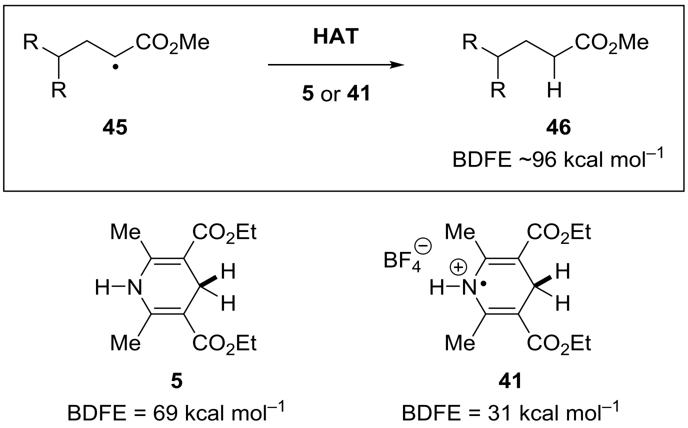

Scheme S5

The formation of dihydropyridine radical **48** prompted us to consider the possibility of a radical chain mechanism, in which SET between **48** and the pyridinium **1** provides oxidised Hantzsch ester (**47**) and dihydropyridine radical **42** (Scheme S6). However, this SET is thermodynamically disfavoured (*E* vs. SCE in MeCN for **48** = –0.75 V;[30] for **1** ≈ –1.0 V)[31] by 5.8 kcal mol^{-1}. Furthermore, measurement of a quantum yield of 0.08 implies that if a radical chain is operative, it is a minor pathway.

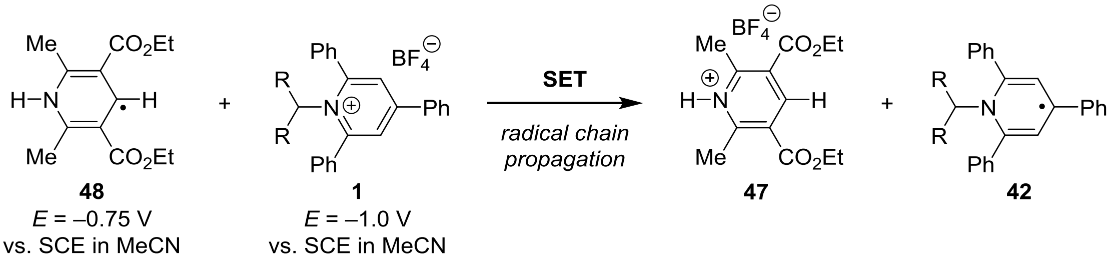

Scheme S6

An alternative mechanism was also considered involving direct photoexcitation of Hantzsch ester to form a highly reducing species **S7** (E = –2.28 vs. SCE in DMF)[32], which could undergo single electron transfer with pyridinium **1** ($E \approx -1.0$ V vs. SCE in MeCN)[31] to form the Hantzsch ester radical cation **41** and dihydropyridine radical **42** (Scheme S7). We attempted to investigate this possibility by performing fluorescence quenching studies, however, this was complicated by overlapping peaks in the absorption and emission spectra of Hantzsch ester (**5**) and cyclohexylamine-derived pyridinium **36**. In particular, the emission spectra for these two species in MeCN have maxima between 460 and 470 nm, making the peaks indistinguishable.

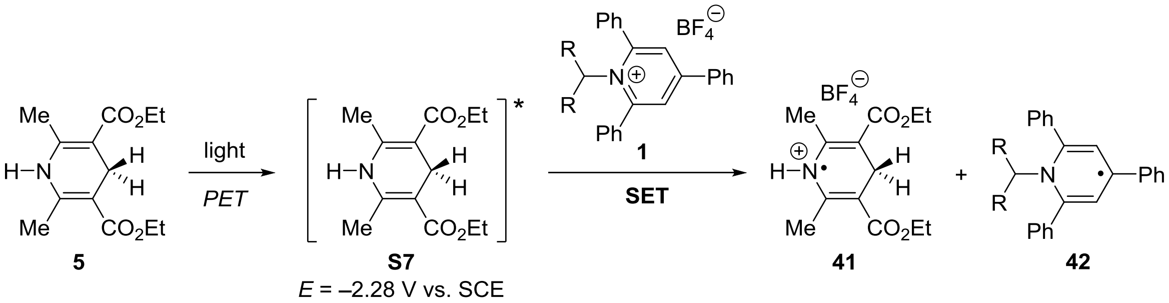

Scheme S7

While it was not possible to study the possibility of an outer sphere electron transfer by fluorescence quenching experiments, our UV/Vis analysis clearly shows EDA complex formation between the pyridinium and Hantzsch ester. Therefore, we believe that the inner sphere electron transfer (after initial EDA complex formation) is the most plausible mechanism.

Reactions with allyl sulfones:

For the reactions with allyl sulfones, after formation of alkyl radical **44**, addition to allyl sulfone **49** gives the β-sulfonyl radical **S8** (Scheme S8). Elimination of sulfinyl radical **S9** then provides the allylated product **S10**. Sulfinyl radical **S9** could then abstract a hydrogen atom from either Hantzsch ester (**5**) or Hantzsch ester radical cation **41** to form benzenesulfinic acid (**S11**).

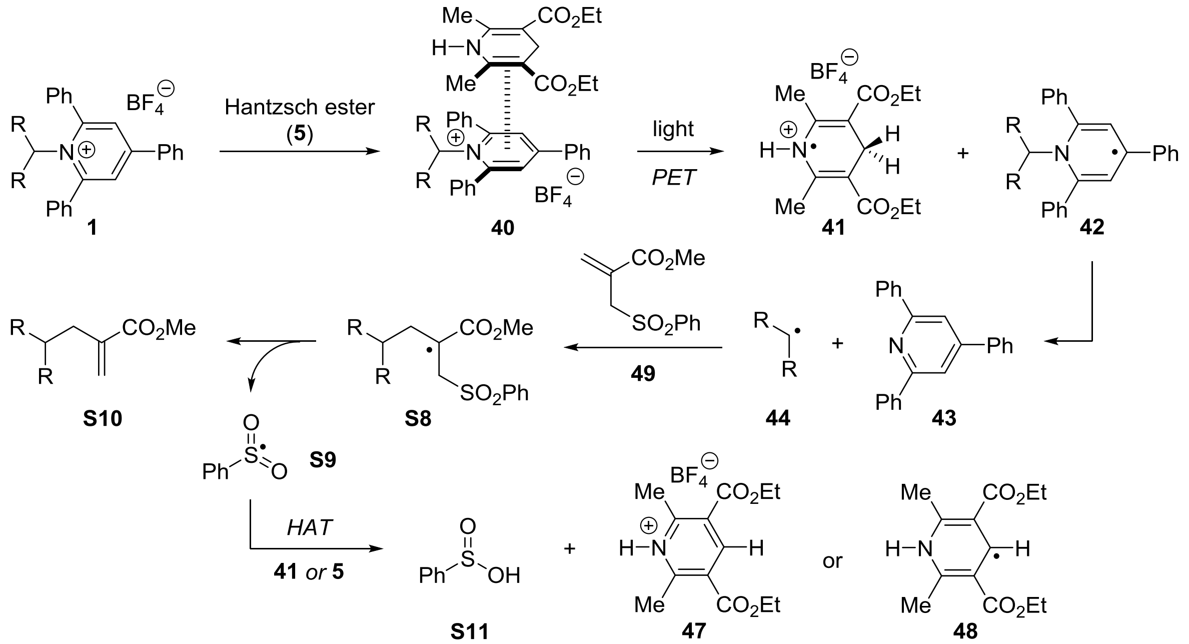

Scheme S8

8.6. Mechanism of Et3N-promoted reactions

For the reaction promoted by Et3N, UV/Vis spectroscopy confirms the formation of an EDA complex between the pyridinium and Et3N. Therefore, we propose the mechanisms shown in Scheme S9.

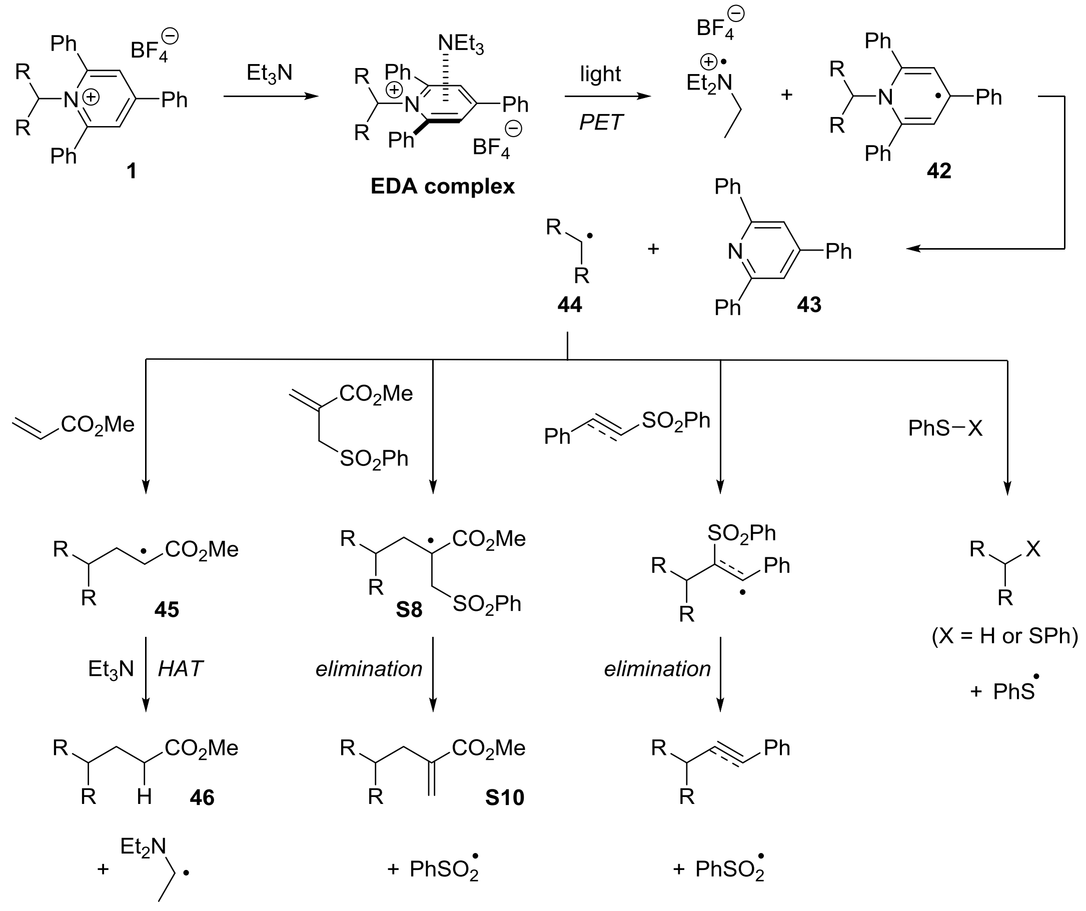

Scheme S9

For the addition–elimination reactions with allyl, alkenyl or alkynyl sulfones, and for the homolytic substitution reactions with diphenyldisulfide or thiophenol, these processes are efficiently promoted by Et3N. However, the Giese reaction promoted by Et3N is less efficient, which is likely because it relies on a HAT process between the α-carbonyl radical **45** and the reductant (Scheme S10). For the reactions with Hantzsch ester, the weak C–H bond (BDFE = 69 kcal mol^{-1})[28] enables efficient HAT to generate the Giese product **46** (BDFE ~96 kcal mol^{-1}).[29] When only Et3N (BDFE = 91 kcal mol^{-1}) is used, this HAT becomes less exergonic and thus less efficient, resulting in lower yields.[33]

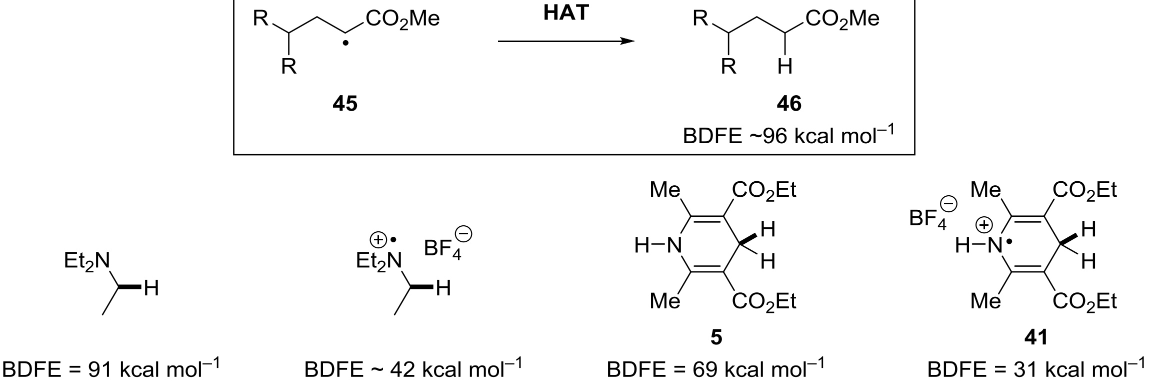

Scheme S10

9. References

1. Wu, J.; He, L.; Noble, A.; Aggarwal, V. K. *J. Am. Chem. Soc.* **2018**, *140*, 10700-10704.
2. Gualandi, A.; Matteucci, E.; Monti, F.; Baschieri, A.; Armaroli, N.; Sambri, L.; Cozzi, P. G. *Chem. Sci.* **2017**, *8*, 1613-1620.
3. Darmency, V.; Scanlan, E. M.; Schaffner, A. P.; Renaud, P. *Org. Synth.* **2006**, *83*, 24-30.
4. Meesin, J.; Katrun, P.; Pareseecharoen, C.; Pohmakotr, M.; Reutrakul, V.; Soorukram, D.; Kuhakarn, C. *J. Org. Chem.* **2016**, *81*, 2744-2752.
5. Noble, A.; MacMillan, D. W. C. *J. Am. Chem. Soc.* **2014**, *136*, 11602-11605.
6. Kamijo, S.; Kamijo, K.; Maruoka, K.; Murafuji, T. *Org. Lett.* **2016**, *18*, 6516-6519.
7. Moure, A. L.; Mauleón, P.; Gómez Arrayás, R.; Carretero, J. C. *Org. Lett.* **2013**, *15*, 2054-2057.
8. Paul, S.; Guin, J. *Green Chem.* **2017**, *19*, 2530-2534.
9. Klein, S. I.; Molino, B. F.; Czekaj, M.; Gardner, C. J.; Chu, V.; Brown, K.; Sabatino, R. D.; Bostwick, J. S.; Kasiewski, C.; Bentley, R.; Windisch, V.; Perrone, M.; Dunwiddie, C. T.; Leadley, R. J. *J. Med. Chem.* **1998**, *41*, 2492-2502.
10. Song, X.; He, H. T.; Siahaan, T. J. *Org. Lett.* **2002**, *4*, 549-552.
11. Breman, A. C.; Ruiz-Olalla, A.; van Maarseveen, J. H.; Ingemann, S.; Hiemstra, H., *Eur. J. Org. Chem.* **2014**, *2014*, 7413-7425.
12. Wang, L.; Thai, K.; Gravel, M. *Org. Lett.* **2009**, *11*, 891-893.
13. Lu, B.; Zhu, F.; Sun, H.-M.; Shen, Q. *Org. Lett.* **2017**, *19*, 1132-1135.
14. Shukla, P.; Sharma, A.; Pallavi, B.; Cheng, C. H. *Tetrahedron* **2015**, *71*, 2260-2266.
15. Mao, J.; Liu, F.; Wang, M.; Wu, L.; Zheng, B.; Liu, S.; Zhong, J.; Bian, Q.; Walsh, P. J. *J. Am. Chem. Soc.* **2014**, *136*, 17662-17668.
16. Sumino, S.; Uno, M.; Huang, H.-J.; Wu, Y.-K.; Ryu, I. *Org. Lett.* **2018**, *20*, 1078-1081.
17. Lai, Y.-L.; Huang, J.-M. *Org. Lett.* **2017**, *19*, 2022-2025.
18. Liu, X.-G.; Zhou, C.-J.; Lin, E.; Han, X.-L.; Zhang, S.-S.; Li, Q.; Wang, H. *Angew. Chem. Int. Ed.* **2018**, *57*, 13096-13100.
19. Koy, M.; Sandfort, F.; Tlahuext-Aca, A.; Quach, L.; Daniliuc, C. G.; Glorius, F. *Chem. Eur. J.* **2018**, *24*, 4552-4555.
20. Stead, D.; Carbone, G.; O'Brien, P.; Campos, K. R.; Coldham, I.; Sanderson, A. *J. Am. Chem. Soc.* **2010**, *132*, 7260-7261.
21. Knutsen, L. J. S.; Lau, J.; Petersen, H.; Thomsen, C.; Weis, J. U.; Shalmi, M.; Judge, M. E.; Hansen, A. J.; Sheardown, M. J. *J. Med. Chem.* **1999**, *42*, 3463-3477.
22. Tan, W. W.; Ong, Y. J.; Yoshikai, N. *Angew. Chem. Int. Ed.* **2017**, *56*, 8240-8244.
23. Lu, Z.; Yang, Y.-Q.; Li, H.-X. *Synthesis* **2016**, *48*, 4221-4227.
24. Alkayar, Z. T. I.; Adams, H.; Coldham, I. *Synlett* **2016**, *27*, 447-449.
25. Cismesia, M. A.; Yoon, T. P. *Chem. Sci.* **2015**, *6*, 5426-5434.
26. Silvi, M.; Schrof, R.; Noble, A.; Aggarwal V. K. *Chem. Eur. J.* **2018**, *24*, 4279-4282.
27. Okada, K.; Matsumoto, K.; Oda, M.; Murai, H.; Akiyama, K.; Ikegami, Y. *Tetrahedron Lett.* **1995**, *36*, 6689-6692.

28. Zhu, X.-Q.; Li, H.-R.; Li, Q.; Ai, T.; Lu, J.-Y.; Yang, Y.; Cheng, J.-P. *Chem. Eur. J.* **2003**, *9*, 871-880.

29. Brocks, J. J.; Beckhaus, H.-D.; Beckwith, A. L. J.; Rüchardt, C. *J. Org. Chem.* **1998**, *63*, 1935-1943.

30. Rono, L. J.; Yayla, H. G.; Wang, D. Y.; Armstrong, M. F.; Knowles, R. R. *J. Am. Chem. Soc.* **2013**, *135*, 17735-17738.

31. Sandfort, F.; Strieth-Kalthoff, F.; Klauck, F. J. R.; James, M. J.; Glorius, F. *Chem. Eur. J.* **2018**, *24*, 17210-17214.

32. Jung, J.; Kim, J.; Park, G.; You, Y.; Cho, E. J. *Adv. Synth. Catal.* **2016**, *358*, 74-80.

33. Beatty, J. W.; Corey R. J. Stephenson, C. J. *Acc. Chem. Res.* **2015**, *48*, 1474-1484.

10. NMR Spectra

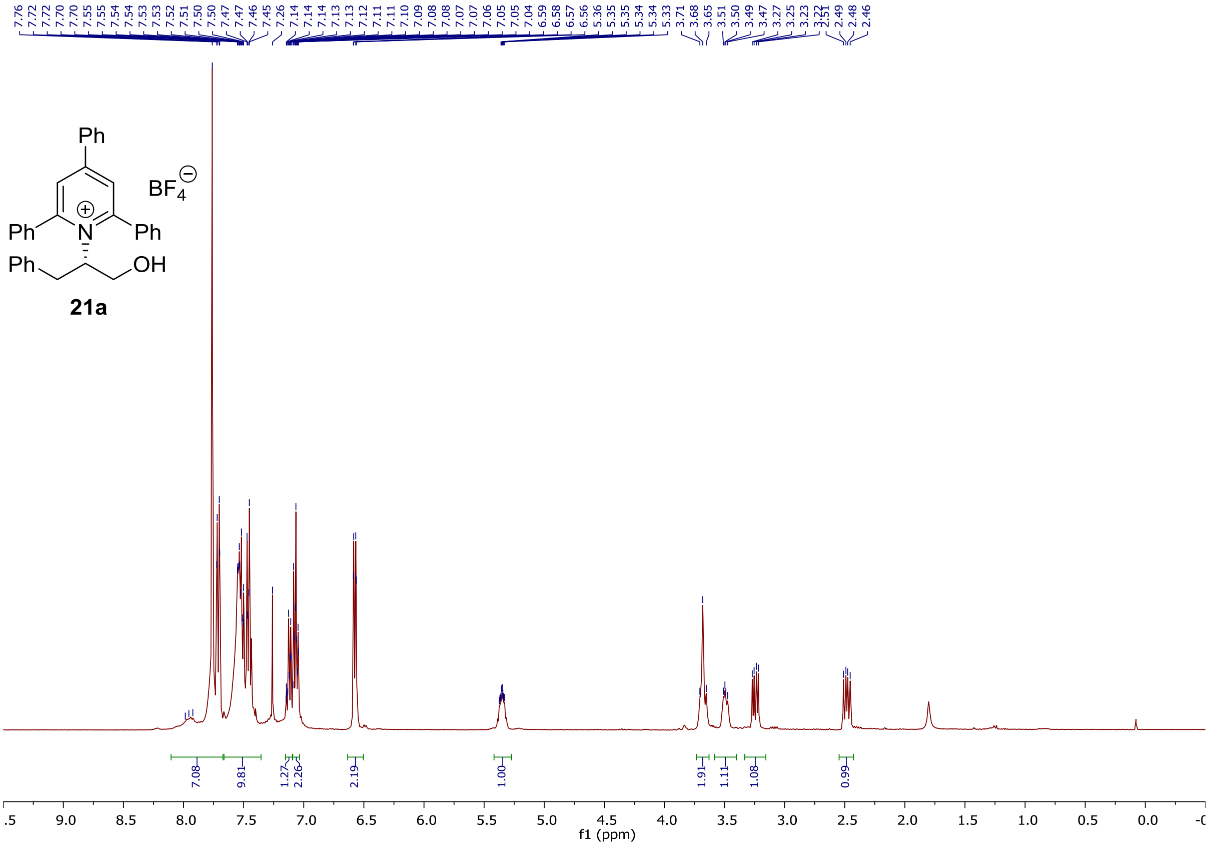

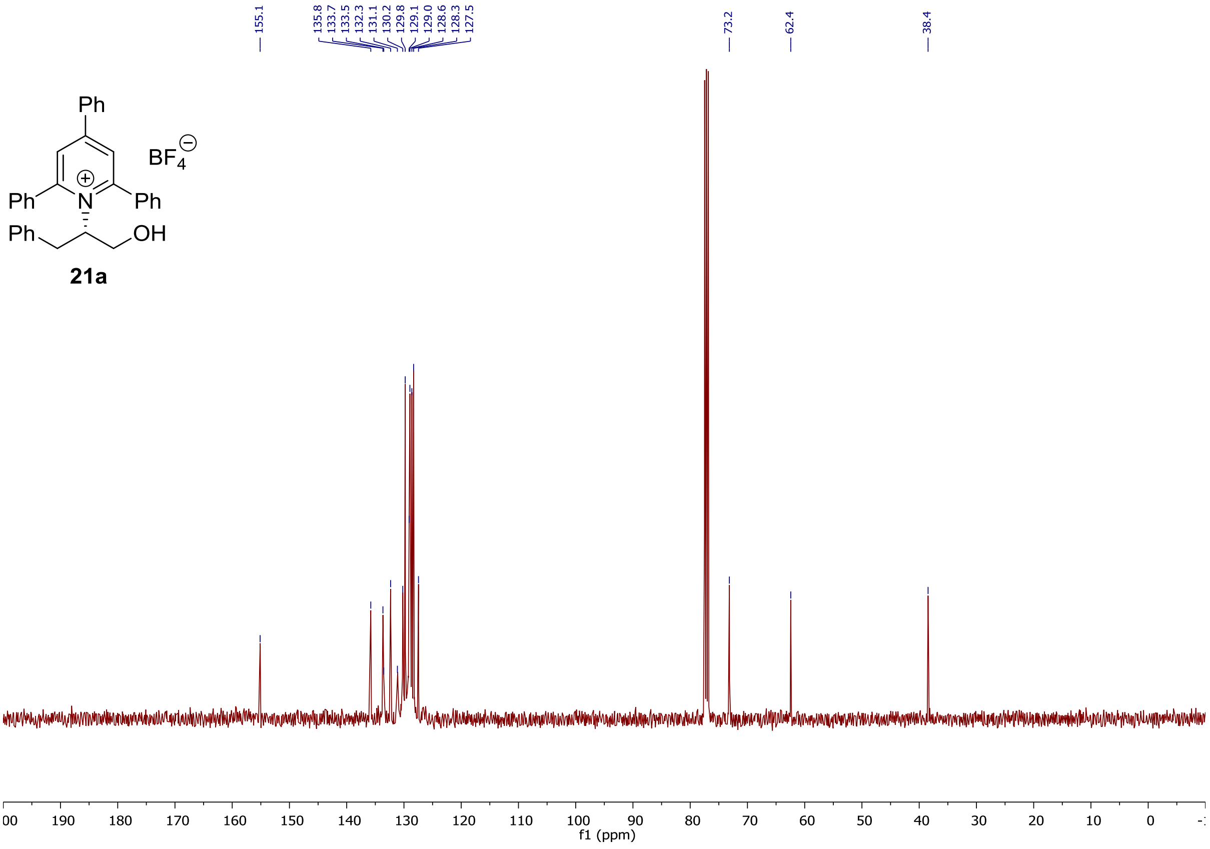

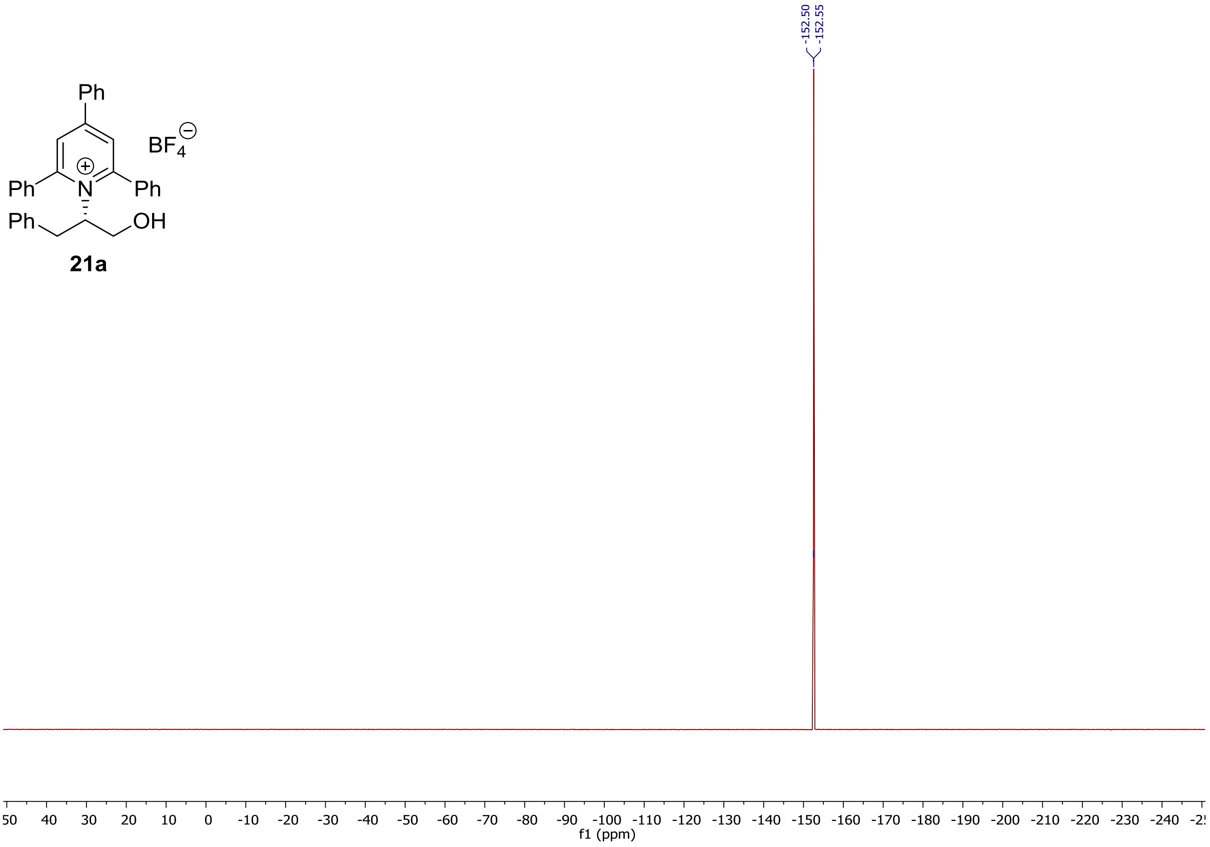

Ph
BF4
Ph
Ph
Ph
OH
N
21a
-152.50
-152.55
f1 (ppm)

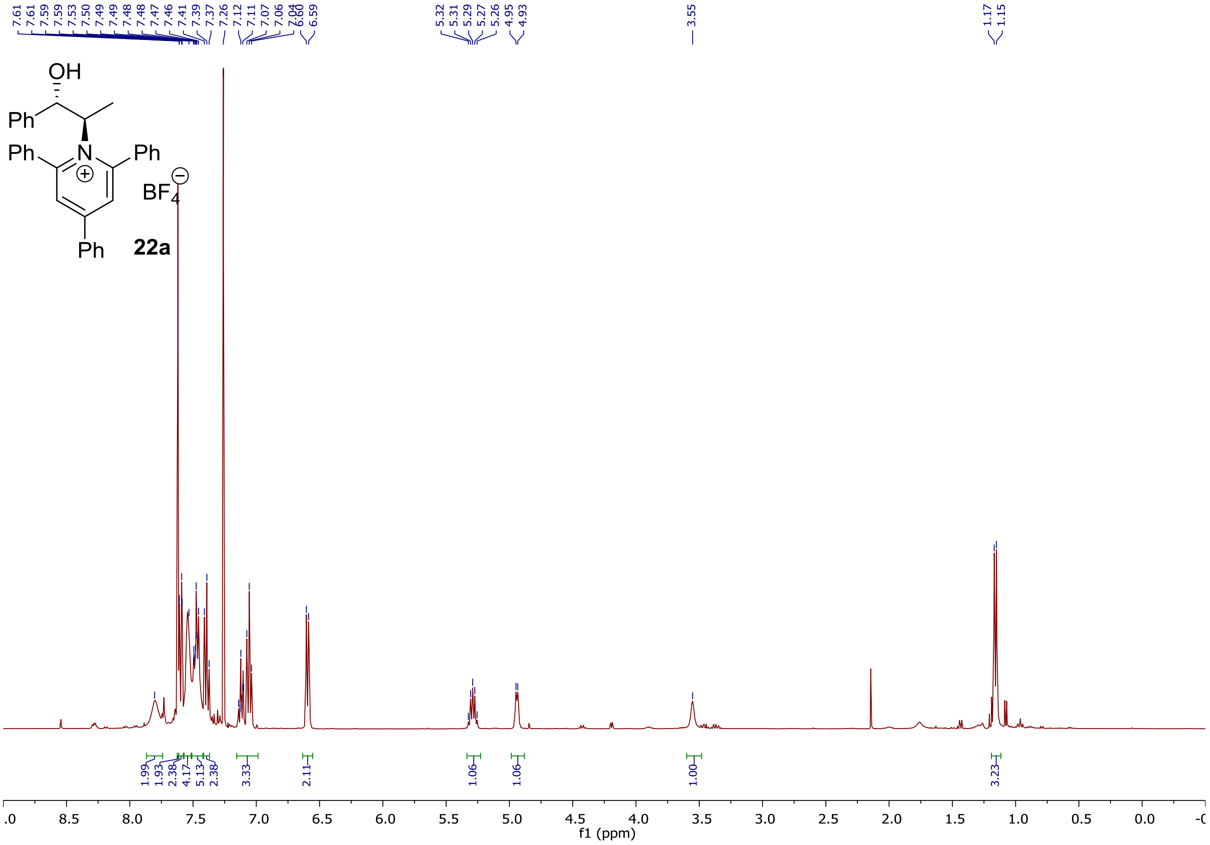

OH
Ph
Ph
Ph
N
BF4
Ph
22a
7.61
7.61
7.59
7.59
7.53
7.50
7.49
7.49
7.48
7.48
7.47
7.46
7.41
7.39
7.37
7.26
7.12
7.11
7.07
7.06
6.59
5.32
5.31
5.29
5.27
5.26
4.95
4.93
3.55
1.17
1.15
1.99
1.93
2.38
4.17
5.13
2.38
3.33
2.11
1.06
1.06
1.00
3.23
f1 (ppm)

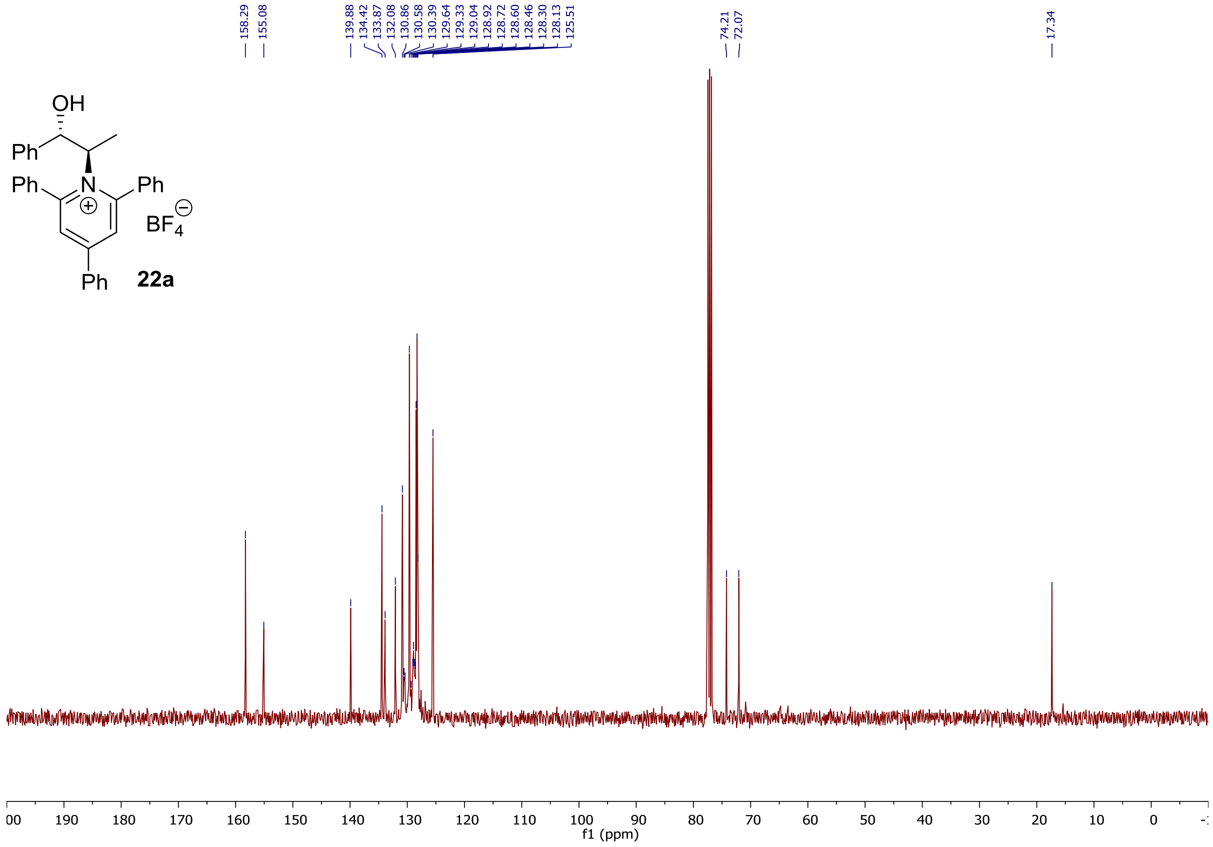
OH
Ph
Ph
N
Ph
BF4
Ph
22a
158.29
155.08
139.88
134.42
133.87
132.08
130.86
130.58
130.39
129.64
129.33
129.04
128.92
128.72
128.60
128.46
128.30
128.13
125.51
74.21
72.07
17.34
f1 (ppm)

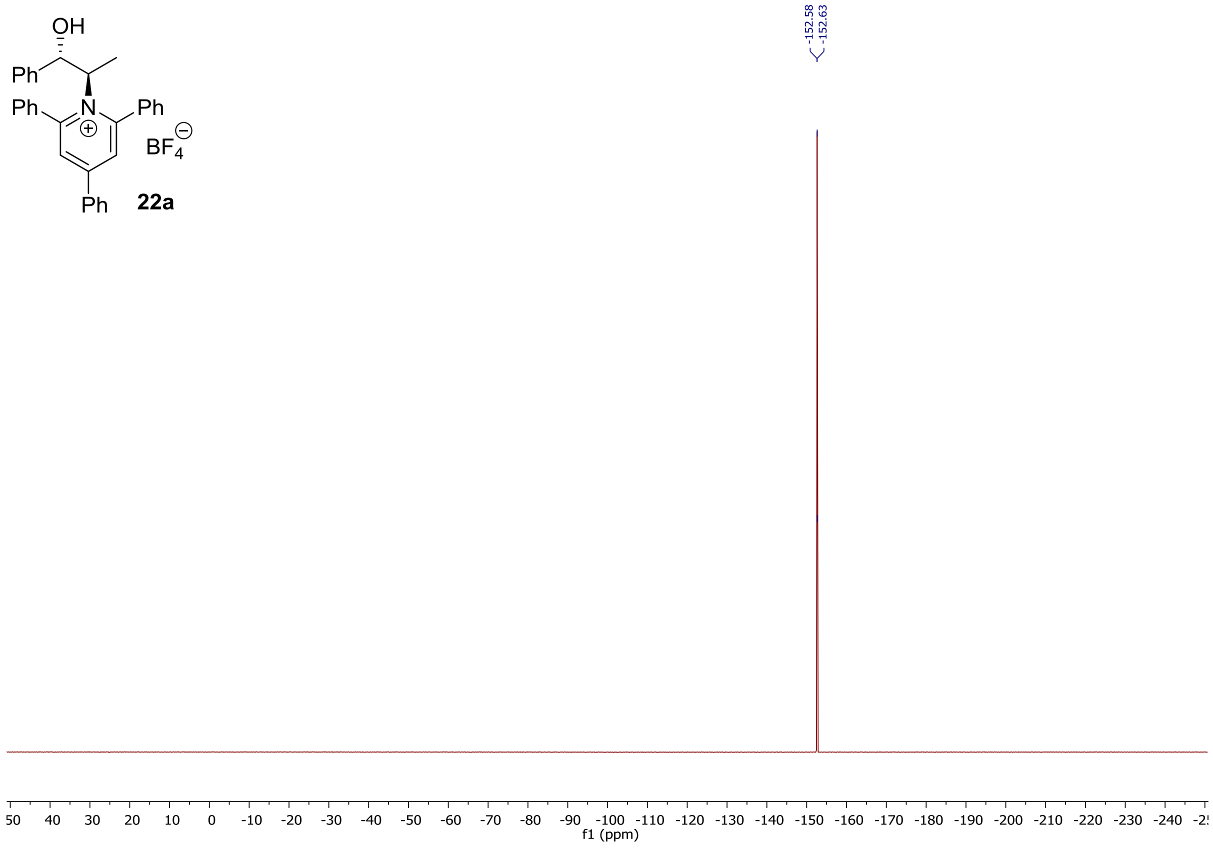
OH
Ph
Ph
N
Ph
BF4
Ph
22a
-152.58
-152.63
f1 (ppm)

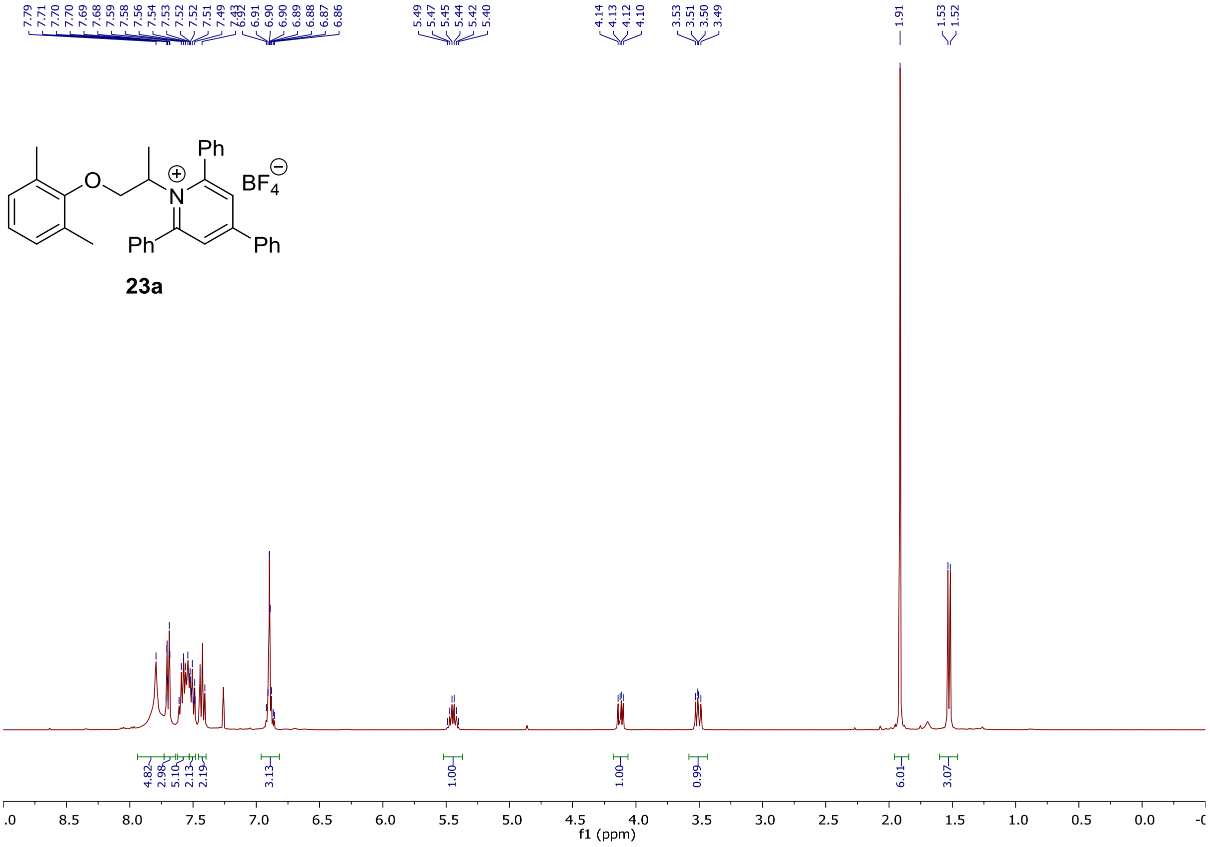
7.79 7.71 7.70 7.69 7.68 7.59 7.58 7.56 7.54 7.53 7.52 7.52 7.51 7.49 7.43 6.92 6.91 6.90 6.90 6.89 6.88 6.87 6.86
5.49 5.47 5.45 5.44 5.42 5.40
4.14 4.13 4.12 4.10
3.53 3.51 3.50 3.49
1.91
1.53 1.52
Ph
BF4
Ph
Ph
23a
4.82 2.98 5.10 2.13 2.19
3.13
1.00
1.00
0.99
6.01
3.07
f1 (ppm)

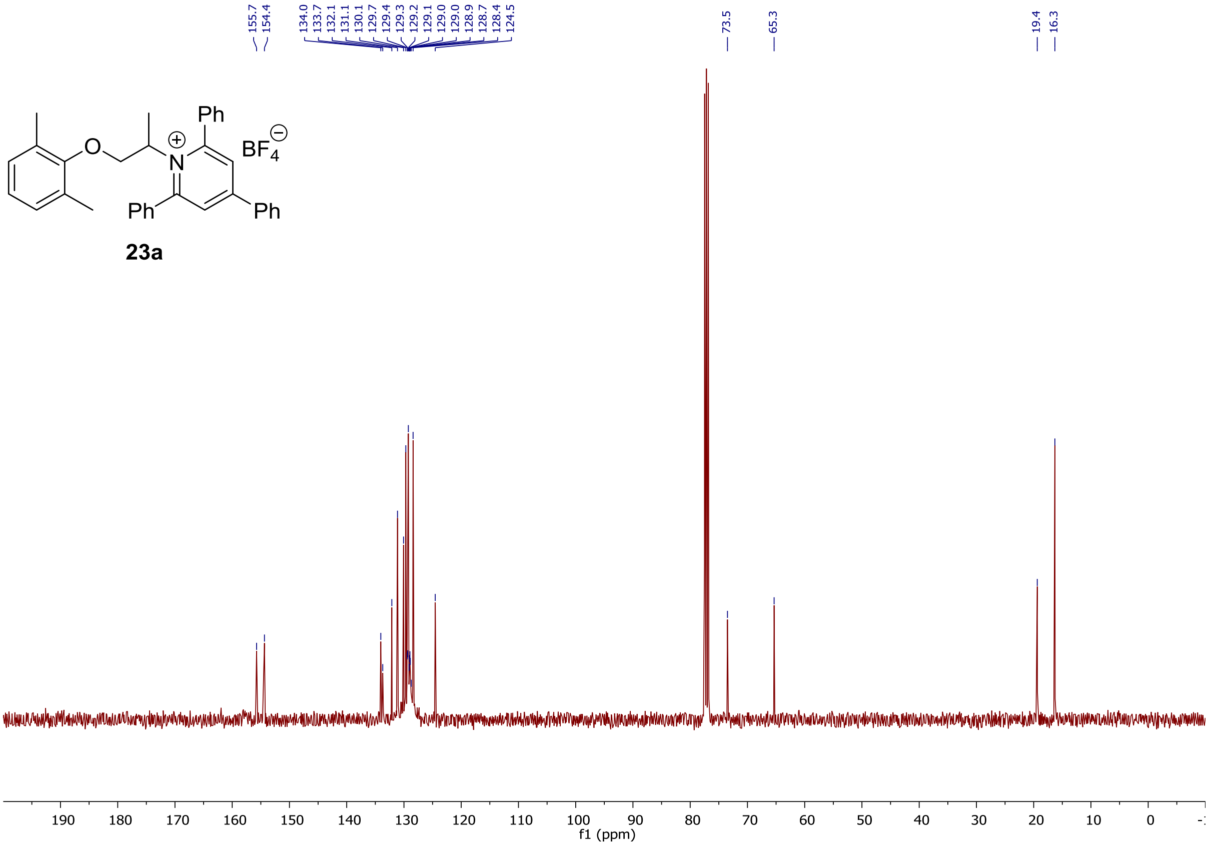
155.7 154.4
134.0 133.7 132.1 131.1 130.1 129.7 129.4 129.3 129.2 129.1 129.0 129.0 128.9 128.7 128.4 124.5
73.5
65.3
19.4
16.3
Ph
BF4
Ph
Ph
23a
f1 (ppm)

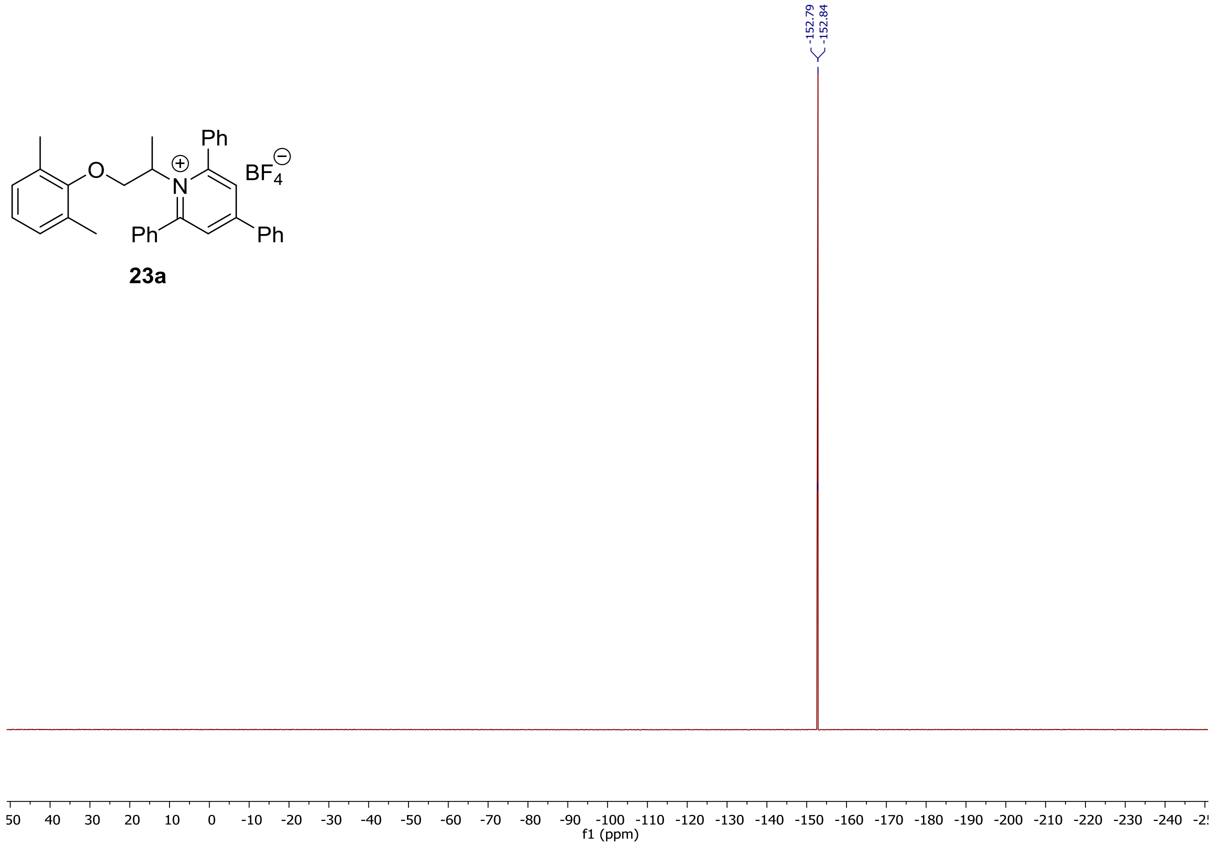
-152.79
-152.84
Ph
BF4
Ph
Ph
23a
50 40 30 20 10 0 -10 -20 -30 -40 -50 -60 -70 -80 -90 -100 -110 -120 -130 -140 -150 -160 -170 -180 -190 -200 -210 -220 -230 -240
f1 (ppm)

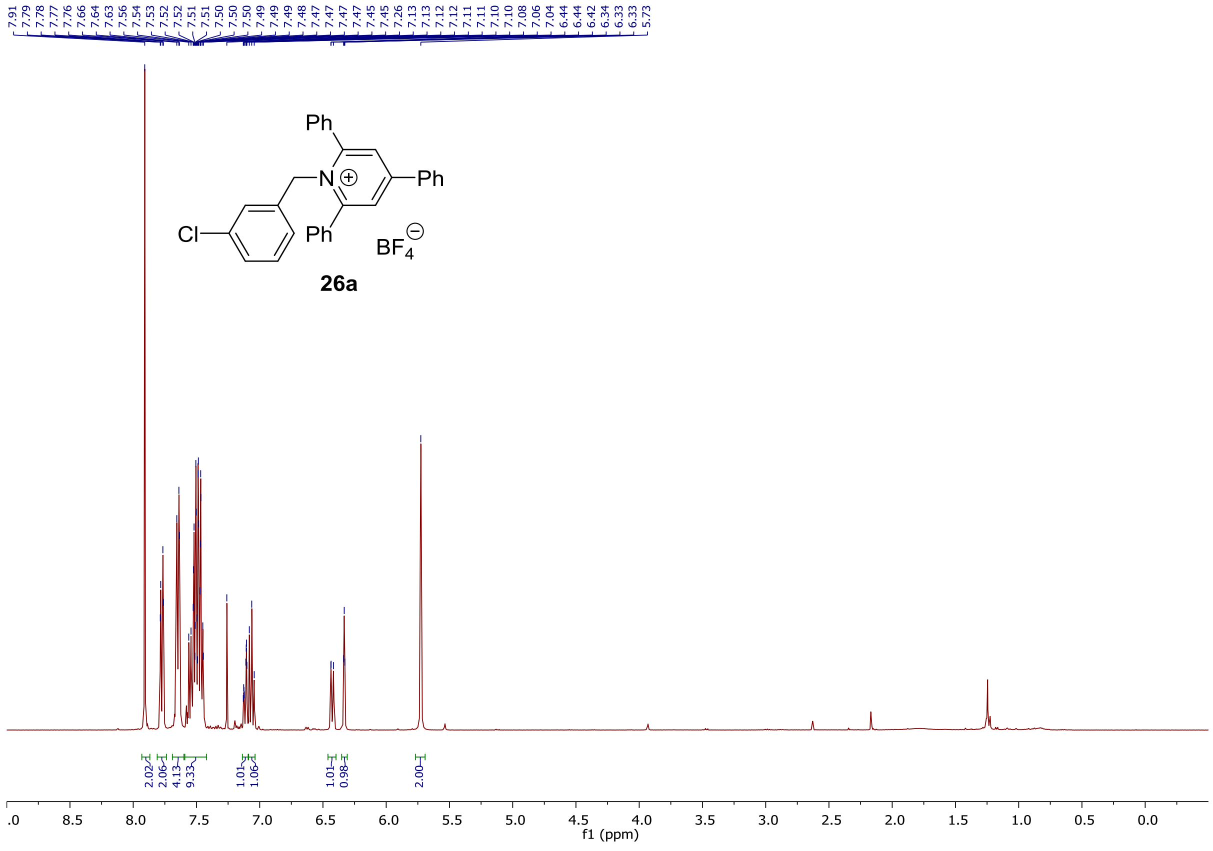
7.91 7.79 7.78 7.77 7.76 7.66 7.64 7.63 7.56 7.54 7.53 7.52 7.52 7.51 7.51 7.50 7.50 7.50 7.49 7.49 7.49 7.48 7.47 7.47 7.47 7.47 7.45 7.45 7.26 7.13 7.13 7.12 7.12 7.11 7.11 7.10 7.10 7.08 7.06 7.04 6.44 6.44 6.42 6.34 6.33 6.33 5.73
Ph
Ph
Cl
Ph
BF4
26a
2.02 2.06 4.13 9.33 1.01 1.06 1.01 0.98 2.00
8.5 8.0 7.5 7.0 6.5 6.0 5.5 5.0 4.5 4.0 3.5 3.0 2.5 2.0 1.5 1.0 0.5 0.0
f1 (ppm)

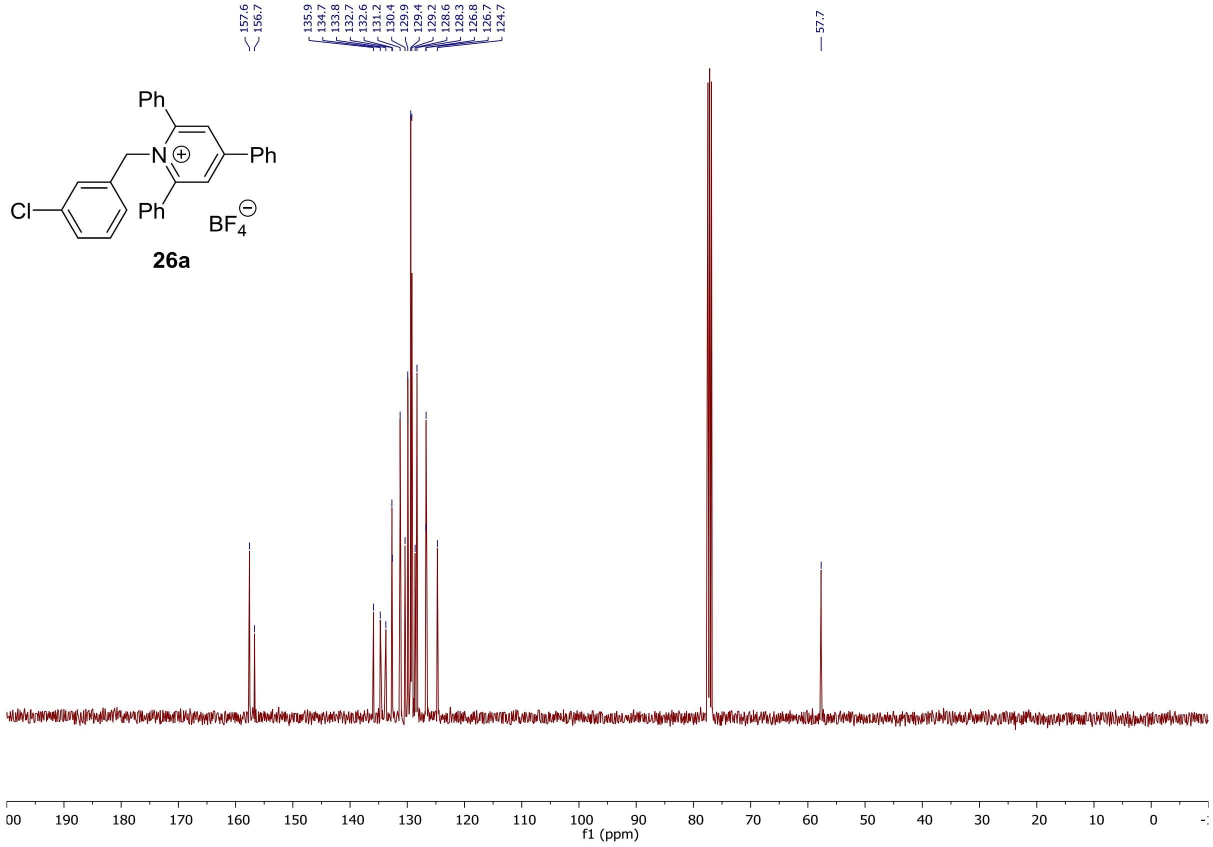
157.6
156.7
135.9
134.7
133.8
132.7
132.6
131.2
130.4
129.9
129.4
129.2
128.6
128.3
126.8
126.7
124.7
57.7
Ph
N
Ph
Cl
Ph
BF4
26a
f1 (ppm)

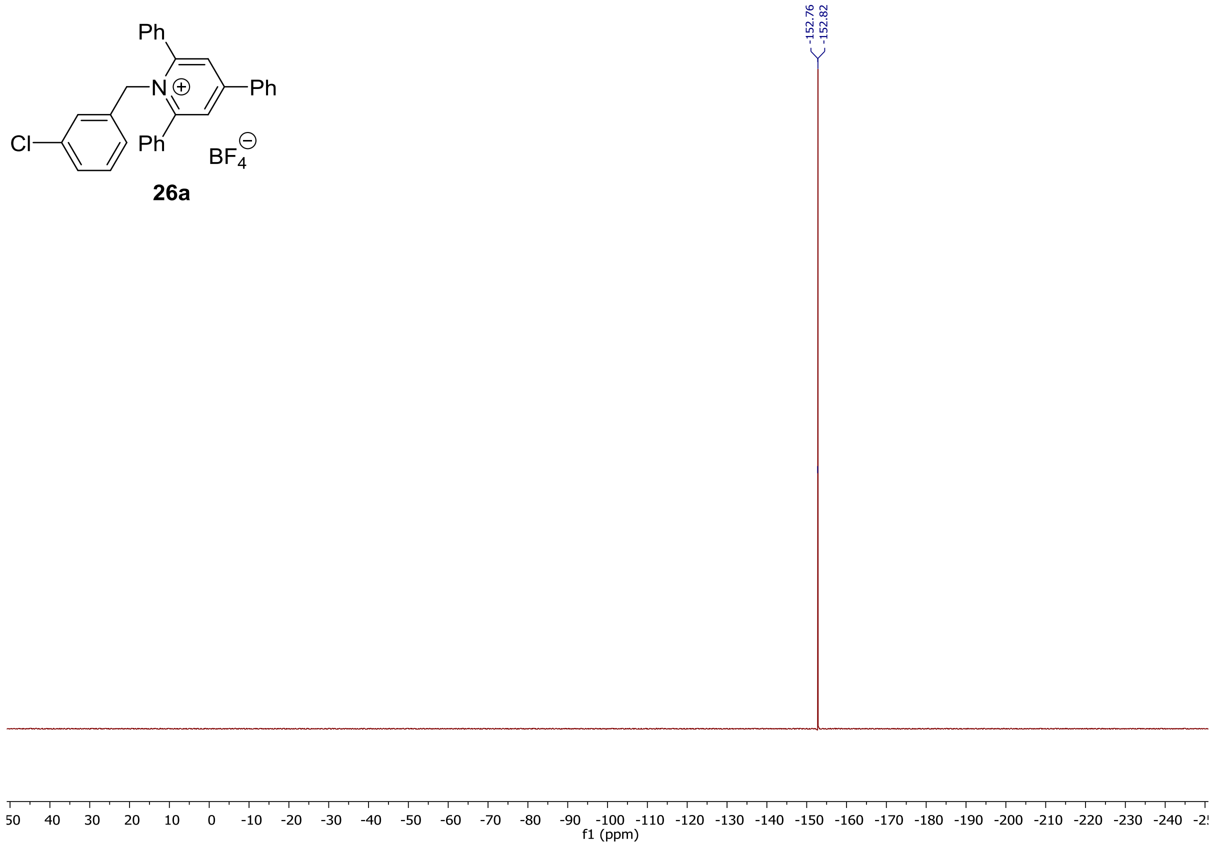
-152.76
-152.82
Ph
N
Ph
Cl
Ph
BF4
26a
f1 (ppm)

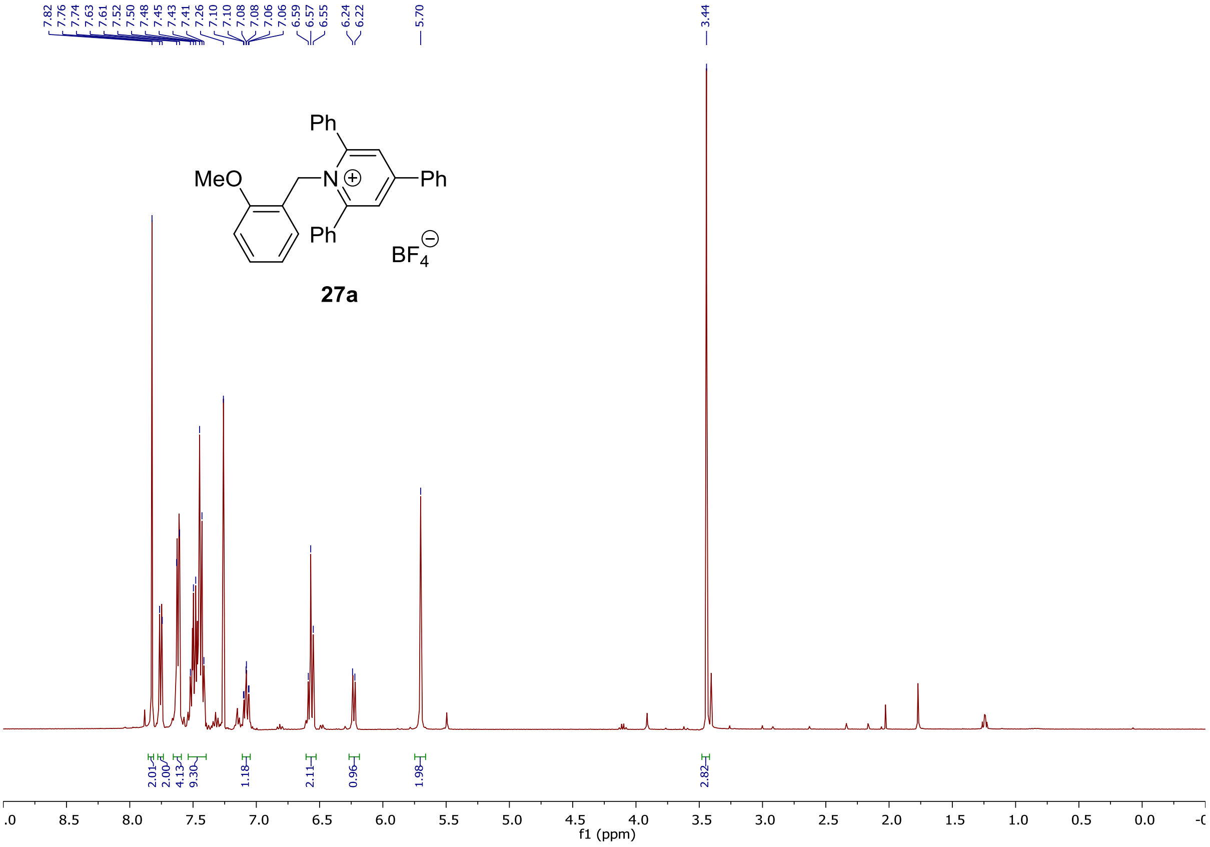
7.82 7.76 7.74 7.63 7.61 7.52 7.50 7.48 7.45 7.43 7.41 7.26 7.10 7.10 7.08 7.08 7.06 7.06 6.59 6.57 6.55 6.24 6.22
5.70
3.44
Ph
MeO
N
Ph
Ph
BF_4
27a
2.01 2.00 4.13 9.30 1.18 2.11 0.96 1.98 2.82
f1 (ppm)

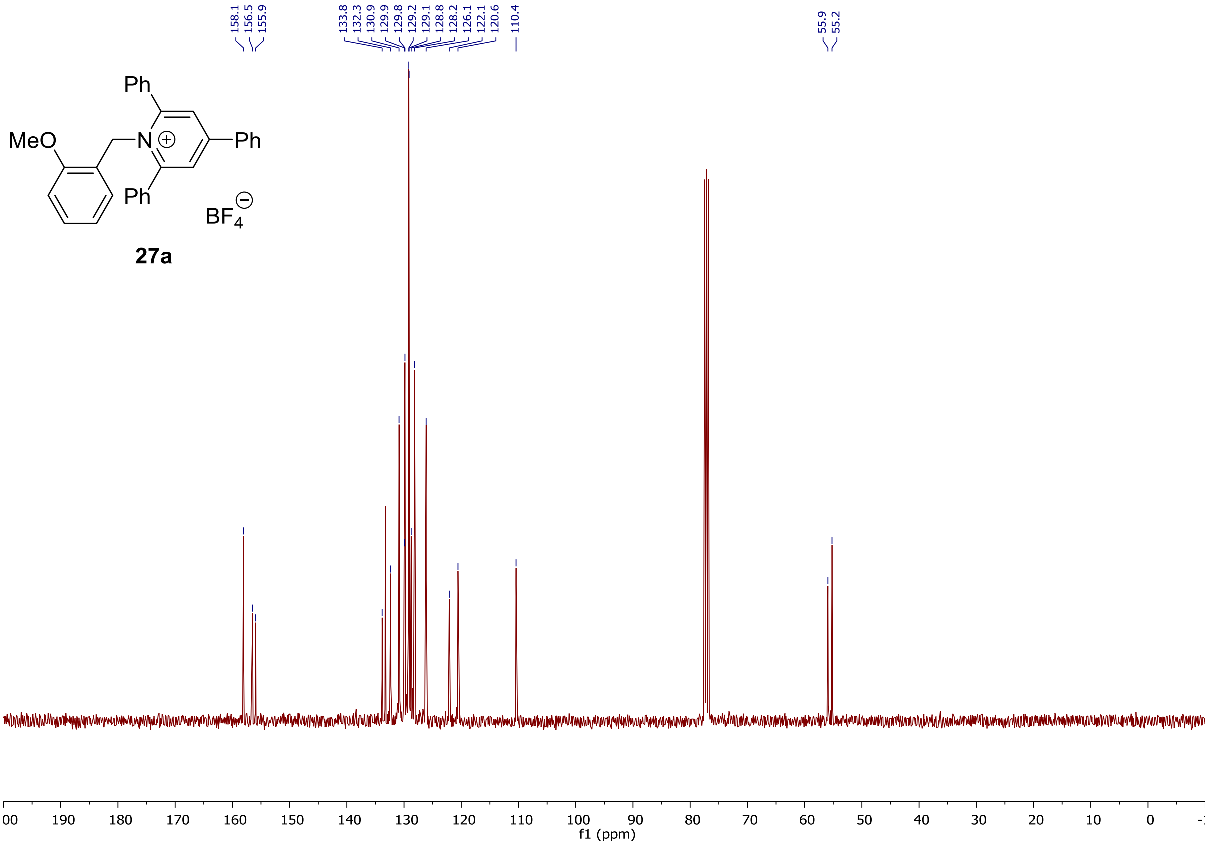
158.1 156.5 155.9
133.8 132.3 130.9 129.9 129.8 129.2 129.1 128.8 128.2 126.1 122.1 120.6
110.4
55.9 55.2
Ph
MeO
N
Ph
Ph
BF_4
27a
f1 (ppm)

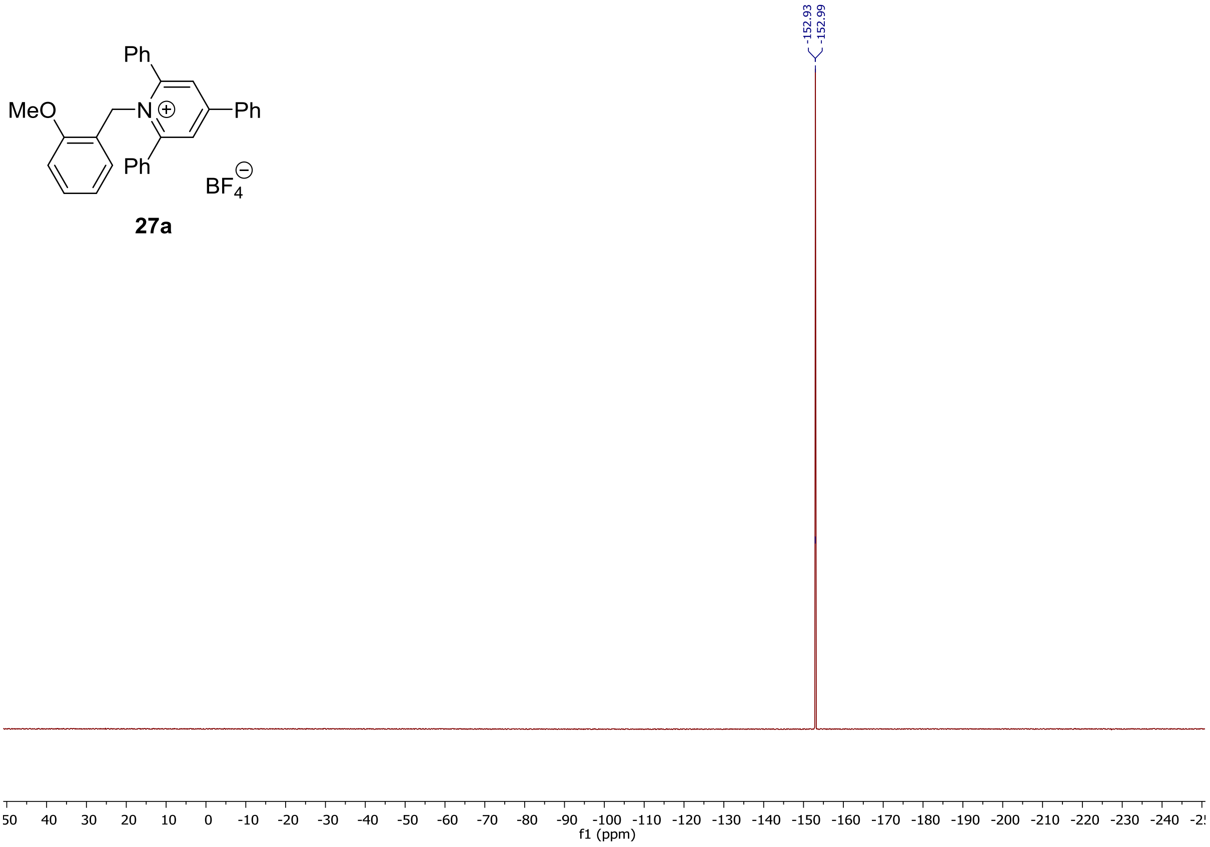
-152.93
-152.99
MeO
Ph
N
Ph
Ph
BF4
27a
f1 (ppm)

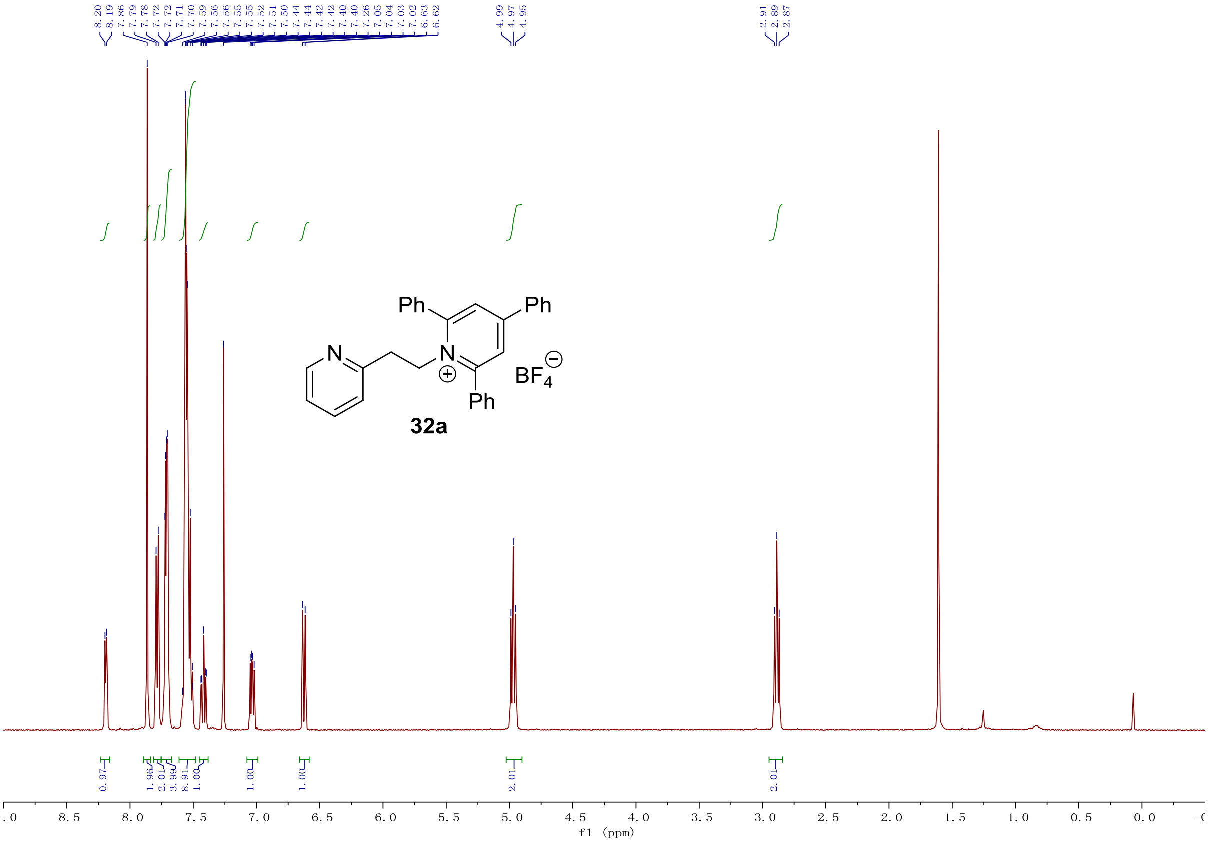
8.20
8.19
7.86
7.79
7.78
7.72
7.72
7.71
7.70
7.59
7.56
7.56
7.55
7.55
7.52
7.51
7.50
7.44
7.44
7.42
7.42
7.40
7.40
7.26
7.05
7.04
7.03
7.02
6.63
6.62
4.99
4.97
4.95
2.91
2.89
2.87
N
Ph
N
Ph
Ph
BF4
32a
0.97
1.96
2.01
3.98
8.91
1.00
1.00
1.00
2.01
2.01
f1 (ppm)

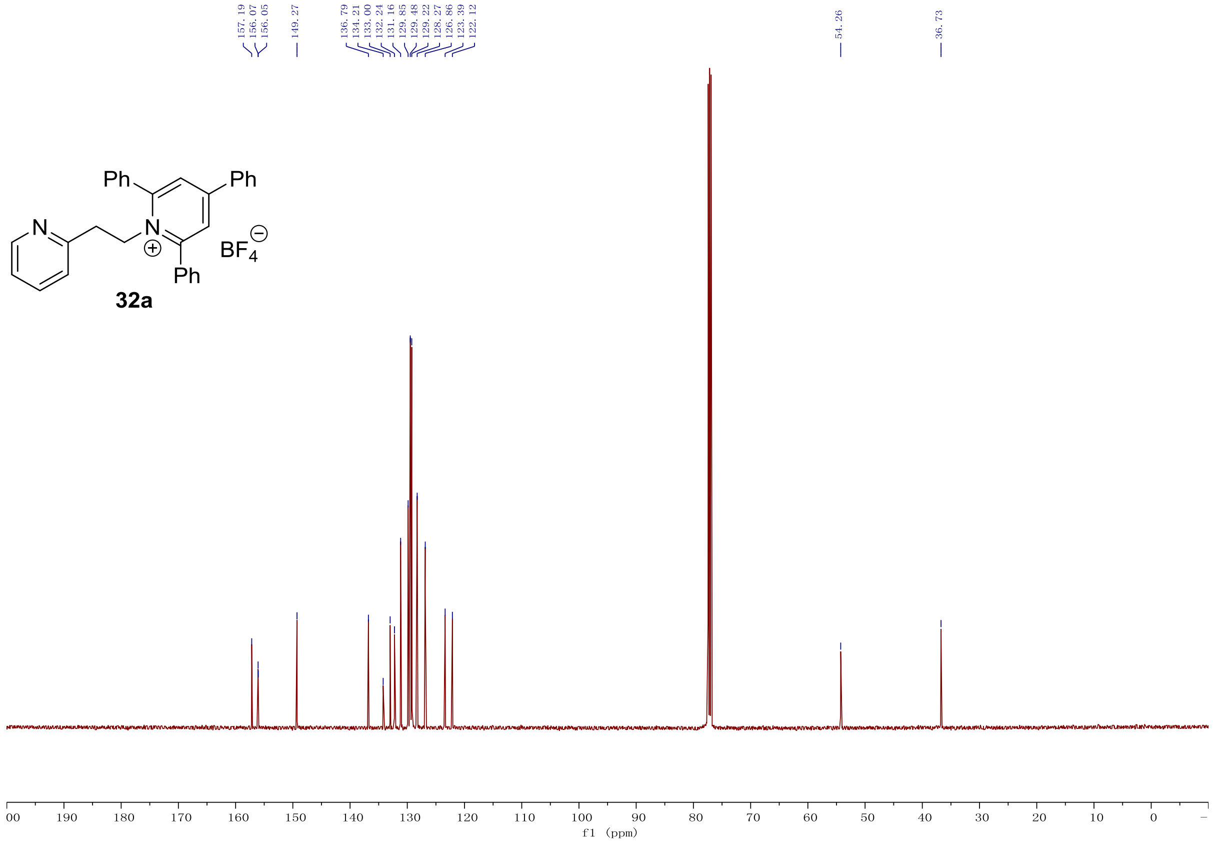

157.19
156.07
156.05
149.27
136.79
134.21
133.00
132.24
131.16
129.85
129.48
129.22
128.27
126.86
123.39
122.12
54.26
36.73
Ph
Ph
N
N
BF4
Ph
f1 (ppm)

32a

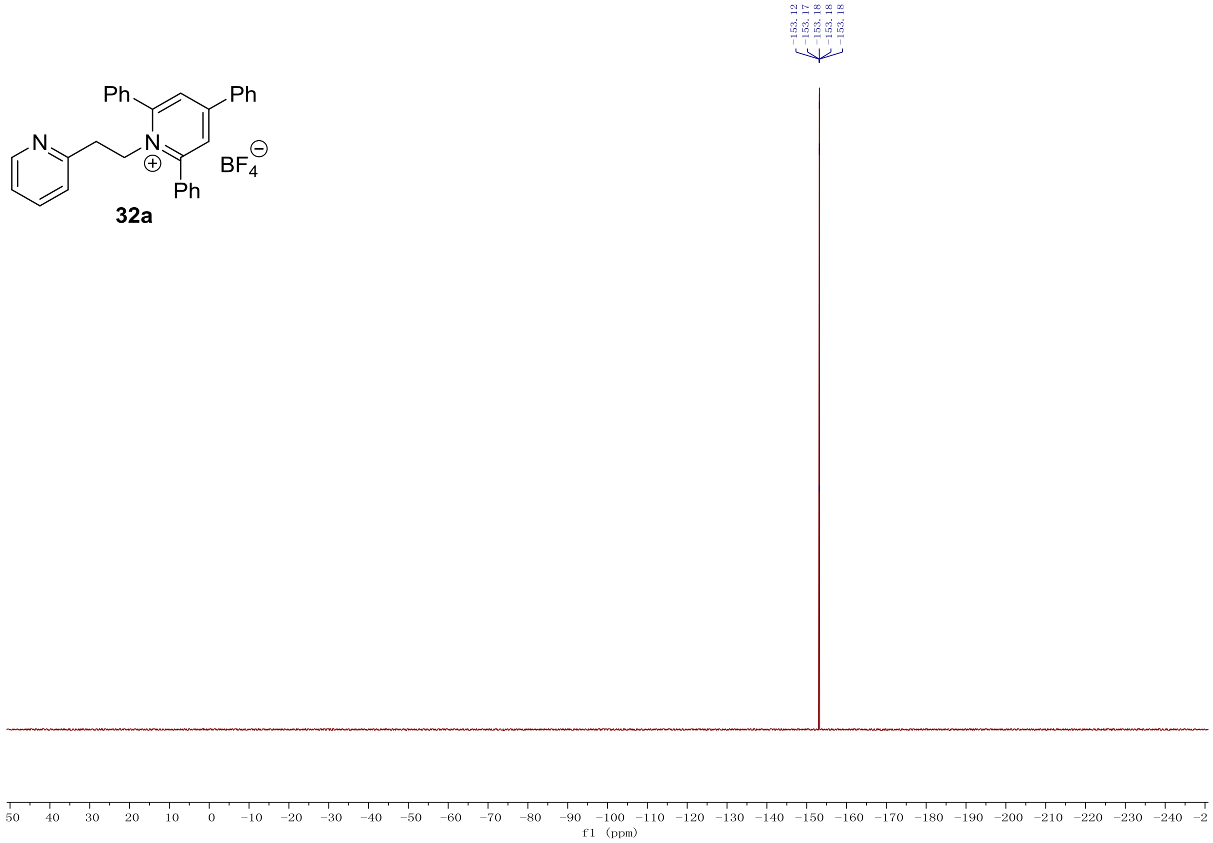

-153.12
-153.17
-153.18
-153.18
-153.18
Ph
Ph
N
N
BF4
Ph
f1 (ppm)

32a

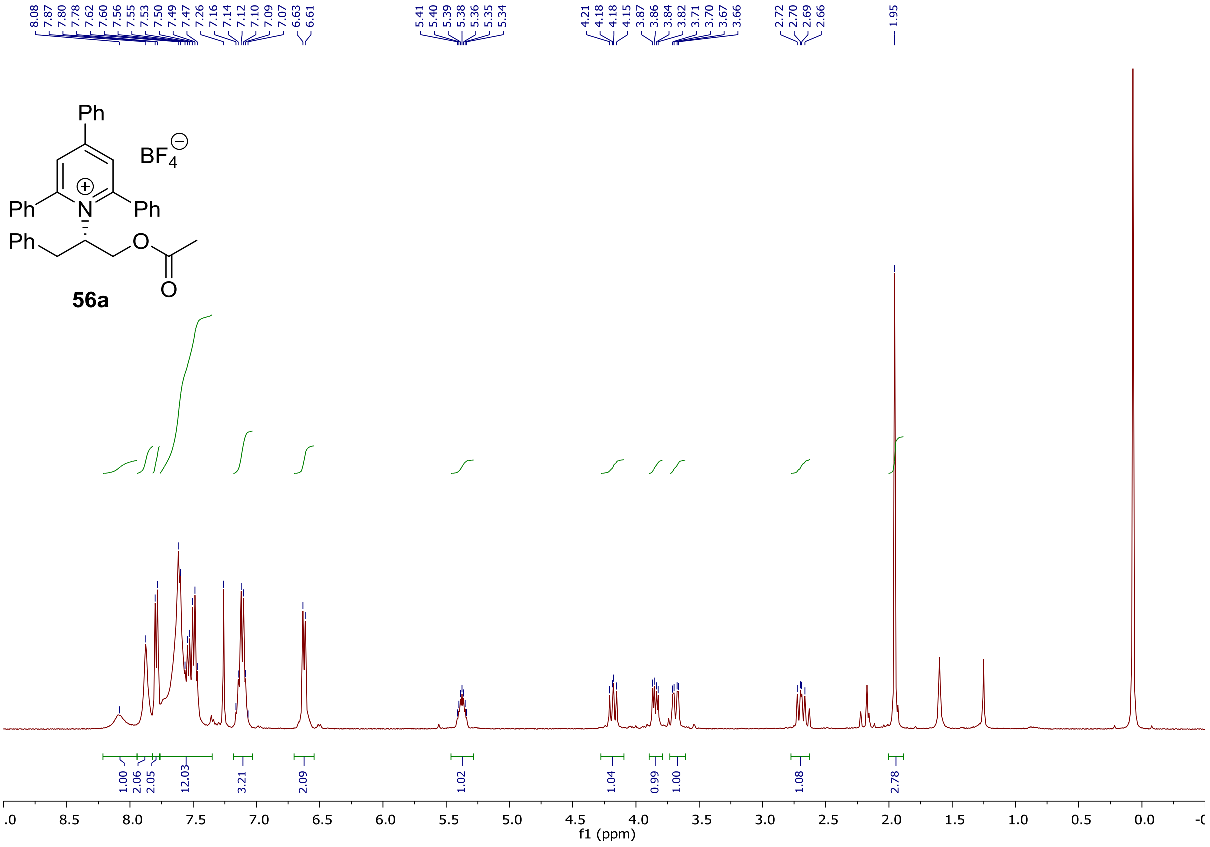
8.08
7.87
7.80
7.78
7.62
7.60
7.56
7.55
7.53
7.50
7.49
7.47
7.26
7.16
7.14
7.12
7.10
7.09
7.07
6.63
6.61
5.41
5.40
5.39
5.38
5.36
5.35
5.34
4.21
4.18
4.18
4.15
3.87
3.86
3.84
3.82
3.71
3.70
3.67
3.66
2.72
2.70
2.69
2.66
1.95
Ph
BF4
Ph
N
Ph
Ph
O
O
56a
1.00
2.06
2.05
12.03
3.21
2.09
1.02
1.04
0.99
1.00
1.08
2.78
f1 (ppm)

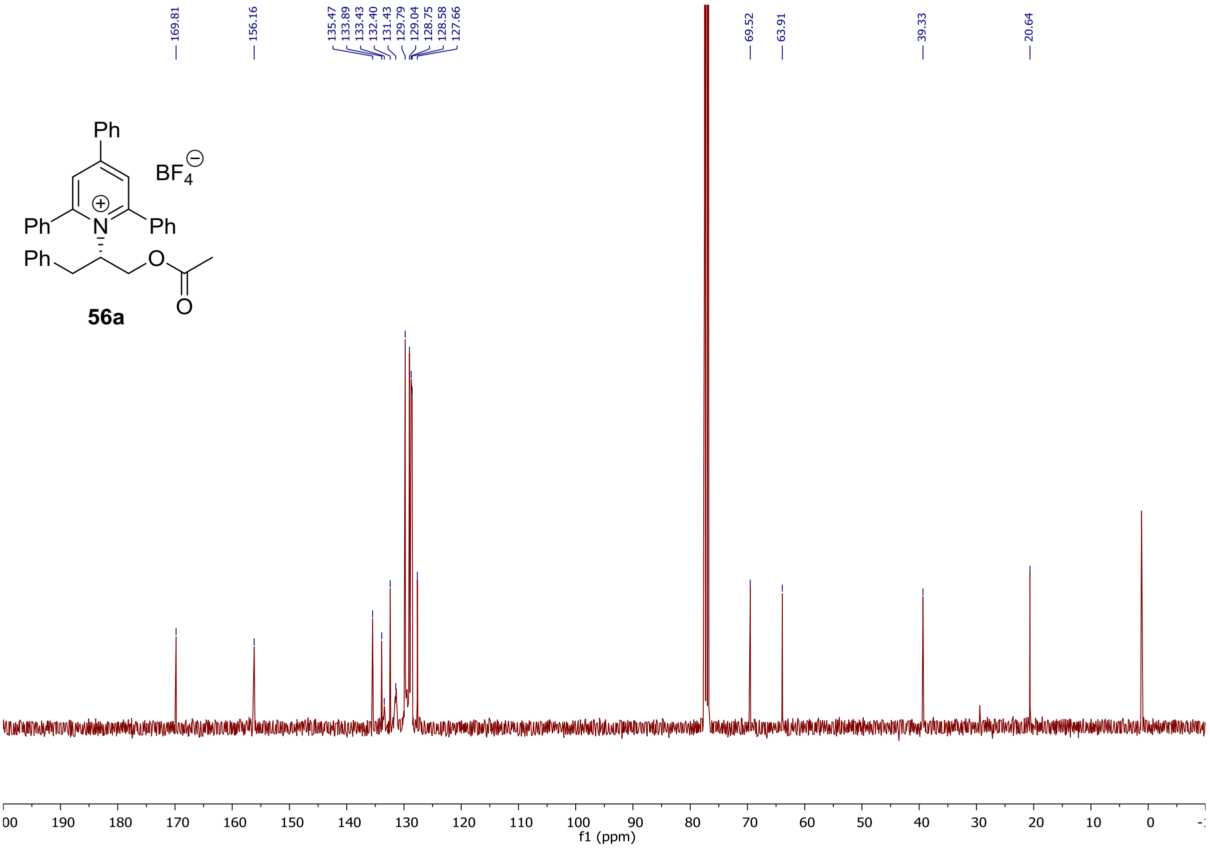
169.81
156.16
135.47
133.89
133.43
132.40
131.43
129.79
129.04
128.75
128.58
127.66
69.52
63.91
39.33
20.64
Ph
BF4
Ph
N
Ph
Ph
O
O
56a
f1 (ppm)

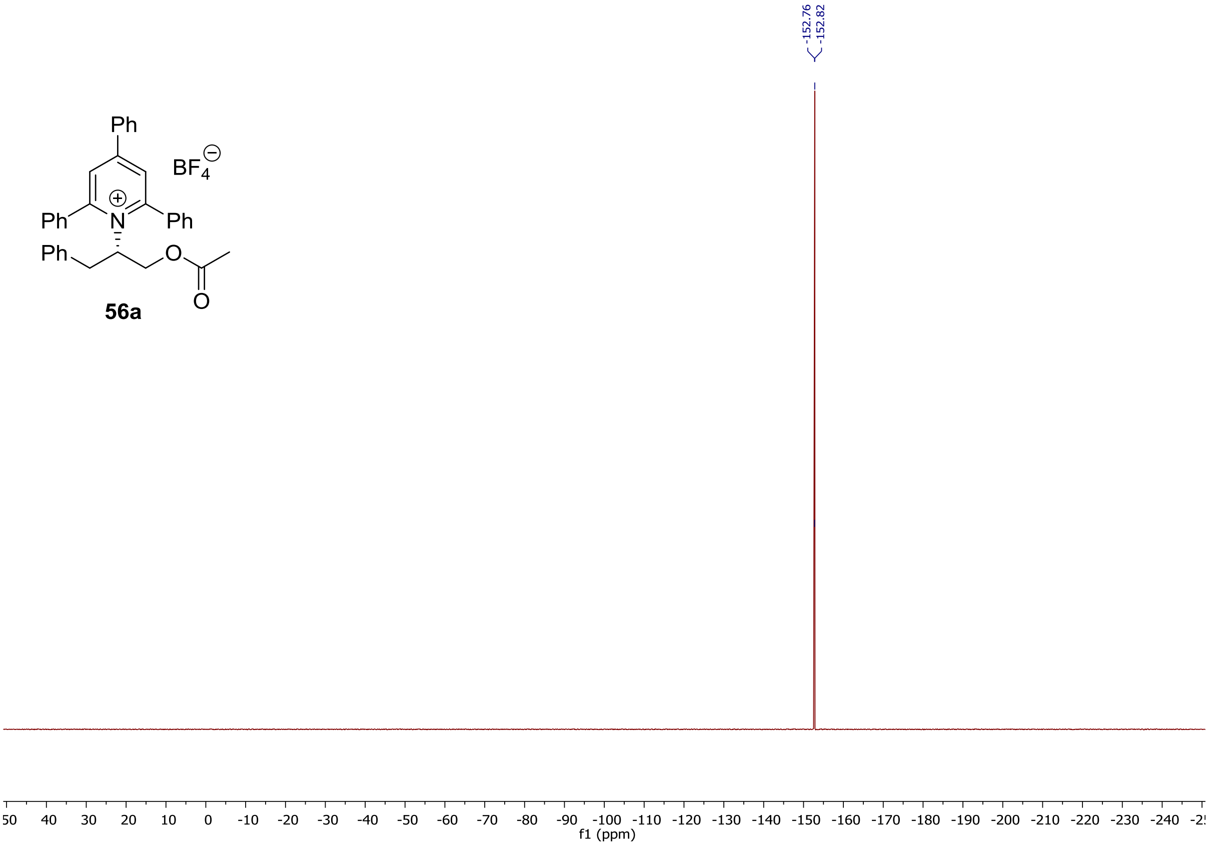
-152.76
-152.82
f1 (ppm)

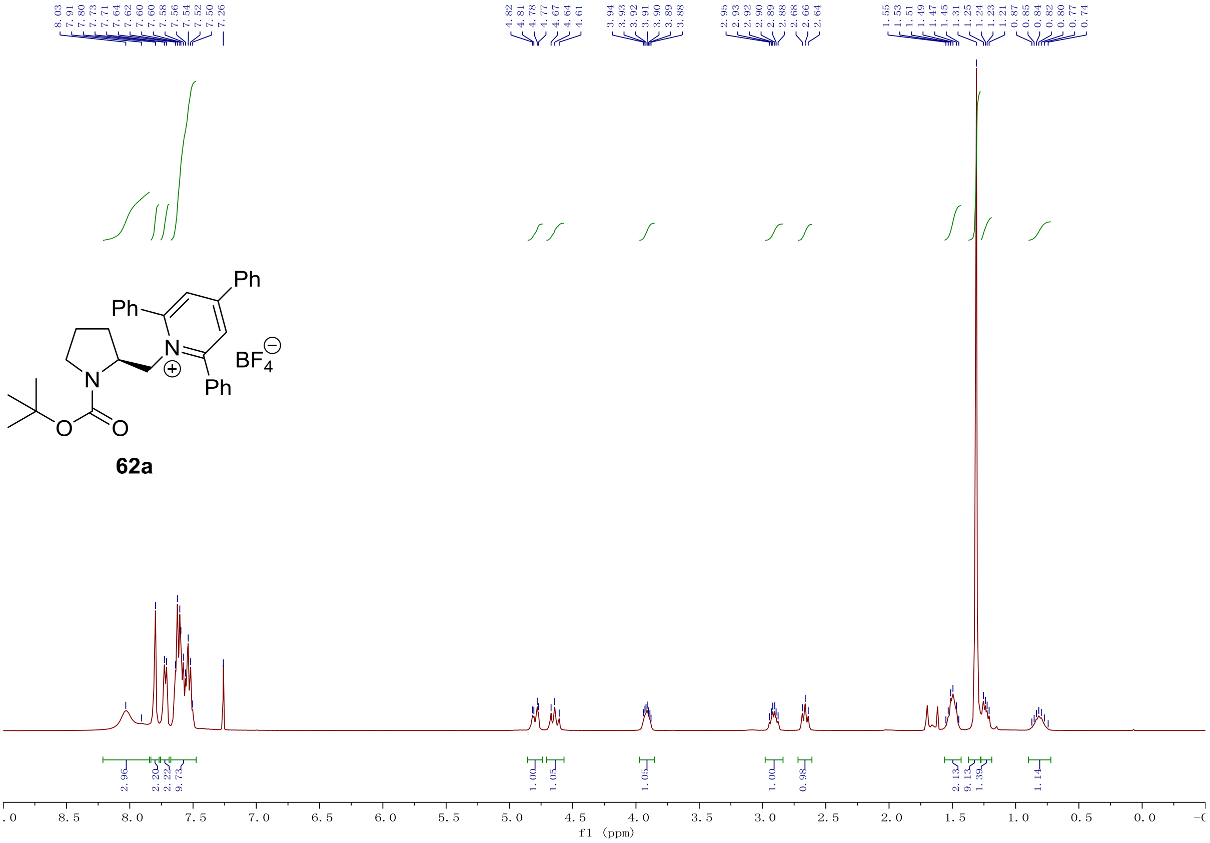
f1 (ppm)

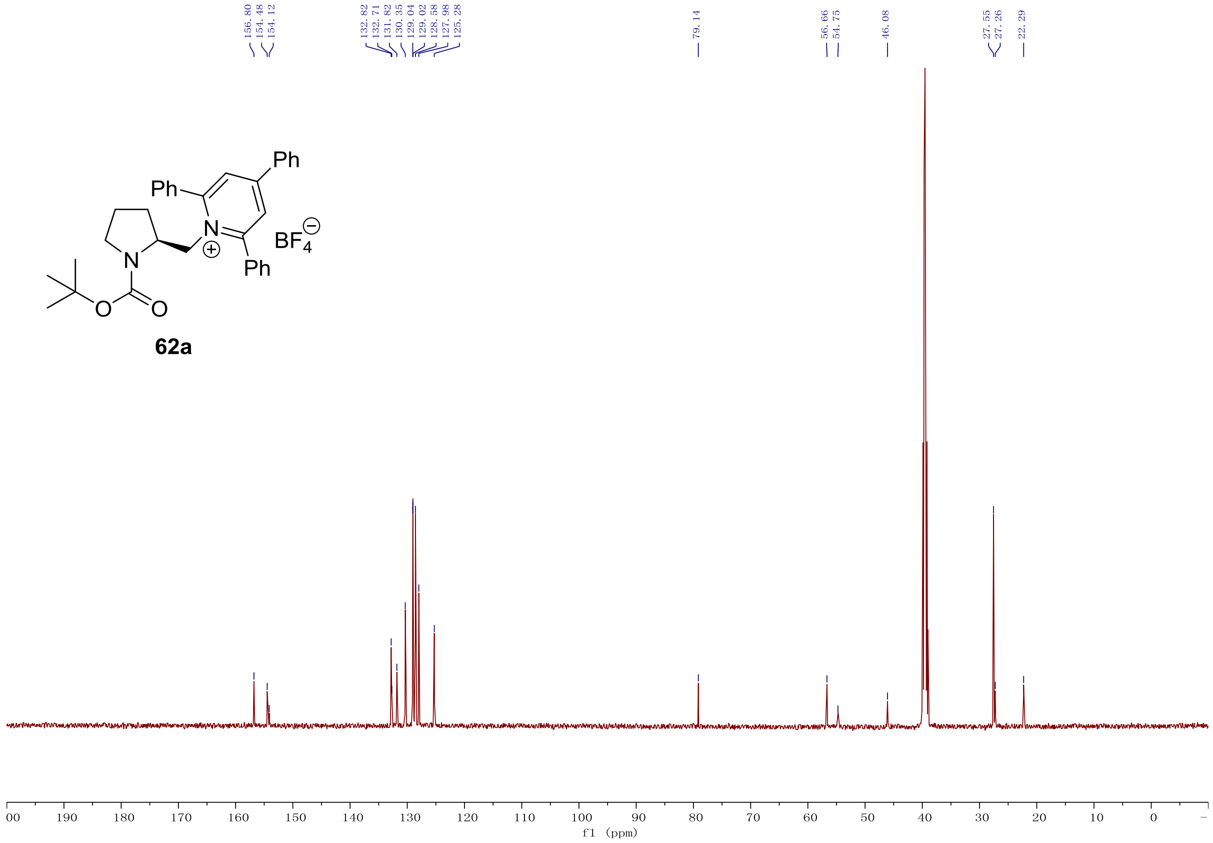
156.80
154.48
154.12
132.82
132.71
131.82
130.35
129.04
129.02
128.58
127.98
125.28
79.14
56.66
54.75
46.08
27.55
27.26
22.29
Ph
Ph
N
N
BF4
Ph
O
O
62a
f1 (ppm)

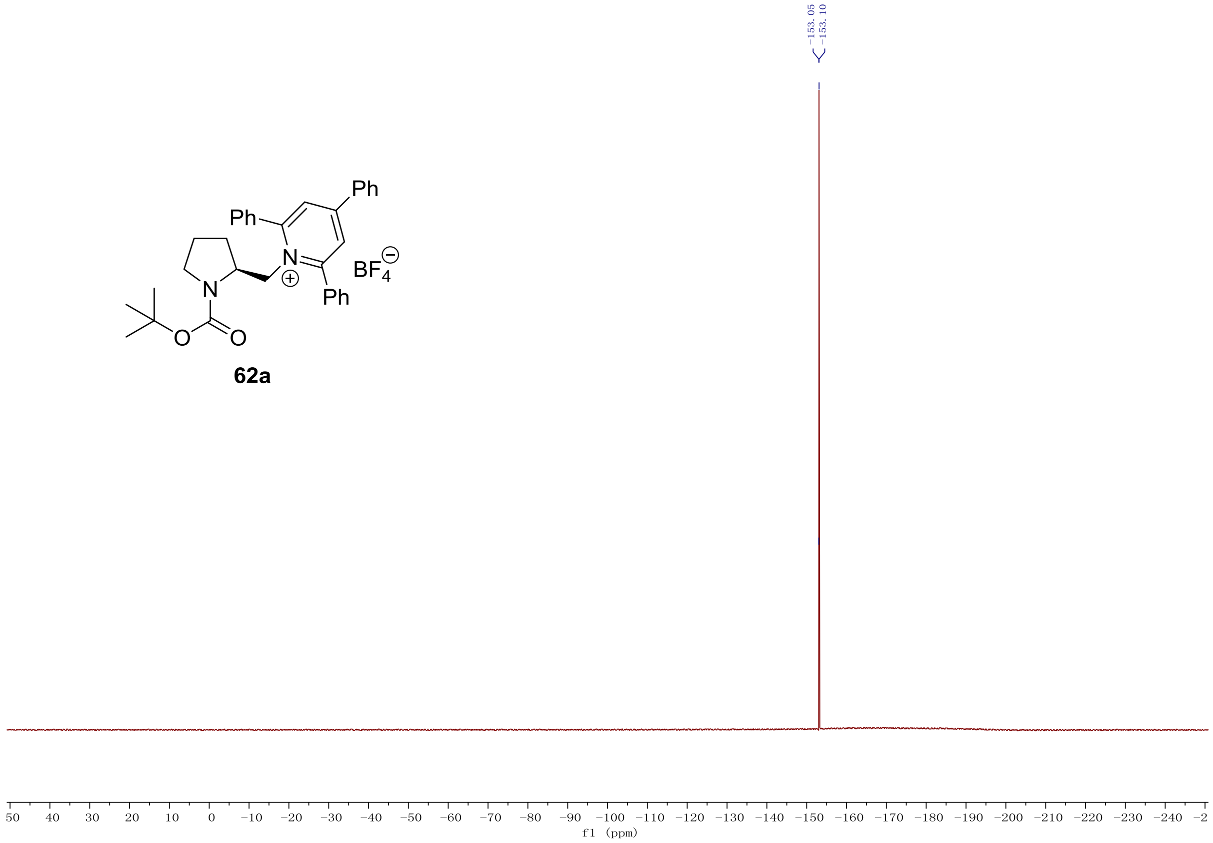
-153.05
-153.10
Ph
Ph
N
N
BF4
Ph
O
O
62a
f1 (ppm)

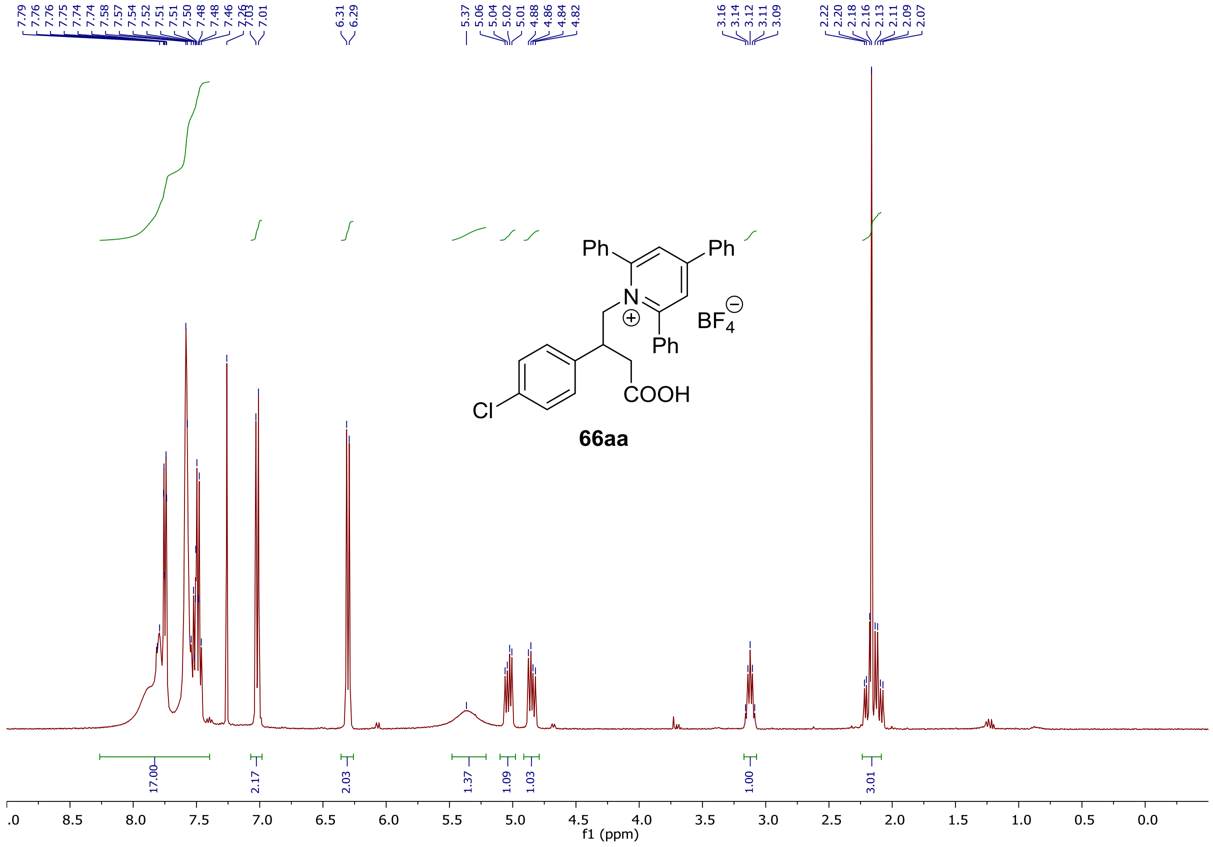
7.79 7.76 7.76 7.75 7.74 7.74 7.58 7.57 7.54 7.52 7.51 7.51 7.50 7.48 7.48 7.46 7.03 7.01
6.31 6.29
5.37 5.06 5.04 5.02 5.01 4.88 4.86 4.84 4.82
3.16 3.14 3.12 3.11 3.09
2.22 2.20 2.18 2.16 2.13 2.11 2.09 2.07
Ph
Ph
N
BF4
Ph
Cl
COOH
66aa
17.00
2.17
2.03
1.37
1.09
1.03
1.00
3.01
f1 (ppm)

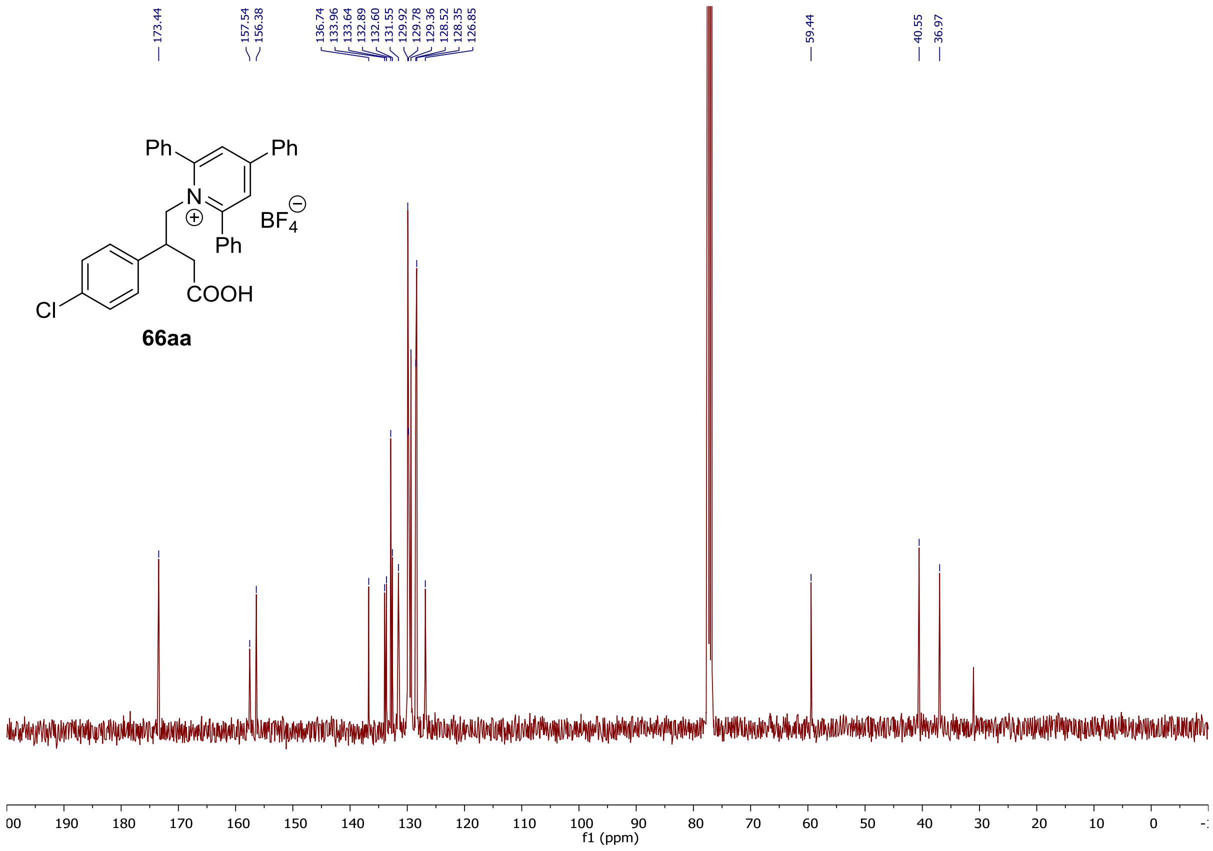
173.44
157.54 156.38
136.74 133.96 133.64 132.89 132.60 131.55 129.92 129.78 129.36 128.52 128.35 126.85
59.44
40.55
36.97
Ph
Ph
N
BF4
Ph
Cl
COOH
66aa
f1 (ppm)

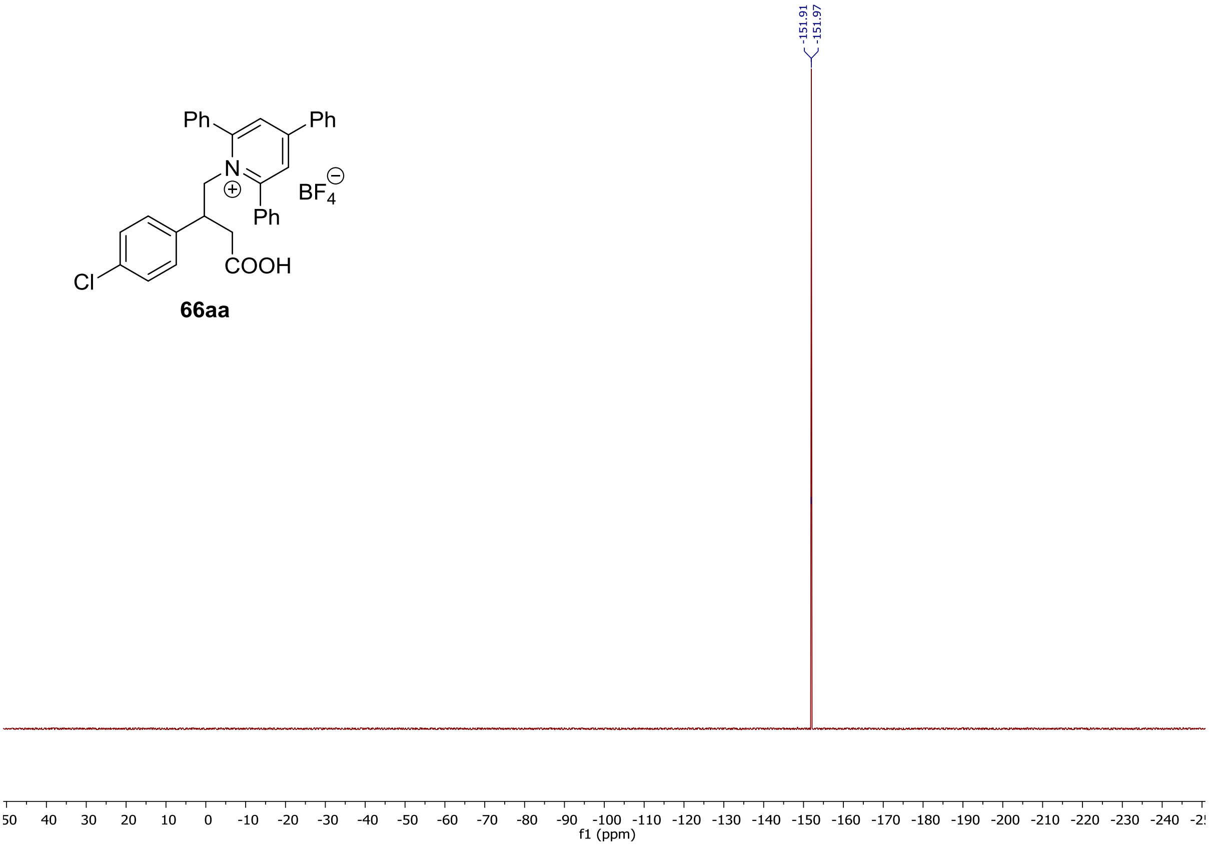
-151.91
-151.97
Ph
Ph
N
BF4
Ph
COOH
Cl
66aa
f1 (ppm)

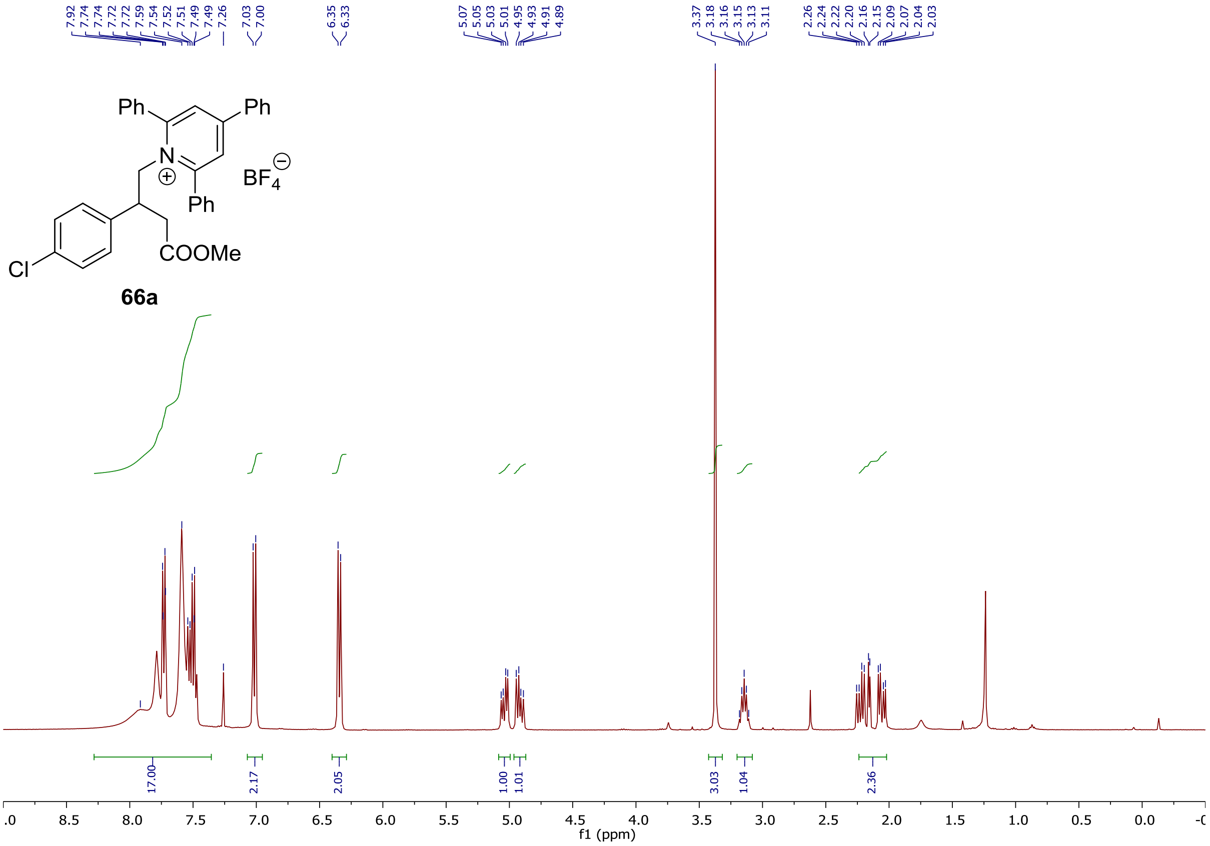
7.92 7.74 7.74 7.72 7.72 7.59 7.54 7.52 7.51 7.49 7.49 7.26 7.03 7.00
6.35 6.33
5.07 5.05 5.03 5.01 4.95 4.93 4.91 4.89
3.37 3.18 3.16 3.15 3.13 3.11
2.26 2.24 2.22 2.20 2.16 2.15 2.09 2.07 2.04 2.03
Ph
Ph
N
BF4
Ph
COOMe
Cl
66a
17.00
2.17
2.05
1.00
1.01
3.03
1.04
2.36
f1 (ppm)

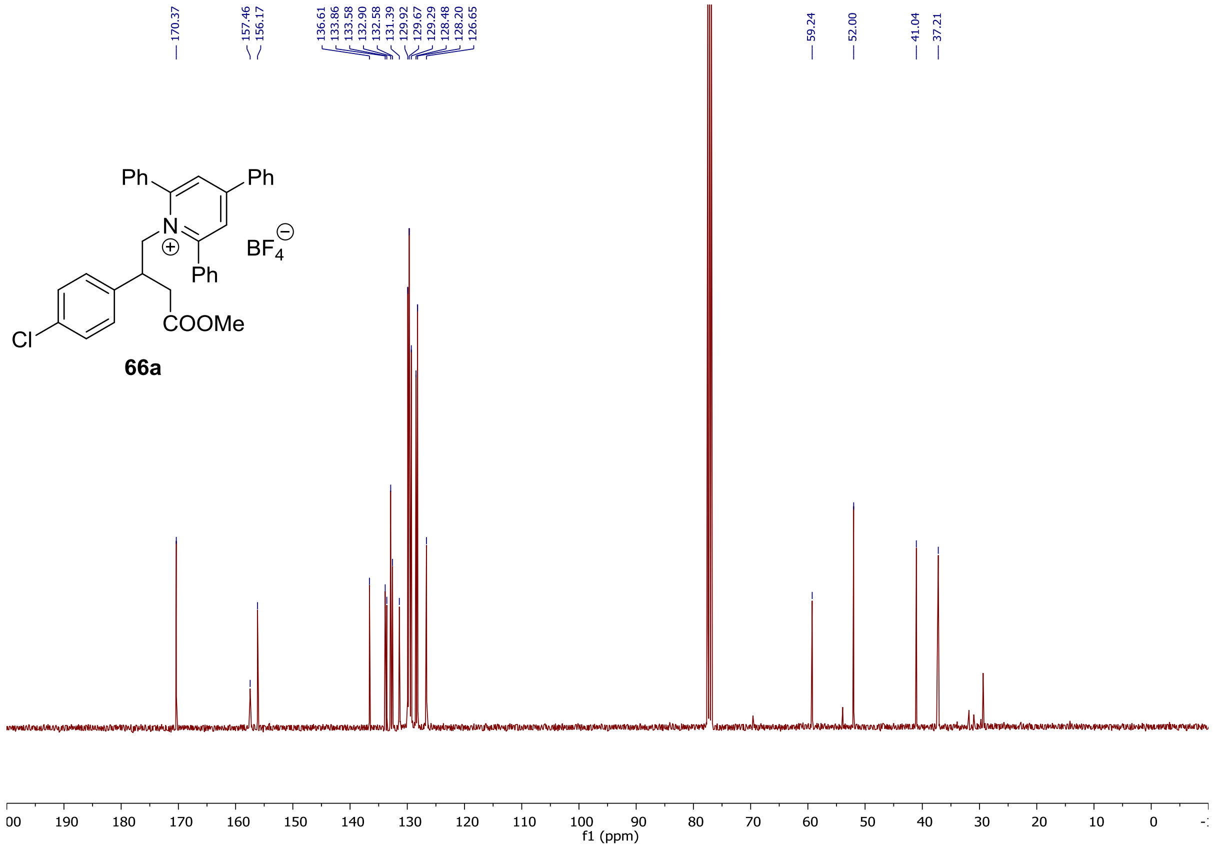
170.37
157.46
156.17
136.61
133.86
133.58
132.90
132.58
131.39
129.92
129.67
129.29
128.48
128.20
126.65
59.24
52.00
41.04
37.21
Ph
Ph
N
BF4
Ph
Cl
COOMe
66a
f1 (ppm)

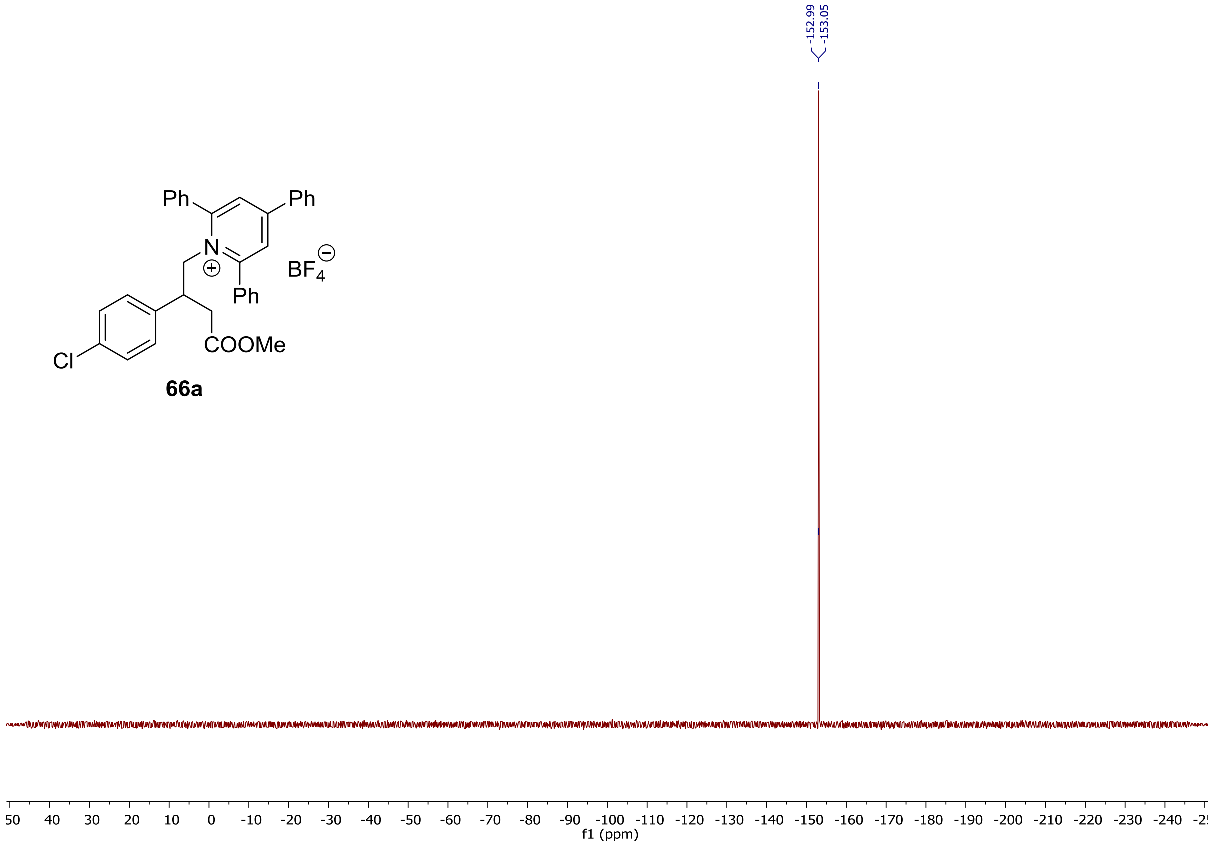
-152.99
-153.05
Ph
Ph
N
BF4
Ph
Cl
COOMe
66a
f1 (ppm)

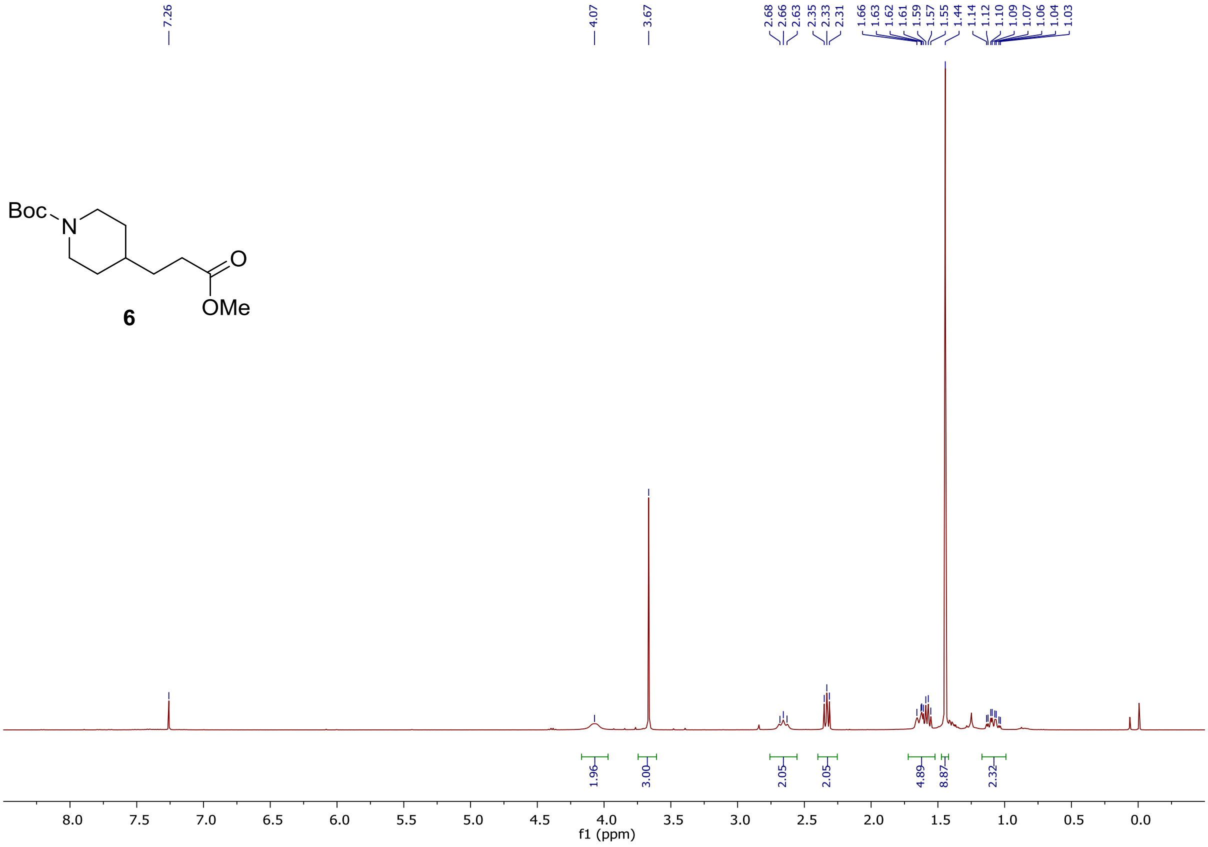
7.26
4.07
3.67
2.68
2.66
2.63
2.35
2.33
2.31
1.66
1.63
1.62
1.61
1.59
1.57
1.55
1.44
1.14
1.12
1.10
1.09
1.07
1.06
1.04
1.03
Boc
N
O
OMe
6
1.96
3.00
2.05
2.05
4.89
8.87
2.32
8.0 7.5 7.0 6.5 6.0 5.5 5.0 4.5 4.0 3.5 3.0 2.5 2.0 1.5 1.0 0.5 0.0
f1 (ppm)

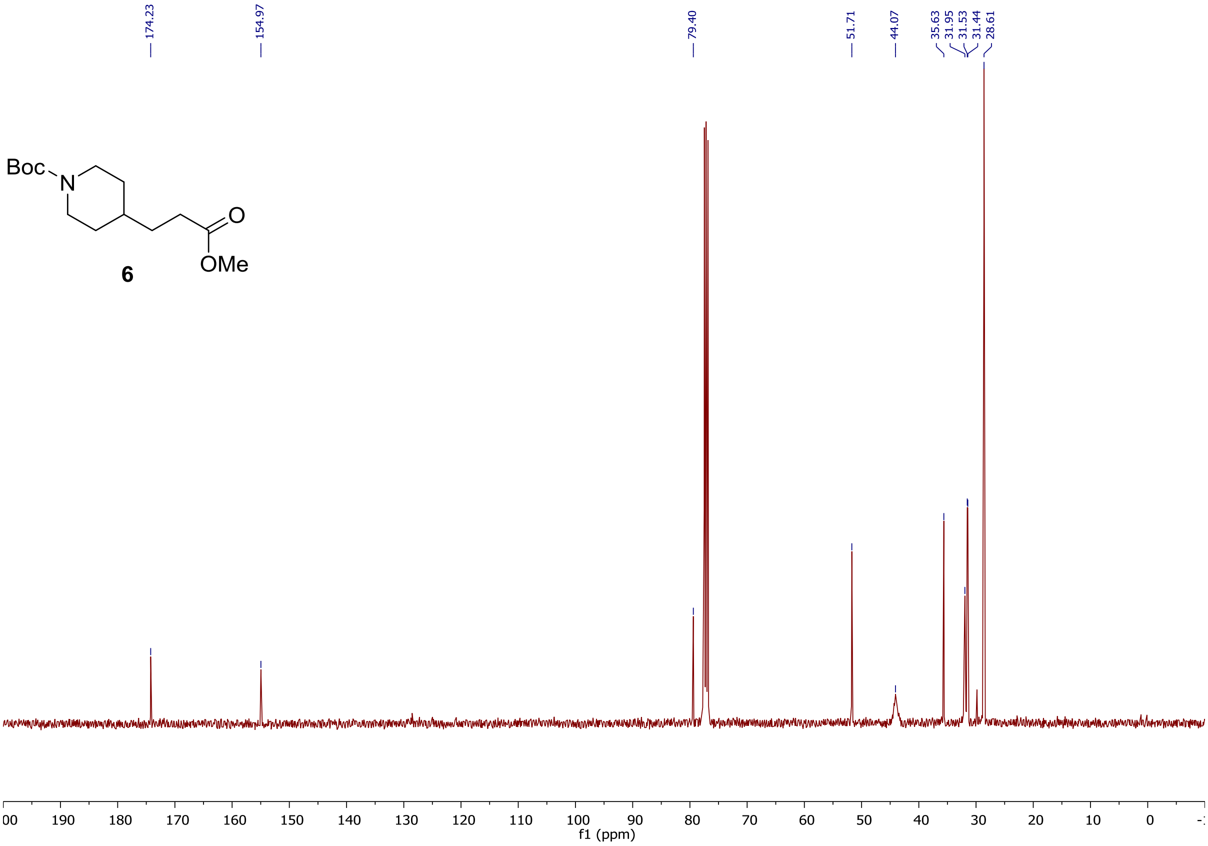
174.23
154.97
79.40
51.71
44.07
35.63
31.95
31.53
31.44
28.61
Boc
N
O
OMe
6
190 180 170 160 150 140 130 120 110 100 90 80 70 60 50 40 30 20 10 0
f1 (ppm)

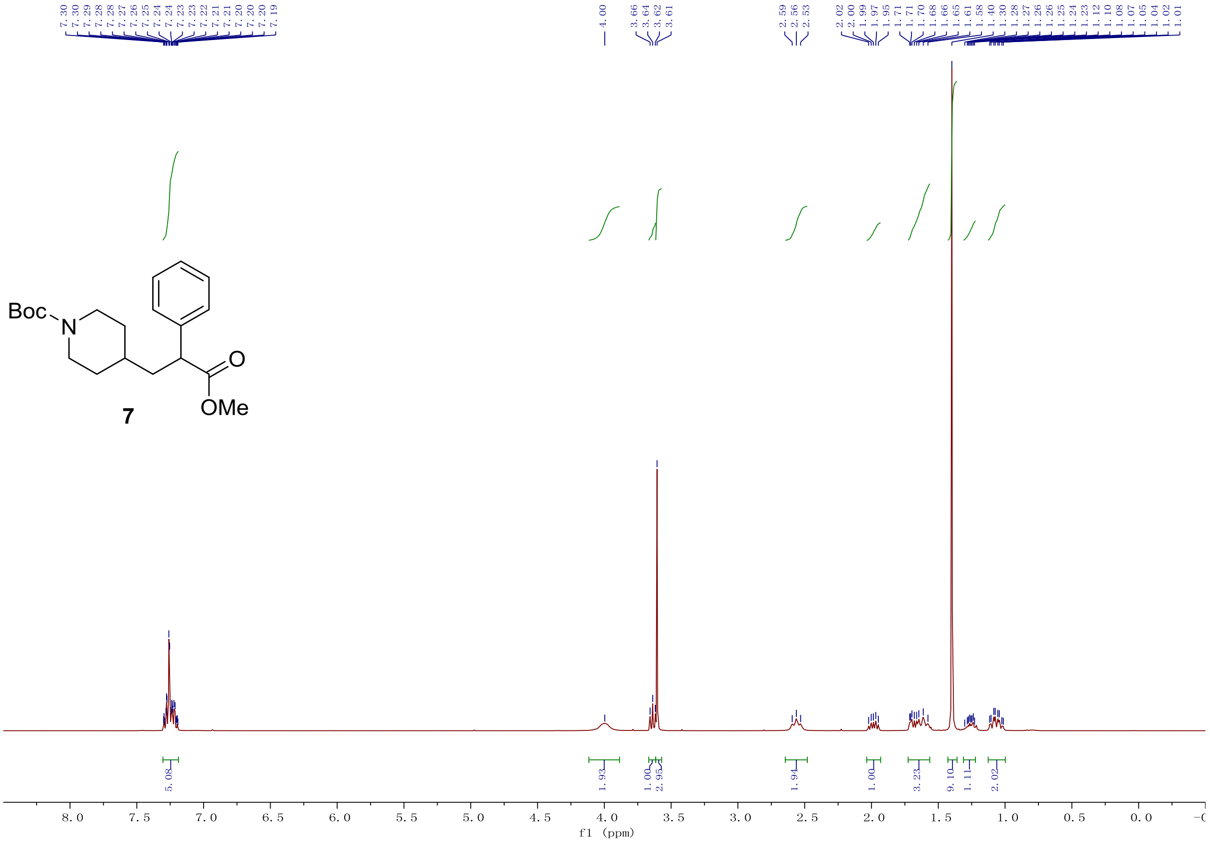
Boc
N
O
OMe
7
f1 (ppm)

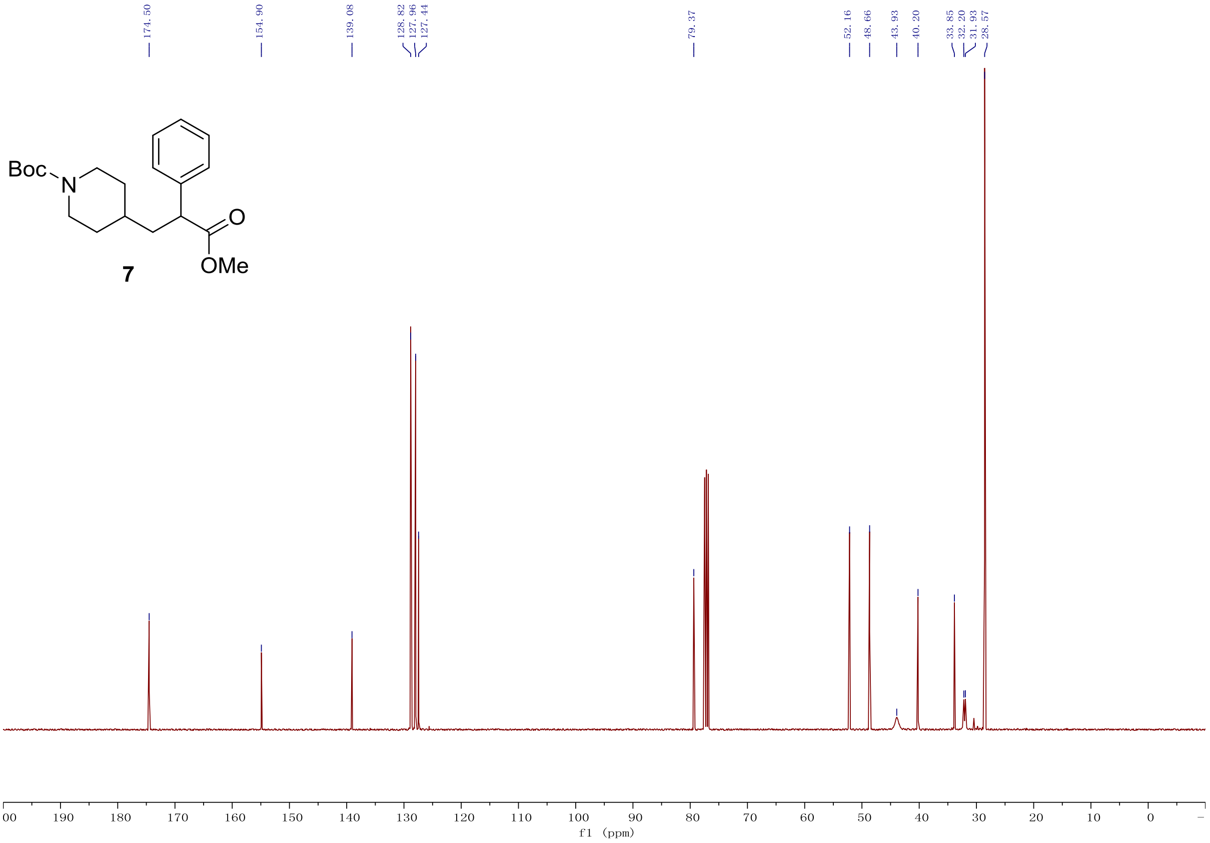
Boc
N
O
OMe
7
174.50
154.90
139.08
128.82
127.96
127.44
79.37
52.16
48.66
43.93
40.20
33.85
32.20
31.93
28.57
f1 (ppm)

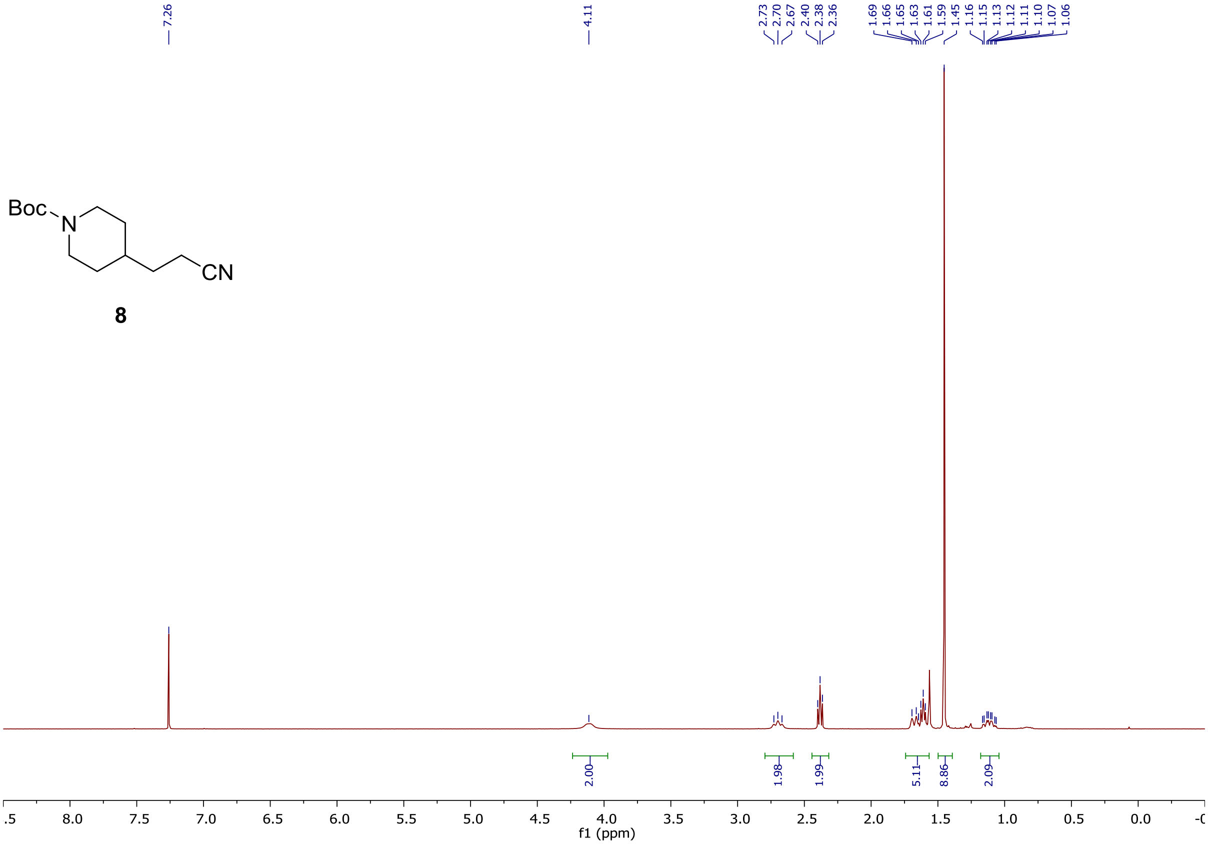
7.26
4.11
2.73
2.70
2.67
2.40
2.38
2.36
1.69
1.66
1.65
1.63
1.61
1.59
1.45
1.16
1.15
1.13
1.12
1.11
1.10
1.07
1.06
Boc
N
CN
8
2.00
1.98
1.99
5.11
8.86
2.09
f1 (ppm)

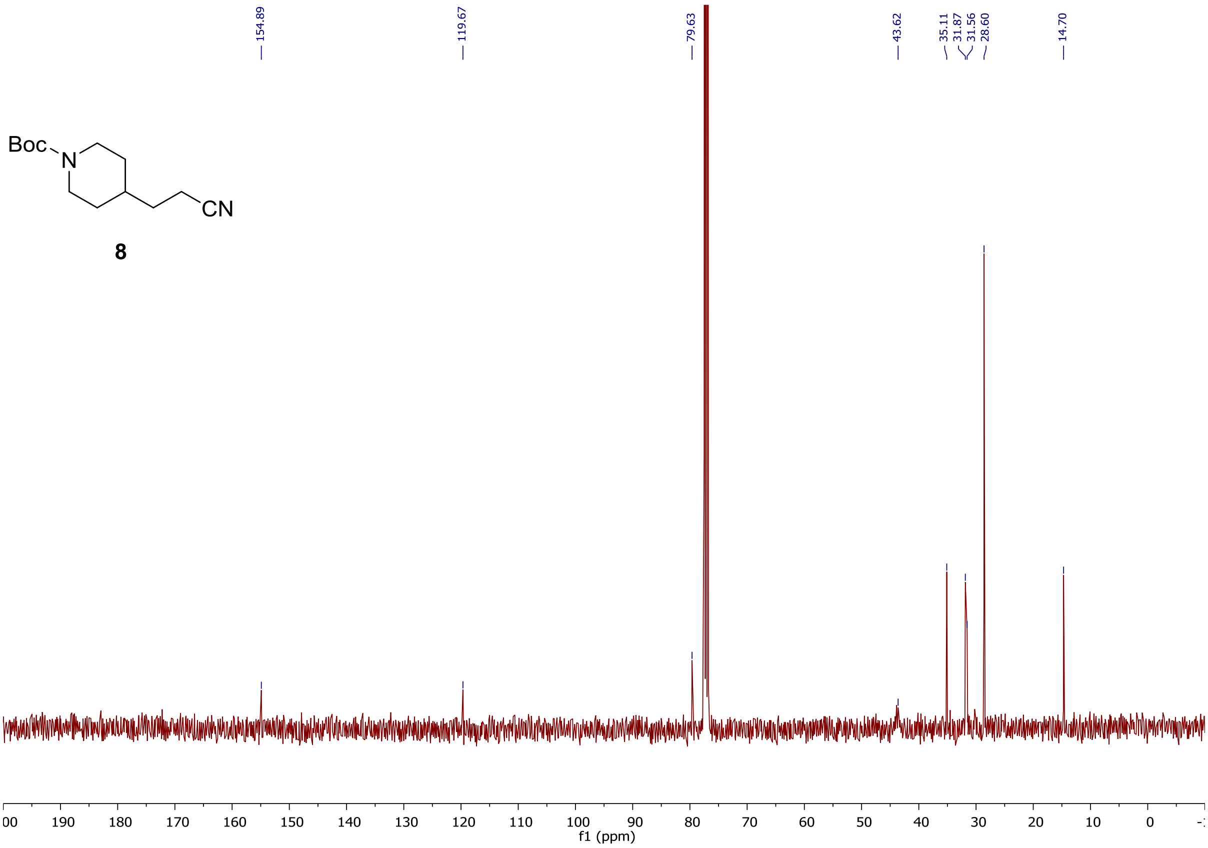
154.89
119.67
79.63
43.62
35.11
31.87
31.56
28.60
14.70
Boc
N
CN
8
f1 (ppm)

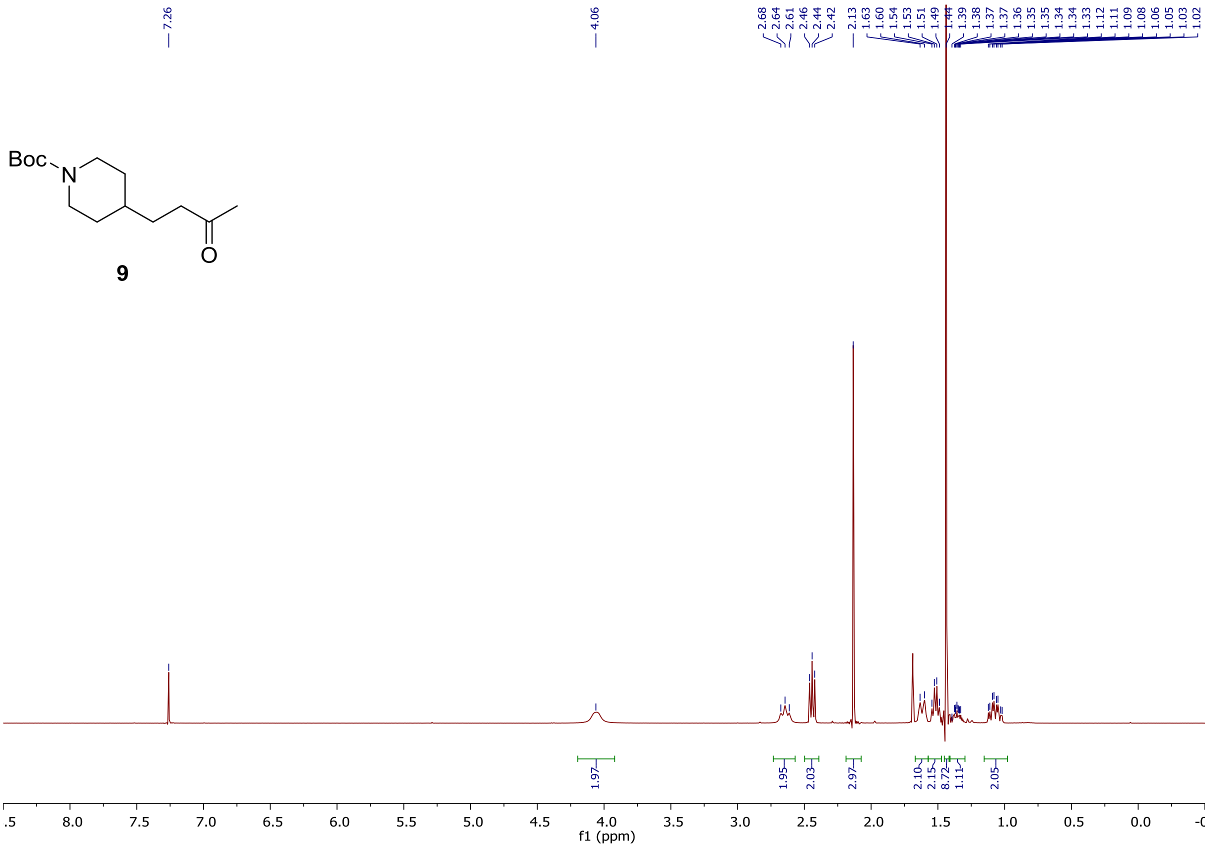
7.26
4.06
2.68 2.64 2.61 2.46 2.44 2.42 2.13 1.63 1.60 1.54 1.53 1.51 1.49 1.44 1.39 1.38 1.37 1.37 1.36 1.35 1.35 1.34 1.34 1.33 1.12 1.11 1.09 1.08 1.06 1.05 1.03 1.02
Boc
N
O
9
1.97
1.95
2.03
2.97
2.10
2.15
8.72
1.11
2.05
f1 (ppm)

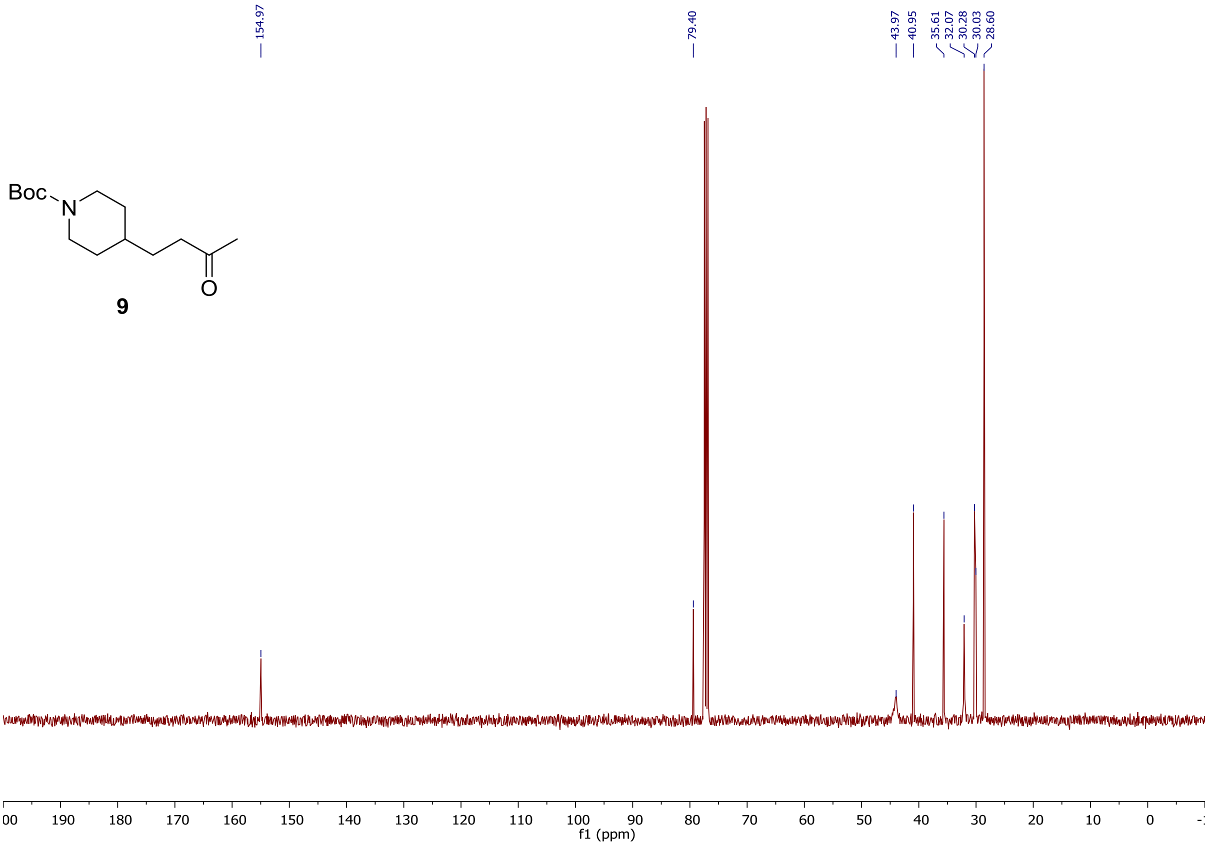
154.97
79.40
43.97
40.95
35.61
32.07
30.28
30.03
28.60
Boc
N
O
9
f1 (ppm)

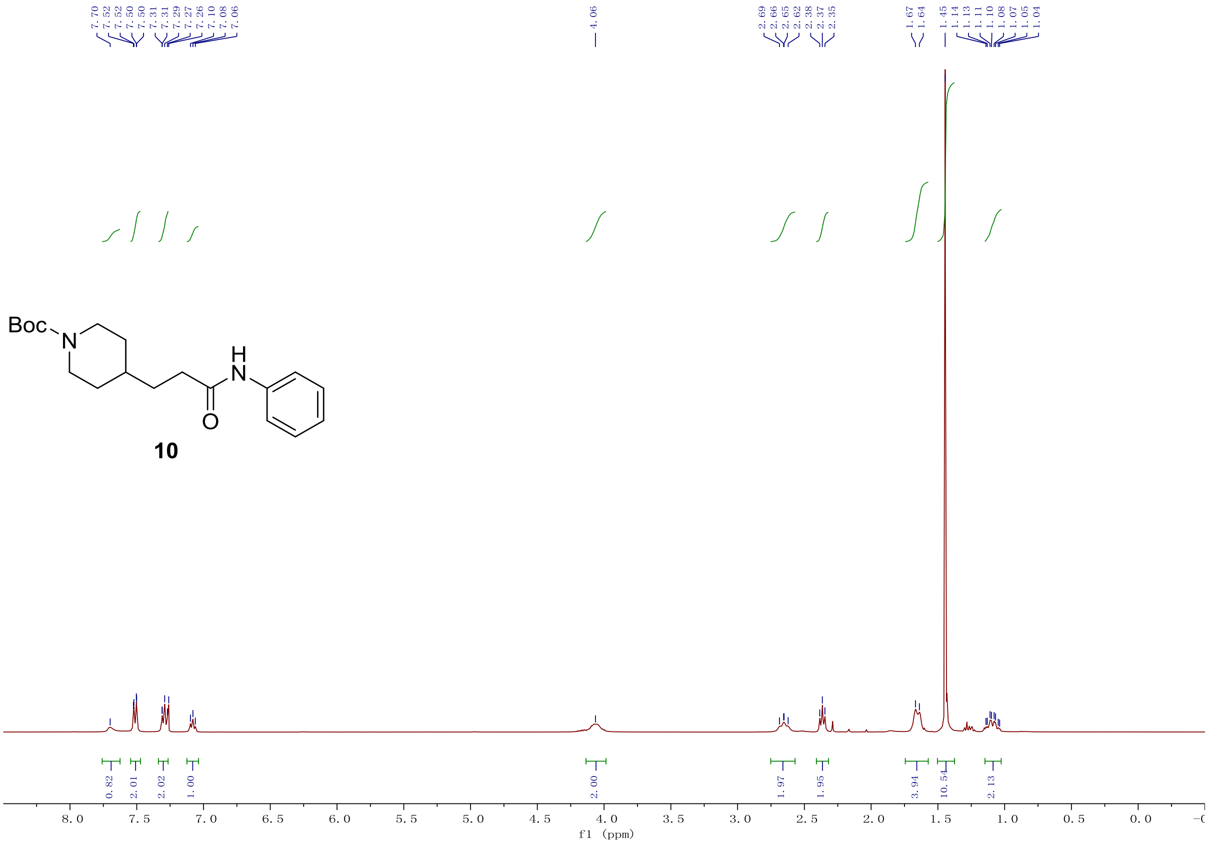

7.70
7.52
7.52
7.50
7.50
7.31
7.31
7.29
7.27
7.26
7.10
7.08
7.06
4.06
2.69
2.66
2.65
2.62
2.38
2.37
2.35
1.67
1.64
1.45
1.14
1.13
1.11
1.10
1.08
1.07
1.05
1.04
Boc
N
H
N
O
10
0.82
2.01
2.02
1.00
2.00
1.97
1.95
3.94
10.54
2.13
f1 (ppm)

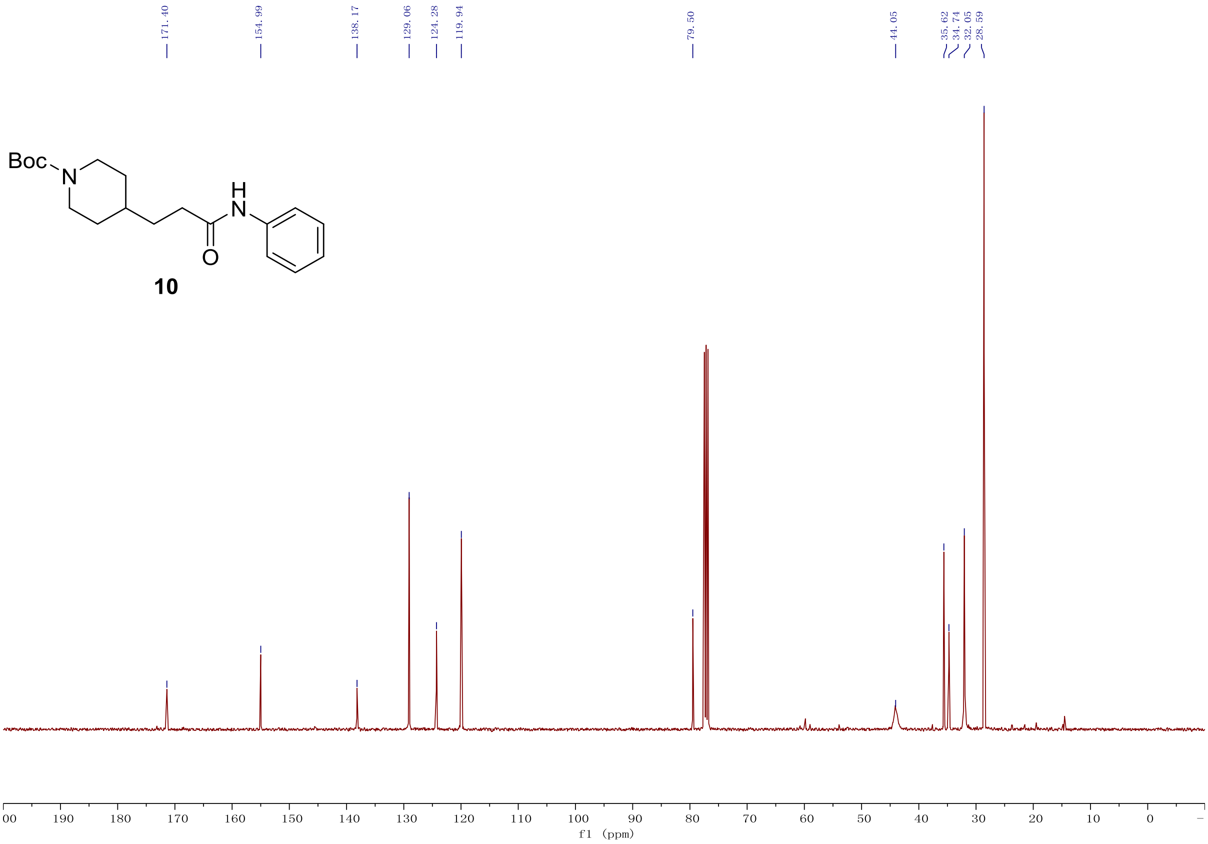

171.40
154.99
138.17
129.06
124.28
119.94
79.50
44.05
35.62
34.74
32.05
28.59
Boc
N
H
N
O
10
f1 (ppm)

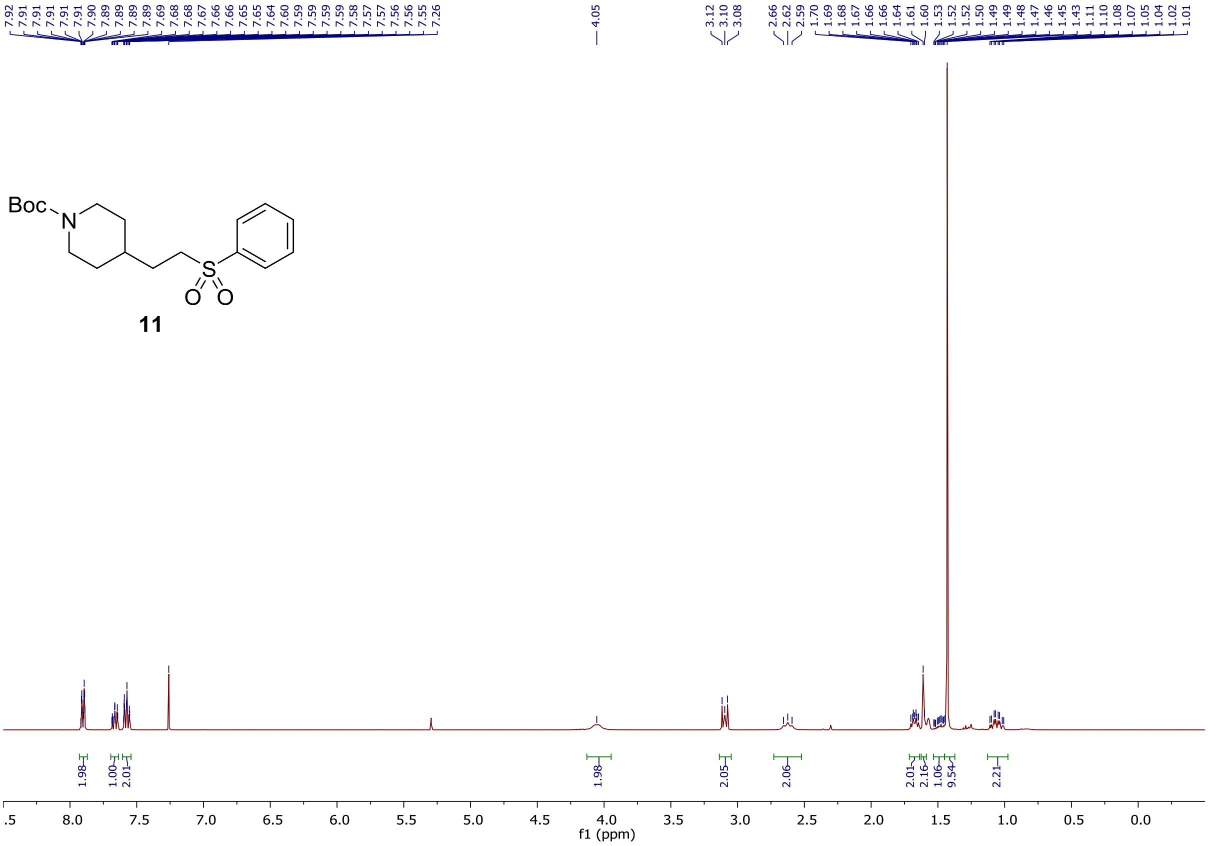

7.92 7.91 7.91 7.91 7.91 7.90 7.89 7.89 7.89 7.89 7.69 7.68 7.68 7.67 7.66 7.66 7.65 7.65 7.64 7.60 7.59 7.59 7.59 7.59 7.58 7.57 7.57 7.56 7.56 7.55 7.26
4.05
3.12 3.10 3.08
2.66 2.62 2.59
1.70 1.69 1.68 1.67 1.66 1.66 1.64 1.61 1.60 1.53 1.52 1.52 1.50 1.49 1.49 1.48 1.47 1.46 1.45 1.43 1.11 1.10 1.08 1.07 1.05 1.04 1.02 1.01
Boc
N
S
O O
11
1.98 1.00 2.01 1.98 2.05 2.06 2.01 2.16 1.06 9.54 2.21
.5 8.0 7.5 7.0 6.5 6.0 5.5 5.0 4.5 4.0 3.5 3.0 2.5 2.0 1.5 1.0 0.5 0.0
f1 (ppm)

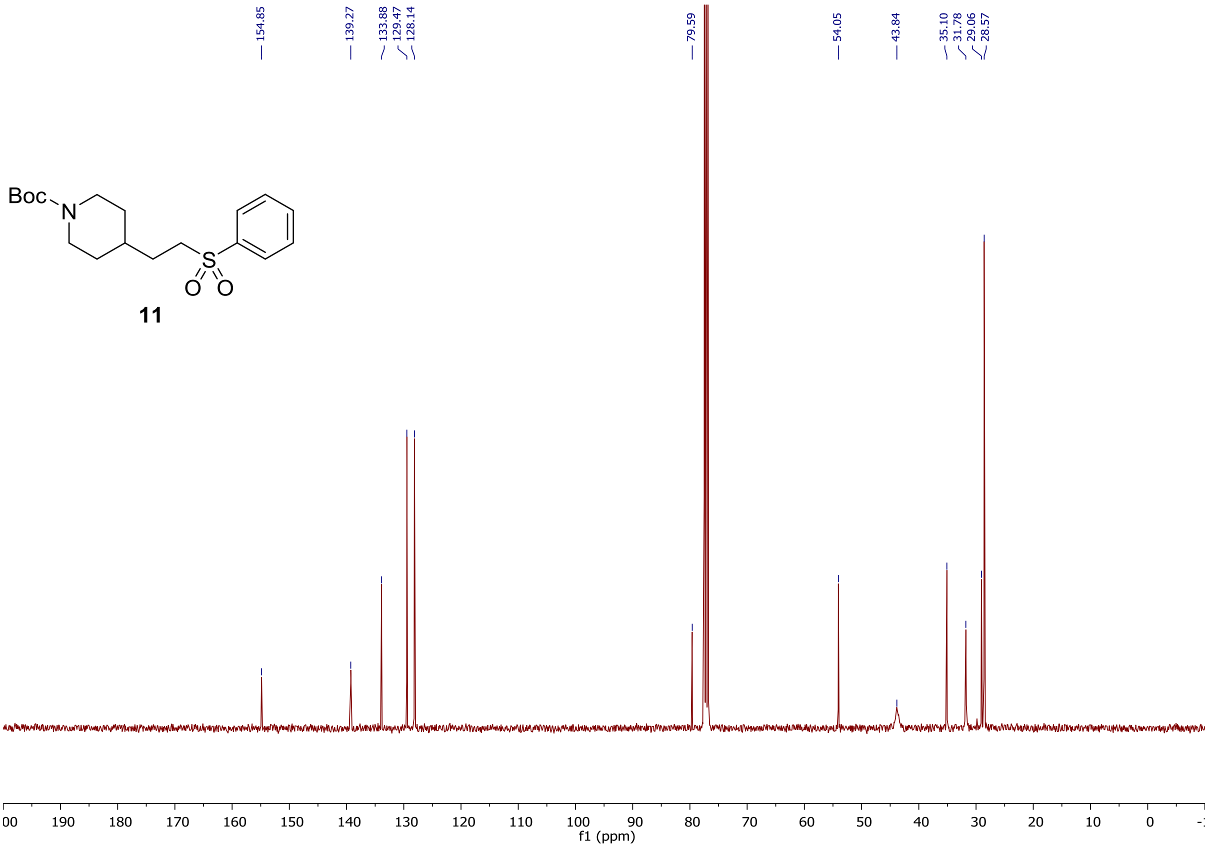

154.85
139.27
133.88 129.47 128.14
79.59
54.05
43.84
35.10 31.78 29.06 28.57
Boc
N
S
O O
11
00 190 180 170 160 150 140 130 120 110 100 90 80 70 60 50 40 30 20 10 0
f1 (ppm)

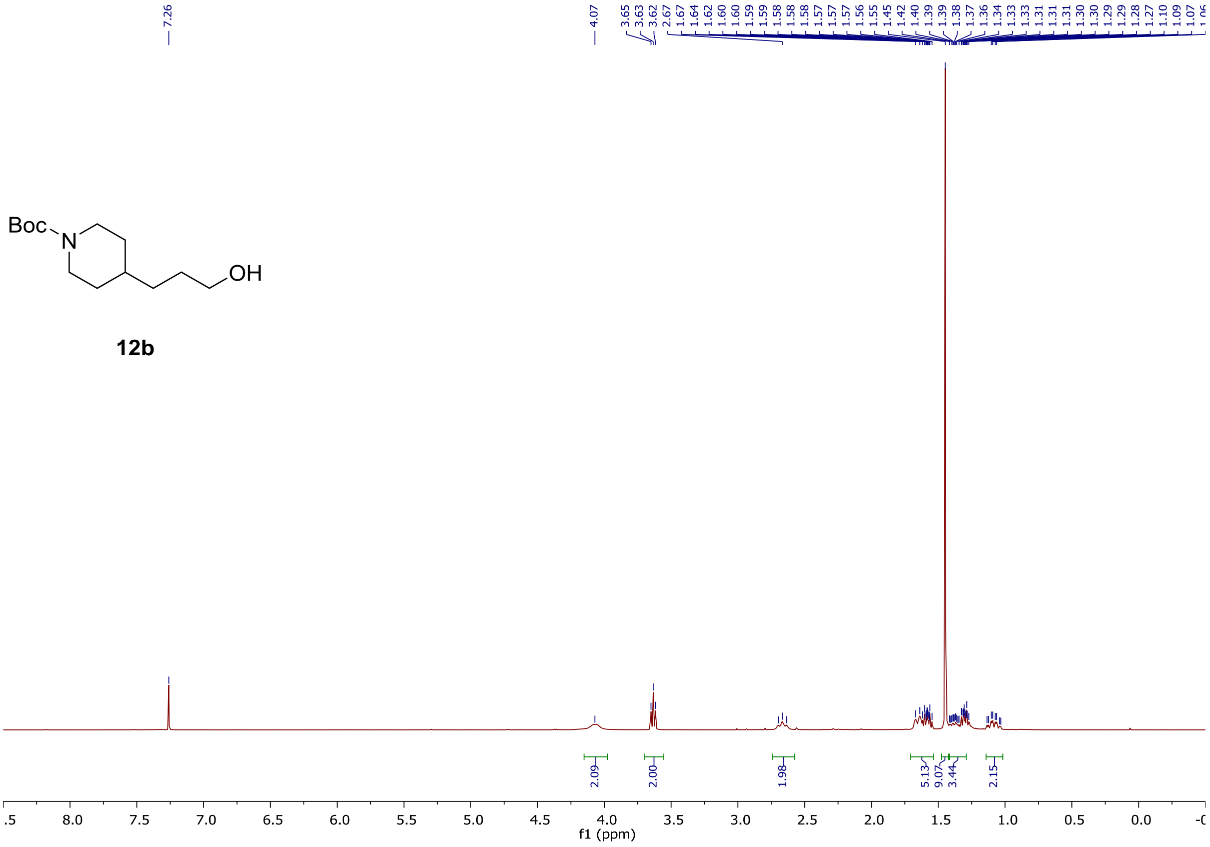
Boc
N
OH
12b
7.26
4.07
3.65
3.63
3.62
2.09
2.00
1.98
5.13
9.07
3.44
2.15
f1 (ppm)

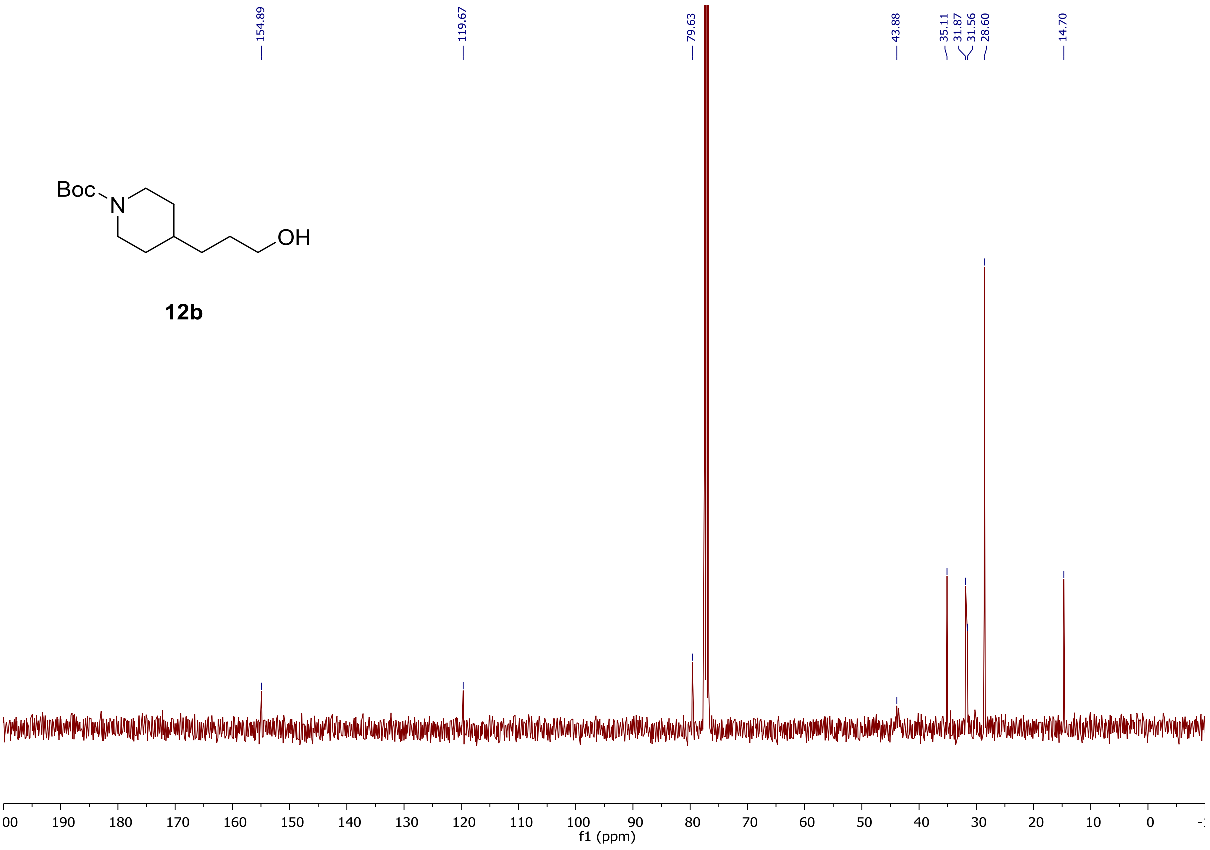
Boc
N
OH
12b
154.89
119.67
79.63
43.88
35.11
31.87
31.56
28.60
14.70
f1 (ppm)

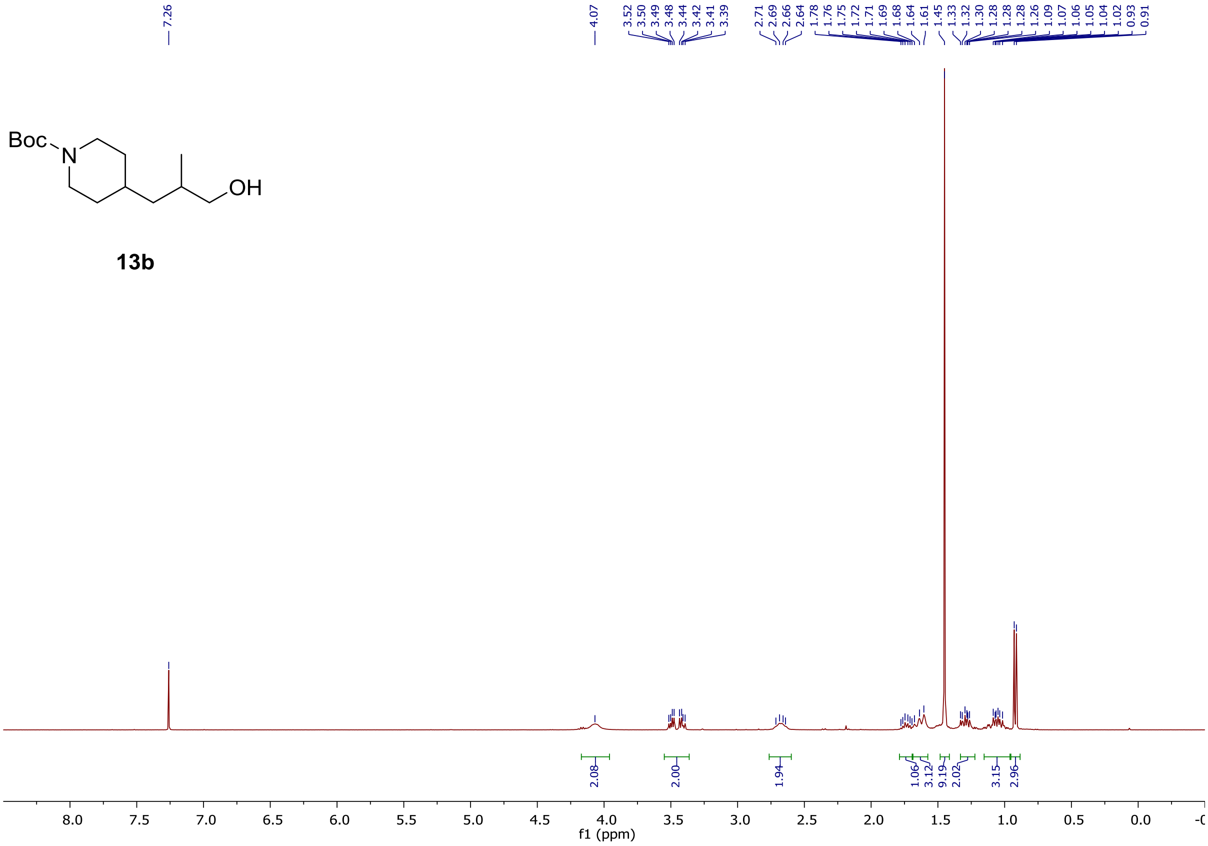
7.26
4.07
3.52 3.50 3.49 3.48 3.44 3.42 3.41 3.39
2.71 2.69 2.66 2.64
1.78 1.76 1.75 1.72 1.71 1.69 1.68 1.64 1.61 1.45 1.33 1.32 1.30 1.28 1.28 1.28 1.26 1.09 1.07 1.06 1.05 1.04 1.02 0.93 0.91
Boc
N
OH
13b
2.08
2.00
1.94
1.06
3.12
9.19
2.02
3.15
2.96
f1 (ppm)

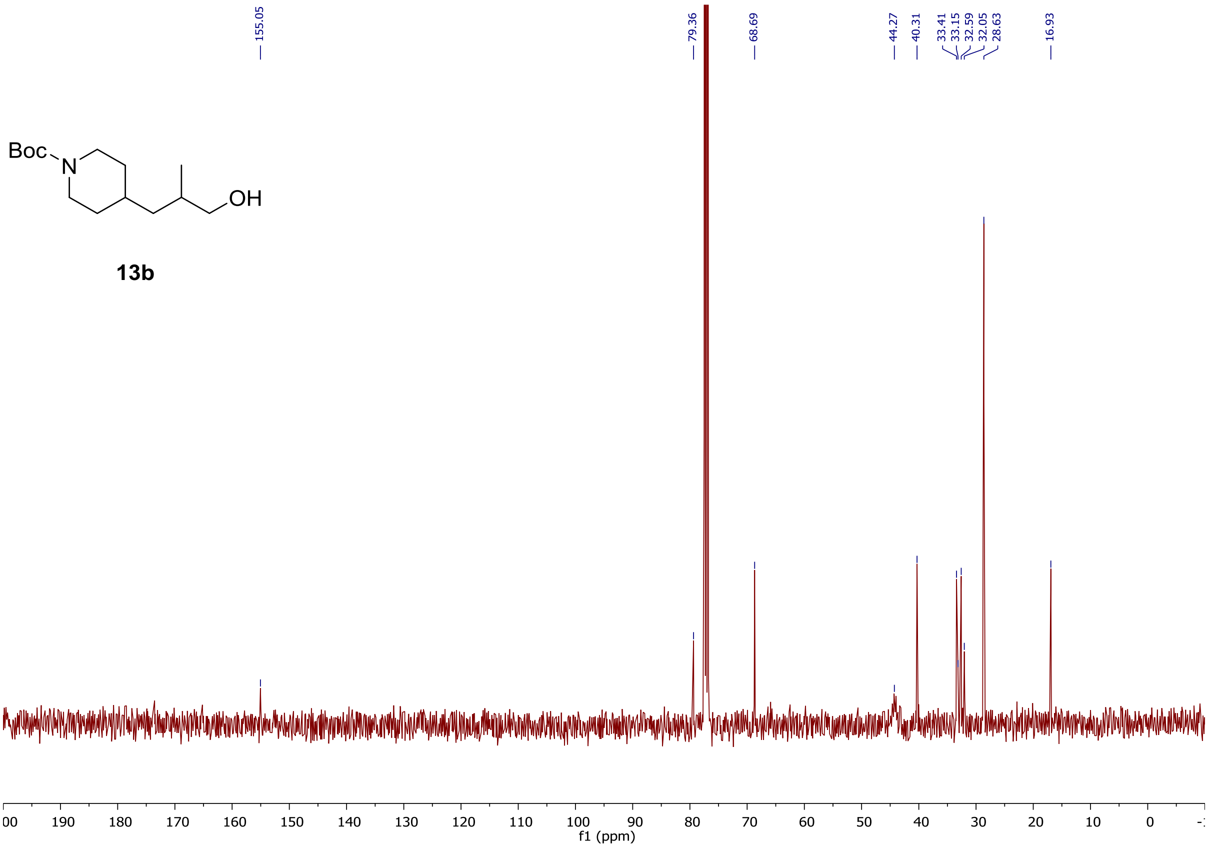
155.05
79.36
68.69
44.27
40.31
33.41 33.15 32.59 32.05 28.63
16.93
Boc
N
OH
13b
f1 (ppm)

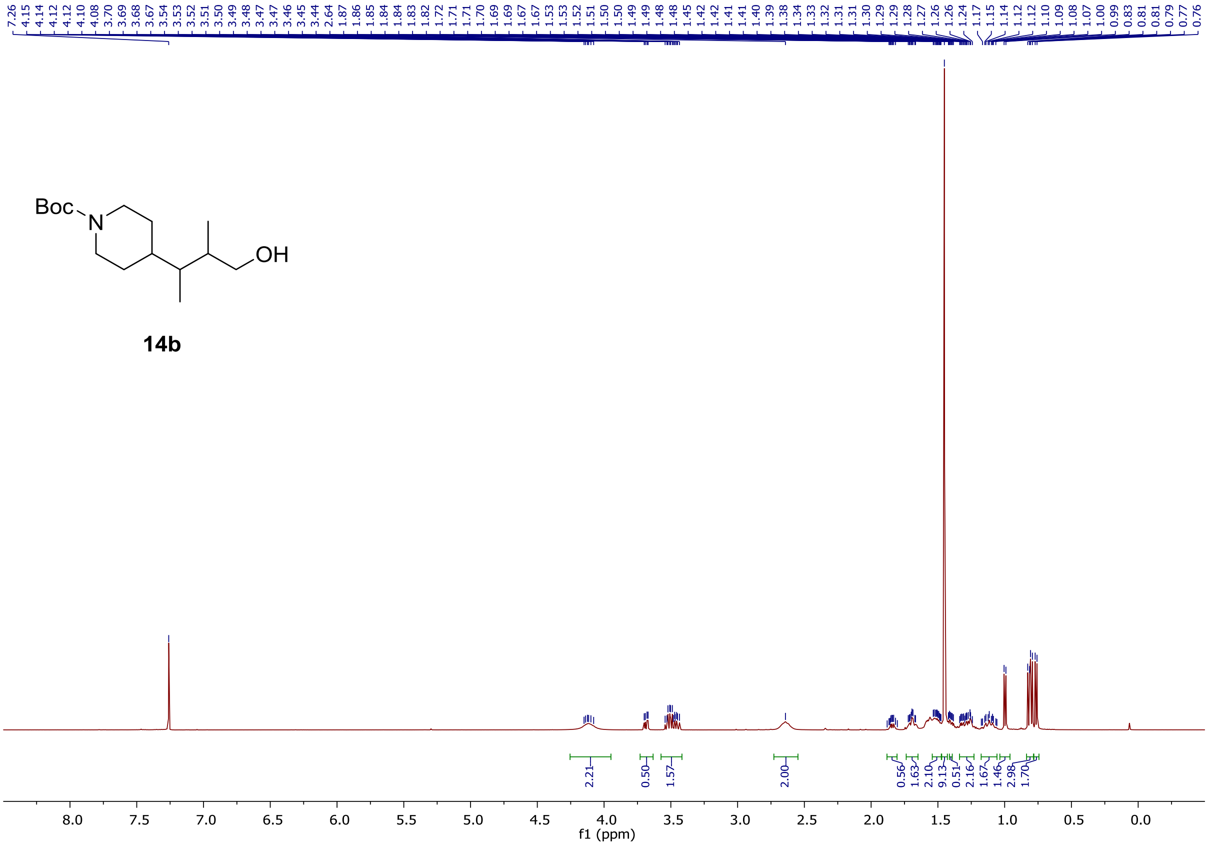
7.26 4.15 4.14 4.12 4.12 4.10 4.08 3.70 3.69 3.68 3.67 3.54 3.53 3.52 3.51 3.50 3.49 3.48 3.47 3.46 3.45 3.44 2.64 1.87 1.86 1.85 1.84 1.83 1.82 1.72 1.71 1.70 1.69 1.67 1.67 1.53 1.53 1.52 1.51 1.50 1.50 1.49 1.49 1.48 1.48 1.45 1.42 1.42 1.41 1.41 1.40 1.39 1.38 1.34 1.33 1.32 1.31 1.31 1.30 1.29 1.29 1.28 1.27 1.26 1.26 1.24 1.17 1.15 1.14 1.12 1.12 1.10 1.09 1.08 1.07 1.00 0.99 0.83 0.81 0.81 0.79 0.77 0.76
Boc
N
OH
14b
2.21 0.50 1.57 2.00 0.56 1.63 2.10 9.13 0.51 2.16 1.67 1.46 2.98 1.70
8.0 7.5 7.0 6.5 6.0 5.5 5.0 4.5 4.0 3.5 3.0 2.5 2.0 1.5 1.0 0.5 0.0
f1 (ppm)

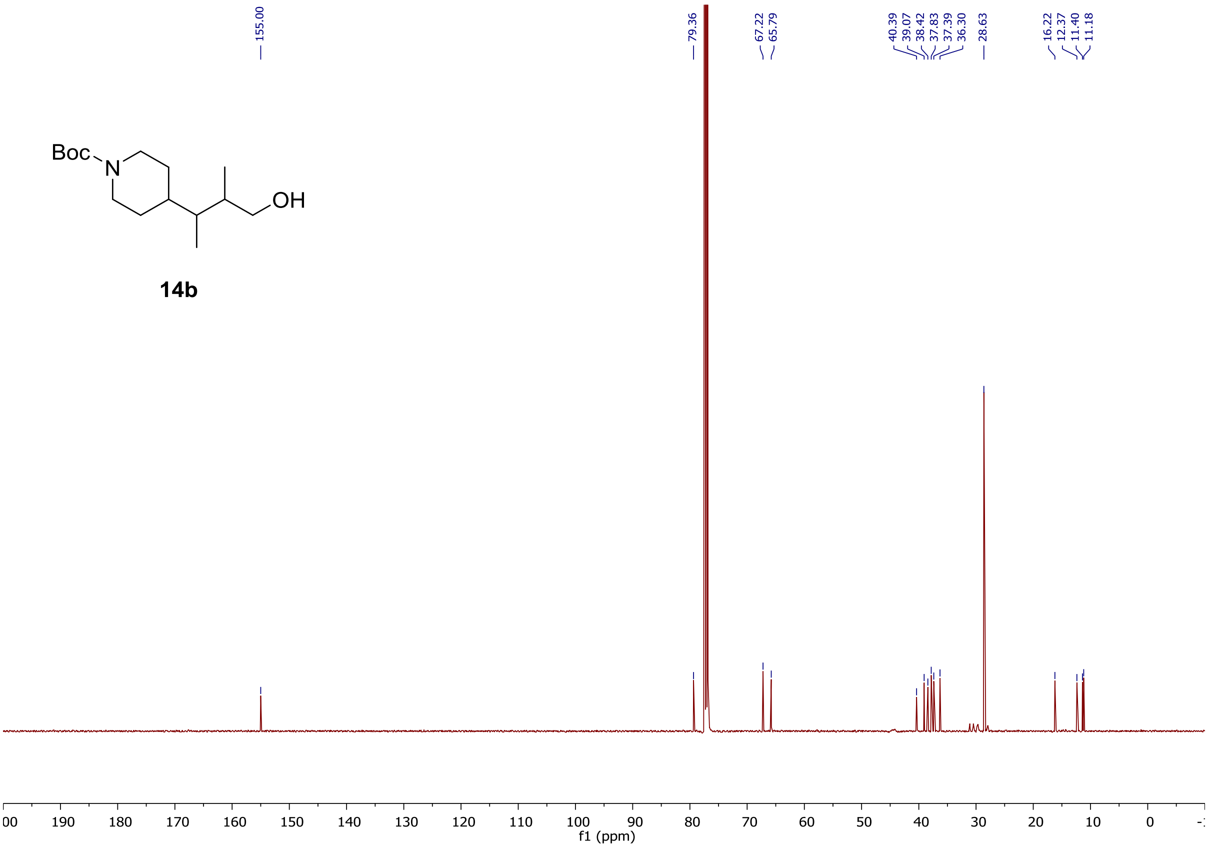
155.00
79.36
67.22 65.79
40.39 39.07 38.42 37.83 37.39 36.30
28.63
16.22 12.37 11.40 11.18
Boc
N
OH
14b
00 190 180 170 160 150 140 130 120 110 100 90 80 70 60 50 40 30 20 10 0
f1 (ppm)

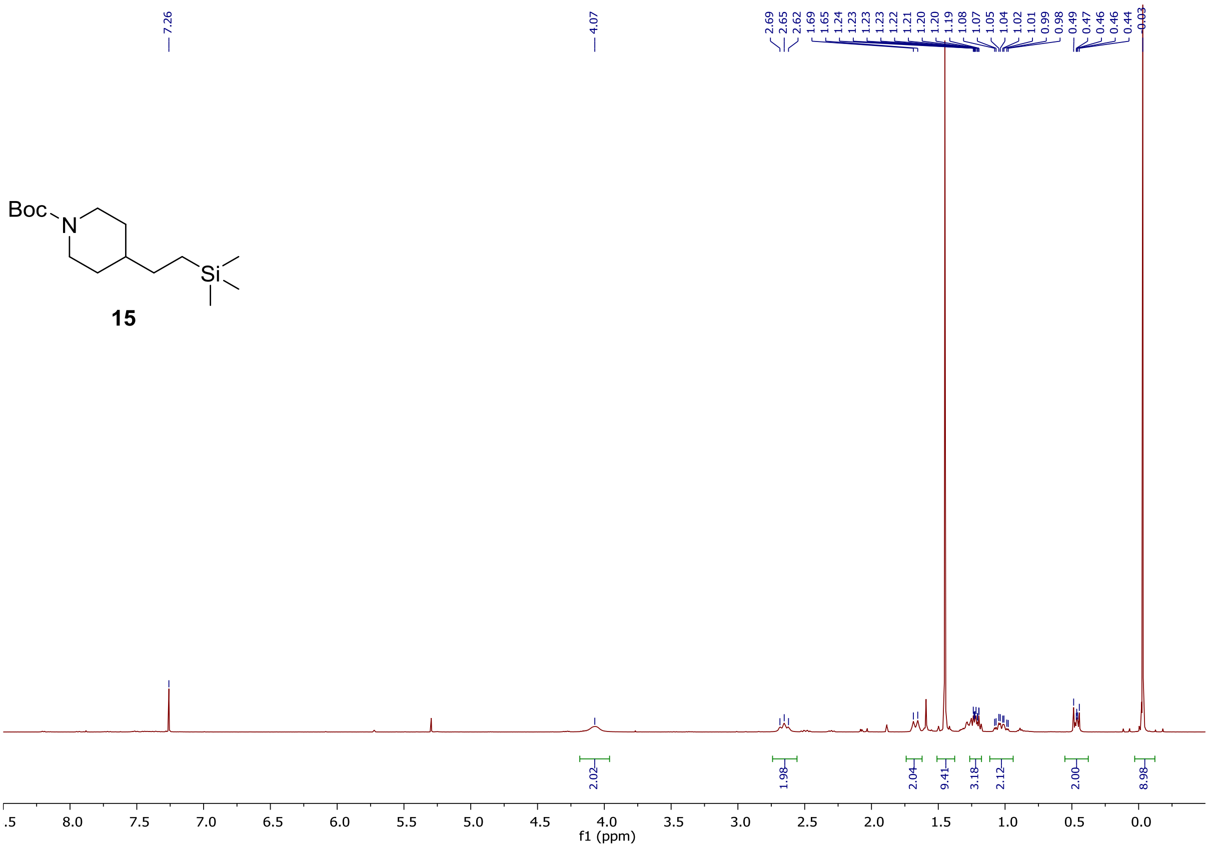
7.26
4.07
2.69 2.65 2.62 1.69 1.65 1.24 1.23 1.23 1.23 1.22 1.21 1.20 1.20 1.19 1.08 1.07 1.05 1.04 1.02 1.01 0.99 0.98 0.49 0.47 0.46 0.46 0.44 0.03
Boc
N
Si
15
2.02 1.98 2.04 9.41 3.18 2.12 2.00 8.98
f1 (ppm)

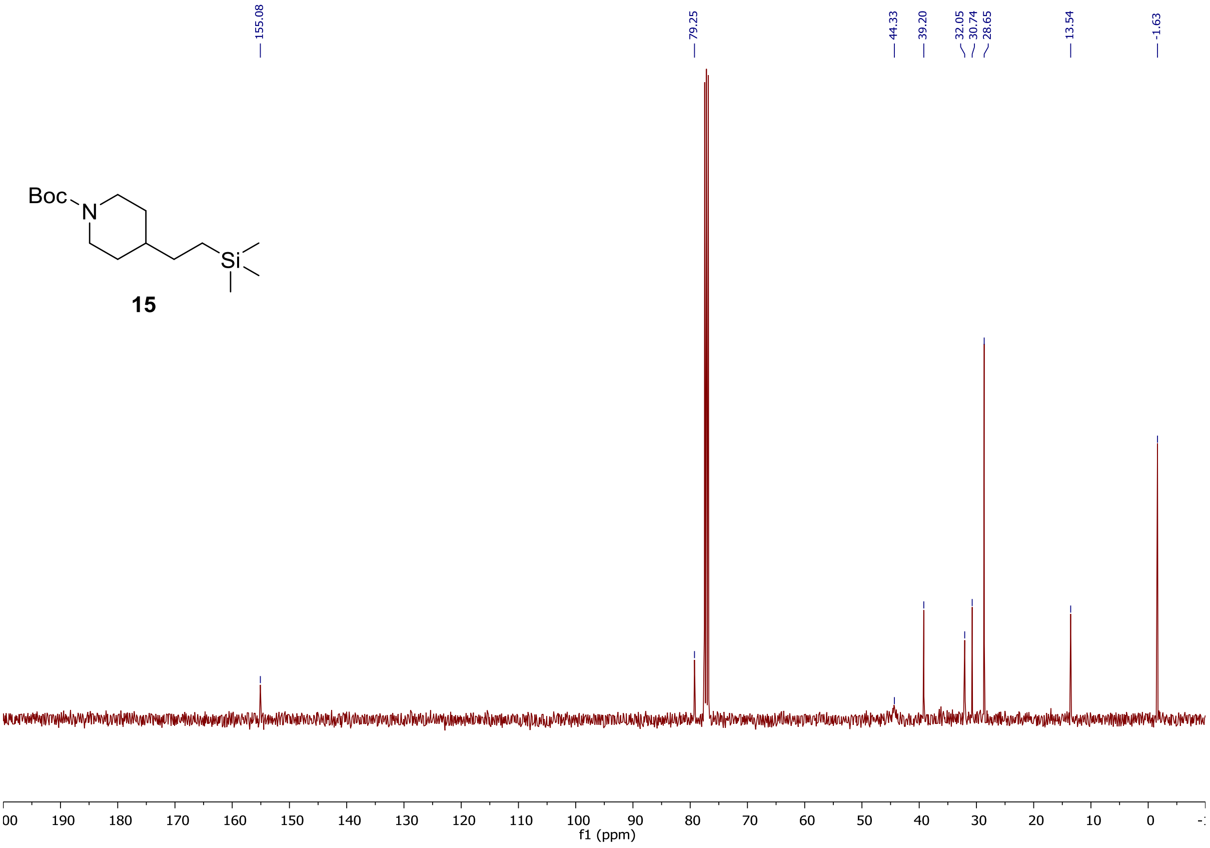
155.08
79.25
44.33 39.20 32.05 30.74 28.65
13.54
-1.63
Boc
N
Si
15
f1 (ppm)

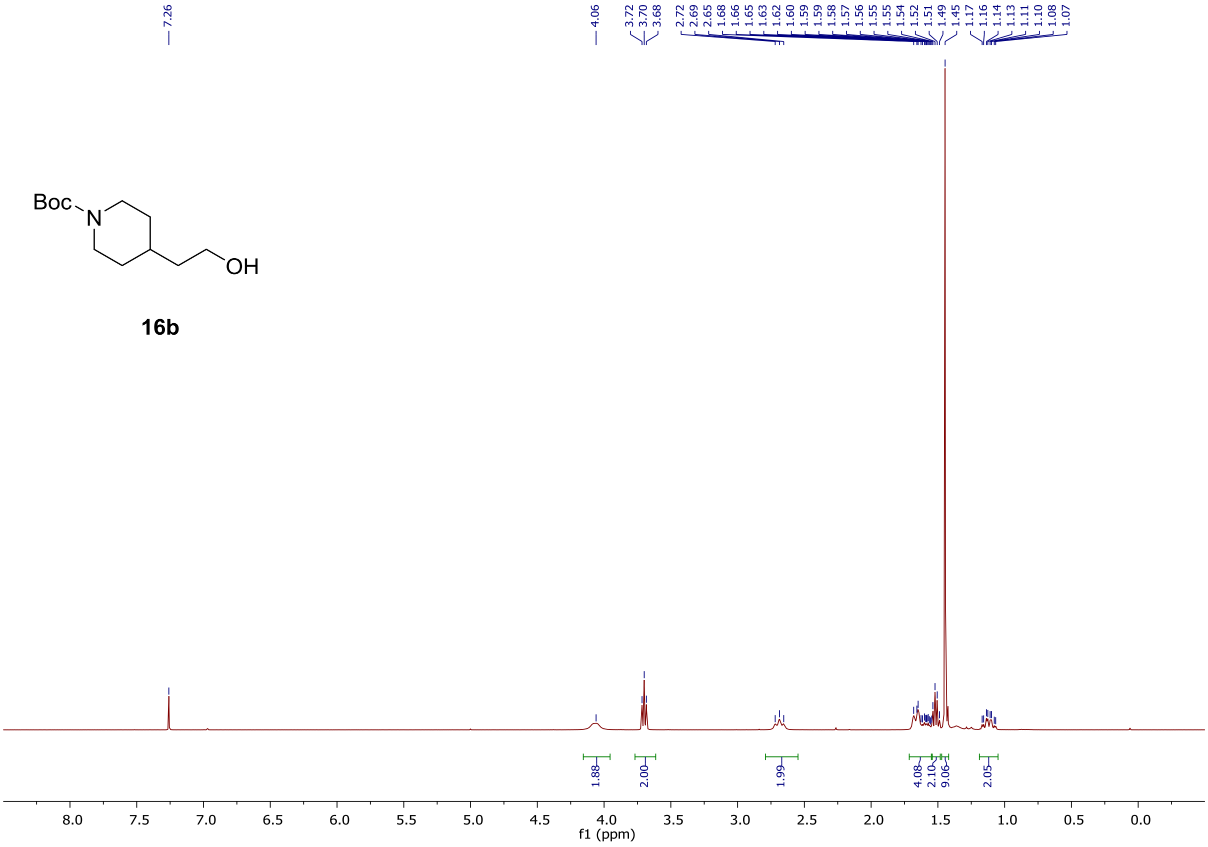
Boc
N
OH
7.26
4.06
3.72
3.70
3.68
2.72
2.69
2.65
1.68
1.66
1.65
1.63
1.62
1.60
1.59
1.59
1.58
1.57
1.56
1.55
1.55
1.54
1.52
1.51
1.49
1.45
1.17
1.16
1.14
1.13
1.11
1.10
1.08
1.07
1.88
2.00
1.99
4.08
2.10
9.06
2.05
f1 (ppm)

16b

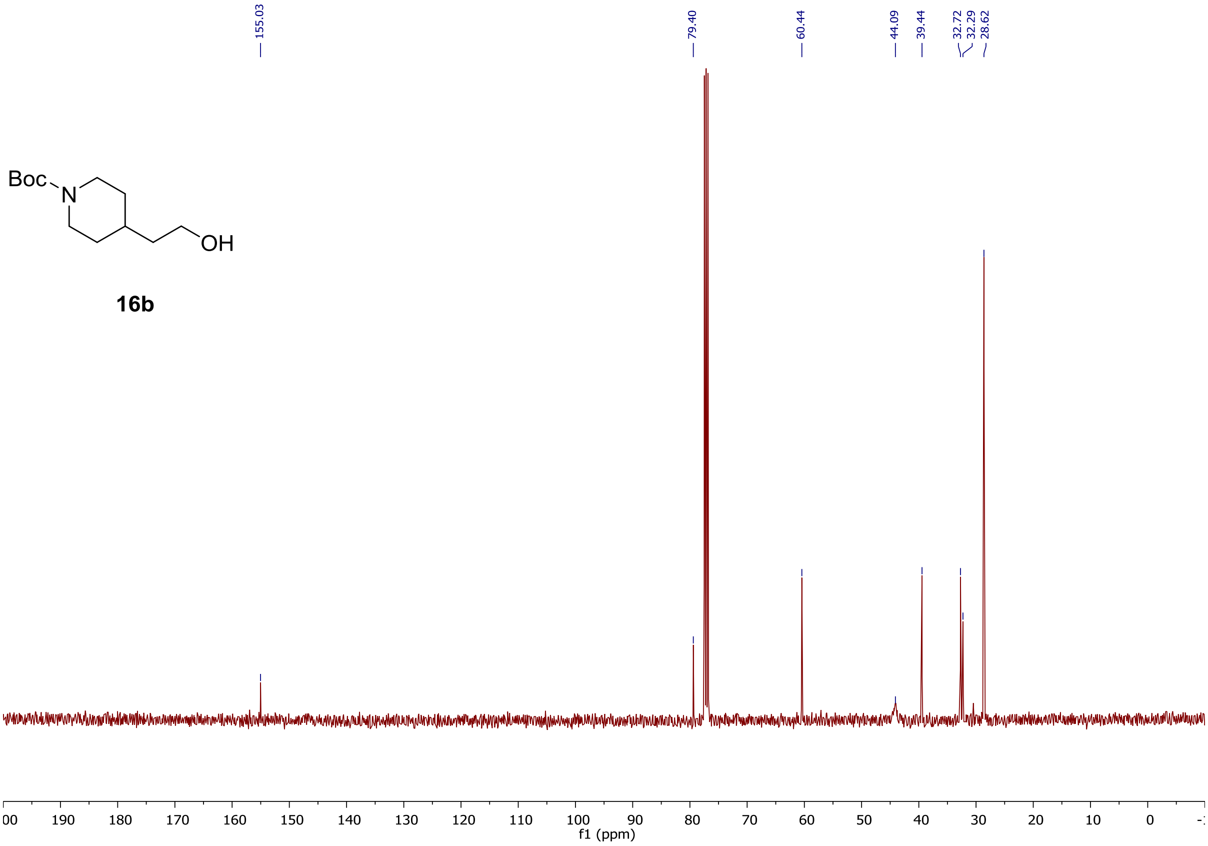
Boc
N
OH
155.03
79.40
60.44
44.09
39.44
32.72
32.29
28.62
f1 (ppm)

16b

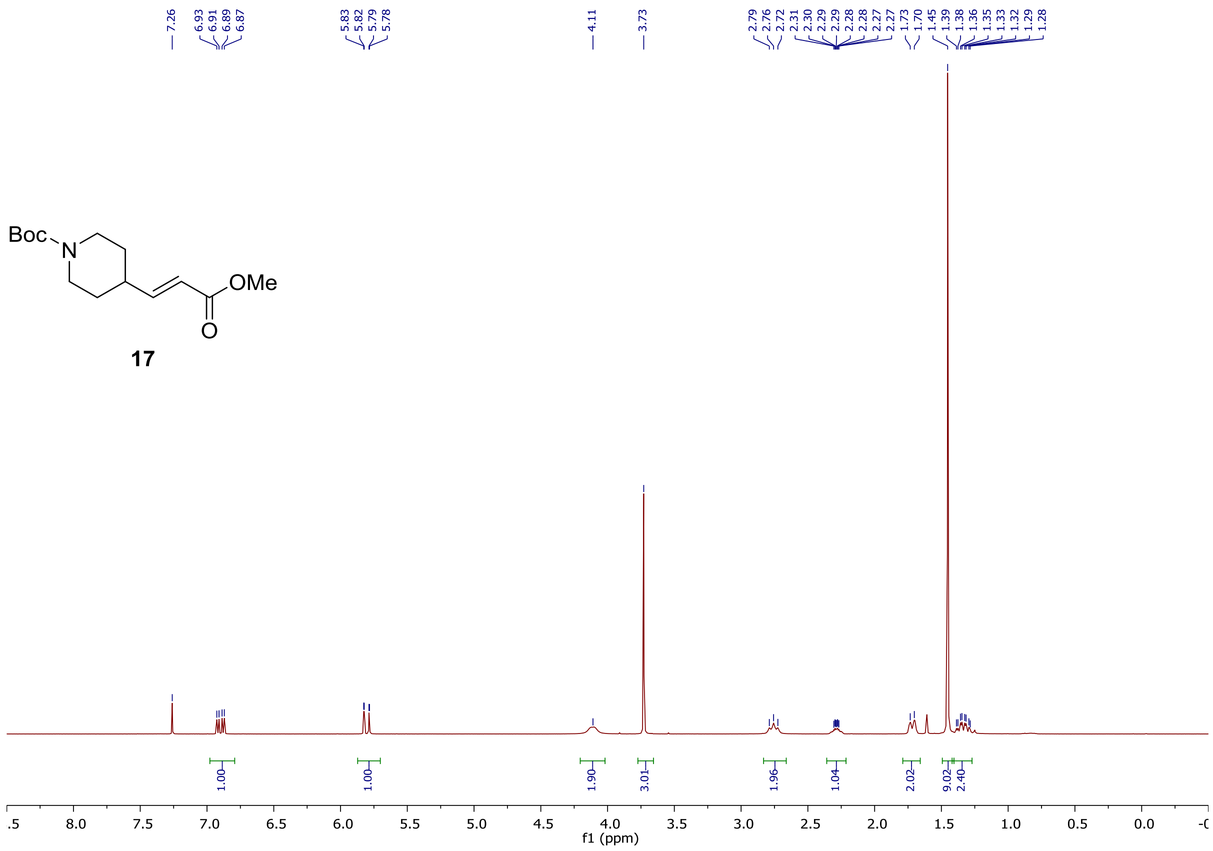

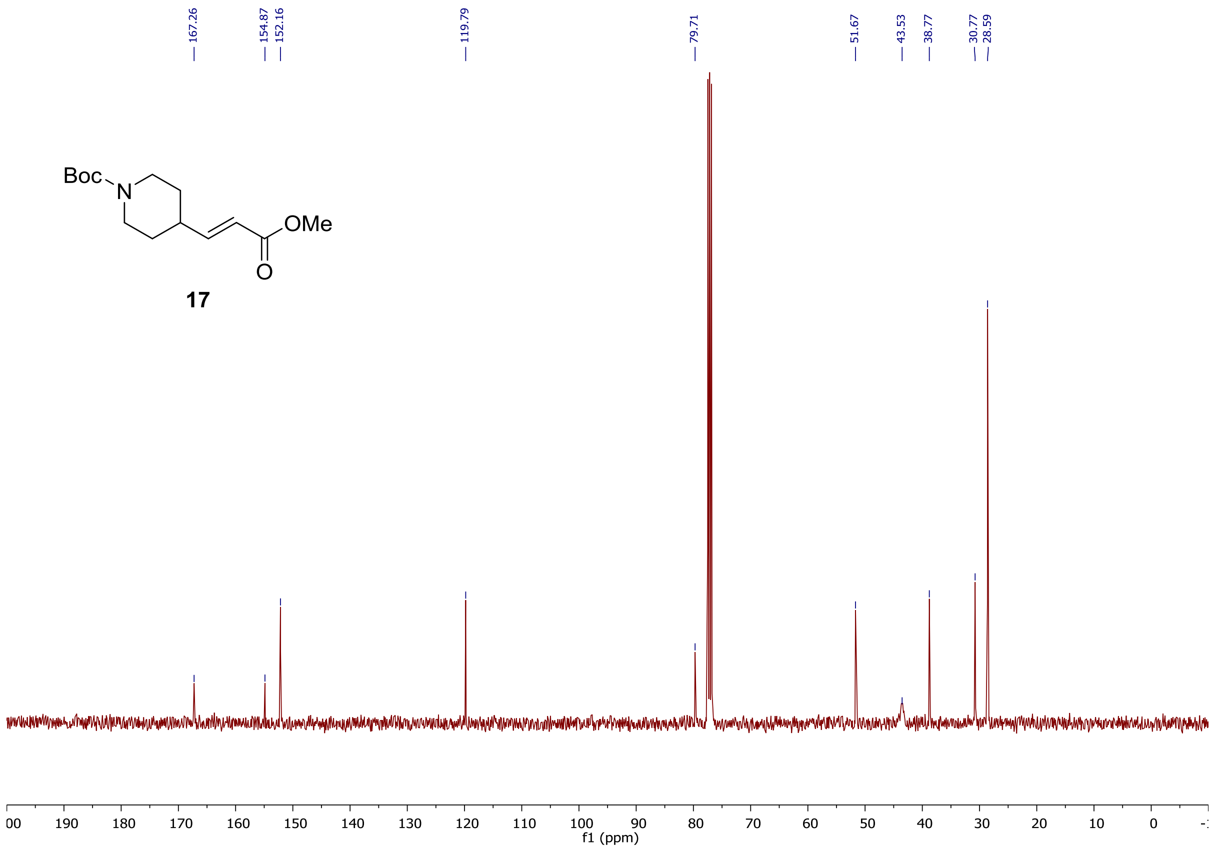

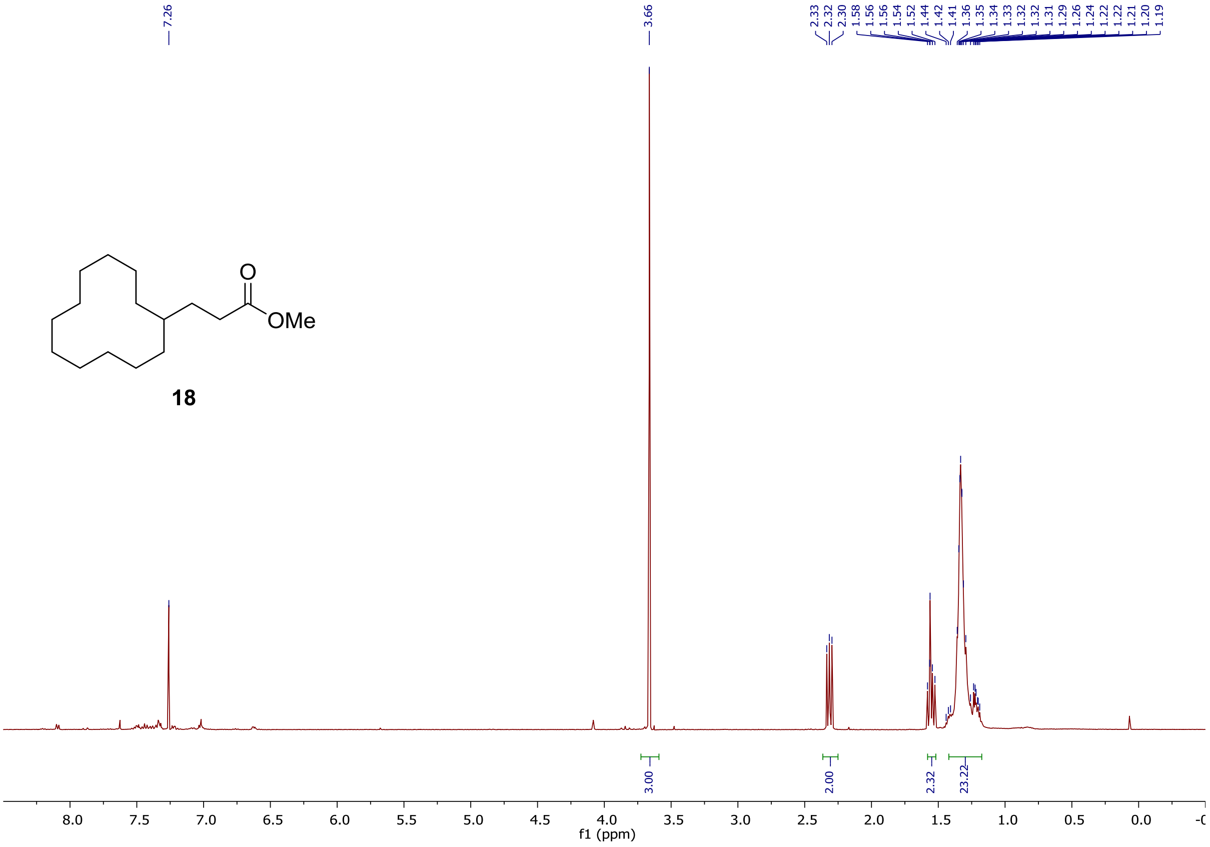
7.26
3.66
2.33 2.32 2.30 1.58 1.56 1.56 1.54 1.52 1.44 1.42 1.41 1.36 1.35 1.34 1.33 1.32 1.32 1.31 1.29 1.26 1.24 1.22 1.22 1.21 1.20 1.19
O
OMe
18
3.00
2.00
2.32
23.22
f1 (ppm)

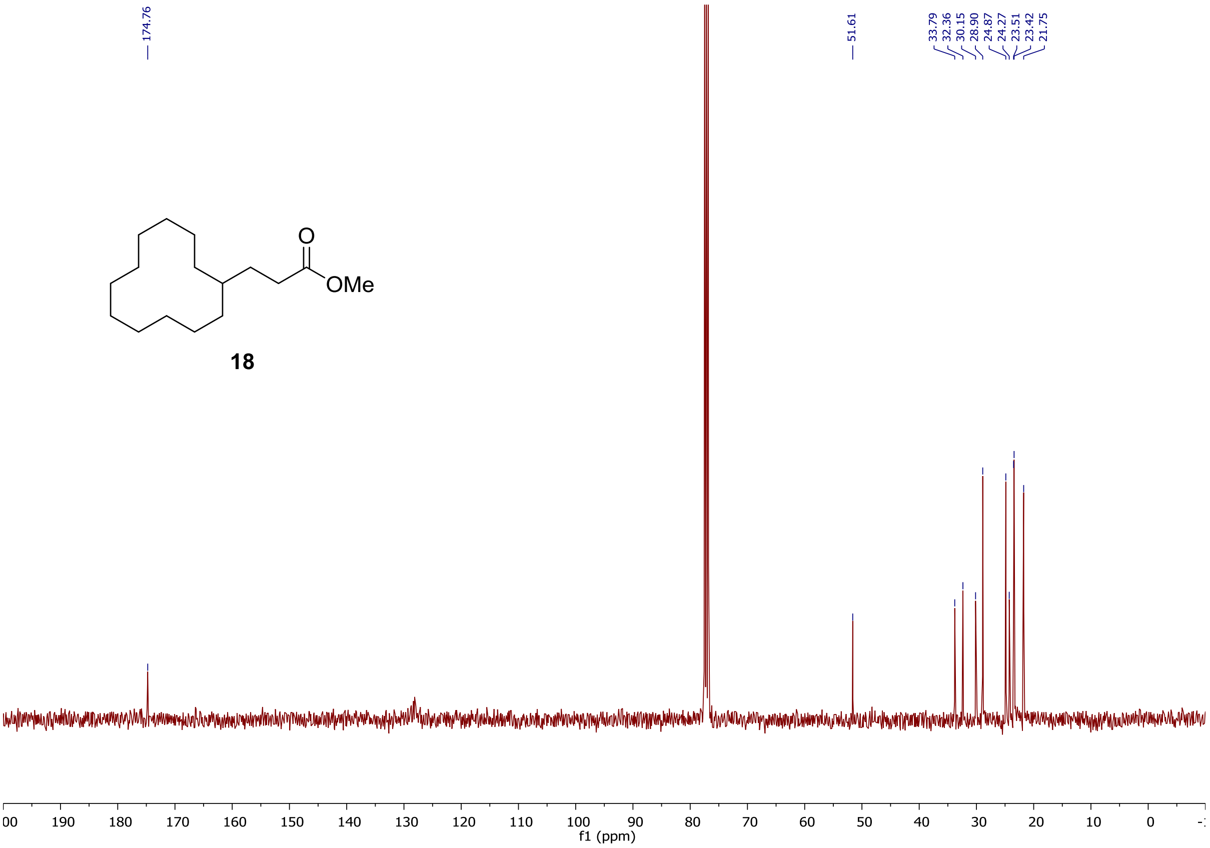
174.76
51.61
33.79 32.36 30.15 28.90 24.87 24.27 23.51 23.42 21.75
O
OMe
18
f1 (ppm)

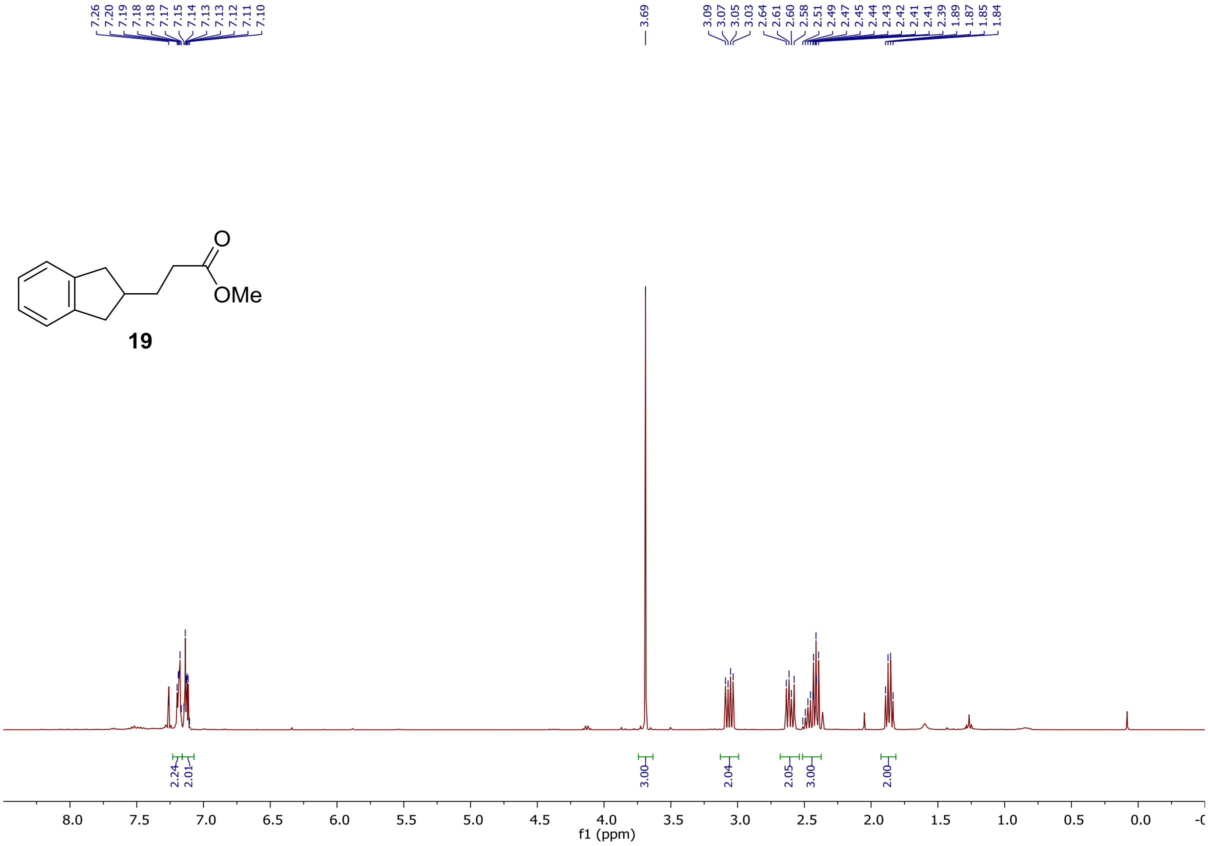

7.26
7.20
7.19
7.18
7.18
7.17
7.15
7.14
7.13
7.13
7.12
7.11
7.10
3.69
3.09
3.07
3.05
3.03
2.64
2.61
2.60
2.58
2.51
2.49
2.47
2.45
2.44
2.43
2.42
2.41
2.41
2.39
1.89
1.87
1.85
1.84
O
OMe
19
2.24
2.01
3.00
2.04
2.05
3.00
2.00
f1 (ppm)

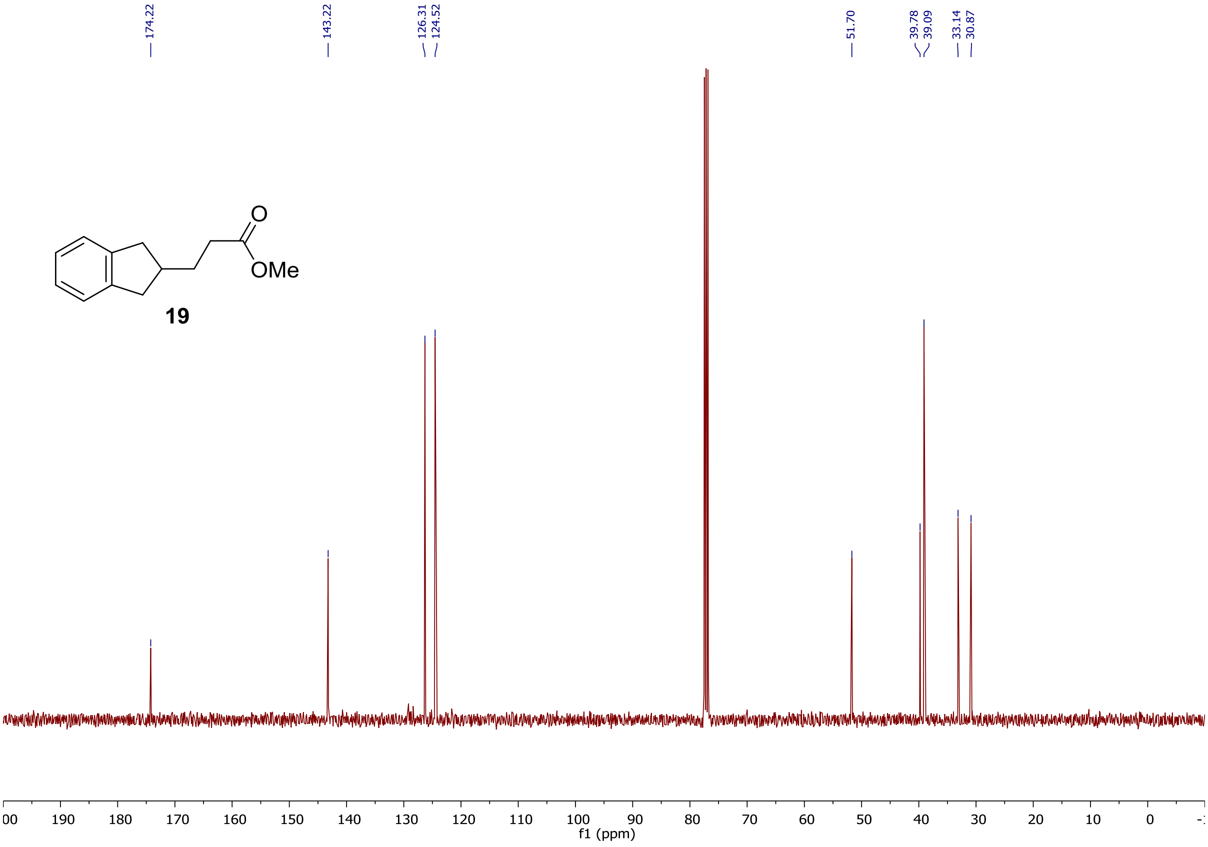

174.22
143.22
126.31
124.52
51.70
39.78
39.09
33.14
30.87
O
OMe
19
f1 (ppm)

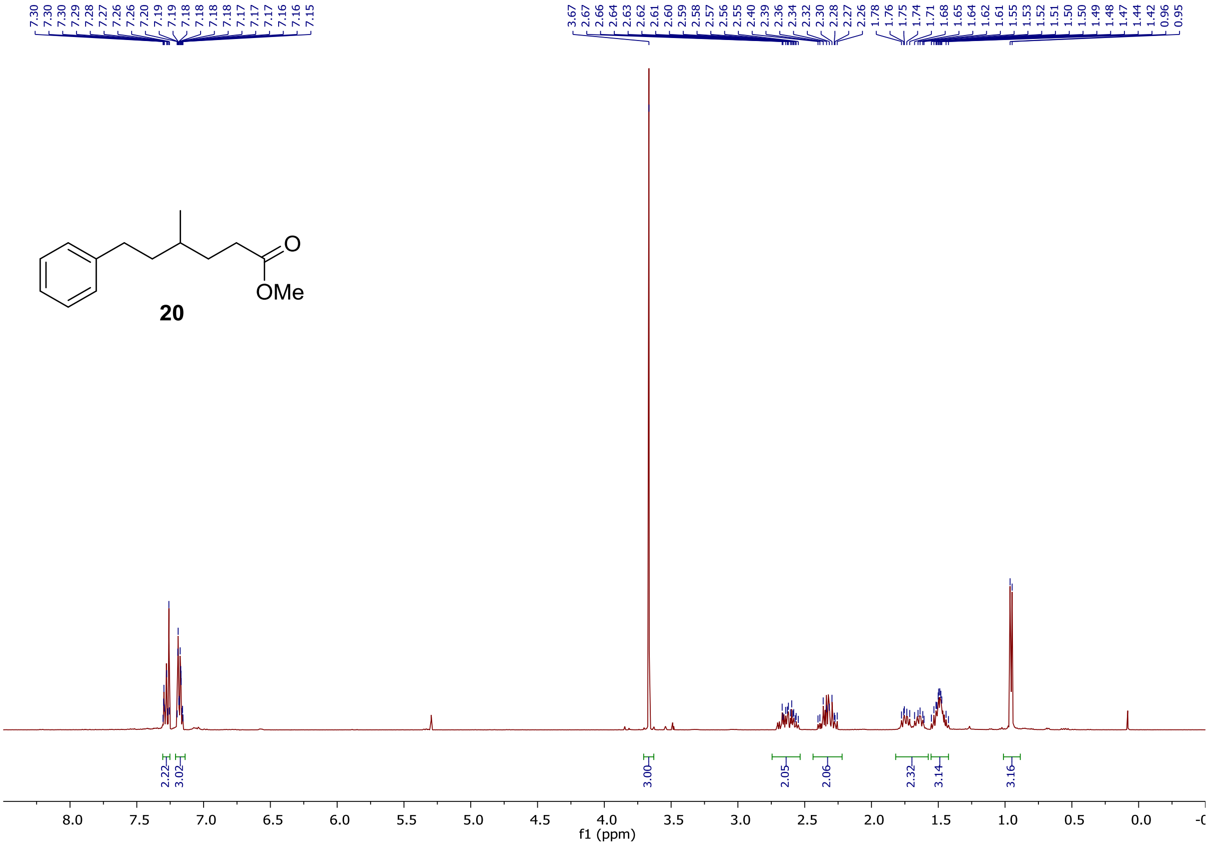
7.30 7.30 7.29 7.28 7.27 7.26 7.26 7.20 7.19 7.19 7.18 7.18 7.18 7.18 7.17 7.17 7.16 7.15
3.67 2.67 2.66 2.64 2.63 2.62 2.61 2.60 2.59 2.58 2.57 2.56 2.55 2.40 2.39 2.36 2.34 2.32 2.30 2.28 2.27 2.26 1.78 1.76 1.75 1.74 1.71 1.68 1.65 1.64 1.62 1.61 1.55 1.53 1.52 1.51 1.50 1.49 1.48 1.47 1.44 1.42 0.96 0.95
O
OMe
20
2.22 3.02 3.00 2.05 2.06 2.32 3.14 3.16
f1 (ppm)

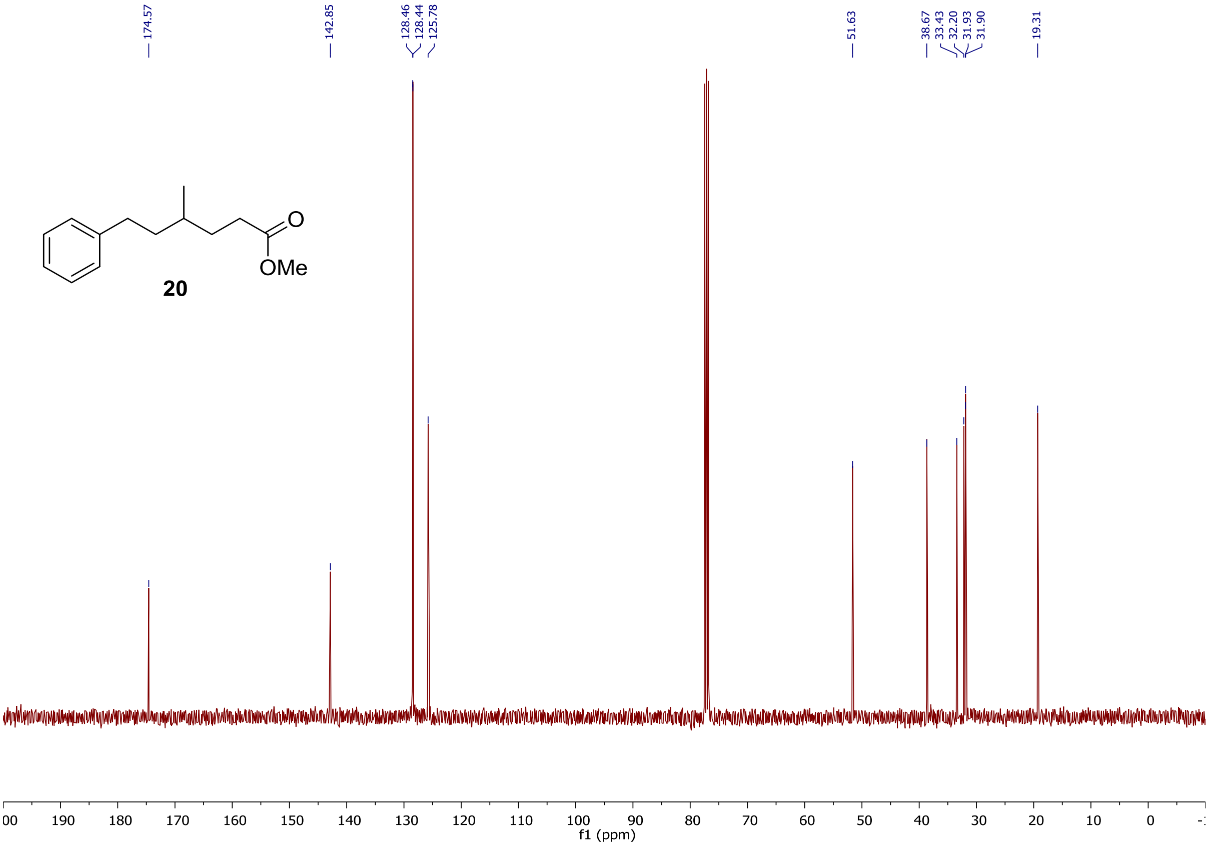
174.57
142.85
128.46 128.44 125.78
51.63
38.67 33.43 32.20 31.93 31.90
19.31
O
OMe
20
f1 (ppm)

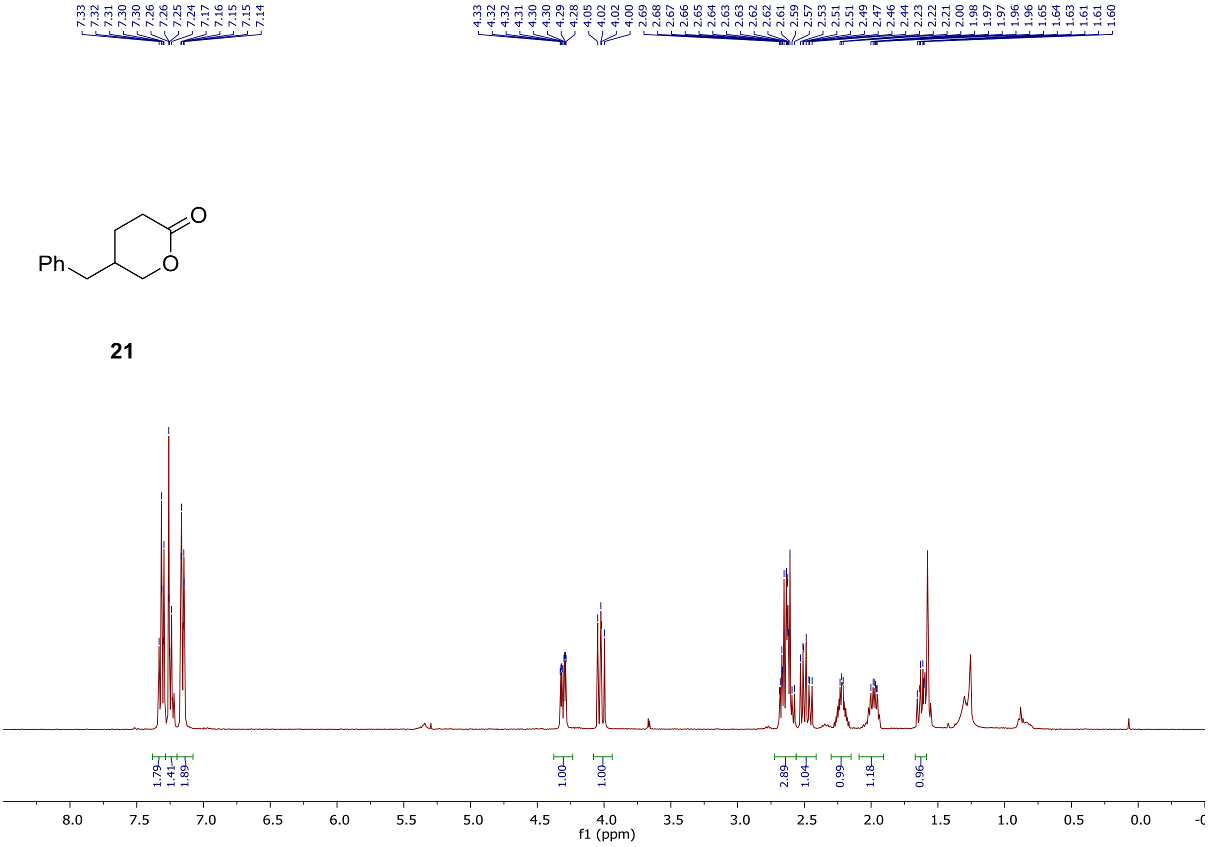
7.33 7.32 7.31 7.30 7.30 7.26 7.25 7.24 7.17 7.16 7.15 7.15 7.14
4.33 4.32 4.32 4.31 4.30 4.30 4.29 4.28 4.05 4.02 4.02 4.00 2.69 2.68 2.67 2.66 2.65 2.64 2.63 2.63 2.62 2.61 2.59 2.57 2.53 2.51 2.51 2.49 2.47 2.46 2.44 2.23 2.22 2.21 2.00 1.98 1.97 1.97 1.96 1.96 1.65 1.64 1.63 1.61 1.61 1.60
Ph
O
O
21
1.79 1.41 1.89 1.00 1.00 2.89 1.04 0.99 1.18 0.96
f1 (ppm)

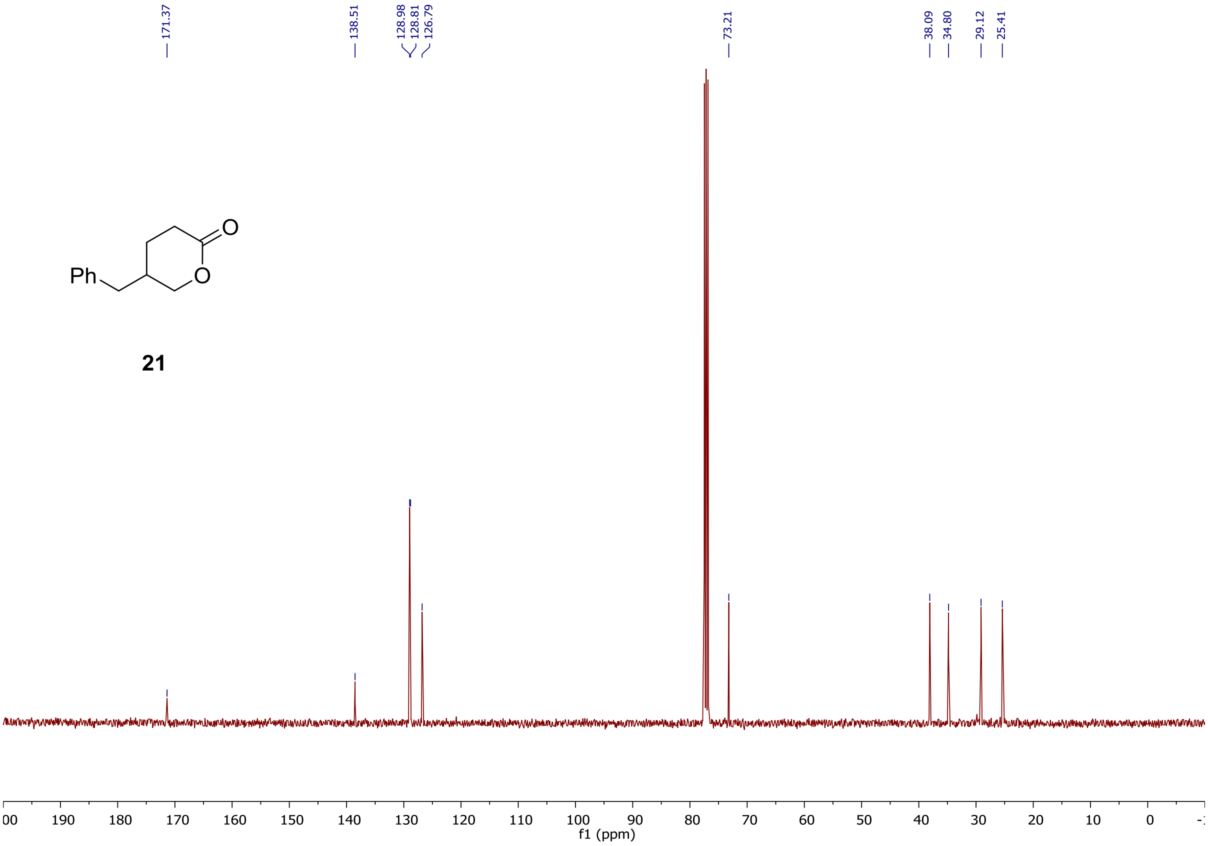
171.37
138.51
128.98 128.81 126.79
73.21
38.09 34.80 29.12 25.41
Ph
O
O
21
f1 (ppm)

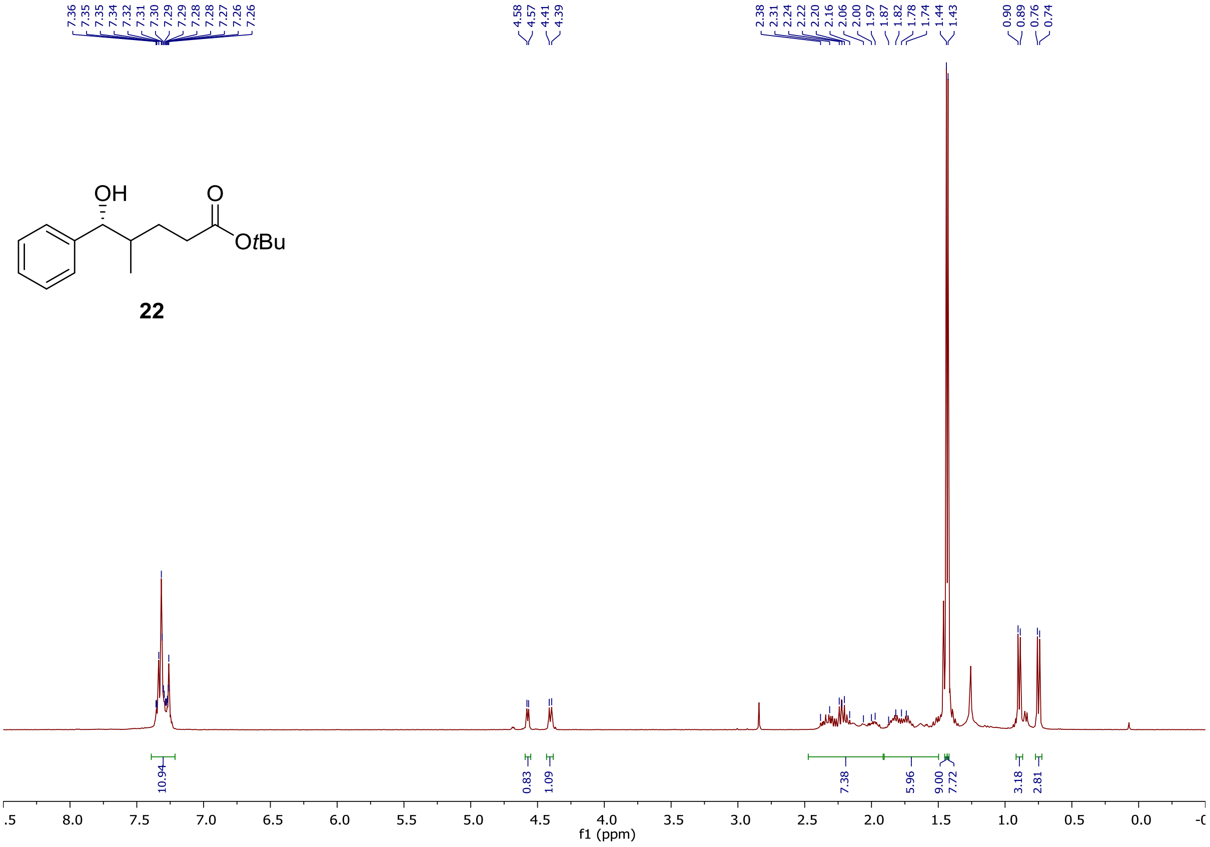
7.36 7.35 7.35 7.34 7.32 7.31 7.30 7.29 7.29 7.28 7.28 7.27 7.26 7.26
4.58 4.57 4.41 4.39
2.38 2.31 2.24 2.22 2.20 2.16 2.06 2.00 1.97 1.87 1.82 1.78 1.74 1.44 1.43
0.90 0.89 0.76 0.74
OH
O
OtBu
22
10.94
0.83
1.09
7.38
5.96
9.00
7.72
3.18
2.81
f1 (ppm)

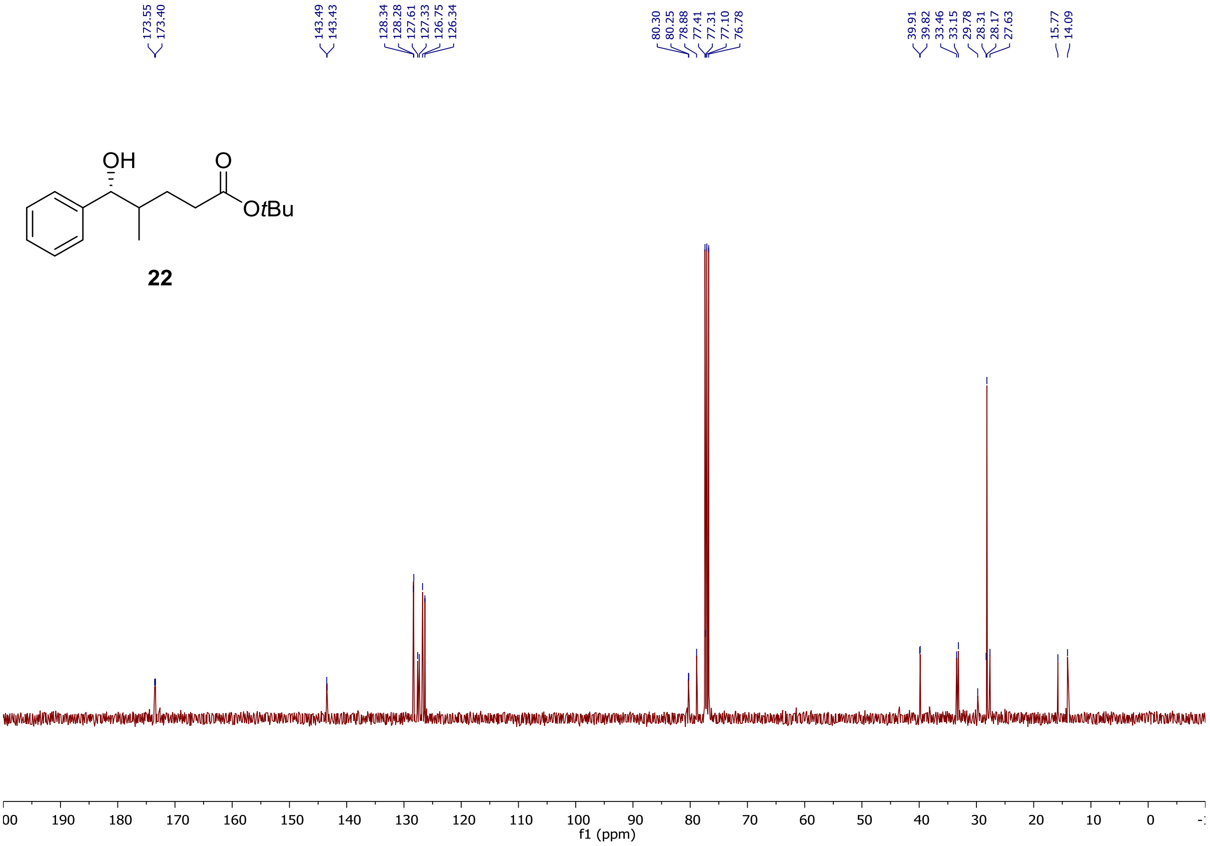
173.55 173.40
143.49 143.43
128.34 128.28 127.61 127.33 126.75 126.34
80.30 80.25 78.88 77.41 77.31 77.10 76.78
39.91 39.82 33.46 33.15 29.78 28.31 28.17 27.63
15.77 14.09
OH
O
OtBu
22
f1 (ppm)

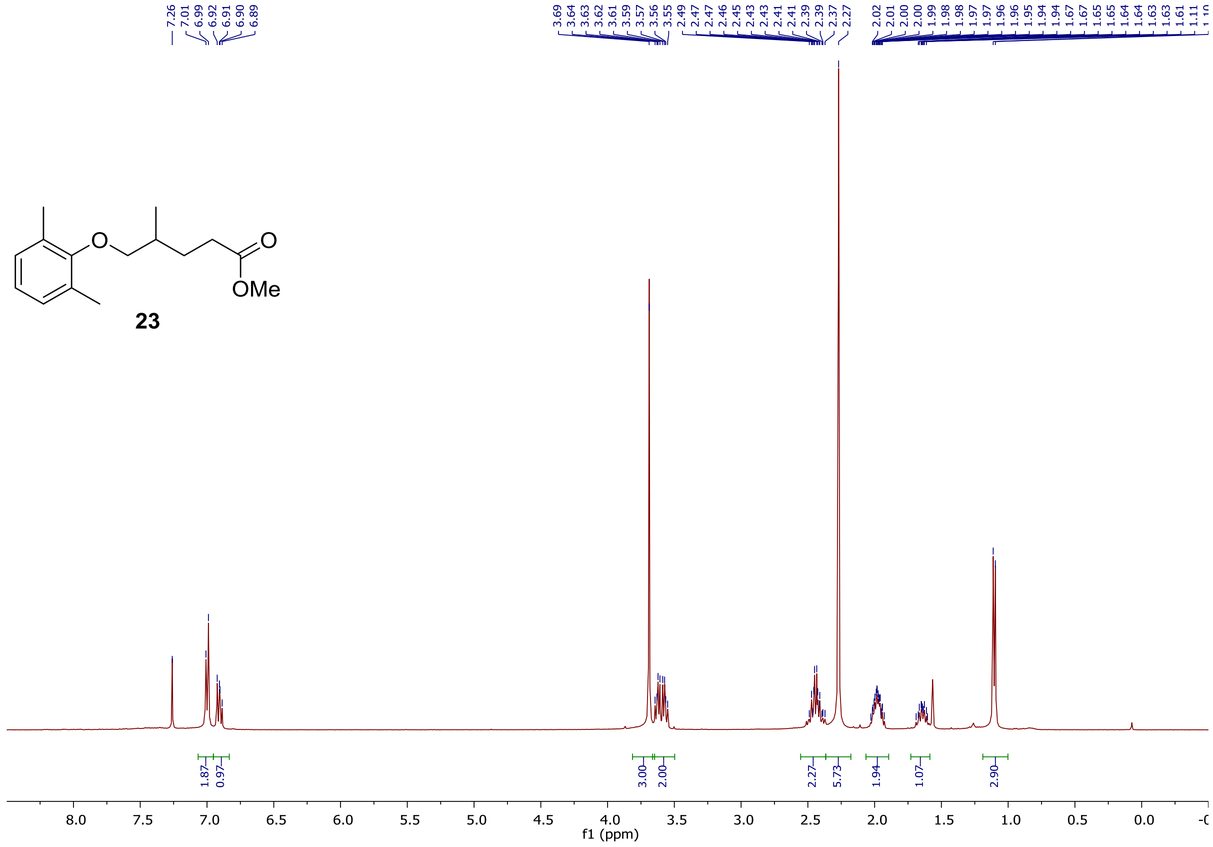

OMe
23
f1 (ppm)

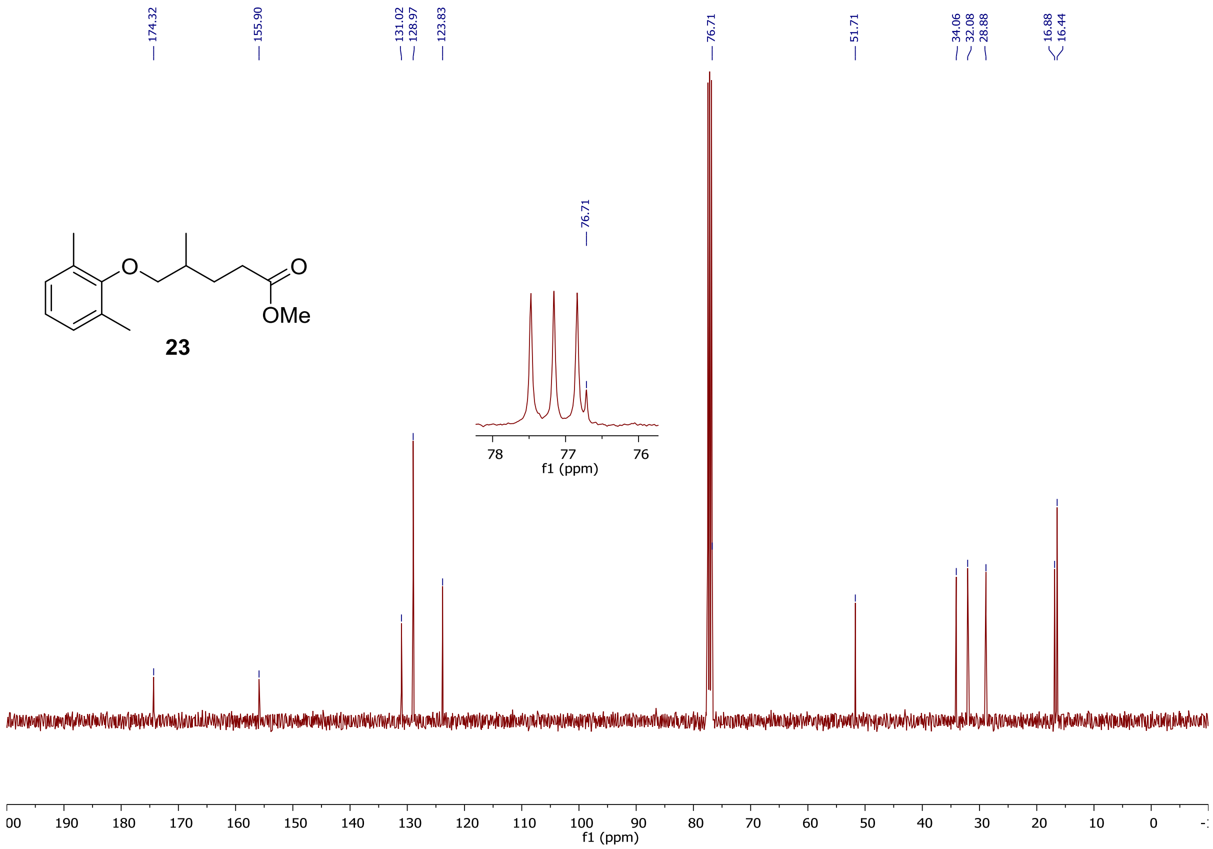

OMe
23
f1 (ppm)

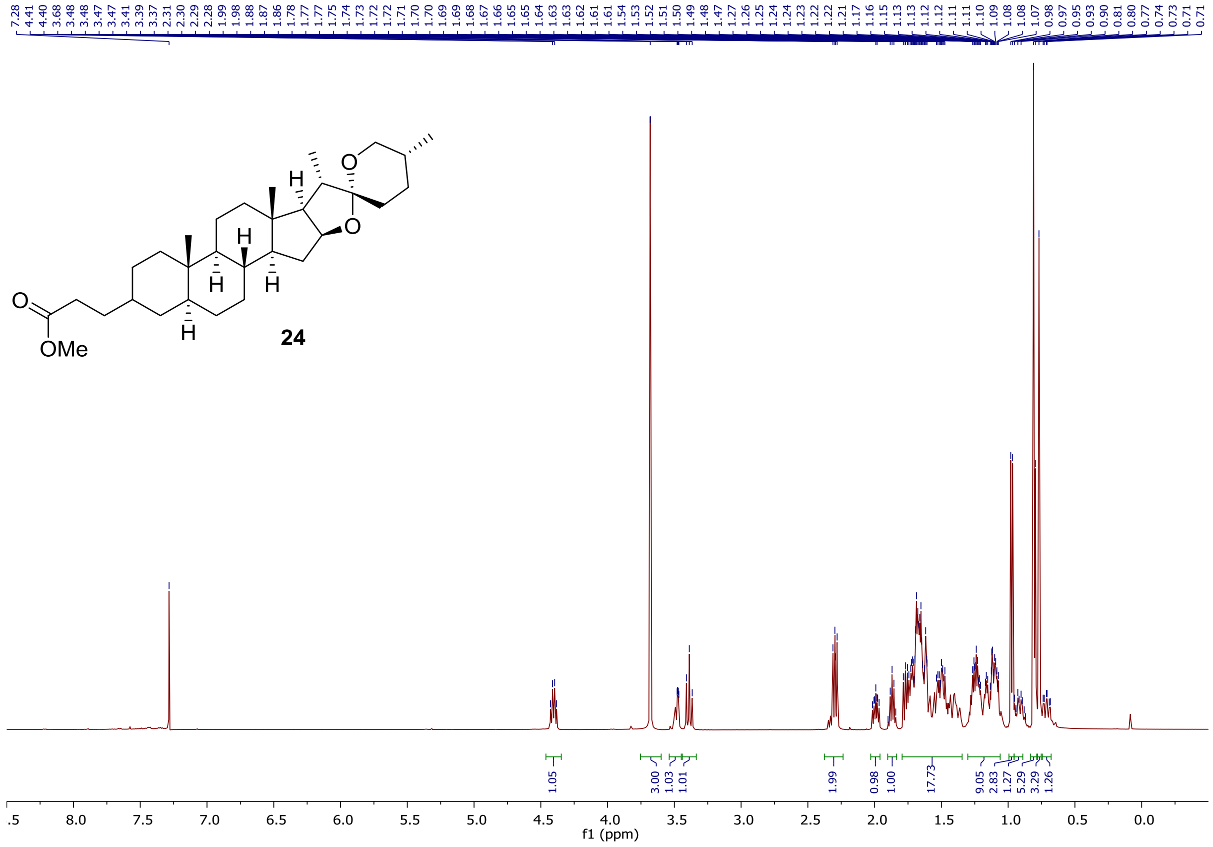
7.28 4.41 4.40 3.68 3.48 3.48 3.47 3.47 3.41 3.39 3.37 2.31 2.30 2.29 2.28 1.99 1.98 1.88 1.87 1.86 1.78 1.77 1.77 1.75 1.74 1.73 1.72 1.72 1.71 1.70 1.69 1.69 1.68 1.67 1.66 1.65 1.65 1.64 1.63 1.63 1.62 1.61 1.61 1.54 1.53 1.52 1.51 1.50 1.49 1.48 1.47 1.27 1.26 1.25 1.24 1.24 1.23 1.22 1.22 1.21 1.17 1.16 1.15 1.13 1.13 1.12 1.12 1.11 1.11 1.10 1.09 1.08 1.08 1.07 0.98 0.97 0.95 0.93 0.90 0.81 0.80 0.77 0.74 0.73 0.71 0.71
O
OMe
H
24
1.05 3.00 1.03 1.01 1.99 0.98 1.00 17.73 9.05 2.83 1.27 5.29 3.29 1.26
f1 (ppm)

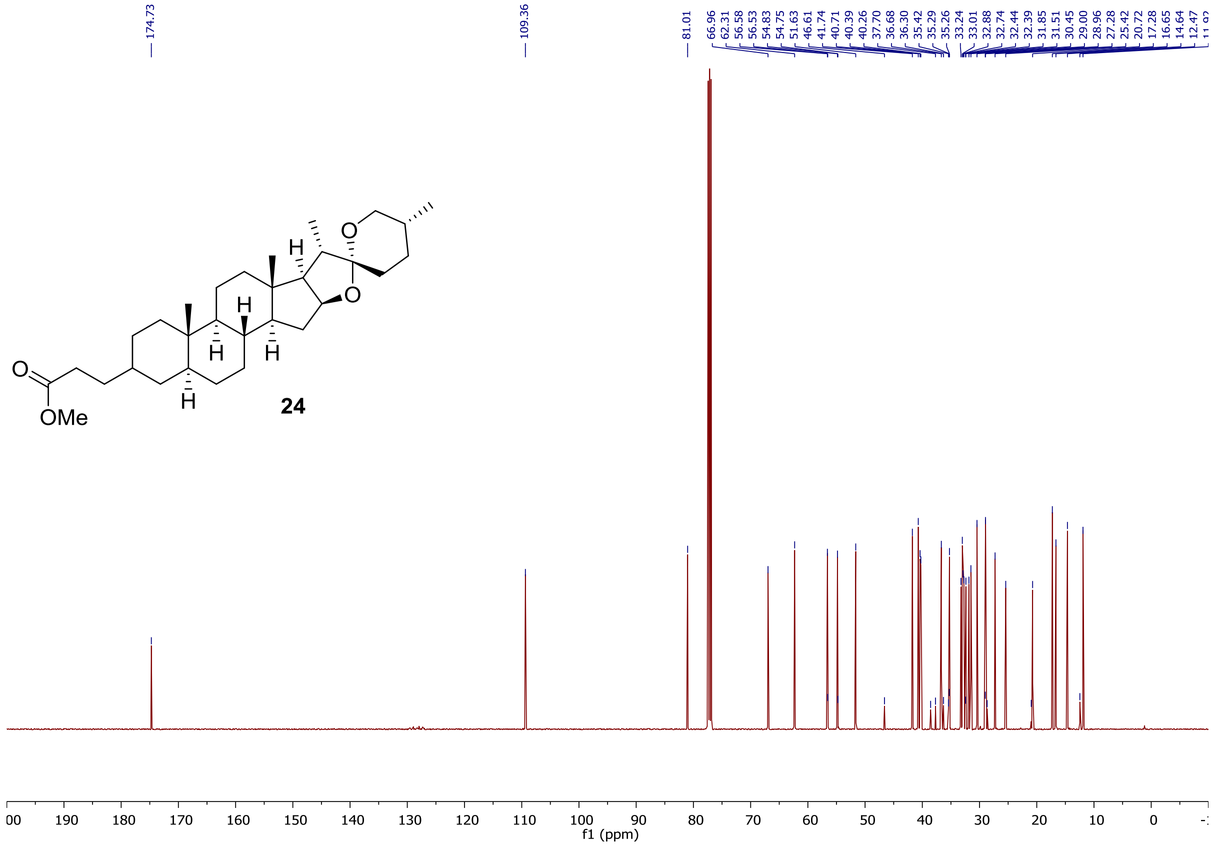
174.73
109.36
81.01
66.96 62.31 56.58 56.53 54.83 54.75 51.63 46.61 41.74 40.71 40.39 40.26 37.70 36.68 36.30 35.42 35.29 35.26 33.24 33.01 32.88 32.74 32.44 32.39 31.85 31.51 30.45 29.00 28.96 27.28 25.42 20.72 17.28 16.65 14.64 12.47
O
OMe
H
24
f1 (ppm)

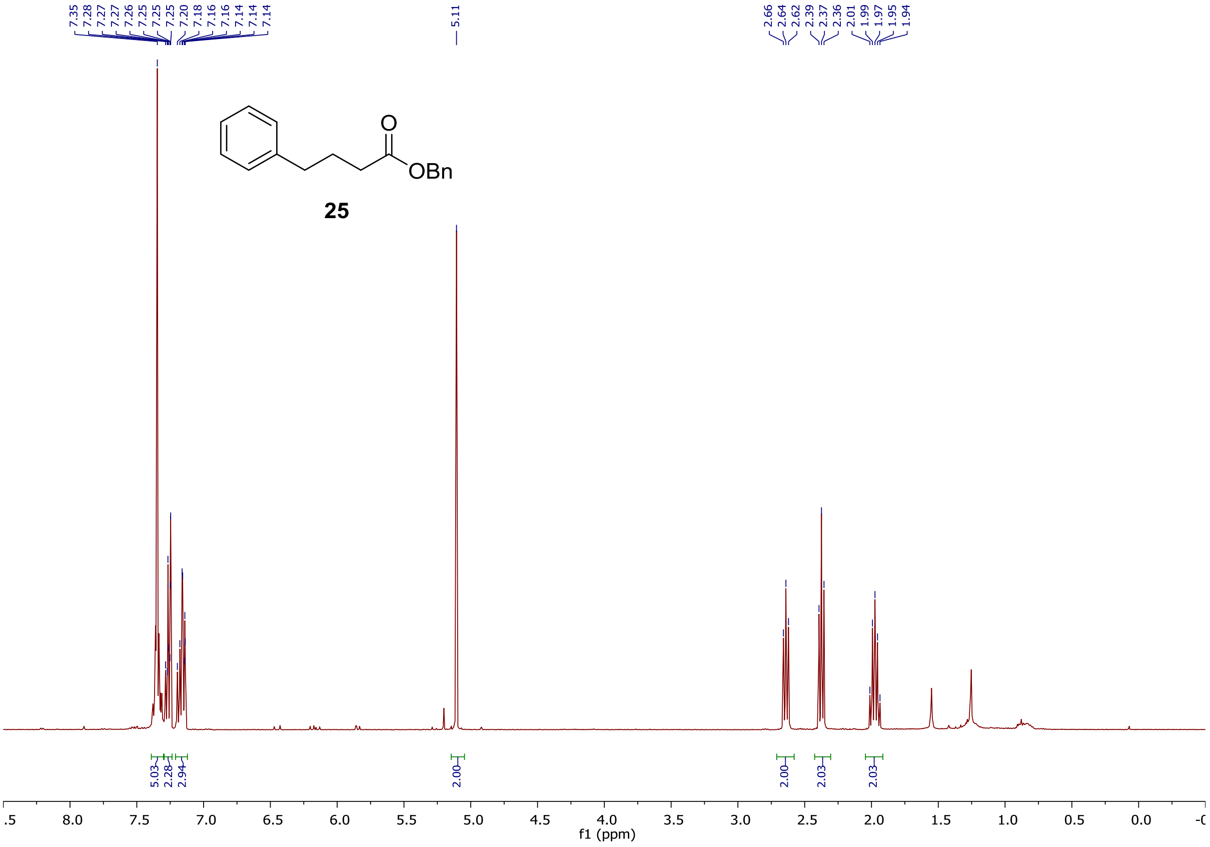

OBn
O
25
f1 (ppm)

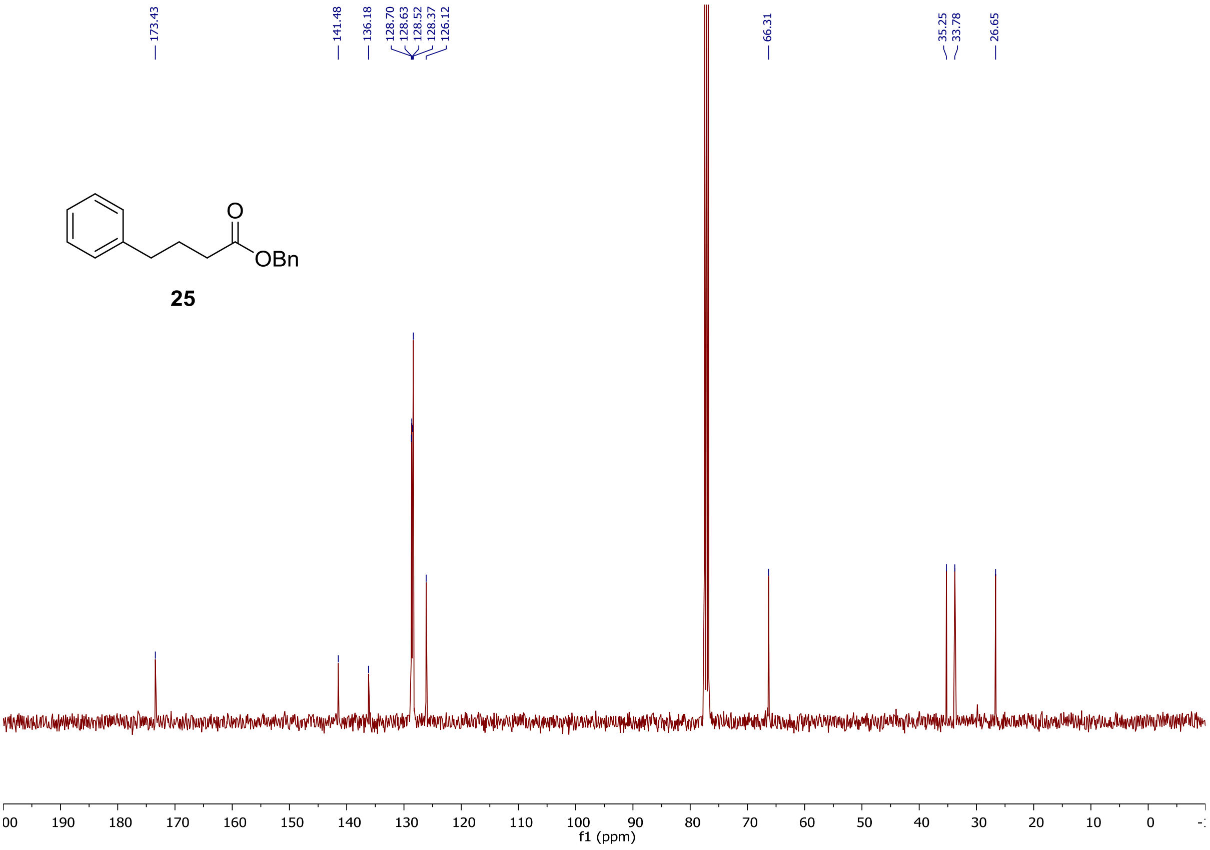

OBn
O
25
f1 (ppm)

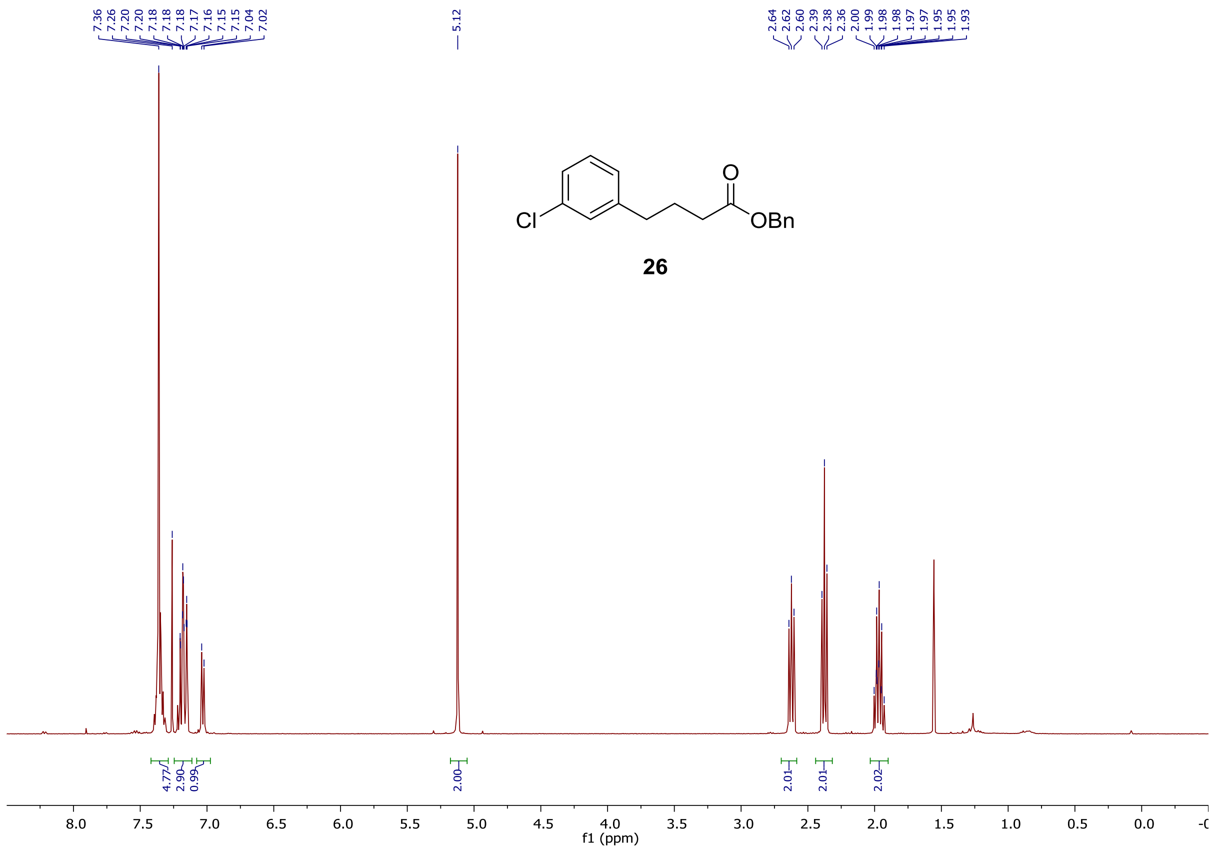
7.36 7.26 7.20 7.20 7.18 7.18 7.18 7.17 7.16 7.15 7.04 7.02
5.12
2.64 2.62 2.60 2.39 2.38 2.36 2.00 1.99 1.98 1.98 1.97 1.97 1.95 1.95 1.93
Cl
O
OBn
26
4.77 2.90 0.99
2.00
2.01 2.01 2.02
f1 (ppm)

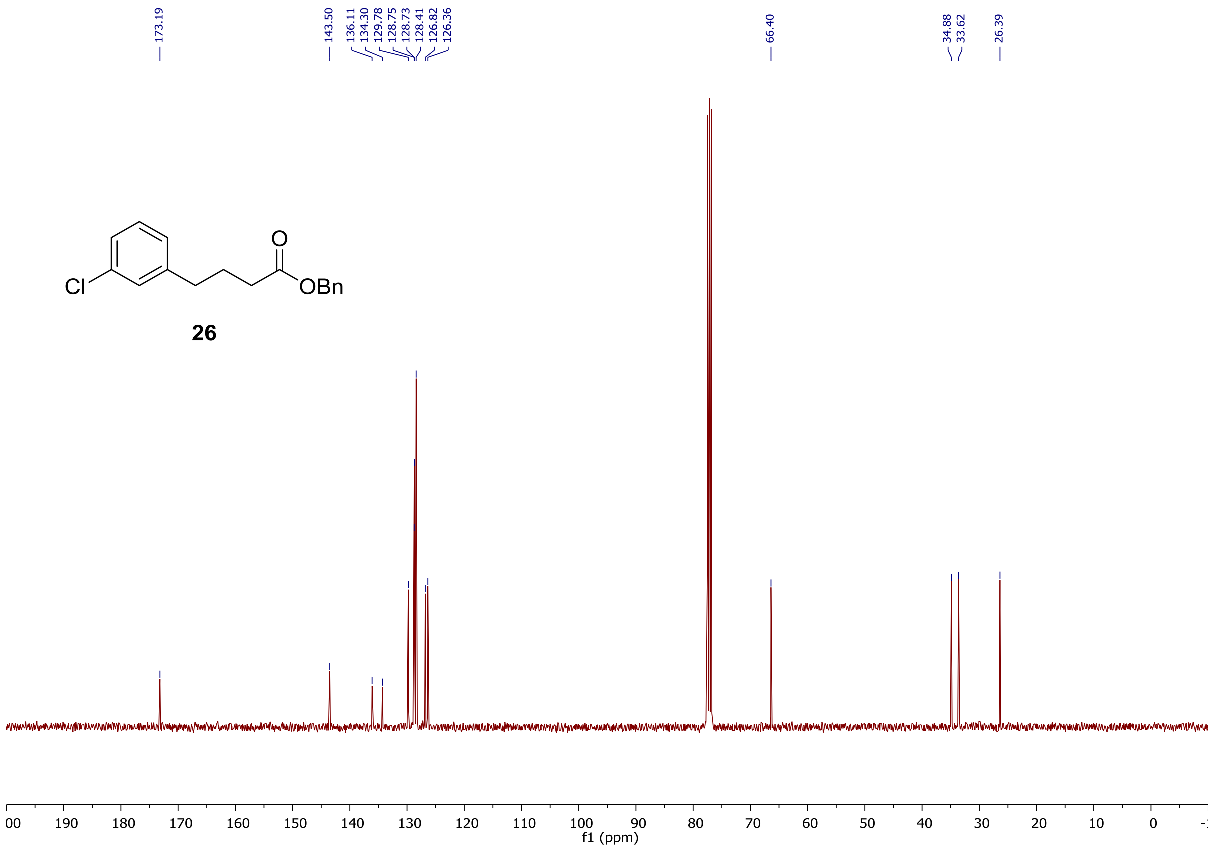
173.19
143.50 136.11 134.30 129.78 128.75 128.73 128.41 126.82 126.36
66.40
34.88 33.62
26.39
Cl
O
OBn
26
f1 (ppm)

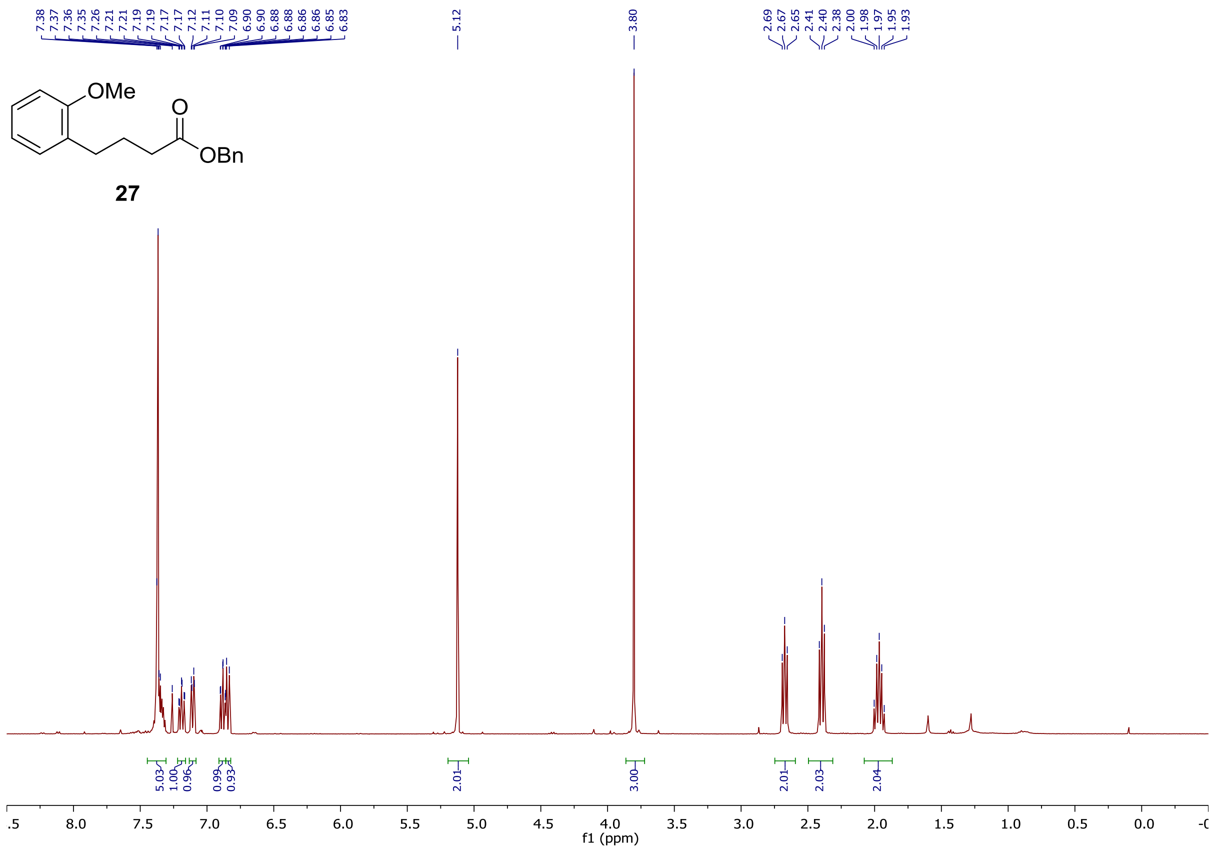

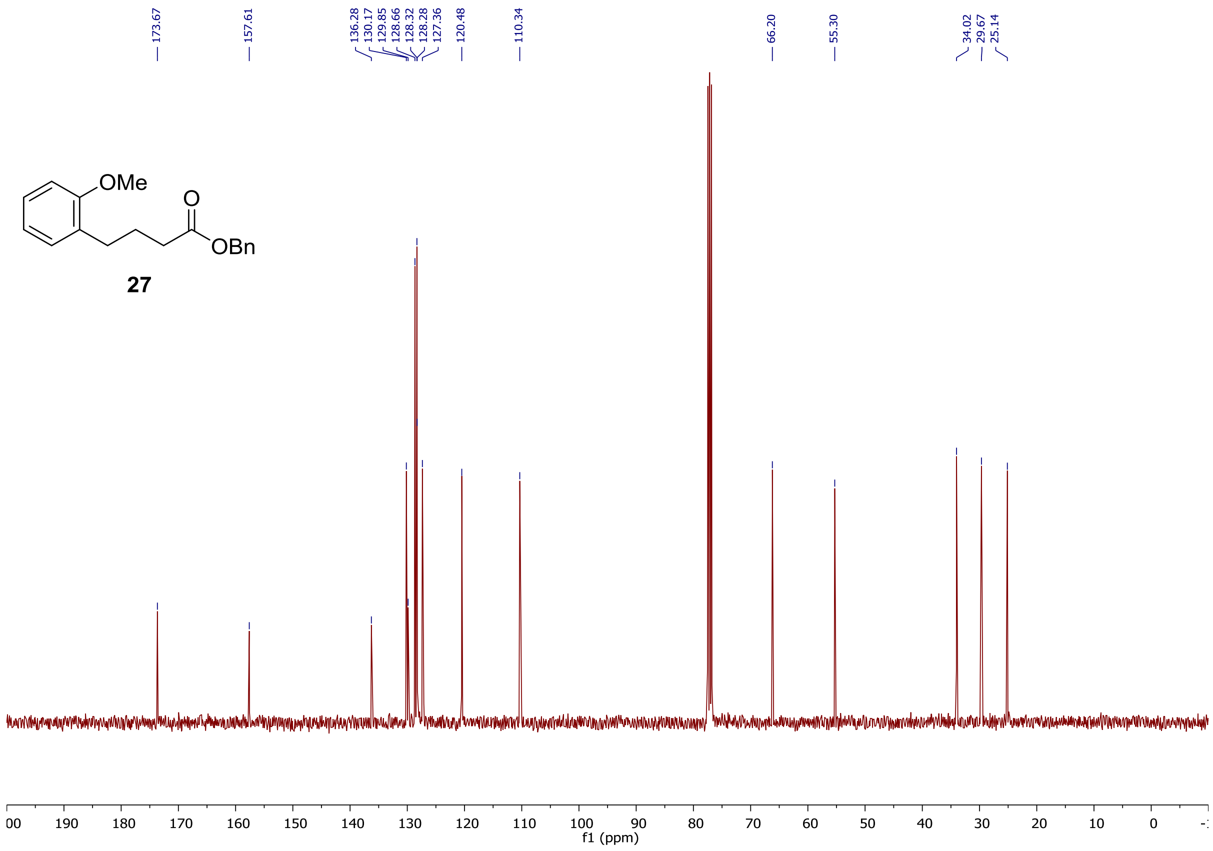

28

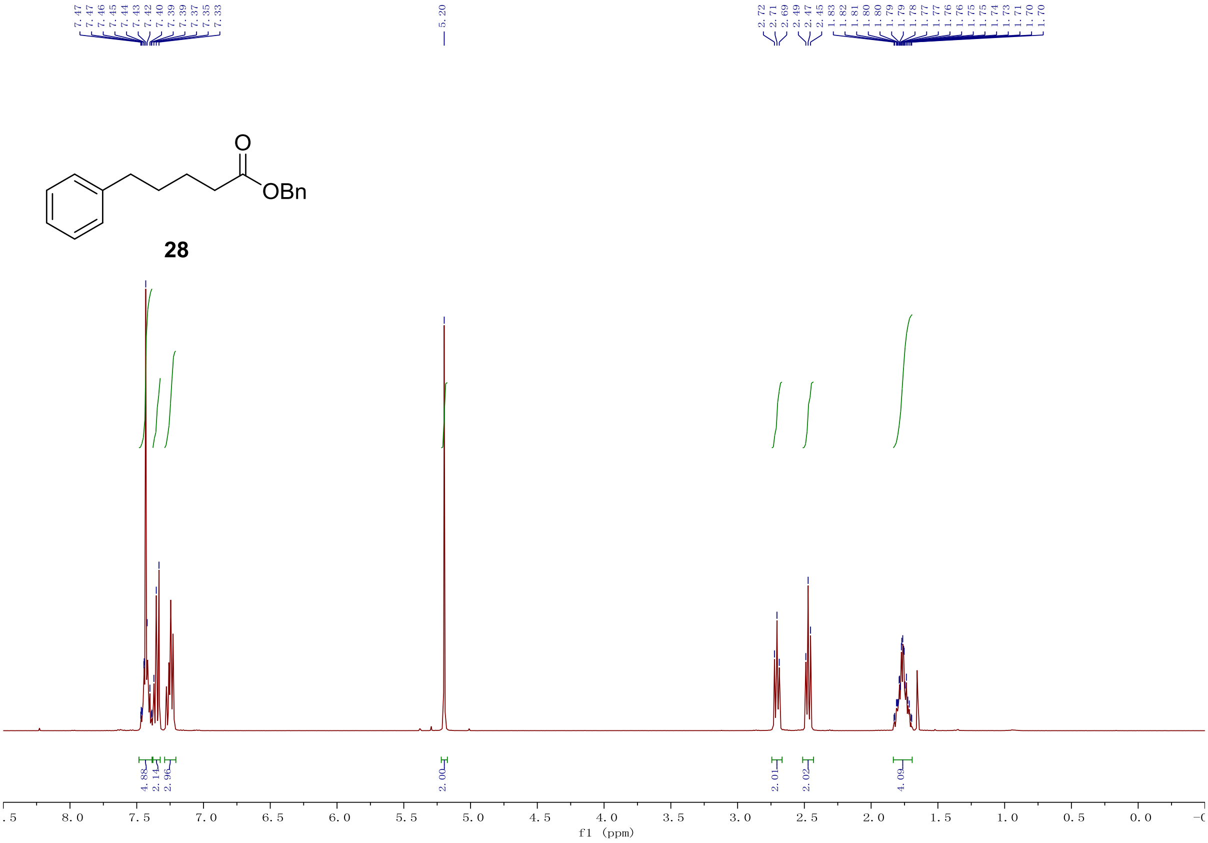

7.47 7.47 7.46 7.45 7.44 7.43 7.42 7.40 7.39 7.39 7.37 7.35 7.33
5.20
2.72 2.71 2.69 2.49 2.47 2.45 1.83 1.82 1.81 1.80 1.79 1.79 1.78 1.77 1.77 1.76 1.76 1.75 1.75 1.74 1.73 1.71 1.70 1.70
4.88 2.14 2.96 2.00 2.01 2.02 4.09
f1 (ppm)

28

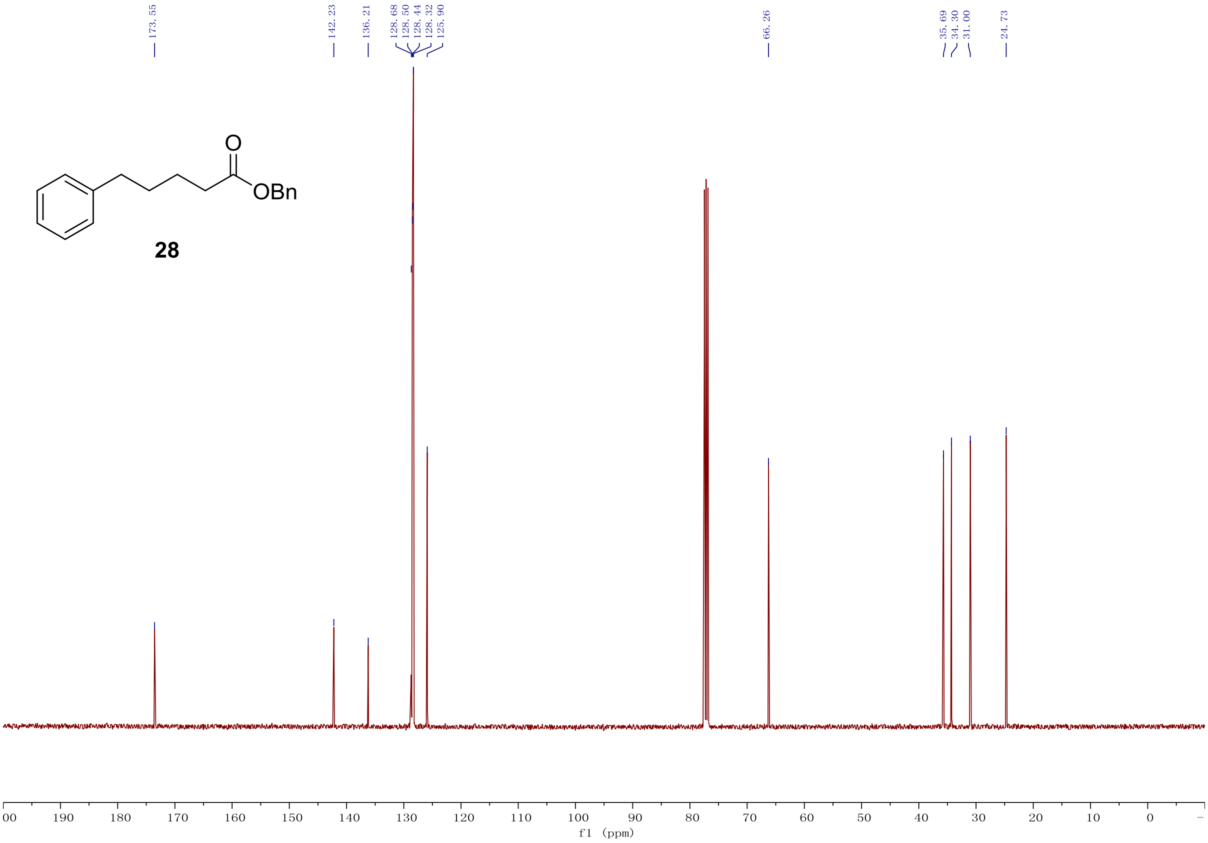

173.55
142.23
136.21
128.68 128.50 128.44 128.32 125.90
66.26
35.69 34.30 31.00
24.73
f1 (ppm)

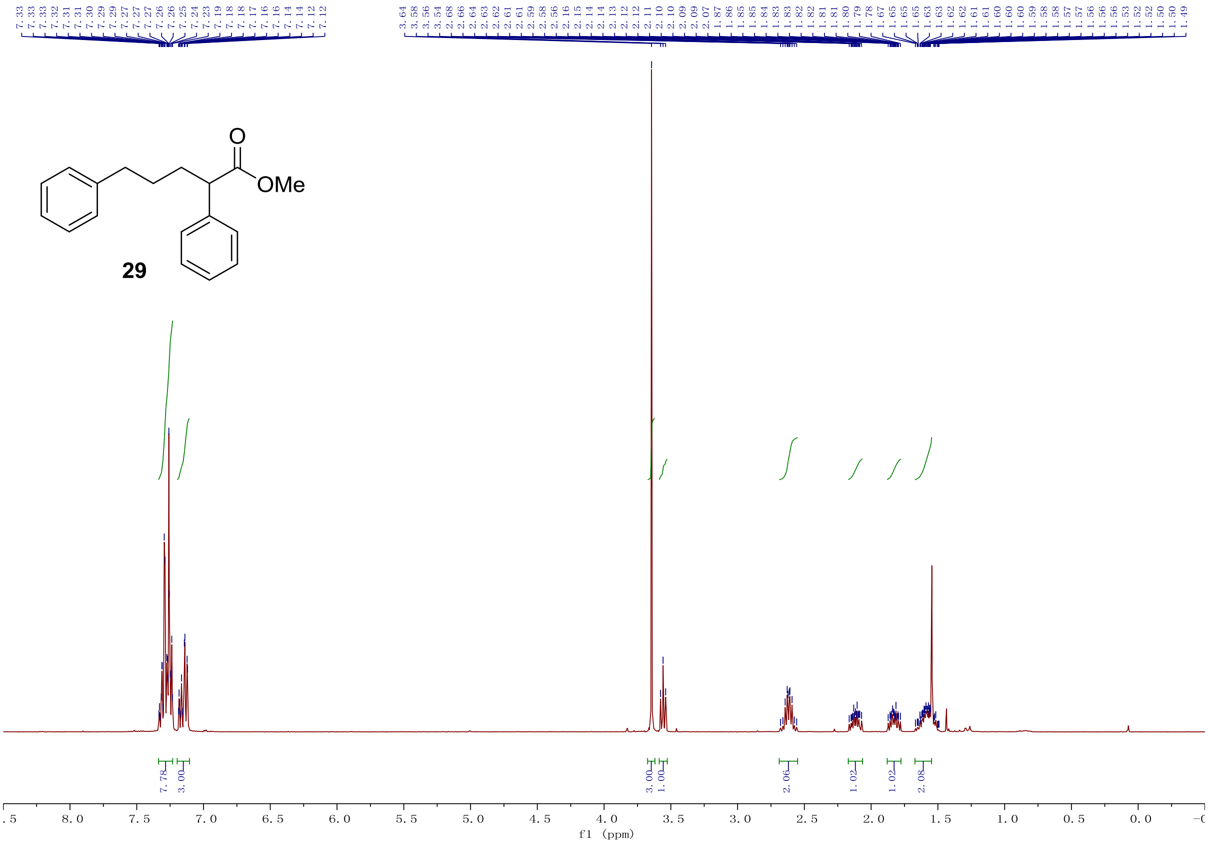

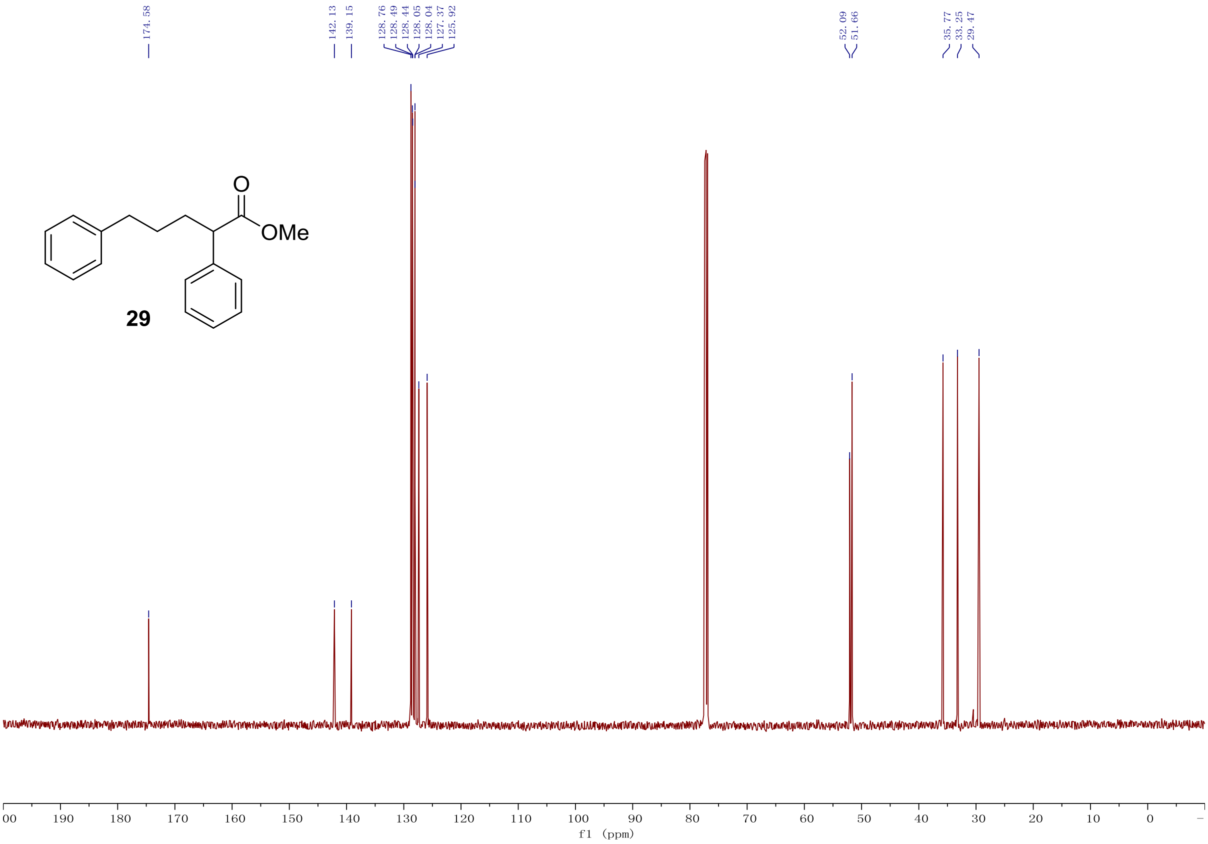

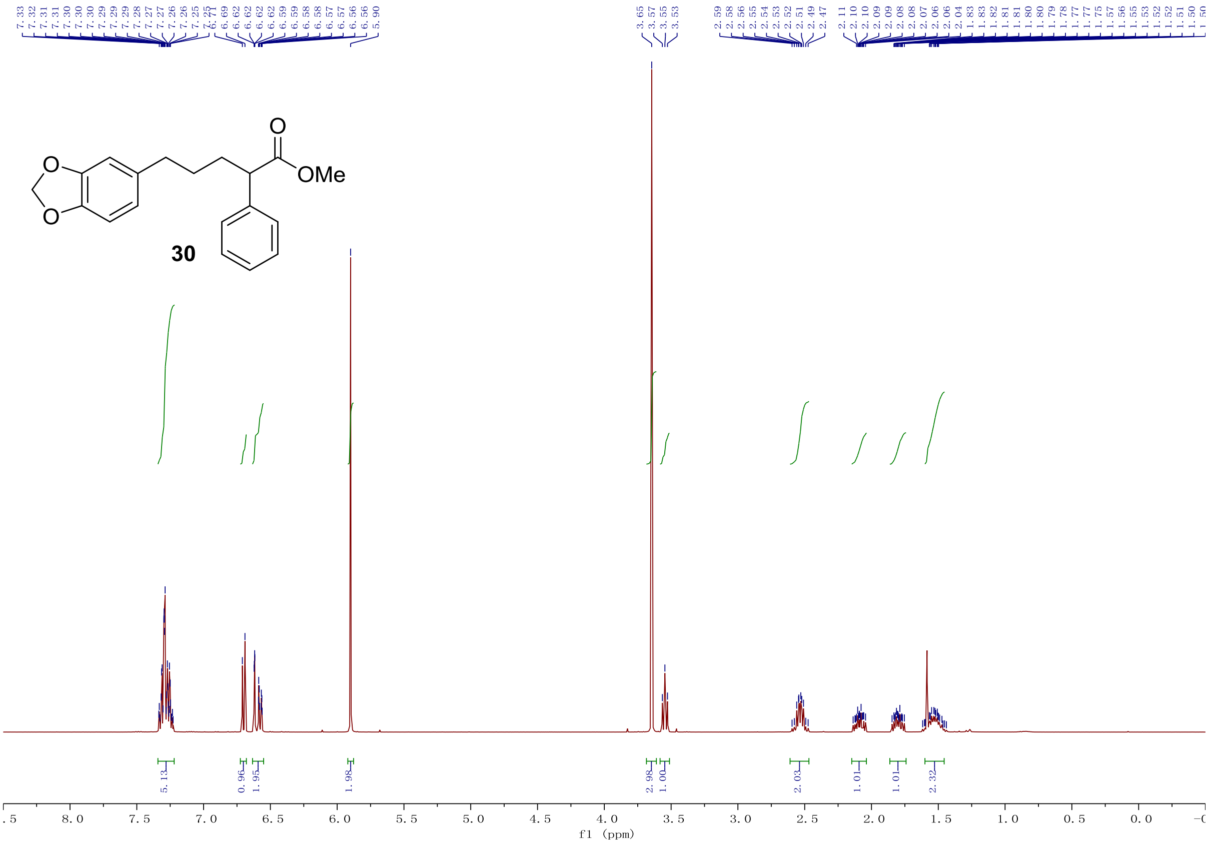

7.33 7.32 7.31 7.30 7.30 7.30 7.29 7.29 7.29 7.28 7.27 7.27 7.26 7.26 7.25 7.25 6.71 6.69 6.62 6.62 6.62 6.59 6.59 6.58 6.58 6.57 6.57 6.56 6.56 5.90
3.65 3.57 3.55 3.53
2.59 2.58 2.56 2.55 2.54 2.53 2.52 2.51 2.49 2.47
2.11 2.10 2.10 2.09 2.09 2.08 2.08 2.07 2.06 2.06 2.04 1.83 1.83 1.82 1.81 1.81 1.80 1.79 1.78 1.77 1.75 1.57 1.56 1.55 1.53 1.52 1.52 1.51 1.50
O
O
O
OMe
30
5.13 0.96 1.95 1.98 2.98 1.00 2.03 1.01 1.01 2.32
.5 8.0 7.5 7.0 6.5 6.0 5.5 5.0 4.5 4.0 3.5 3.0 2.5 2.0 1.5 1.0 0.5 0.0
f1 (ppm)

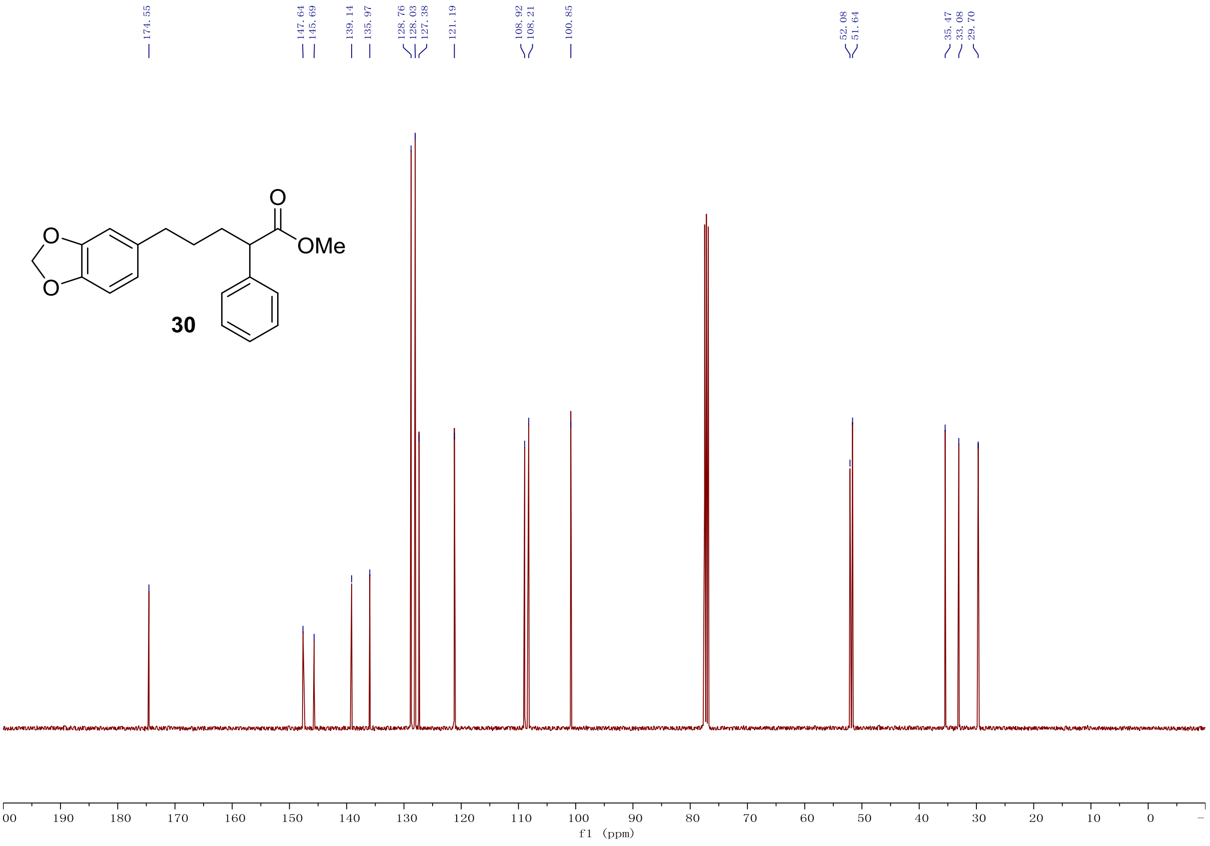

174.55
147.64 145.69
139.14 135.97
128.76 128.03 127.38
121.19
108.92 108.21
100.85
52.08 51.64
35.47 33.08 29.70
O
O
O
OMe
30
00 190 180 170 160 150 140 130 120 110 100 90 80 70 60 50 40 30 20 10 0
f1 (ppm)

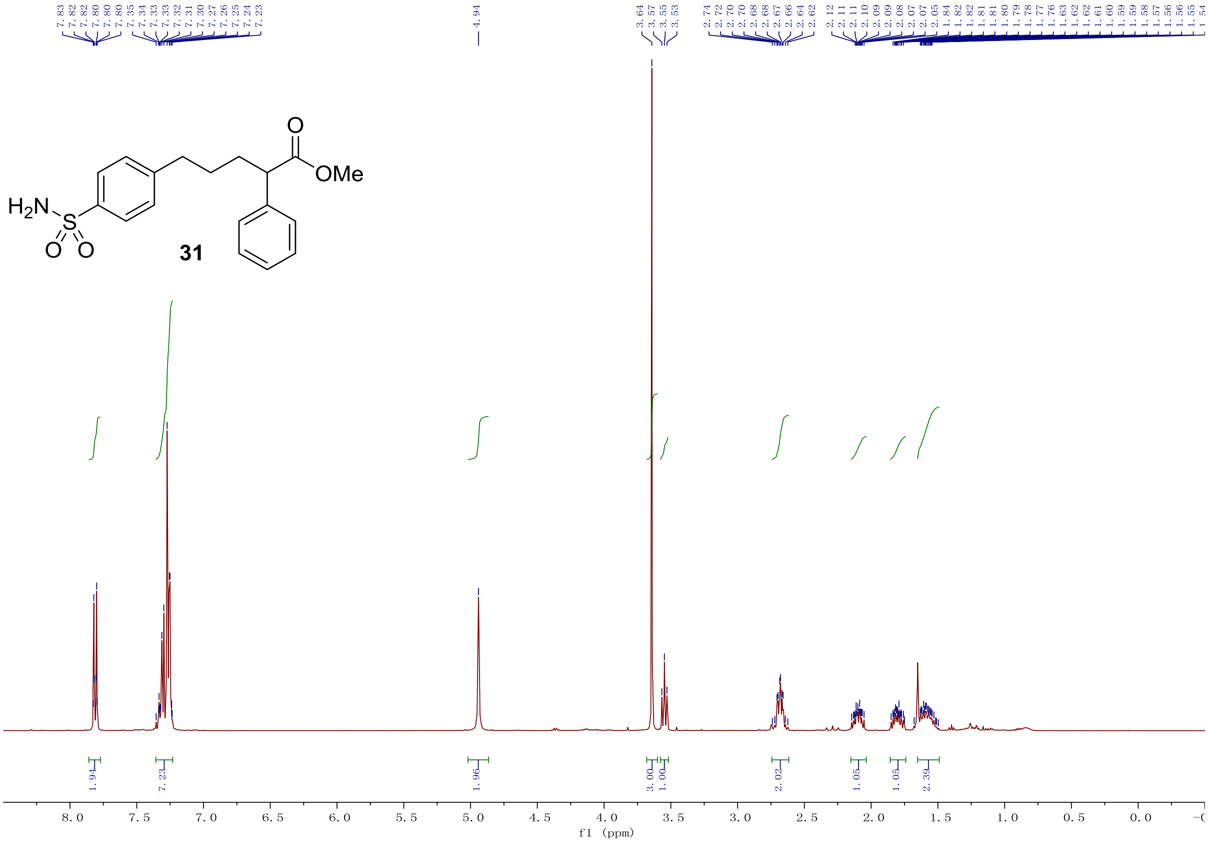
7.83
7.82
7.82
7.80
7.80
7.80
7.35
7.34
7.33
7.33
7.32
7.31
7.30
7.27
7.26
7.25
7.24
7.23
4.94
3.64
3.57
3.55
3.53
2.74
2.72
2.70
2.70
2.68
2.68
2.67
2.66
2.64
2.62
2.12
2.11
2.11
2.10
2.09
2.09
2.08
2.07
2.07
2.05
1.84
1.82
1.82
1.81
1.81
1.80
1.79
1.78
1.77
1.76
1.63
1.62
1.62
1.61
1.60
1.59
1.59
1.58
1.57
1.56
1.55
O
OMe
H_2N
S
O
O
31
1.94
7.23
1.96
3.00
1.00
2.02
1.05
1.05
2.39
8.0
7.5
7.0
6.5
6.0
5.5
5.0
4.5
4.0
3.5
3.0
2.5
2.0
1.5
1.0
0.5
0.0
f1 (ppm)

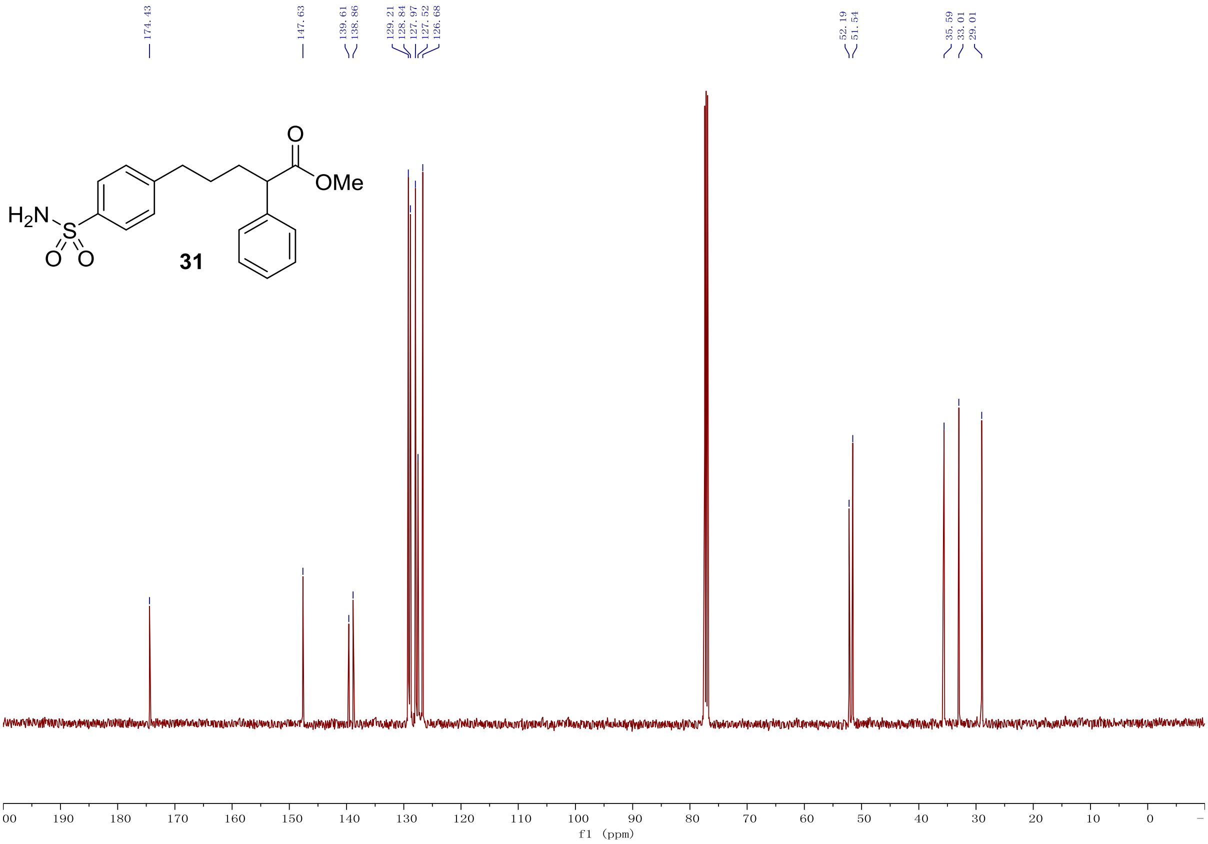
174.43
147.63
139.61
138.86
129.21
128.84
127.97
127.52
126.68
52.19
51.54
35.59
33.01
29.01
O
OMe
H_2N
S
O
O
31
190
180
170
160
150
140
130
120
110
100
90
80
70
60
50
40
30
20
10
0
f1 (ppm)

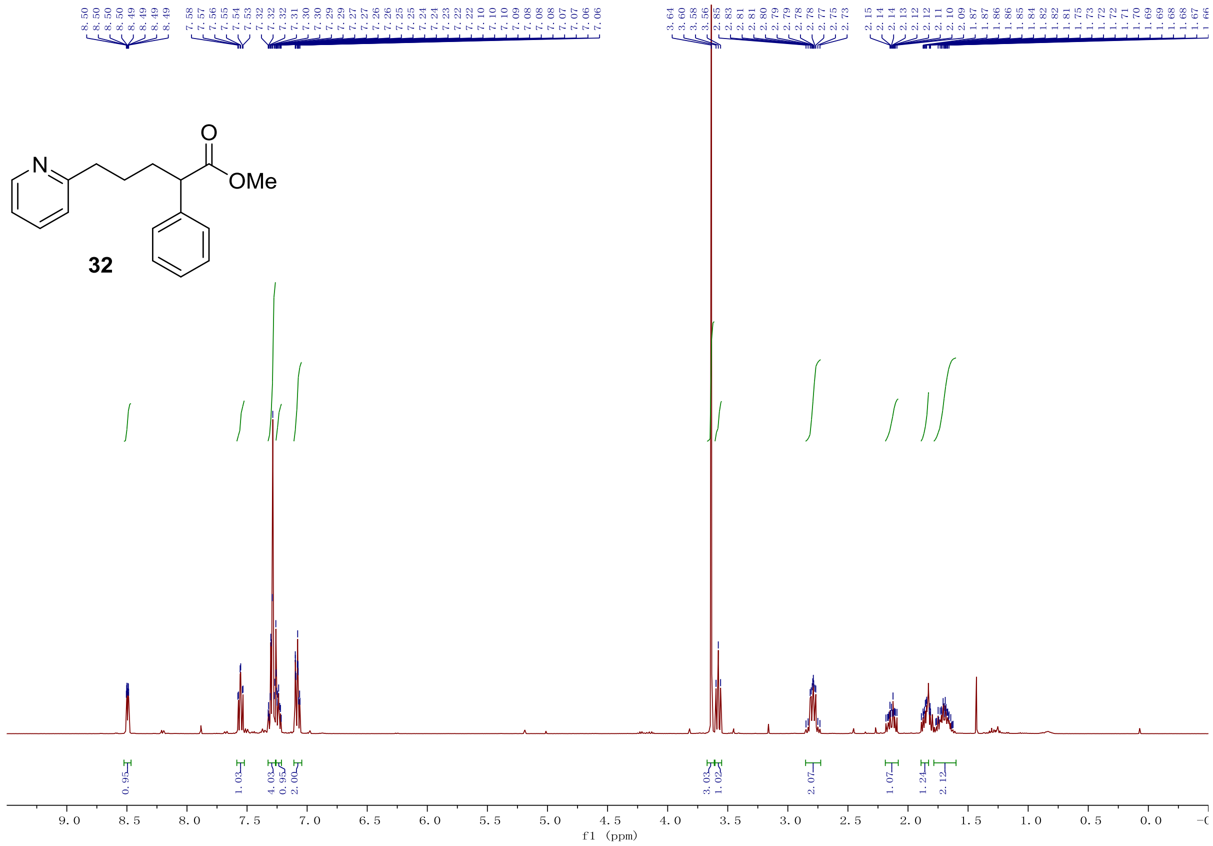
8.50
8.50
8.50
8.49
8.49
8.49
8.49
7.58
7.57
7.56
7.55
7.54
7.53
3.64
3.60
3.58
3.56
OMe
32
0.95
1.03
4.03
0.95
2.00
3.03
1.02
2.07
1.07
1.24
2.12
f1 (ppm)

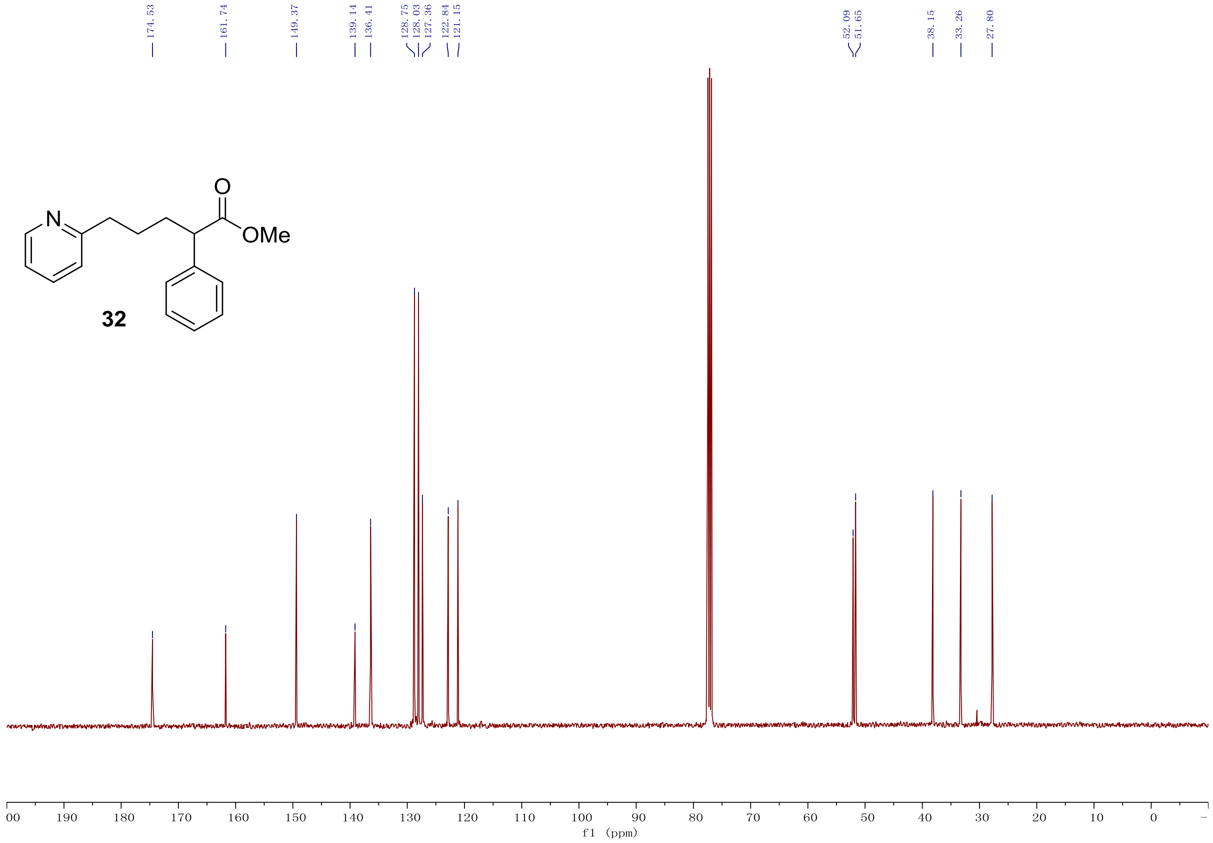
174.53
161.74
149.37
139.14
136.41
128.75
128.03
127.36
122.84
121.15
52.09
51.65
38.15
33.26
27.80
OMe
32
f1 (ppm)

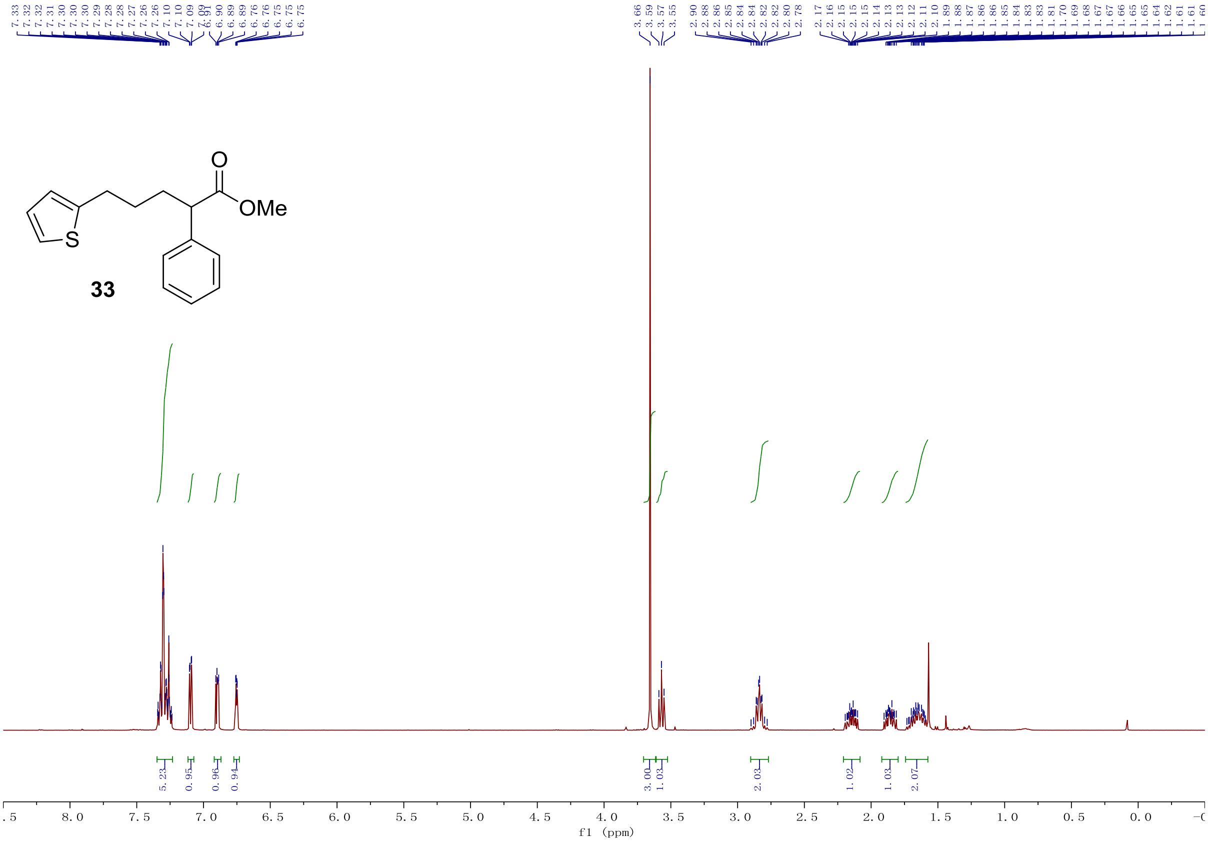
7.33 7.32 7.31 7.30 7.30 7.29 7.28 7.28 7.27 7.26 7.26 7.10 7.10 7.09 7.09 6.91 6.90 6.89 6.89 6.76 6.76 6.75 6.75
3.66 3.59 3.57 3.55
2.90 2.88 2.86 2.85 2.84 2.84 2.82 2.82 2.80 2.78
2.17 2.16 2.15 2.15 2.14 2.13 2.13 2.12 2.11 2.10 1.89 1.88 1.87 1.86 1.86 1.85 1.84 1.83 1.81 1.70 1.69 1.68 1.67 1.67 1.66 1.65 1.64 1.62 1.61 1.60
O
OMe
S
33
5.23
0.95
0.96
0.94
3.00
1.03
2.03
1.02
1.03
2.07
f1 (ppm)

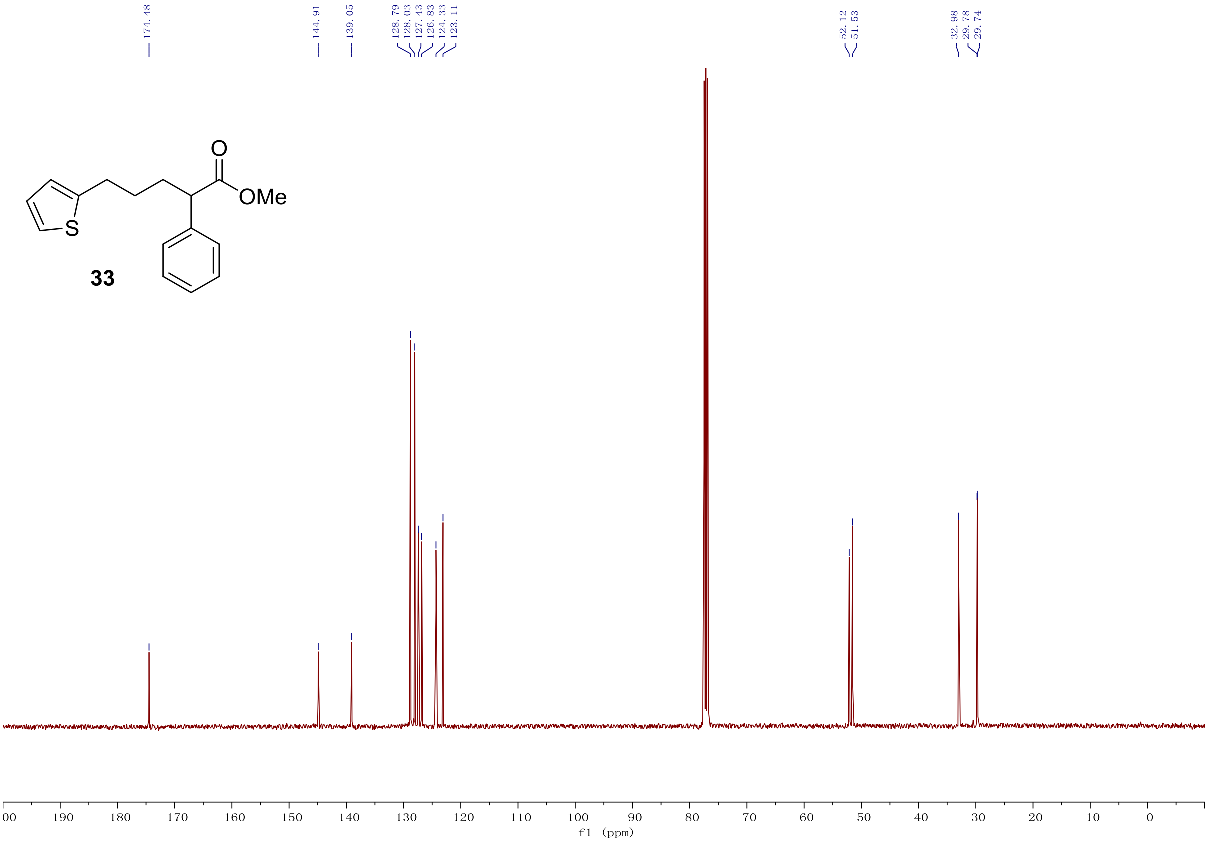
174.48
144.91
139.05
128.79 128.03 127.43 126.83 124.33 123.11
52.12 51.53
32.98 29.78 29.74
O
OMe
S
33
f1 (ppm)

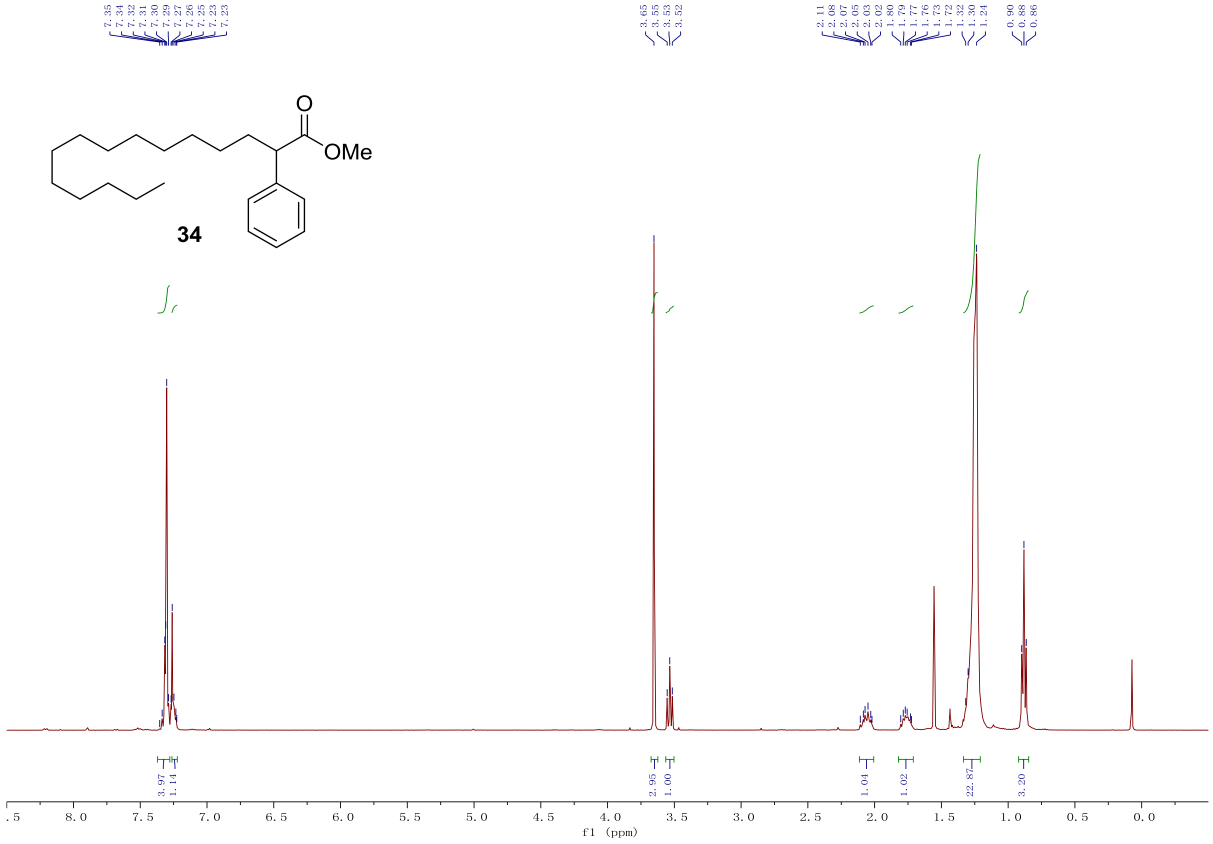
7.35 7.34 7.32 7.31 7.30 7.29 7.27 7.26 7.25 7.23 7.23
3.65 3.55 3.53 3.52
2.11 2.08 2.07 2.05 2.03 2.02 1.80 1.79 1.77 1.76 1.73 1.72 1.32 1.30 1.24
0.90 0.88 0.86
O
OMe
34
3.97 1.14 2.95 1.00 1.04 1.02 22.87 3.20
f1 (ppm)

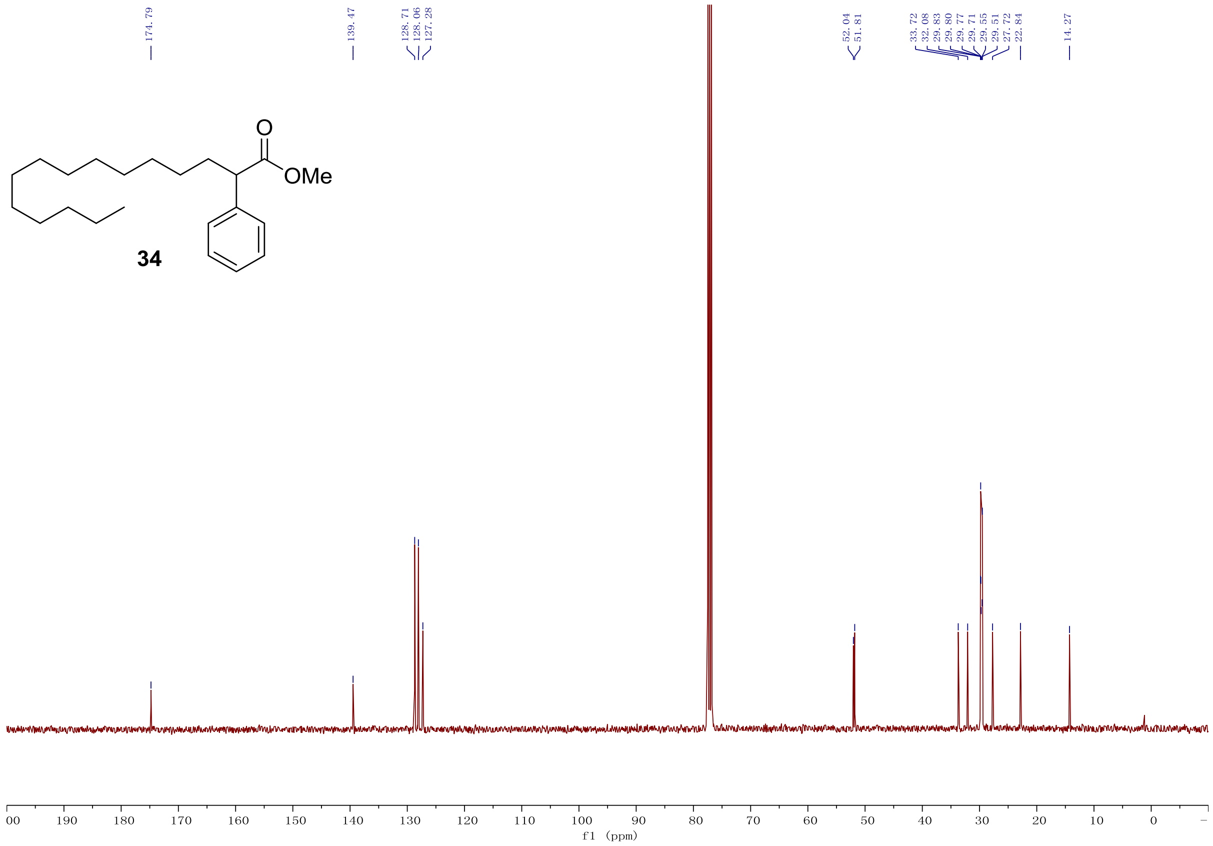
174.79
139.47
128.71 128.06 127.28
52.04 51.81
33.72 32.08 29.83 29.80 29.77 29.71 29.55 29.51 27.72 22.84
14.27
O
OMe
34
f1 (ppm)

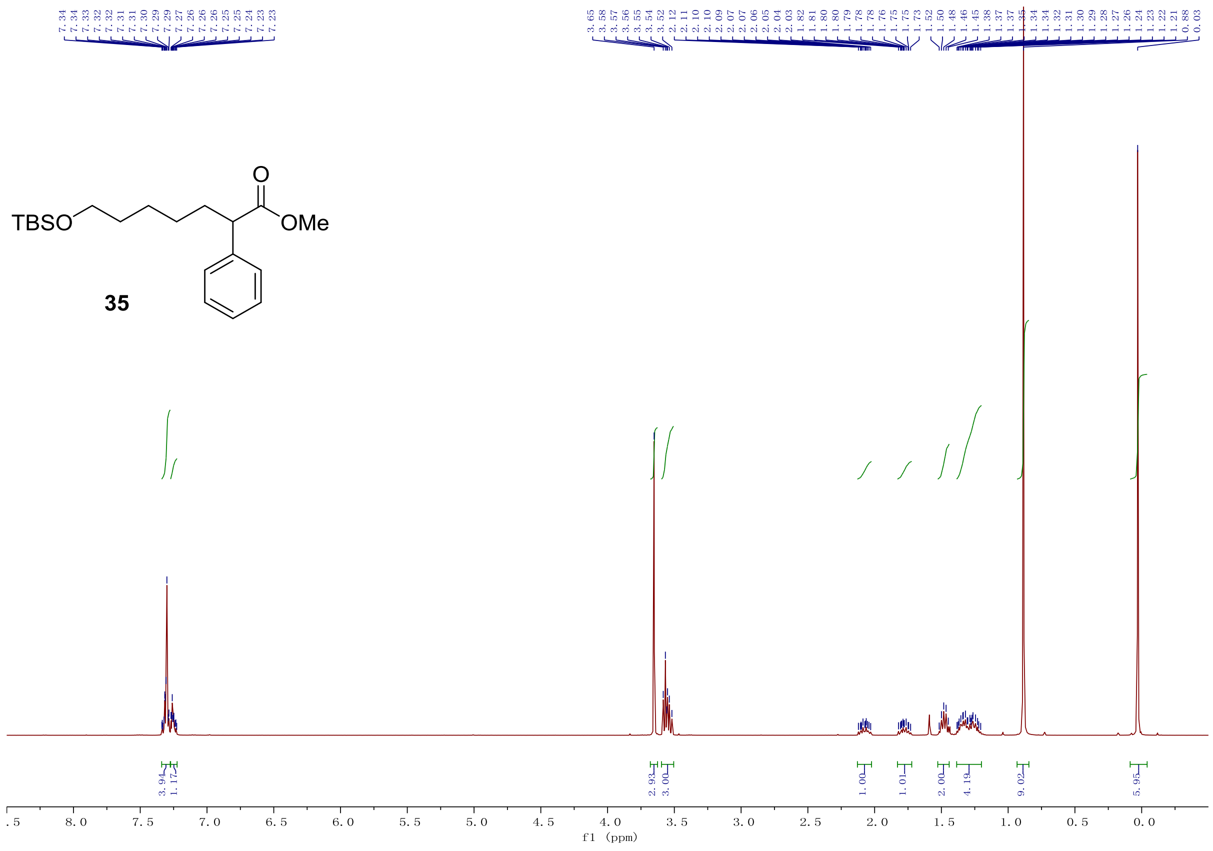

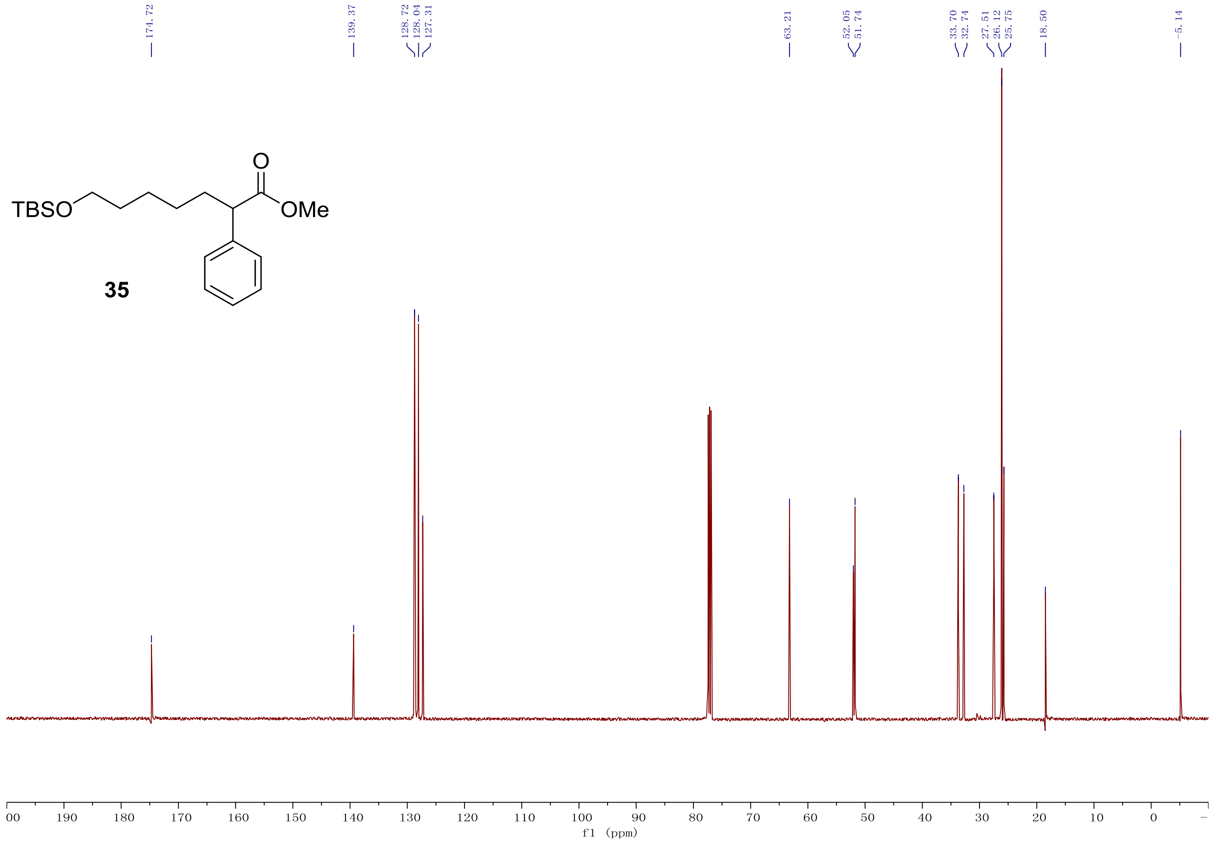

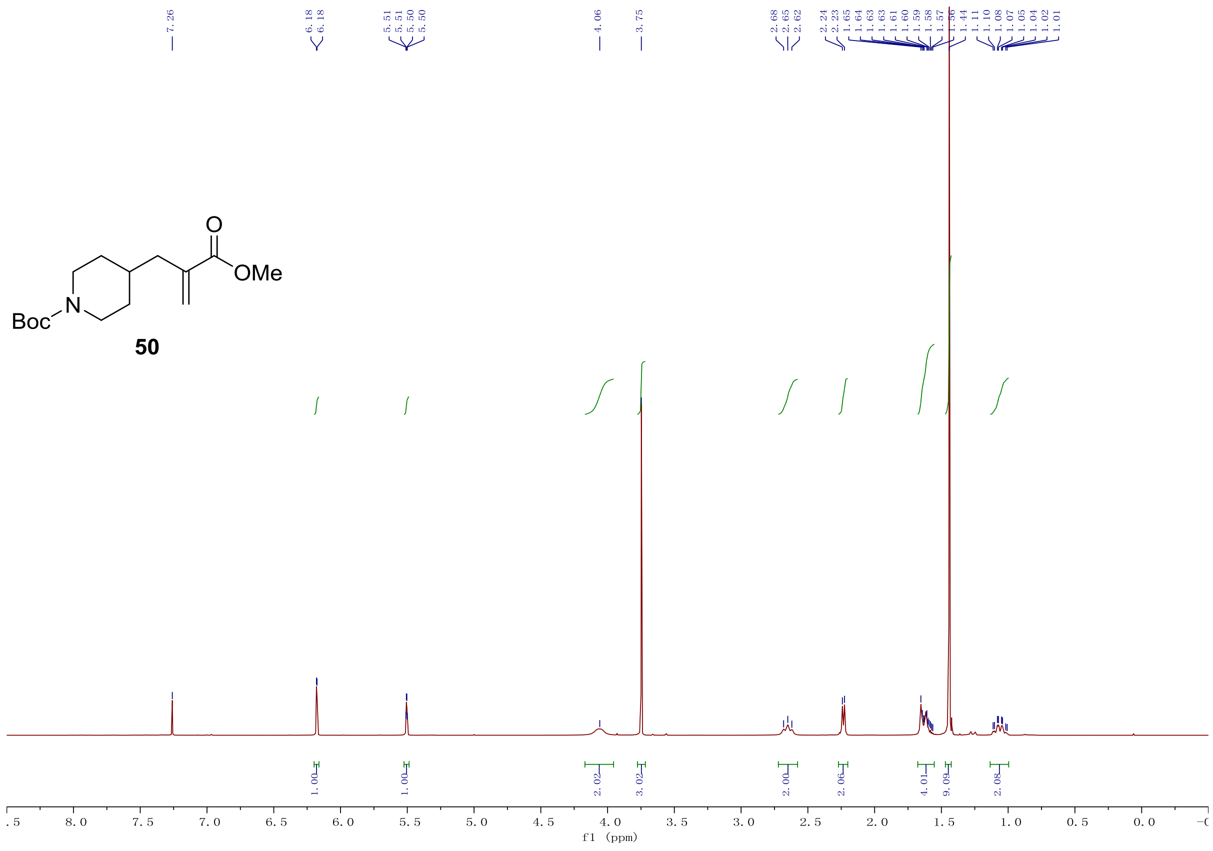
7.26
6.18
6.18
5.51
5.51
5.50
5.50
4.06
3.75
2.68
2.65
2.62
2.24
2.23
1.65
1.64
1.63
1.61
1.60
1.59
1.58
1.57
1.56
1.44
1.11
1.10
1.08
1.07
1.05
1.04
1.02
1.01
1.00
1.00
2.02
3.02
2.00
2.06
4.01
9.09
2.08
8.0
7.5
7.0
6.5
6.0
5.5
5.0
4.5
4.0
3.5
3.0
2.5
2.0
1.5
1.0
0.5
0.0
f1 (ppm)
O
OMe
N
Boc
50

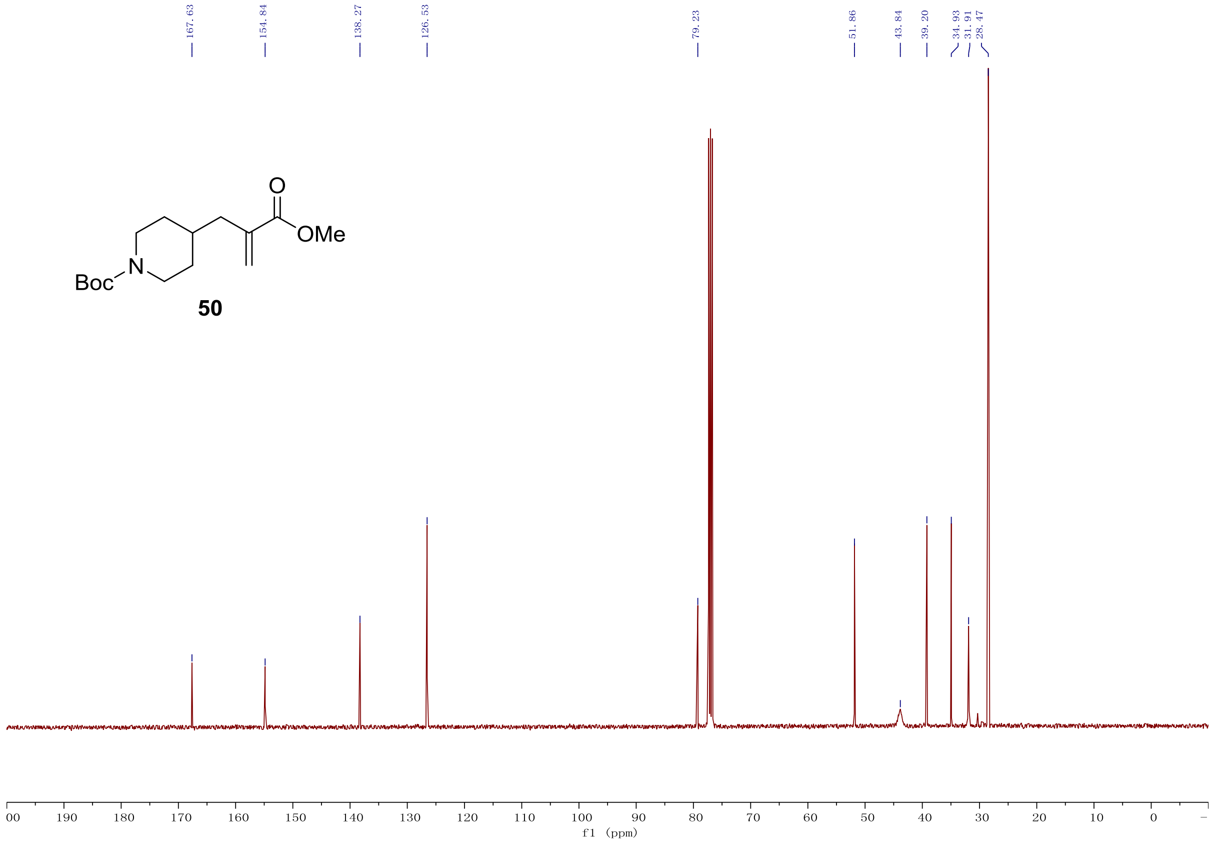
167.63
154.84
138.27
126.53
79.23
51.86
43.84
39.20
34.93
31.91
28.47
190
180
170
160
150
140
130
120
110
100
90
80
70
60
50
40
30
20
10
0
f1 (ppm)
O
OMe
N
Boc
50

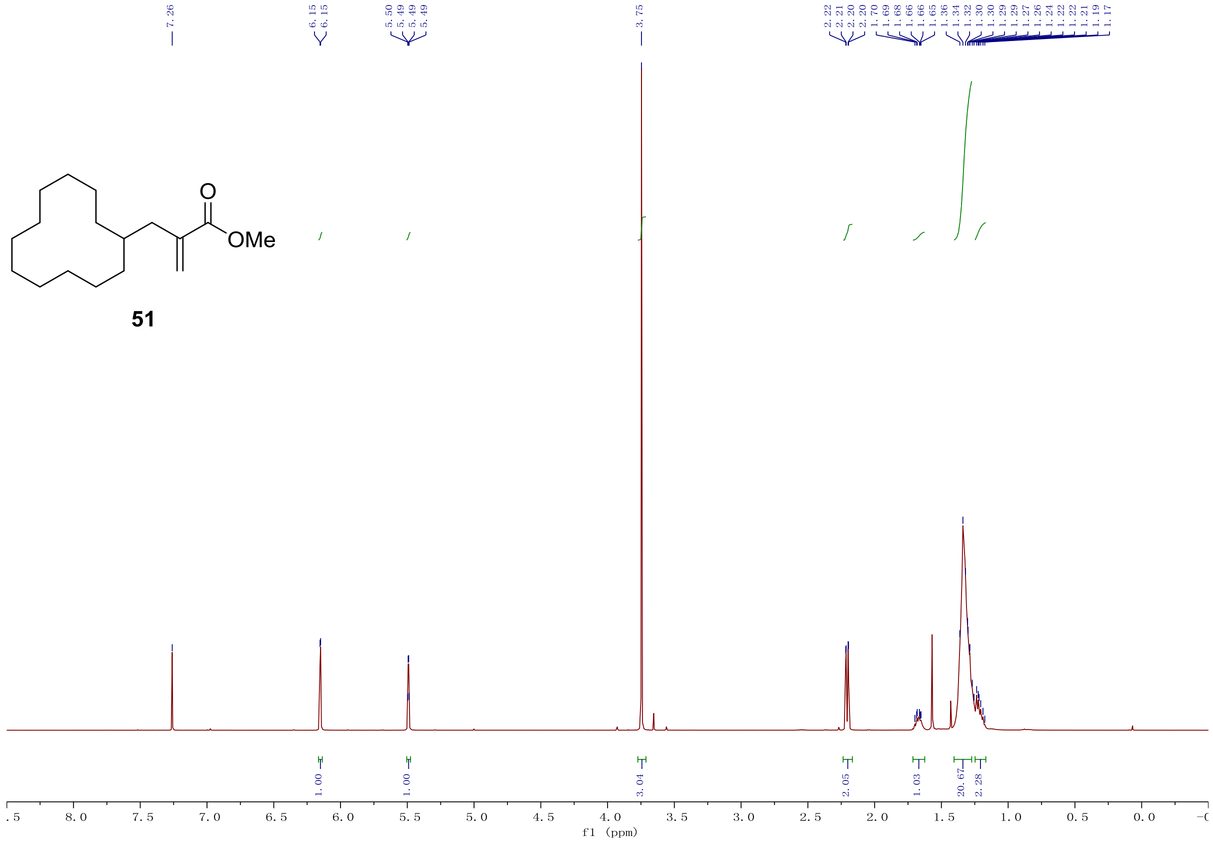

7.26
6.15
6.15
5.50
5.49
5.49
5.49
3.75
2.22
2.21
2.20
2.20
1.70
1.69
1.68
1.66
1.66
1.65
1.36
1.34
1.32
1.30
1.30
1.29
1.29
1.27
1.26
1.24
1.22
1.22
1.21
1.19
1.17
O
OMe
1.00
1.00
3.04
2.05
1.03
20.67
2.28
f1 (ppm)

51

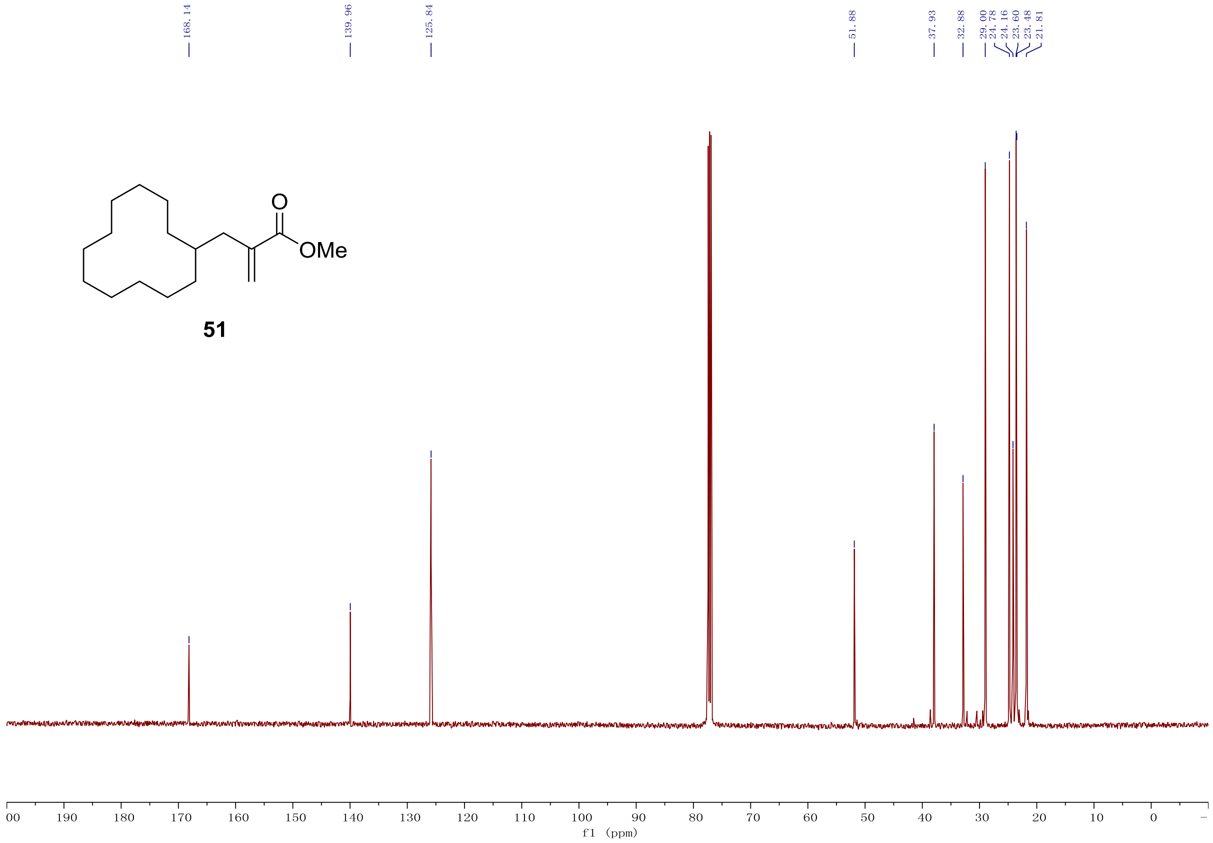

168.14
139.96
125.84
51.88
37.93
32.88
29.00
24.78
24.16
23.60
23.48
21.81
O
OMe
f1 (ppm)

51

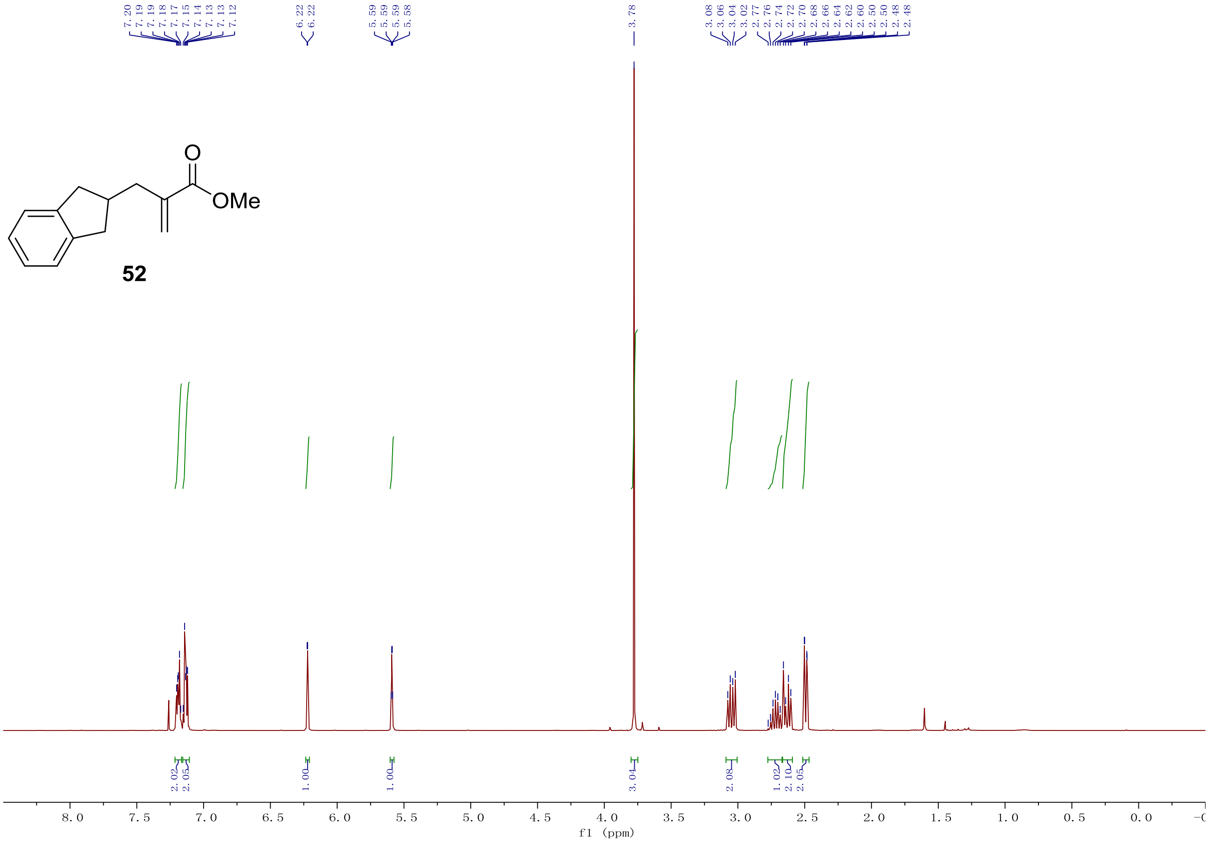
7.20 7.19 7.18 7.17 7.15 7.14 7.13 7.13 7.12
6.22 6.22
5.59 5.59 5.59 5.58
3.78
3.08 3.06 3.04 3.02 2.77 2.76 2.74 2.72 2.70 2.68 2.66 2.64 2.62 2.60 2.50 2.48
O
OMe
52
2.02 2.05 1.00 1.00 3.04 2.08 1.02 2.10 2.05
f1 (ppm)

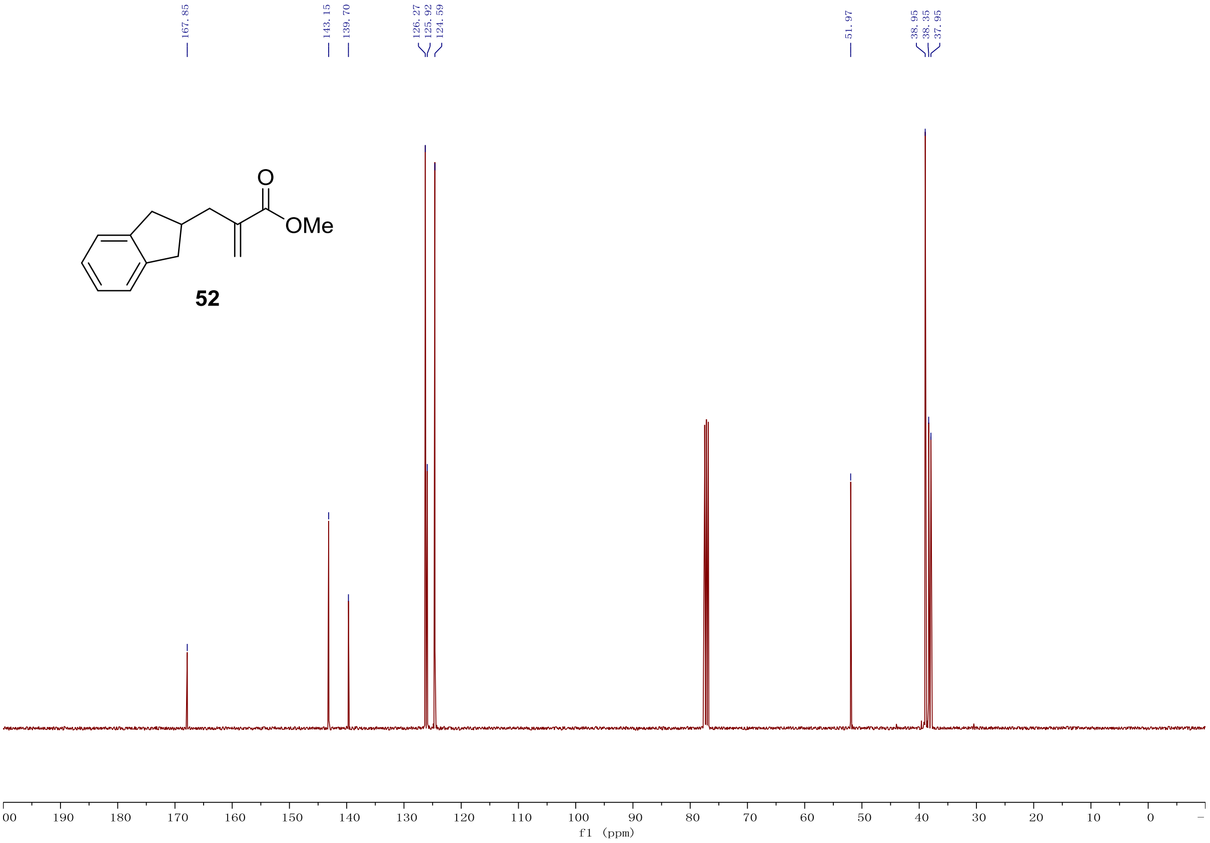
167.85
143.15 139.70
126.27 125.92 124.59
51.97
38.95 38.35 37.95
O
OMe
52
f1 (ppm)

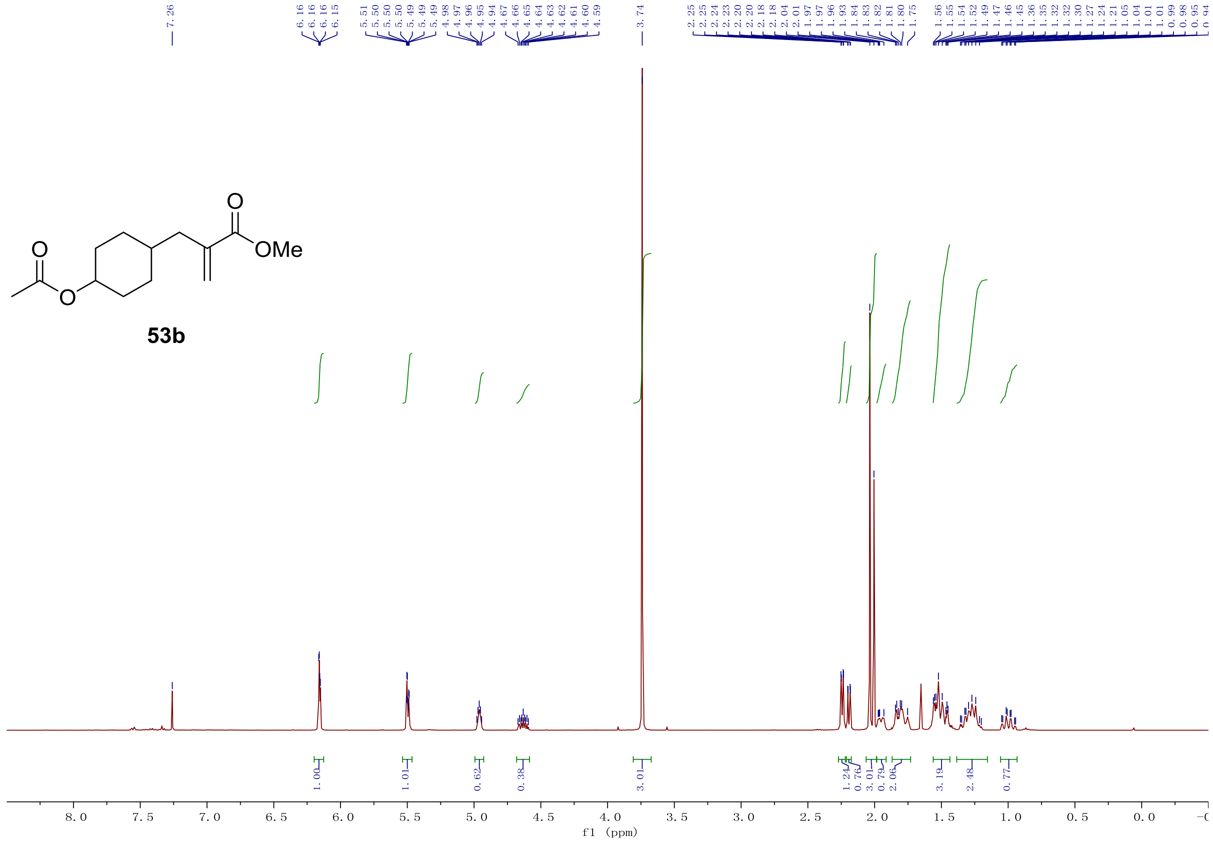

53b

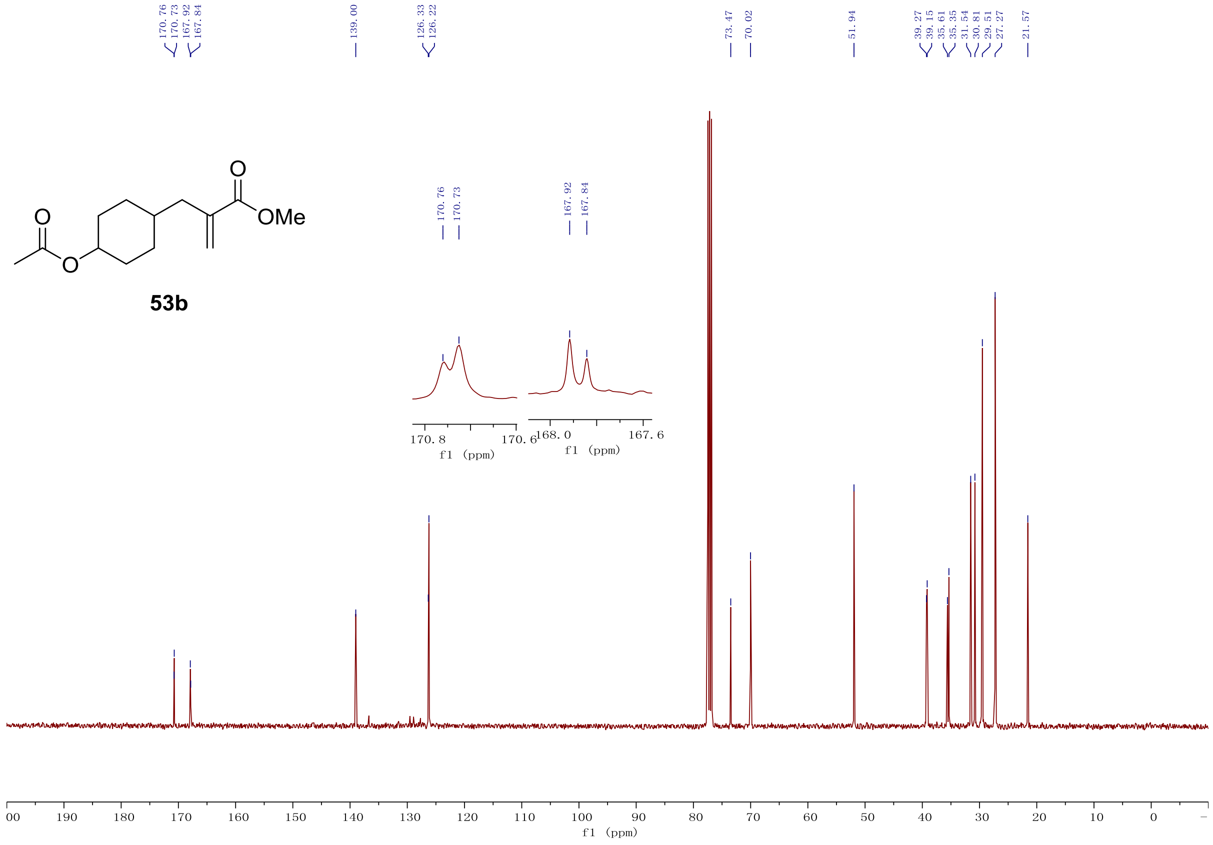

53b

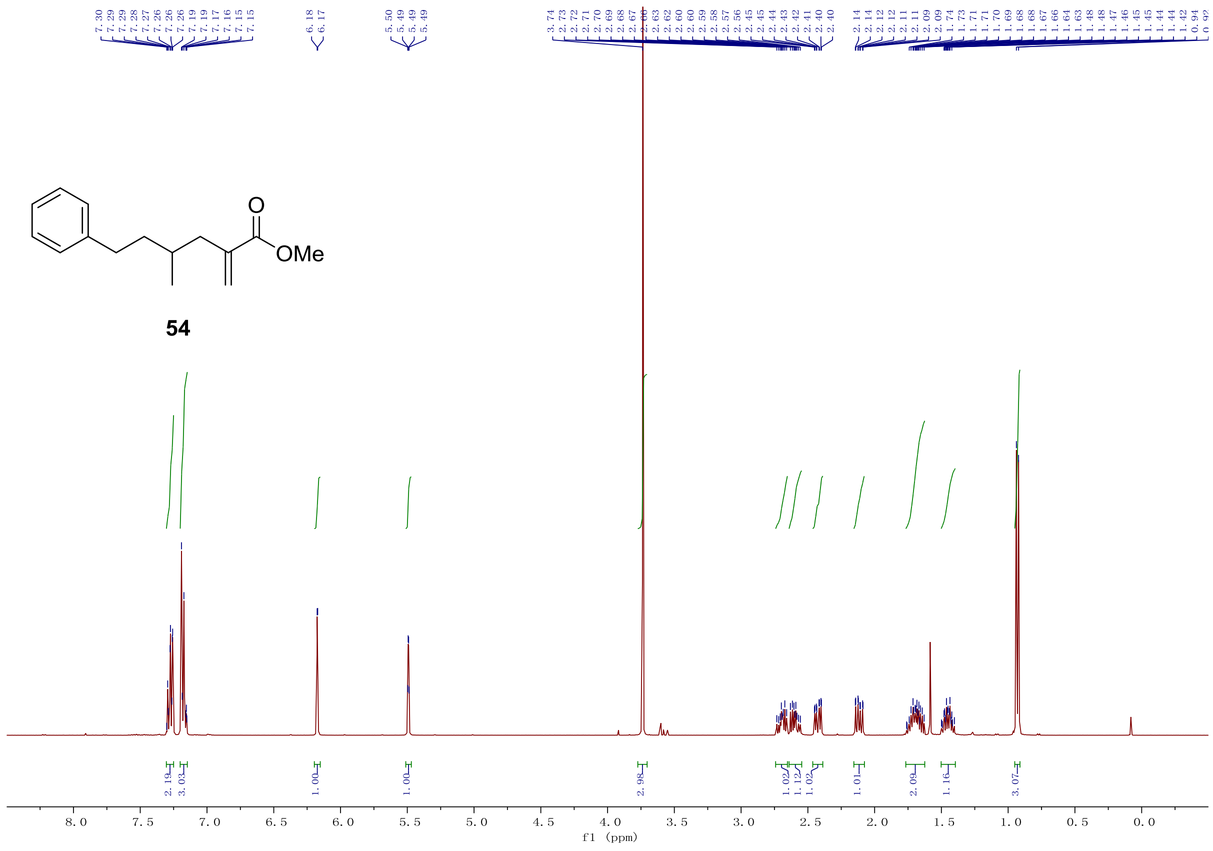
O
OMe
54
f1 (ppm)

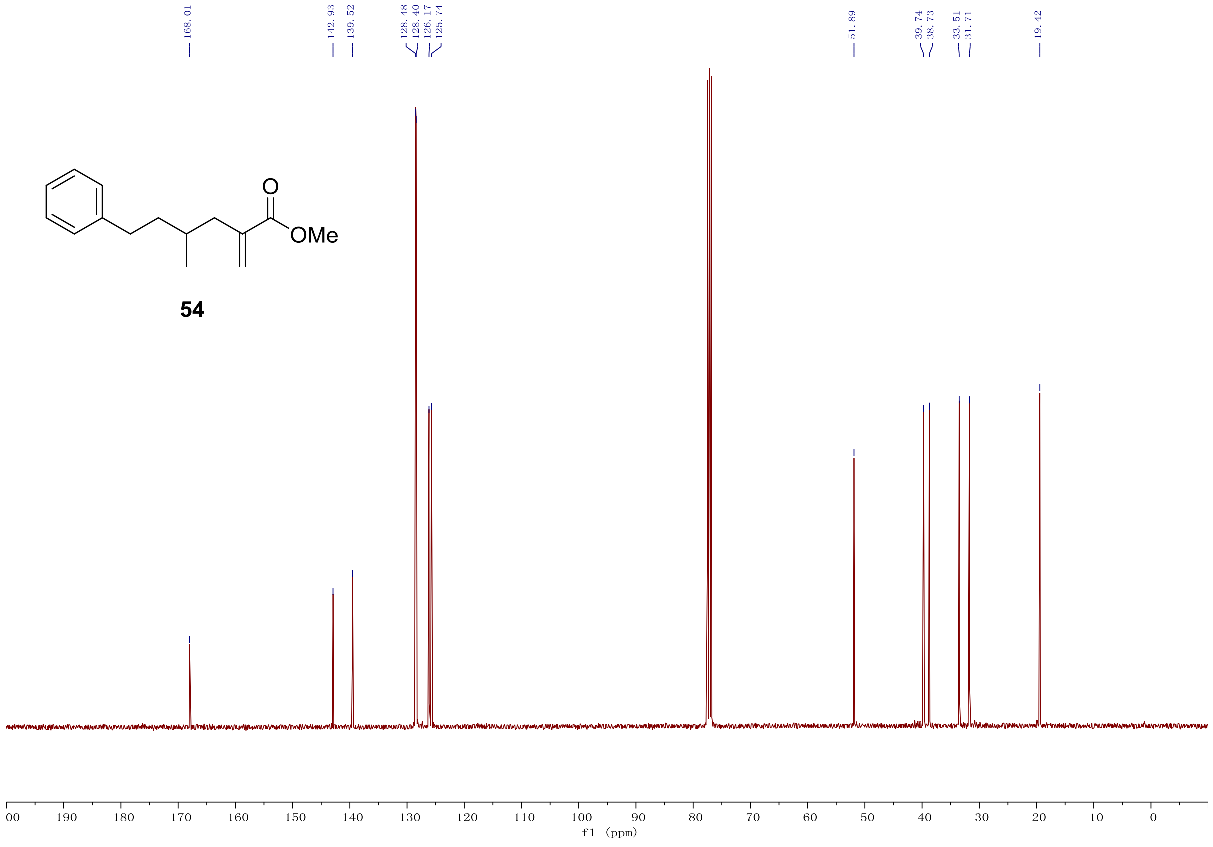
168.01
142.93
139.52
128.48
128.40
126.17
125.74
51.89
39.74
38.73
33.51
31.71
19.42
O
OMe
54
f1 (ppm)

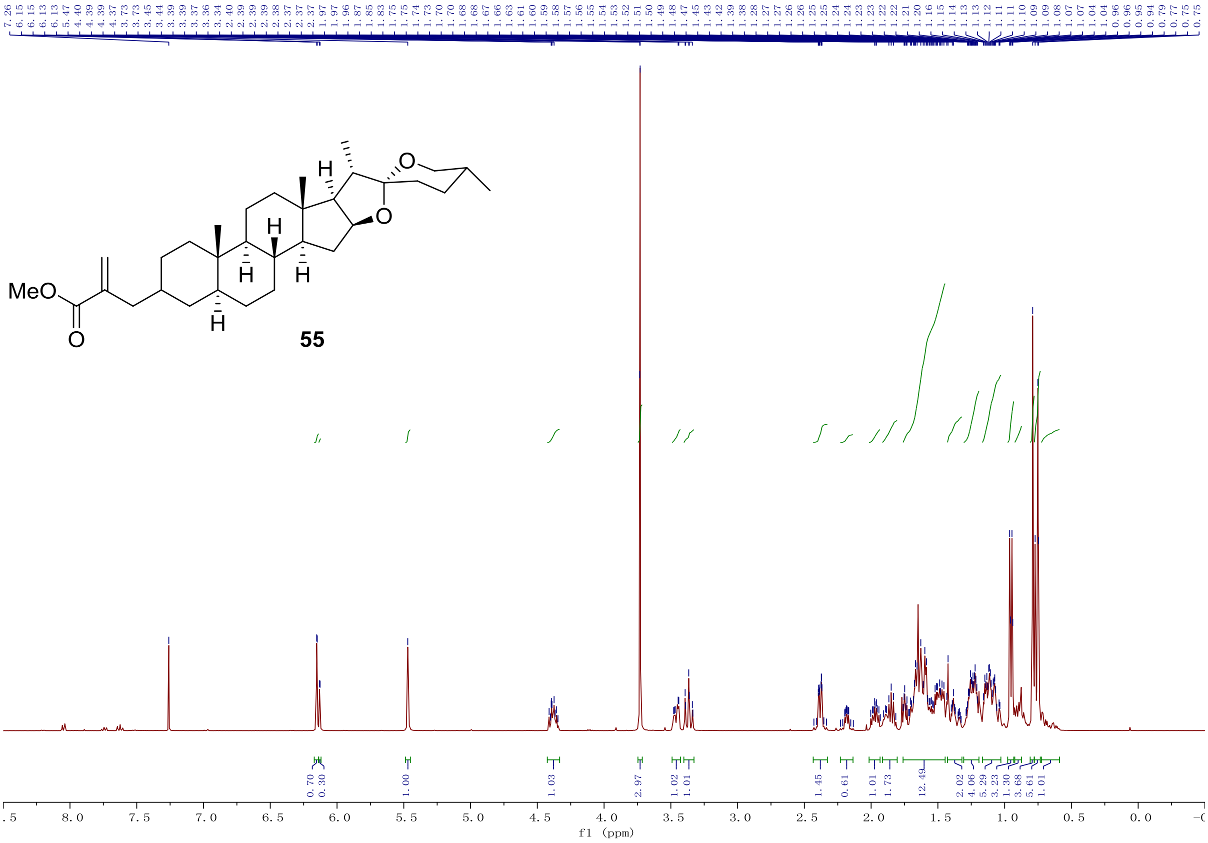
MeO
O
H
55
f1 (ppm)

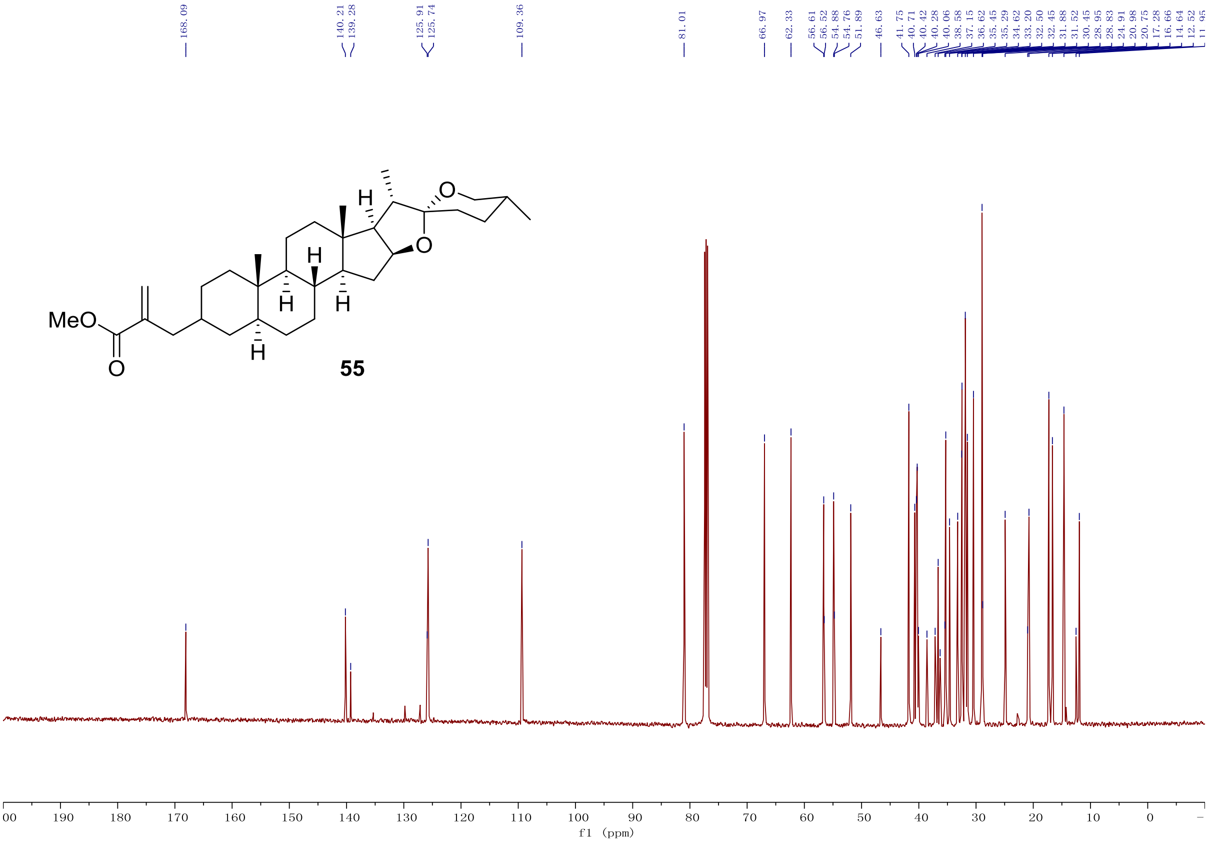
MeO
O
H
55
f1 (ppm)

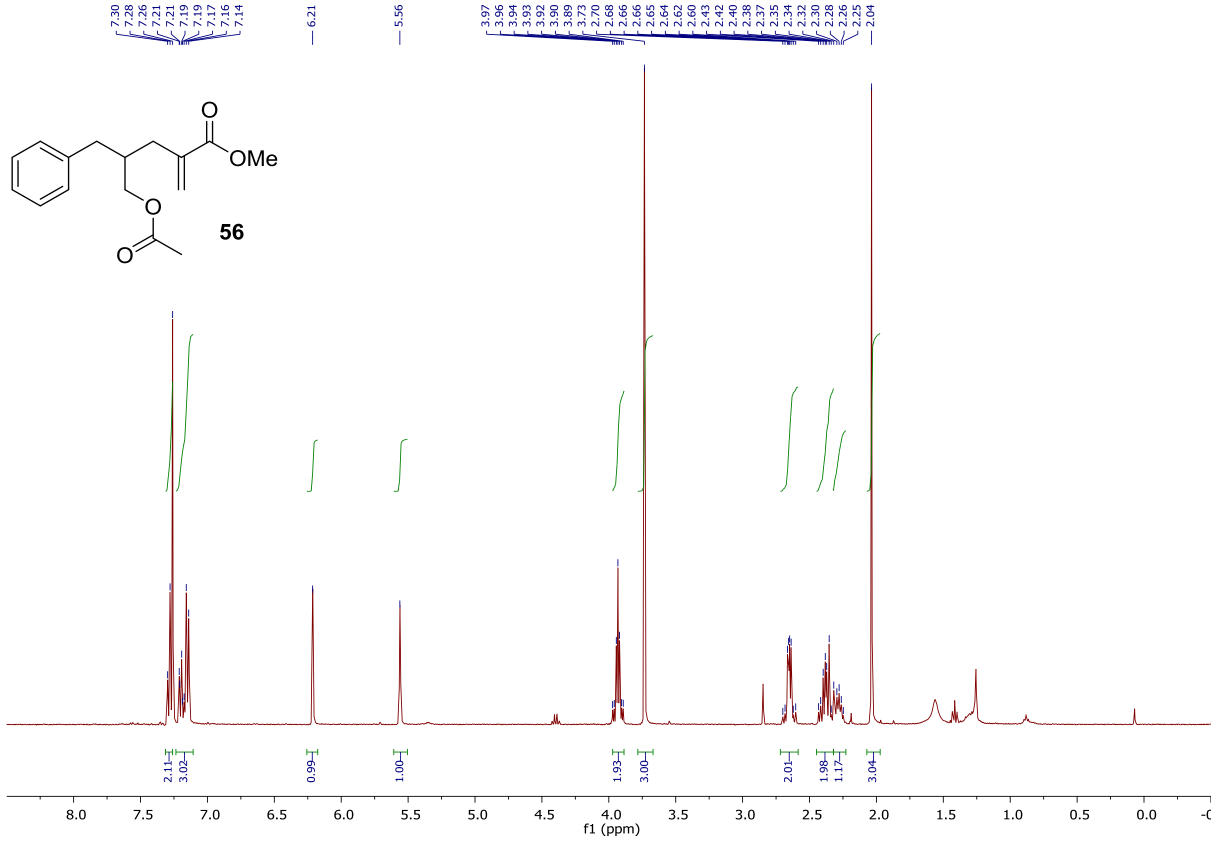

7.30 7.28 7.26 7.21 7.21 7.19 7.19 7.17 7.16 7.14
6.21
5.56
3.97 3.96 3.94 3.93 3.92 3.90 3.89 3.73 2.70 2.68 2.66 2.66 2.65 2.64 2.62 2.60 2.43 2.42 2.40 2.38 2.37 2.35 2.34 2.32 2.30 2.28 2.26 2.25 2.04
OMe
56
2.11 3.02 0.99 1.00 1.93 3.00 2.01 1.98 1.17 3.04
f1 (ppm)

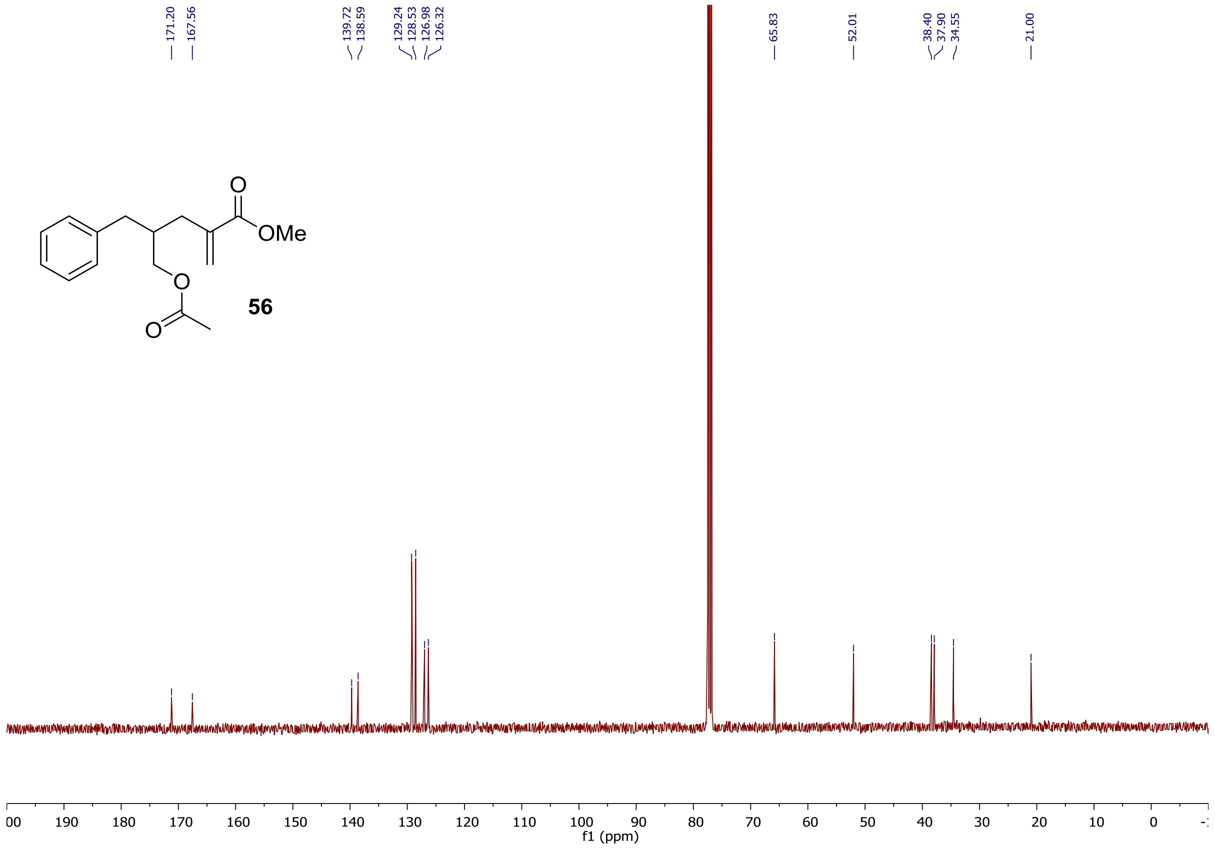

171.20 167.56
139.72 138.59
129.24 128.53 126.98 126.32
65.83
52.01
38.40 37.90 34.55
21.00
OMe
56
f1 (ppm)

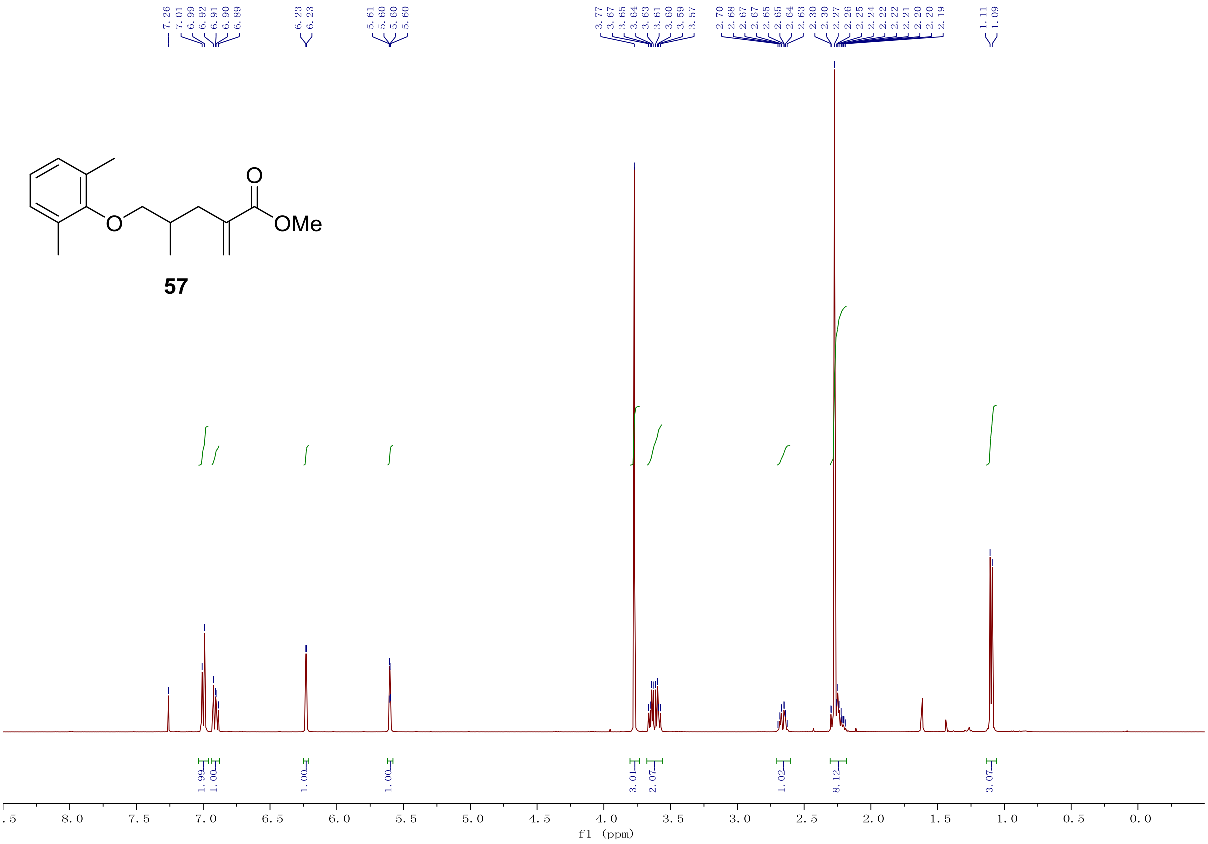
7.26
7.01
6.99
6.92
6.91
6.90
6.89
6.23
6.23
5.61
5.60
5.60
5.60
3.77
3.67
3.65
3.64
3.63
3.61
3.60
3.59
3.57
2.70
2.68
2.67
2.67
2.65
2.65
2.64
2.63
2.30
2.27
2.26
2.25
2.24
2.22
2.22
2.21
2.20
2.20
2.19
1.11
1.09
O
OMe
O
57
1.99
1.00
1.00
1.00
3.01
2.07
1.02
8.12
3.07
f1 (ppm)

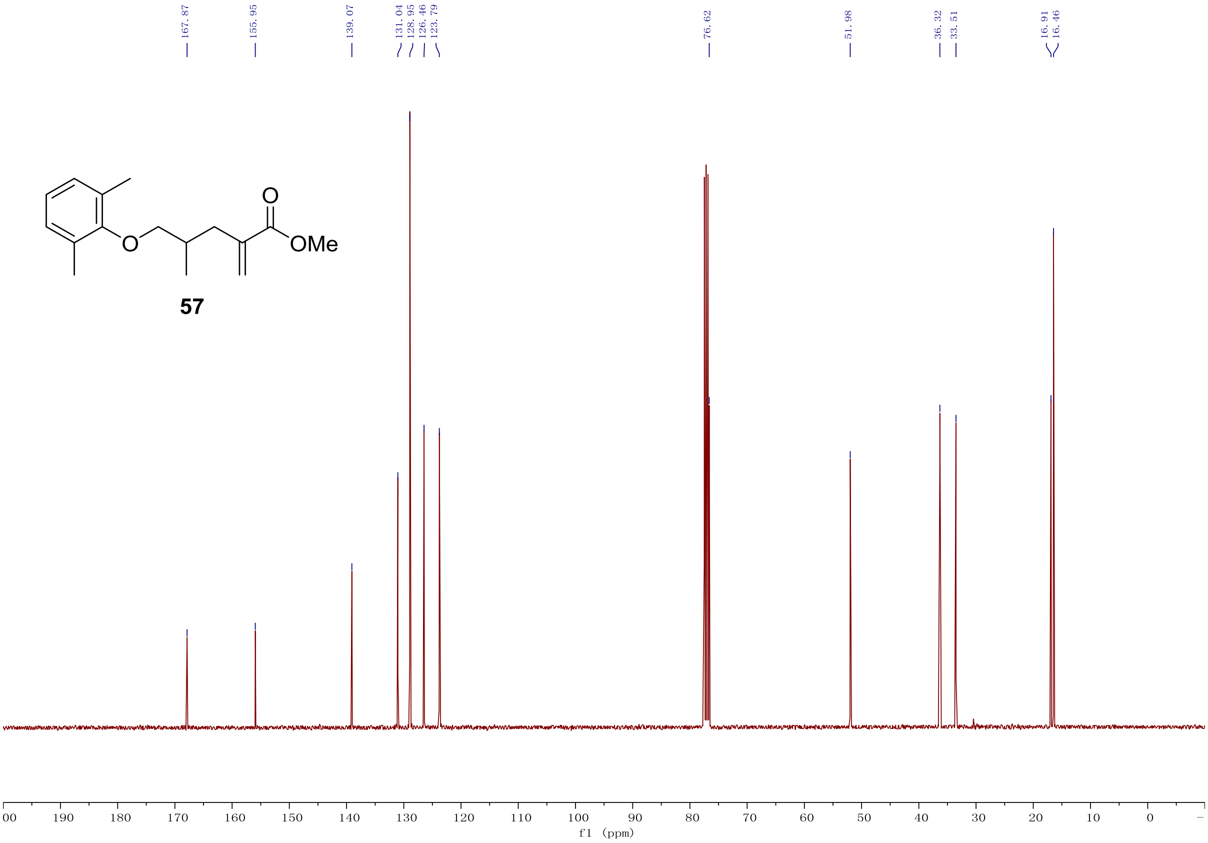
167.87
155.95
139.07
131.04
128.95
126.46
123.79
76.62
51.98
36.32
33.51
16.91
16.46
O
OMe
O
57
f1 (ppm)

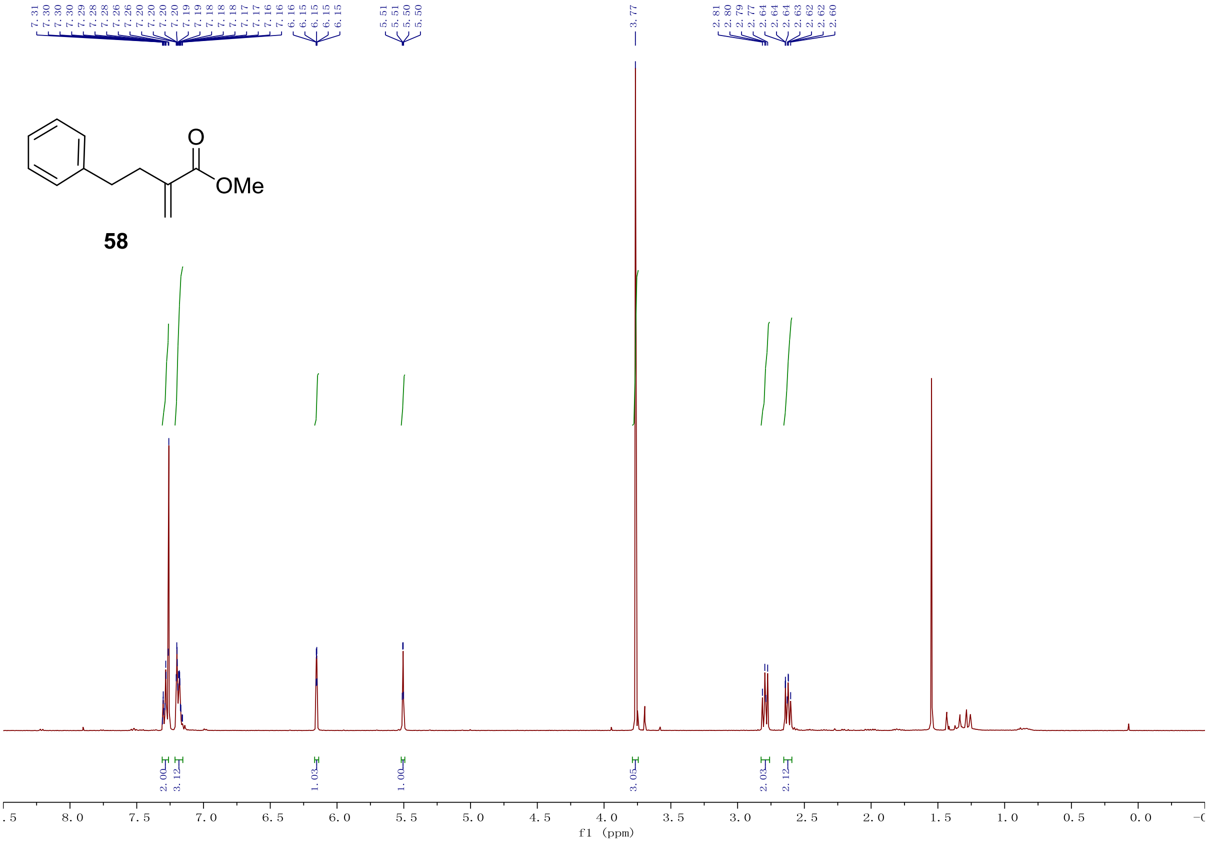
7.31 7.30 7.30 7.29 7.28 7.28 7.26 7.26 7.20 7.20 7.20 7.19 7.19 7.18 7.18 7.17 7.17 7.16 7.16
6.16 6.15 6.15 6.15
5.51 5.51 5.50 5.50
3.77
2.81 2.80 2.79 2.77 2.64 2.64 2.64 2.63 2.62 2.62 2.60
O
OMe
58
2.00
3.12
1.03
1.00
3.05
2.03
2.12
f1 (ppm)

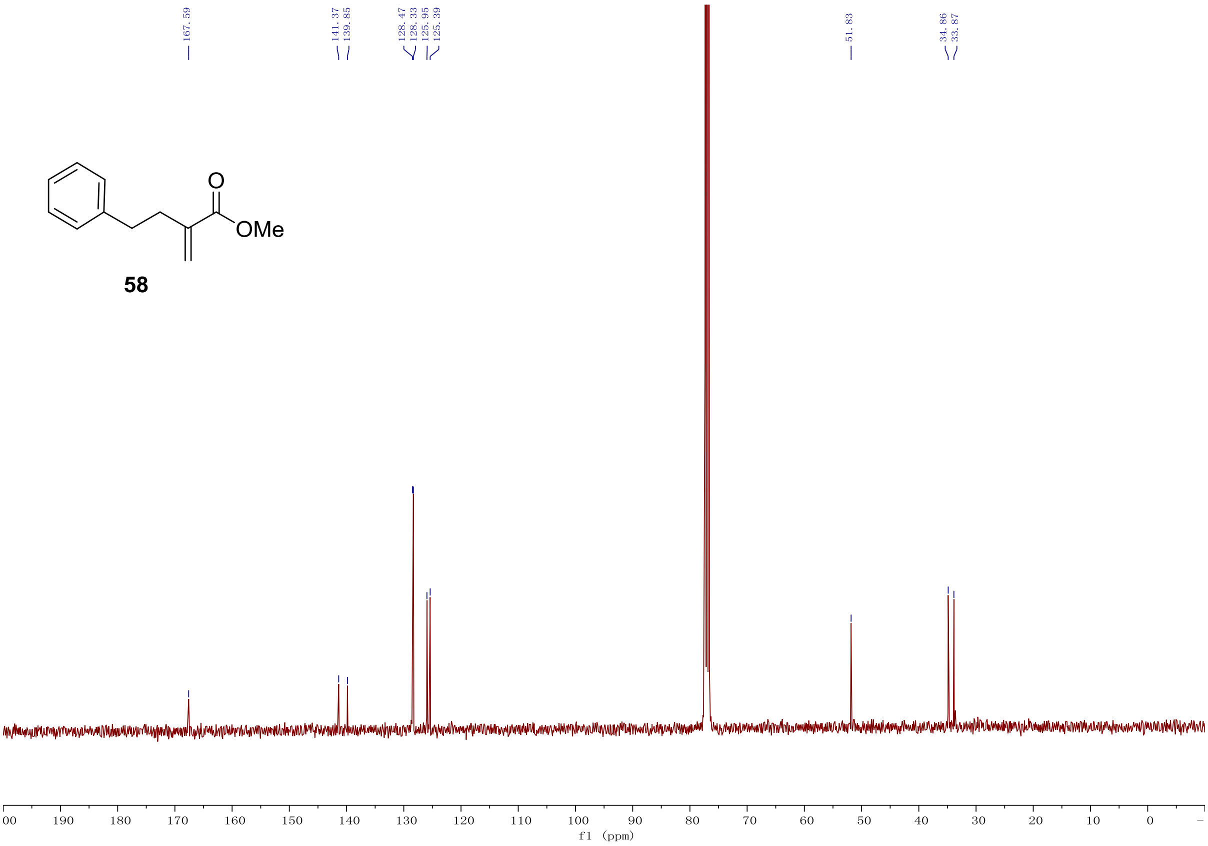
167.59
141.37 139.85
128.47 128.33 125.95 125.39
51.83
34.86 33.87
O
OMe
58
f1 (ppm)

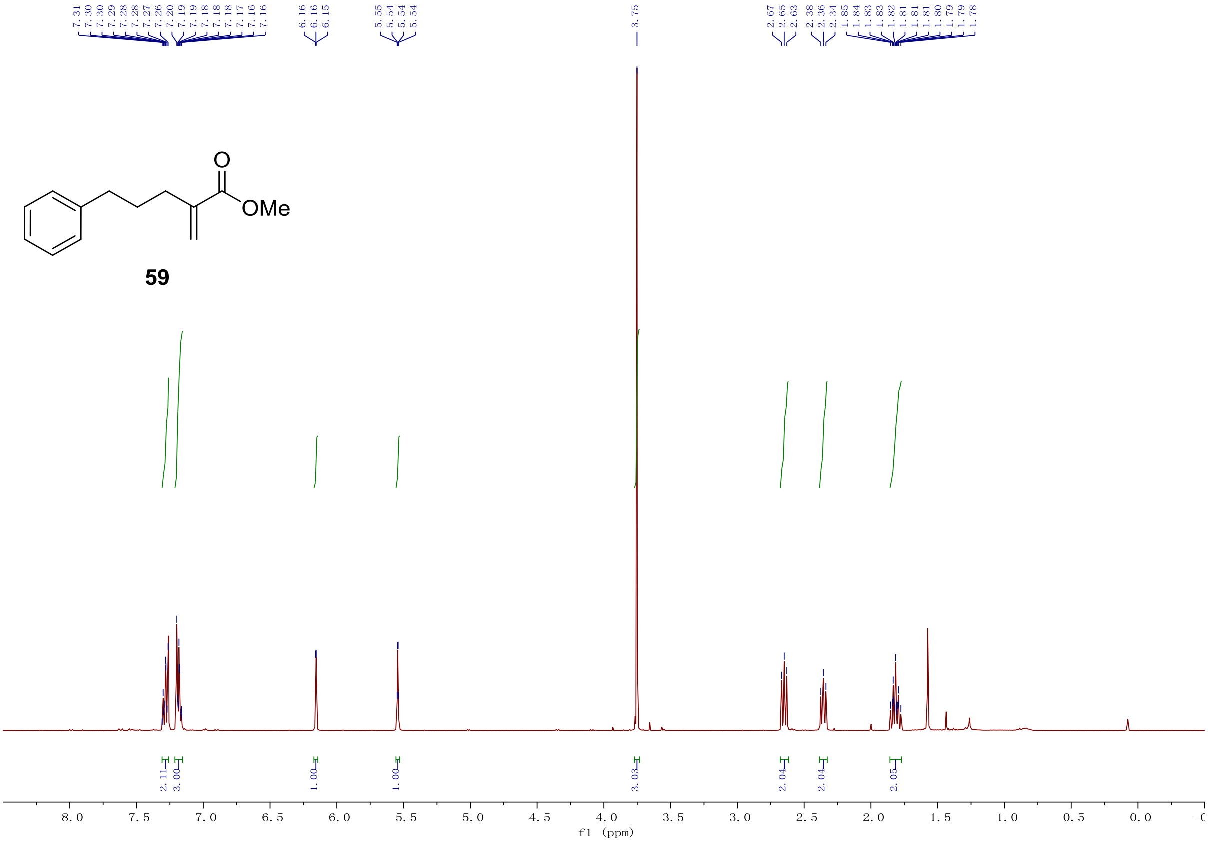
O
OMe
59
f1 (ppm)

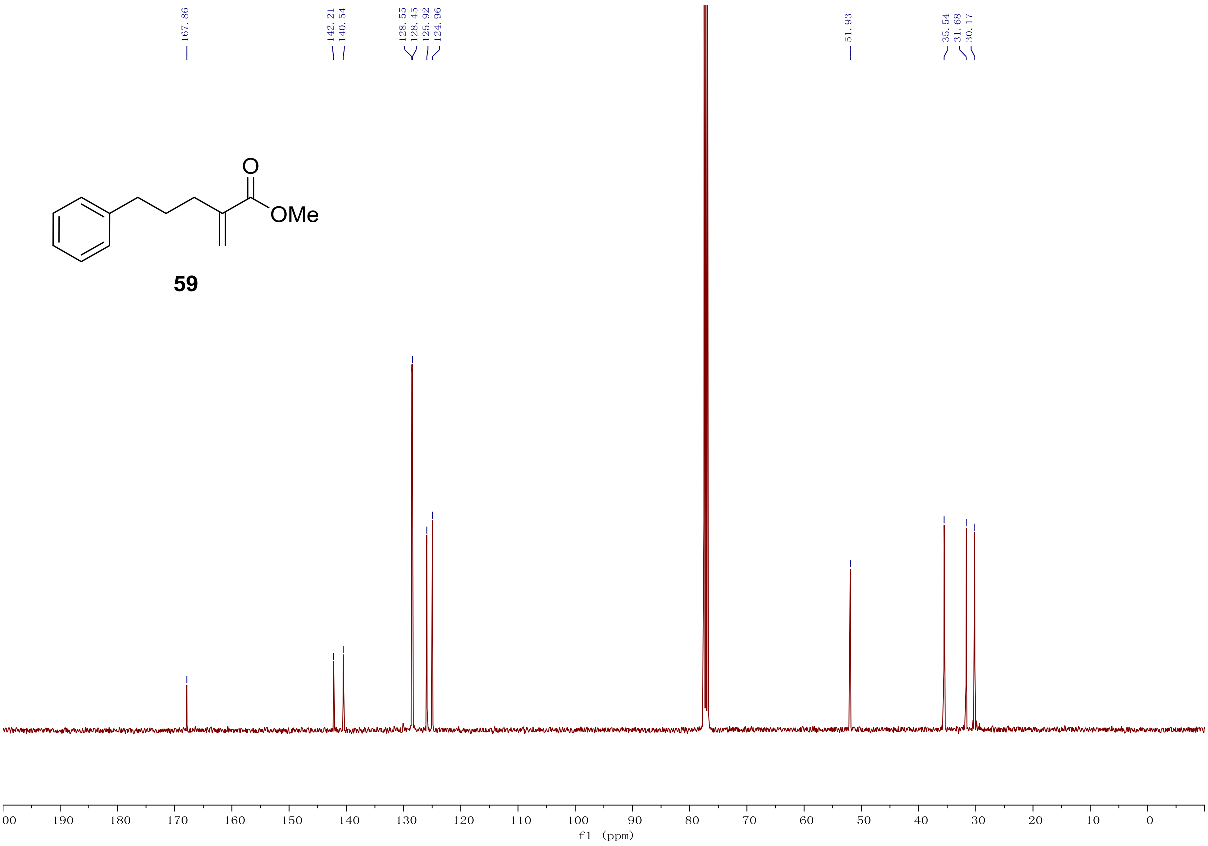
O
OMe
59
f1 (ppm)

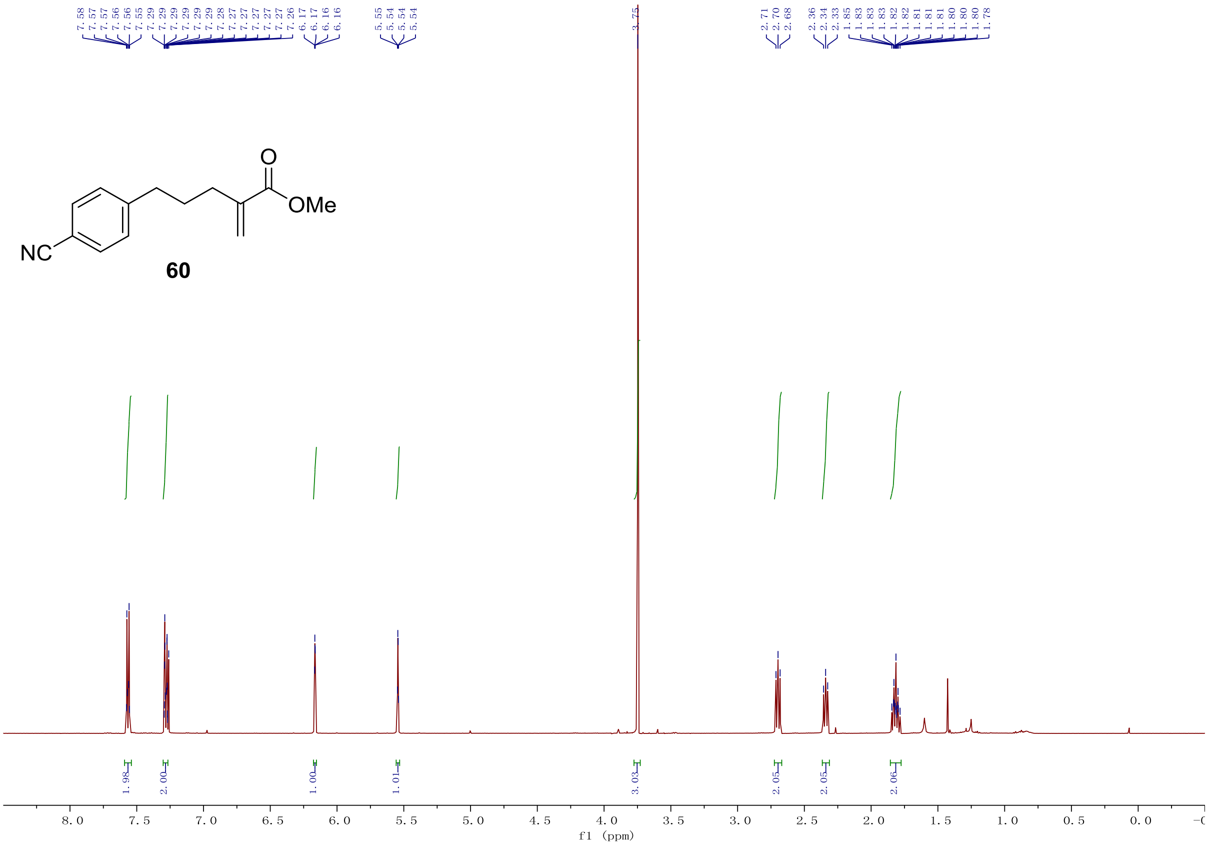
7.58 7.57 7.57 7.56 7.56 7.55 7.29 7.29 7.29 7.29 7.29 7.29 7.28 7.27 7.27 7.27 7.27 7.27 7.26 6.17 6.17 6.16 6.16
5.55 5.54 5.54 5.54
3.75
2.71 2.70 2.68
2.36 2.34 2.33 1.85 1.83 1.83 1.83 1.82 1.82 1.81 1.81 1.81 1.80 1.80 1.80 1.78
O
OMe
NC
60
1.98
2.00
1.00
1.01
3.03
2.05
2.05
2.06
8.0 7.5 7.0 6.5 6.0 5.5 5.0 4.5 4.0 3.5 3.0 2.5 2.0 1.5 1.0 0.5 0.0
f1 (ppm)

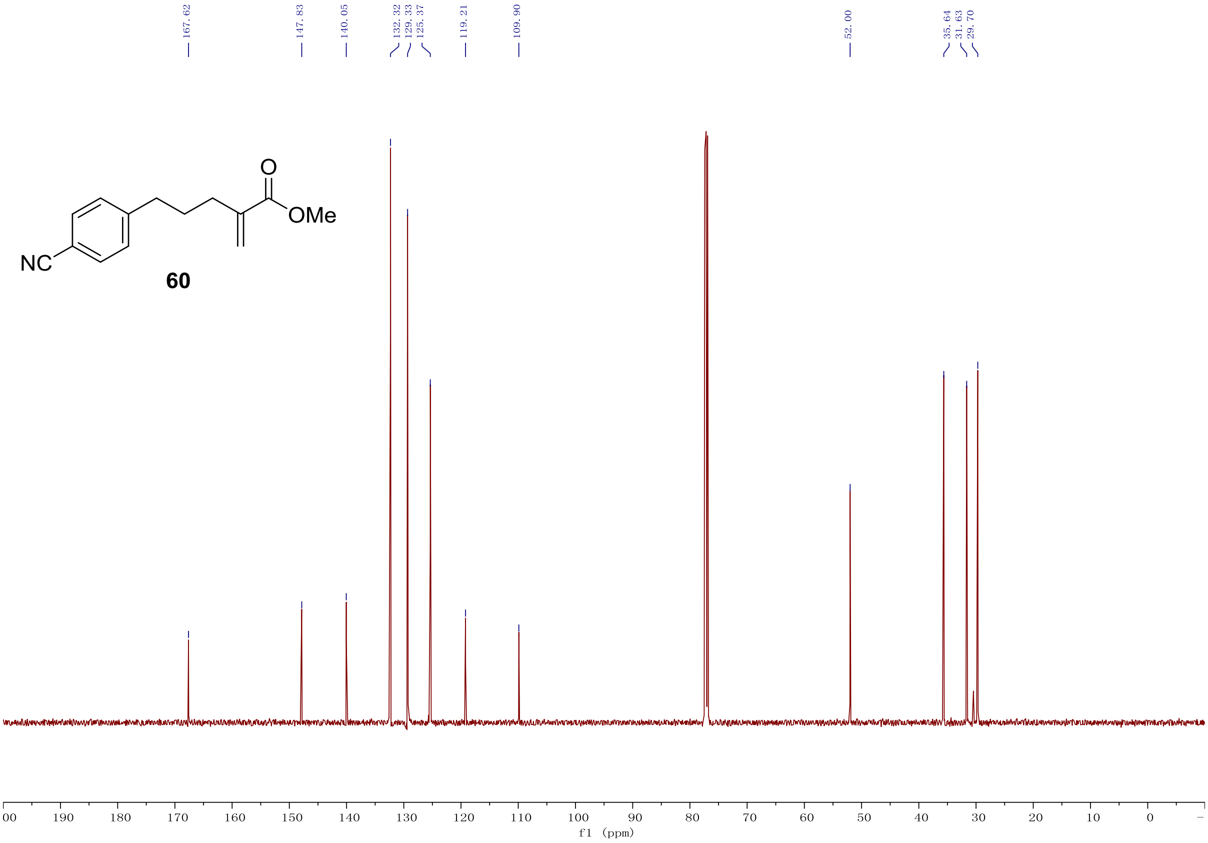
167.62
147.83
140.05
132.32 129.33 125.37
119.21
109.90
52.00
35.64 31.63 29.70
O
OMe
NC
60
190 180 170 160 150 140 130 120 110 100 90 80 70 60 50 40 30 20 10 0
f1 (ppm)

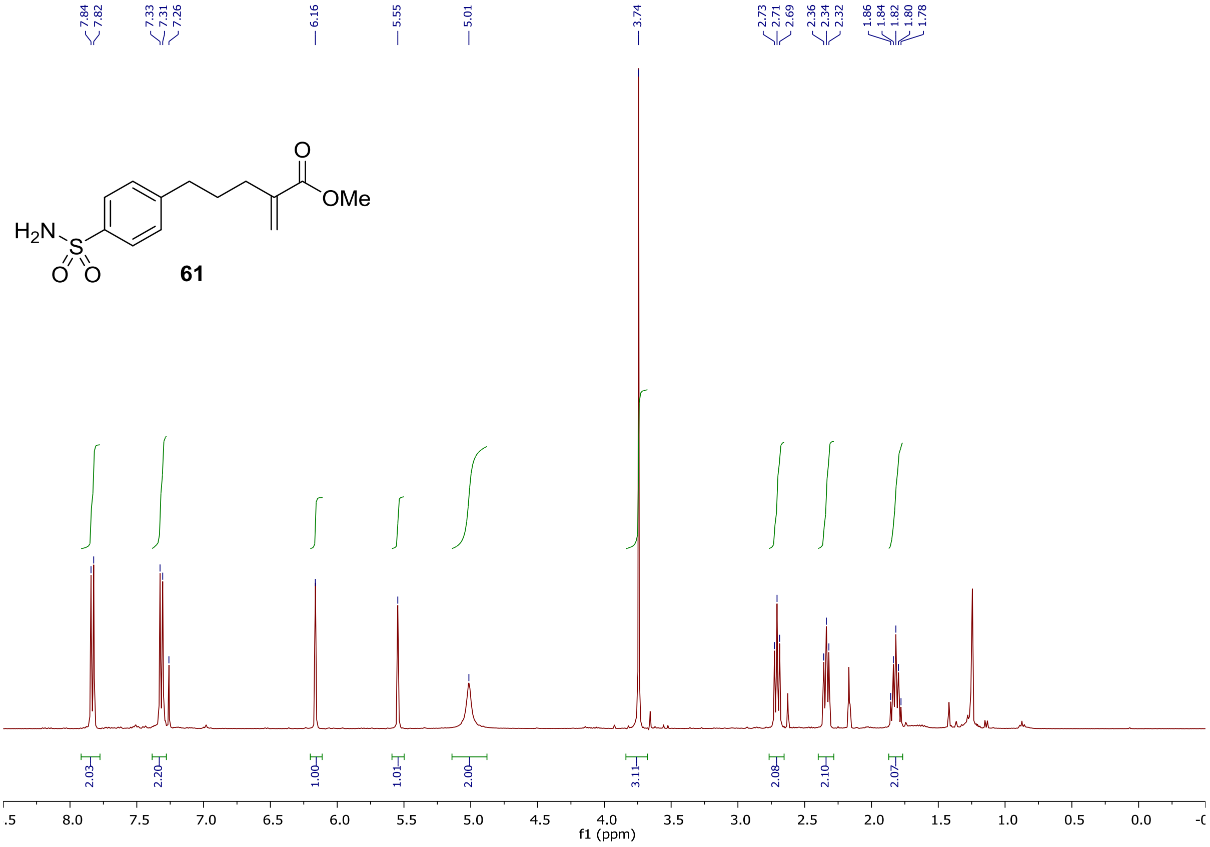
7.84
7.82
7.33
7.31
7.26
6.16
5.55
5.01
3.74
2.73
2.71
2.69
2.36
2.34
2.32
1.86
1.84
1.82
1.80
1.78
H2N
S
O
O
OMe
61
2.03
2.20
1.00
1.01
2.00
3.11
2.08
2.10
2.07
f1 (ppm)

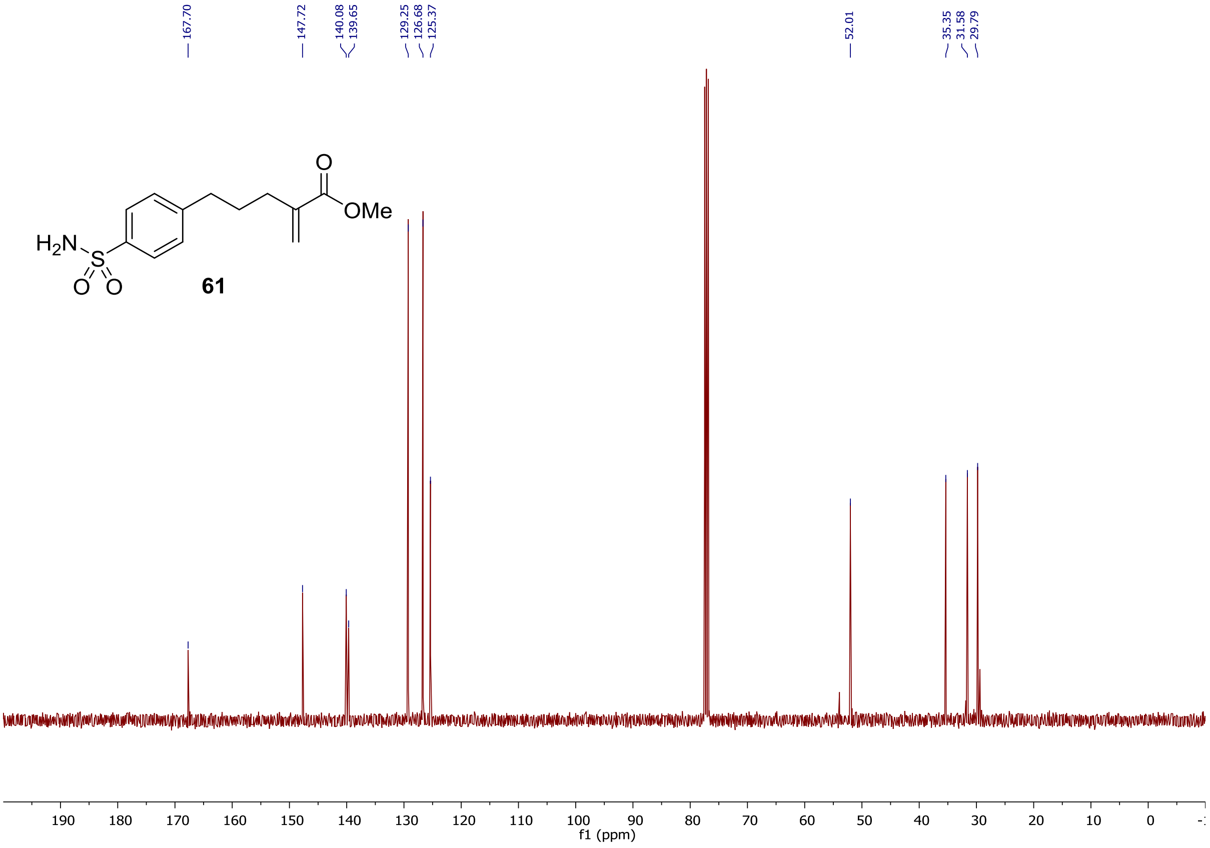
167.70
147.72
140.08
139.65
129.25
126.68
125.37
52.01
35.35
31.58
29.79
H2N
S
O
O
OMe
61
f1 (ppm)

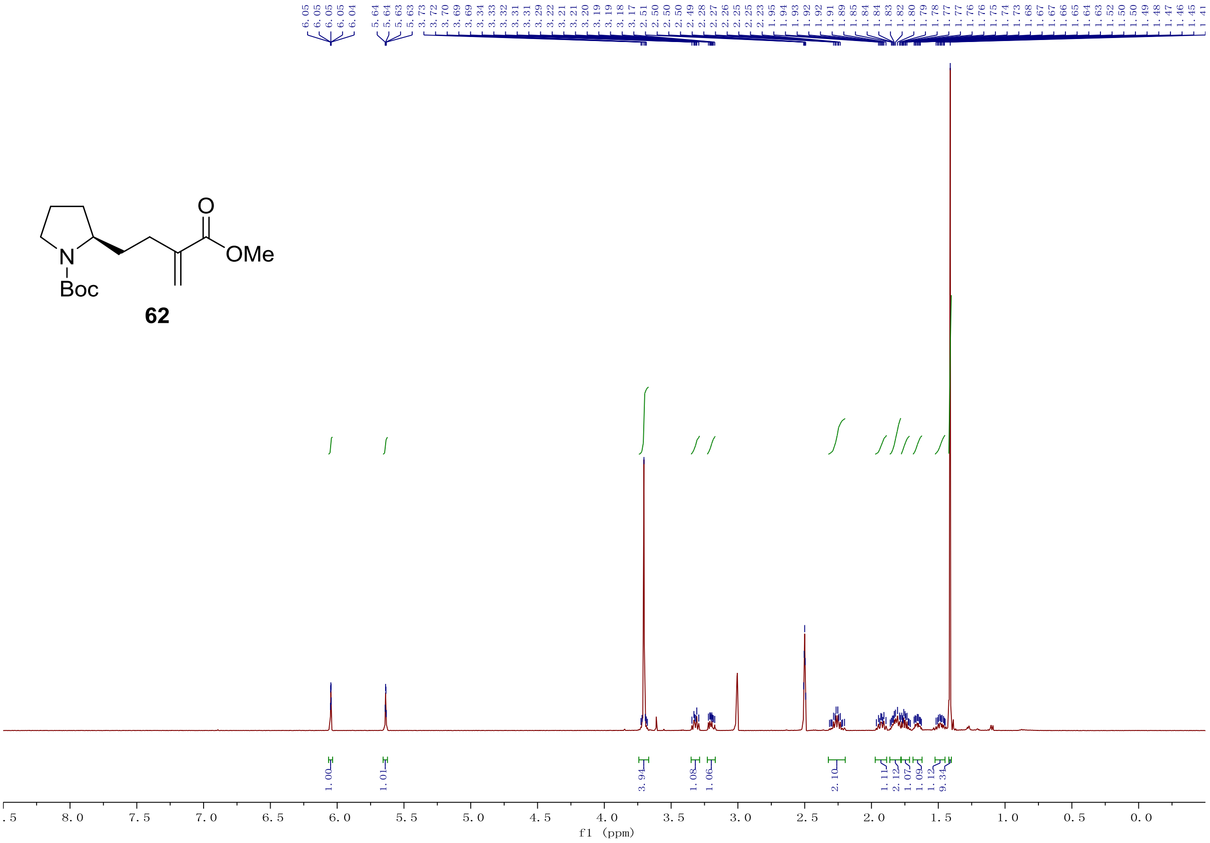
6.05 6.05 6.05 6.05 6.04 5.64 5.64 5.63 5.63 3.73 3.72 3.70 3.69 3.34 3.33 3.32 3.31 3.29 3.22 3.21 3.20 3.19 3.19 3.18 3.17 2.51 2.50 2.50 2.50 2.49 2.28 2.27 2.26 2.25 2.25 2.23 1.95 1.94 1.93 1.92 1.92 1.91 1.89 1.85 1.84 1.84 1.83 1.82 1.80 1.79 1.78 1.77 1.77 1.76 1.76 1.75 1.74 1.73 1.68 1.67 1.66 1.65 1.64 1.63 1.52 1.50 1.49 1.48 1.47 1.46 1.45 1.41
N
Boc
O
OMe
1.00
1.01
3.94
1.08
1.06
2.10
1.11
2.12
1.07
1.09
1.12
9.34
f1 (ppm)

62

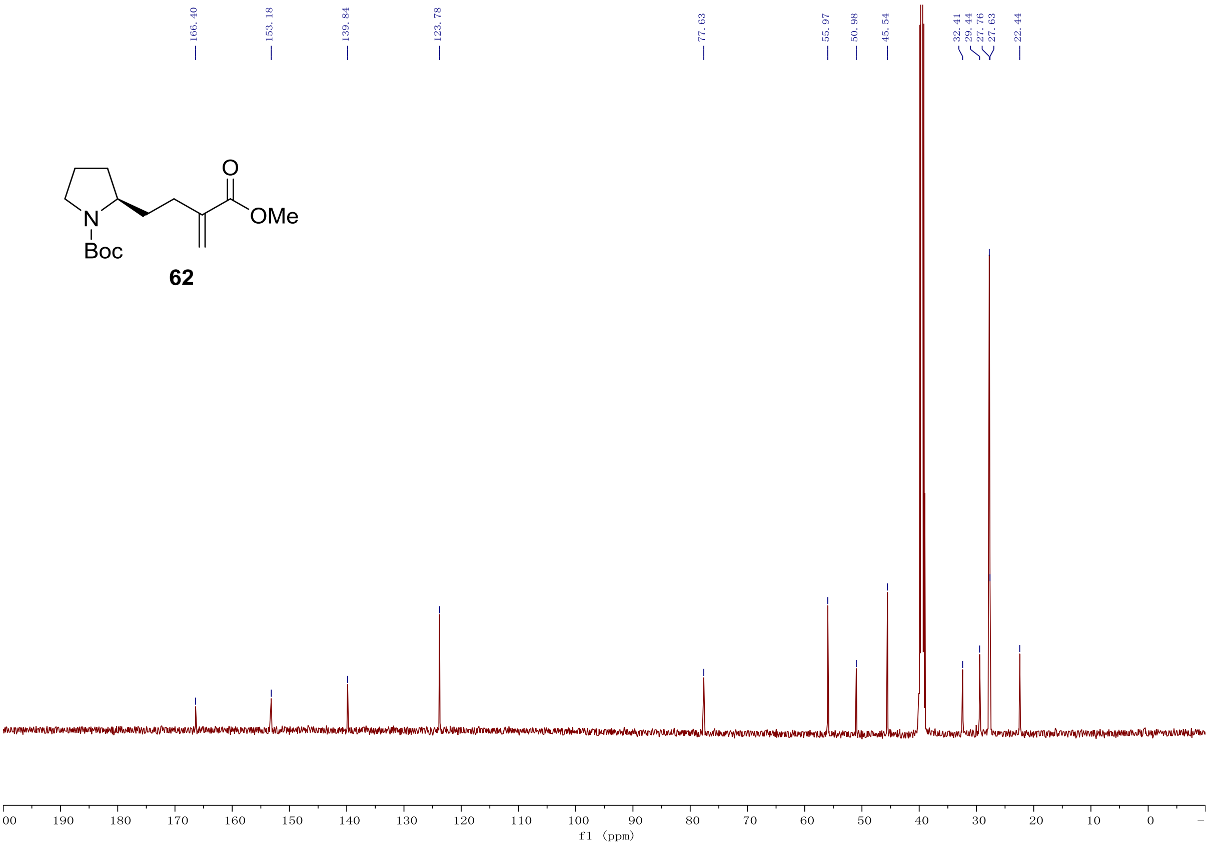
166.40
153.18
139.84
123.78
77.63
55.97
50.98
45.54
32.41
29.44
27.76
27.63
22.44
N
Boc
O
OMe
f1 (ppm)

62

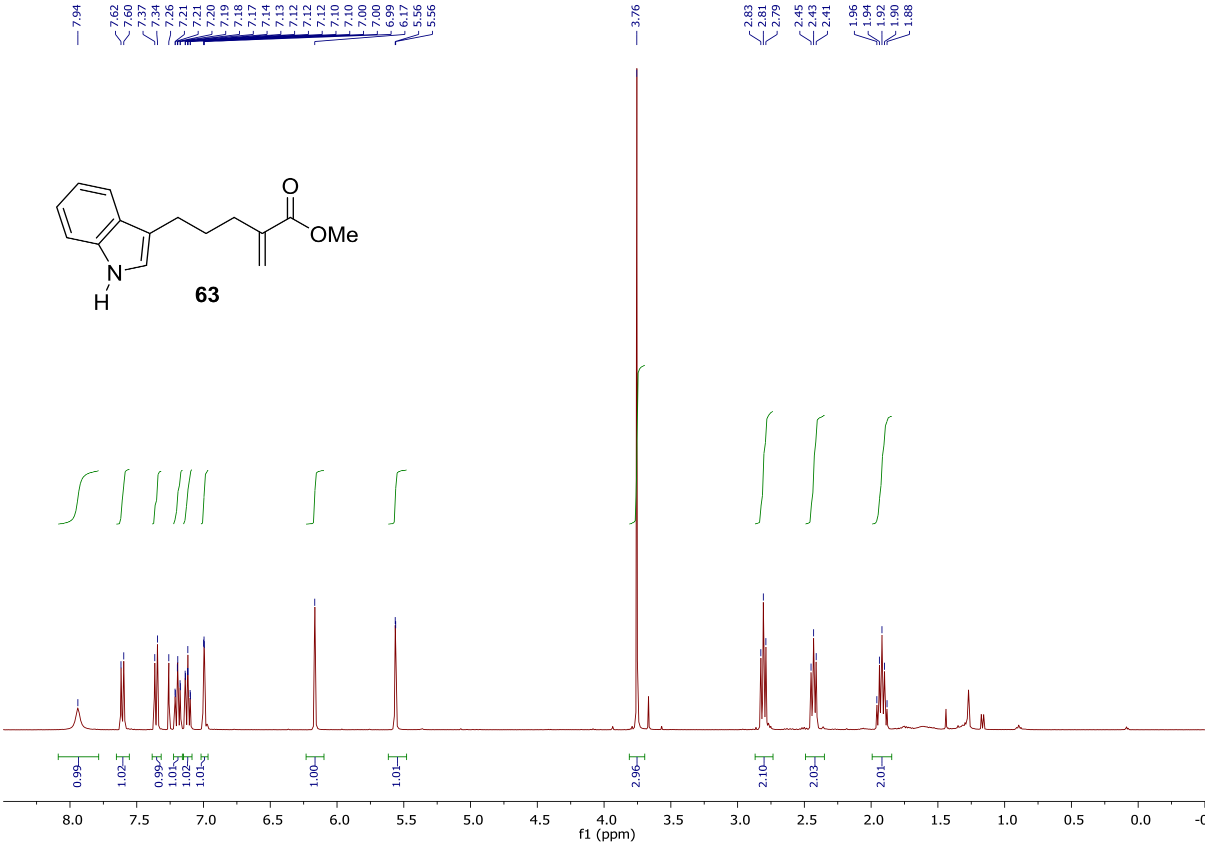
7.94
7.62
7.60
7.37
7.34
7.26
7.21
7.21
7.20
7.19
7.18
7.17
7.14
7.13
7.12
7.12
7.12
7.10
7.10
7.00
7.00
6.99
6.17
5.56
5.56
3.76
2.83
2.81
2.79
2.45
2.43
2.41
1.96
1.94
1.92
1.90
1.88
O
OMe
N
H
63
0.99
1.02
0.99
1.01
1.02
1.01
1.00
1.01
2.96
2.10
2.03
2.01
f1 (ppm)

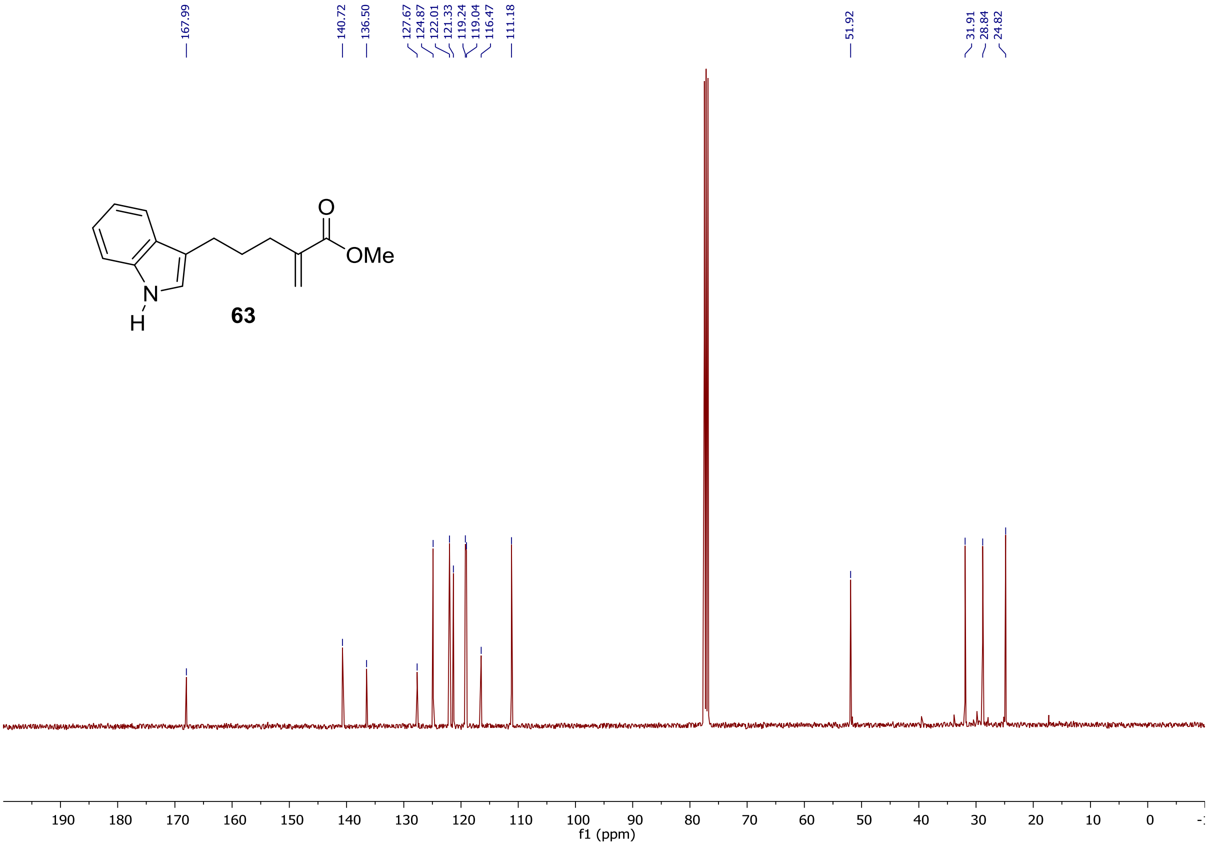
167.99
140.72
136.50
127.67
124.87
122.01
121.33
119.24
119.04
116.47
111.18
51.92
31.91
28.84
24.82
O
OMe
N
H
63
f1 (ppm)

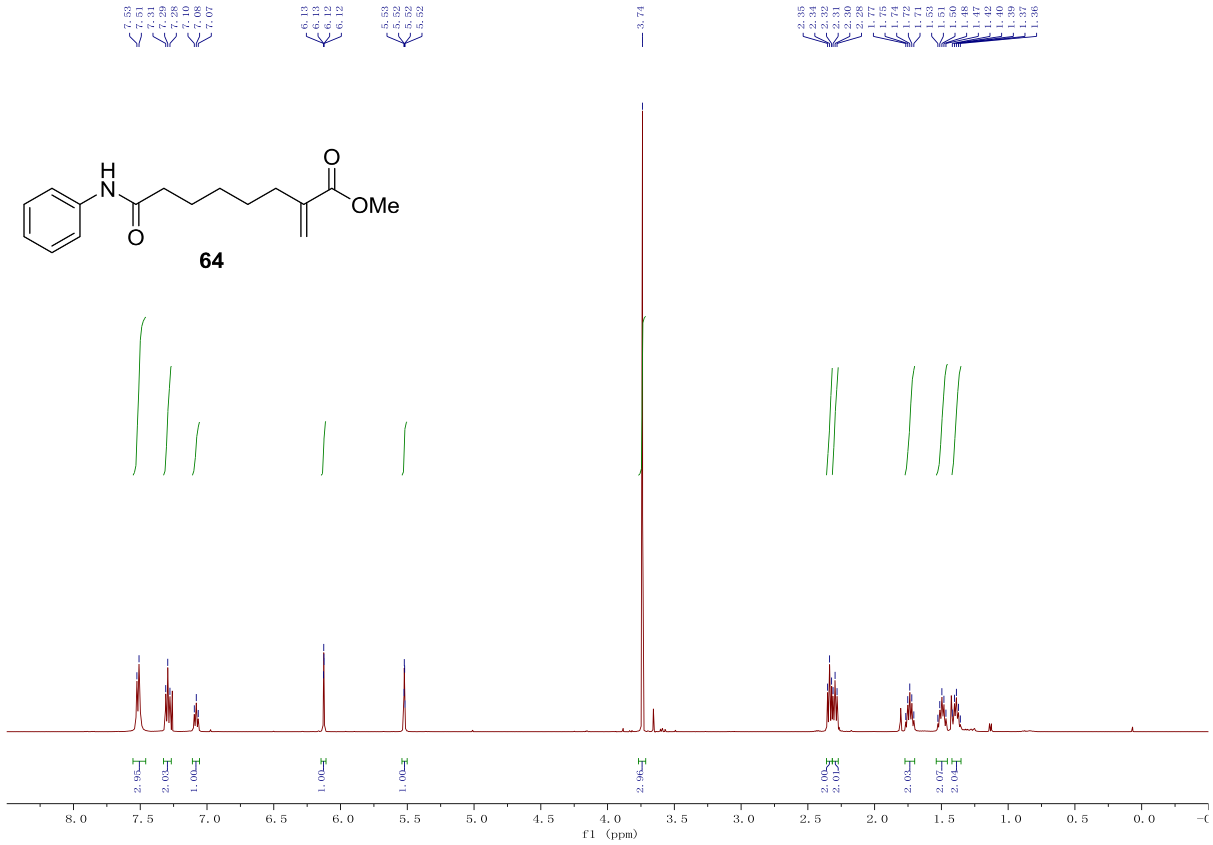
64
OMe
f1 (ppm)

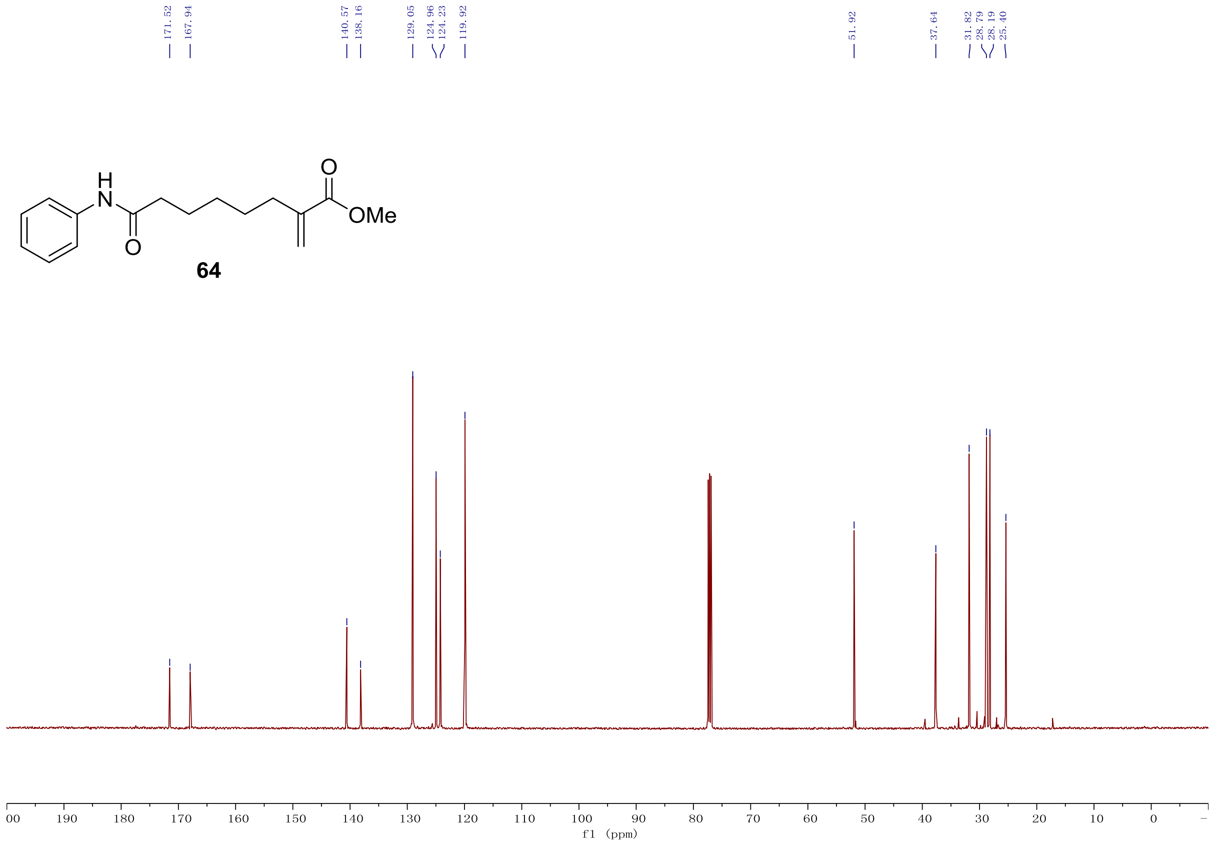
64
OMe
f1 (ppm)

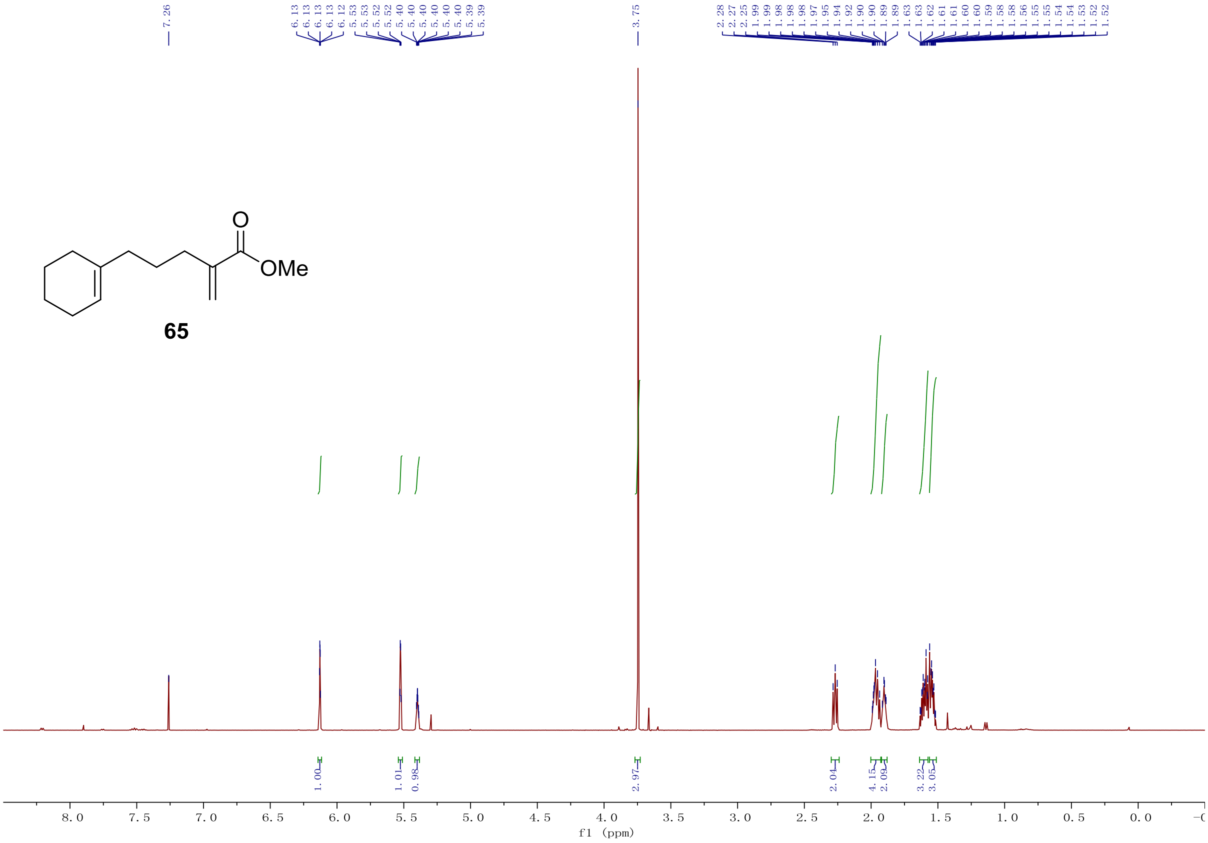

7.26
6.13
6.13
6.13
6.13
6.12
5.53
5.53
5.52
5.52
5.40
5.40
5.40
5.40
5.40
5.40
5.39
5.39
3.75
2.28
2.27
2.25
1.99
1.99
1.98
1.98
1.98
1.98
1.97
1.95
1.94
1.92
1.90
1.90
1.89
1.89
1.63
1.62
1.61
1.61
1.60
1.60
1.59
1.58
1.58
1.56
1.55
1.55
1.54
1.54
1.53
1.52
1.52
OMe
O
65
1.00
1.01
0.98
2.97
2.04
4.15
2.09
3.22
3.05
f1 (ppm)

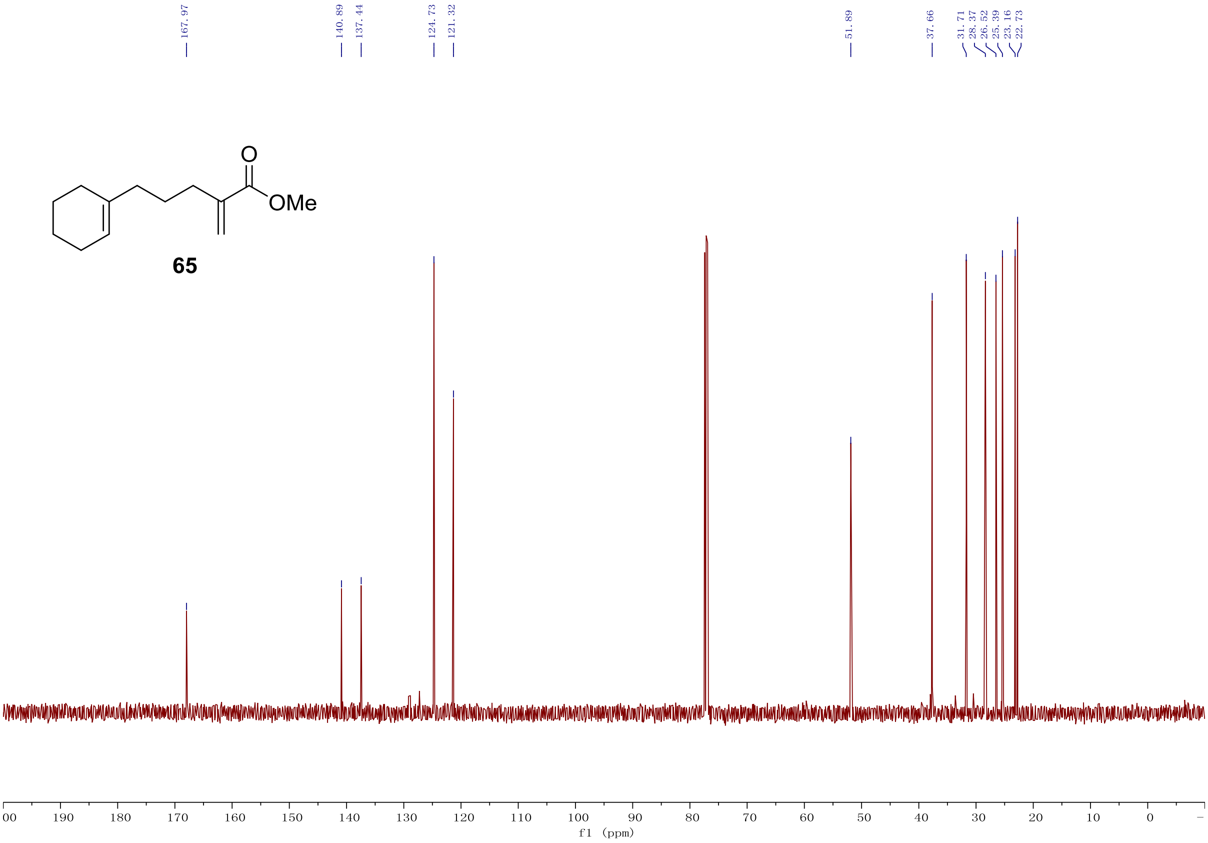

167.97
140.89
137.44
124.73
121.32
51.89
37.66
31.71
28.37
26.52
25.39
23.16
22.73
O
OMe
65
f1 (ppm)

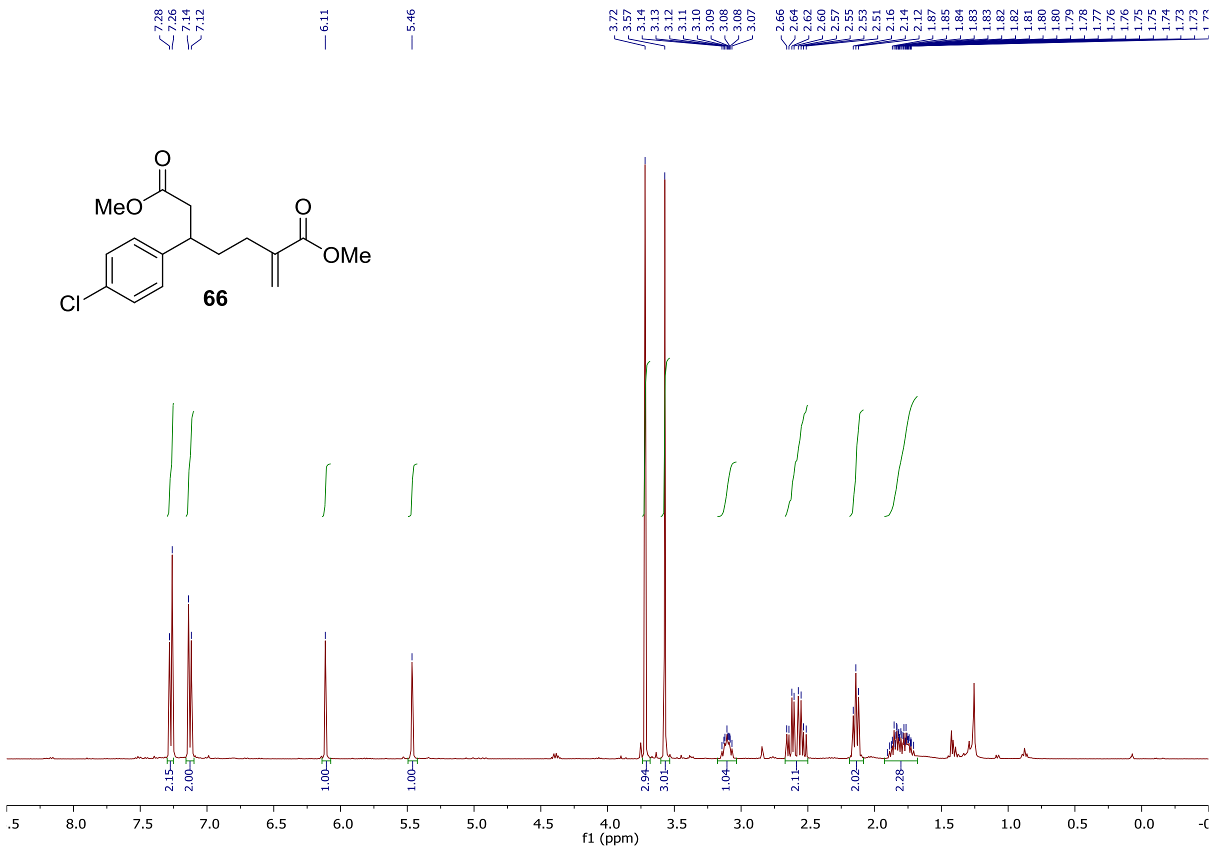

7.28
7.26
7.14
7.12
6.11
5.46
3.72
3.57
3.14
3.13
3.12
3.11
3.10
3.09
3.08
3.08
3.07
2.66
2.64
2.62
2.60
2.57
2.55
2.53
2.51
2.16
2.14
2.12
1.87
1.85
1.84
1.83
1.83
1.82
1.82
1.81
1.80
1.80
1.79
1.78
1.77
1.76
1.76
1.75
1.74
1.73
1.73
MeO
O
O
OMe
Cl
66
2.15
2.00
1.00
1.00
2.94
3.01
1.04
2.11
2.02
2.28
f1 (ppm)

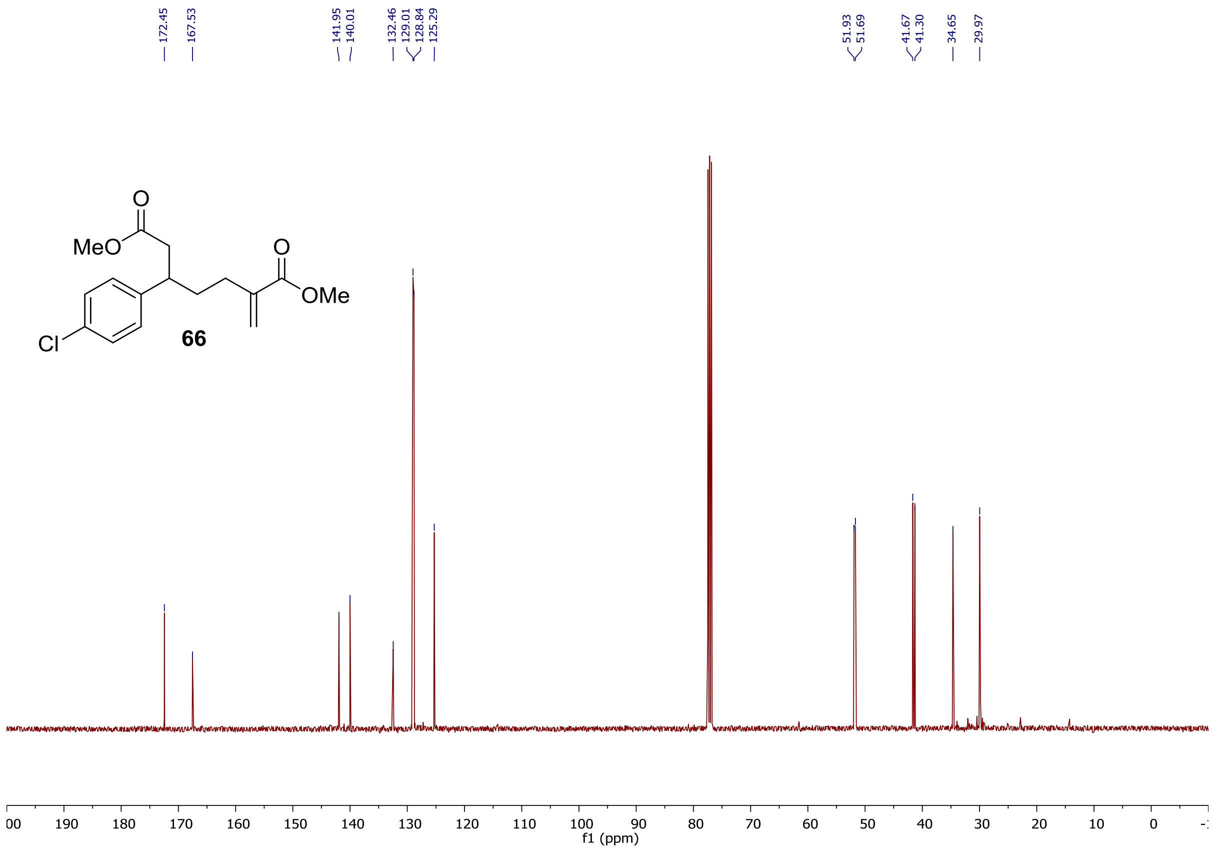

172.45
167.53
141.95
140.01
132.46
129.01
128.84
125.29
51.93
51.69
41.67
41.30
34.65
29.97
MeO
O
O
OMe
Cl
66
f1 (ppm)

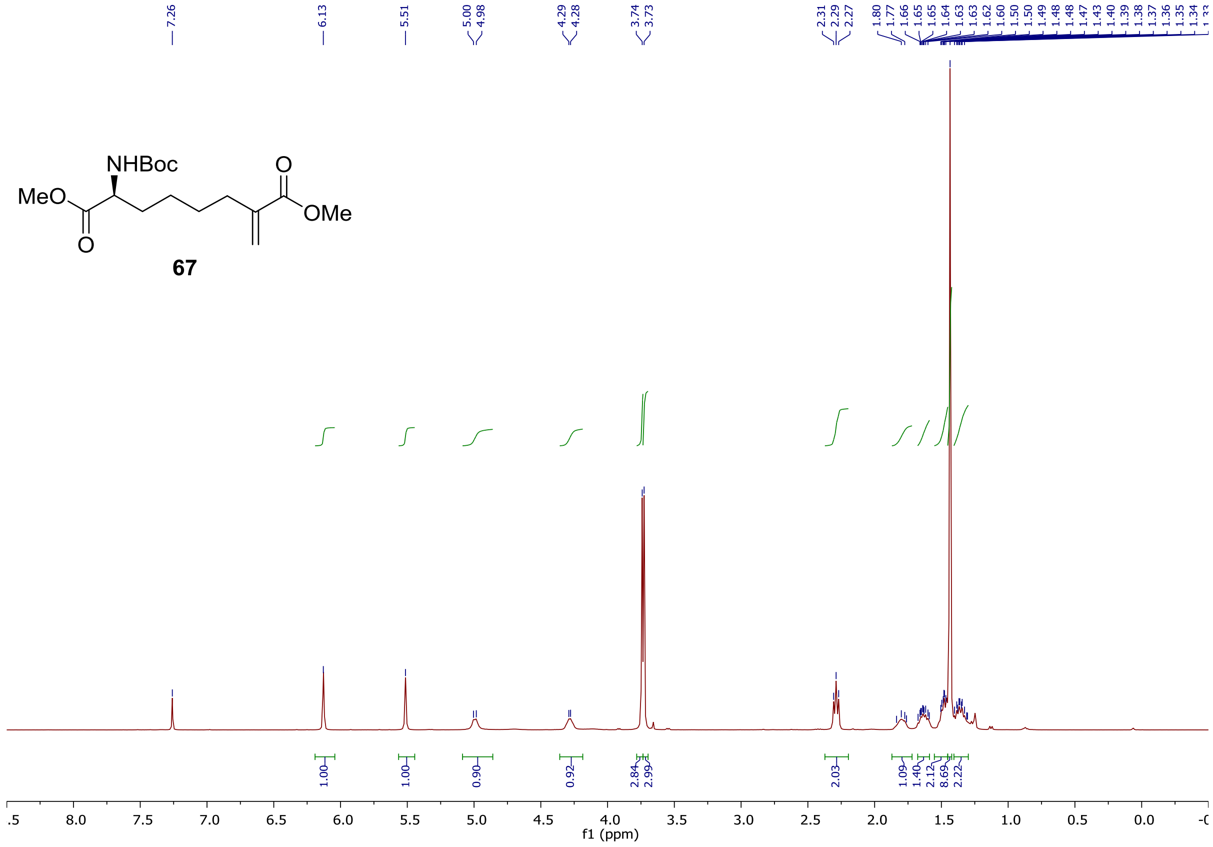
NHBoc
MeO
O
O
OMe
67
f1 (ppm)

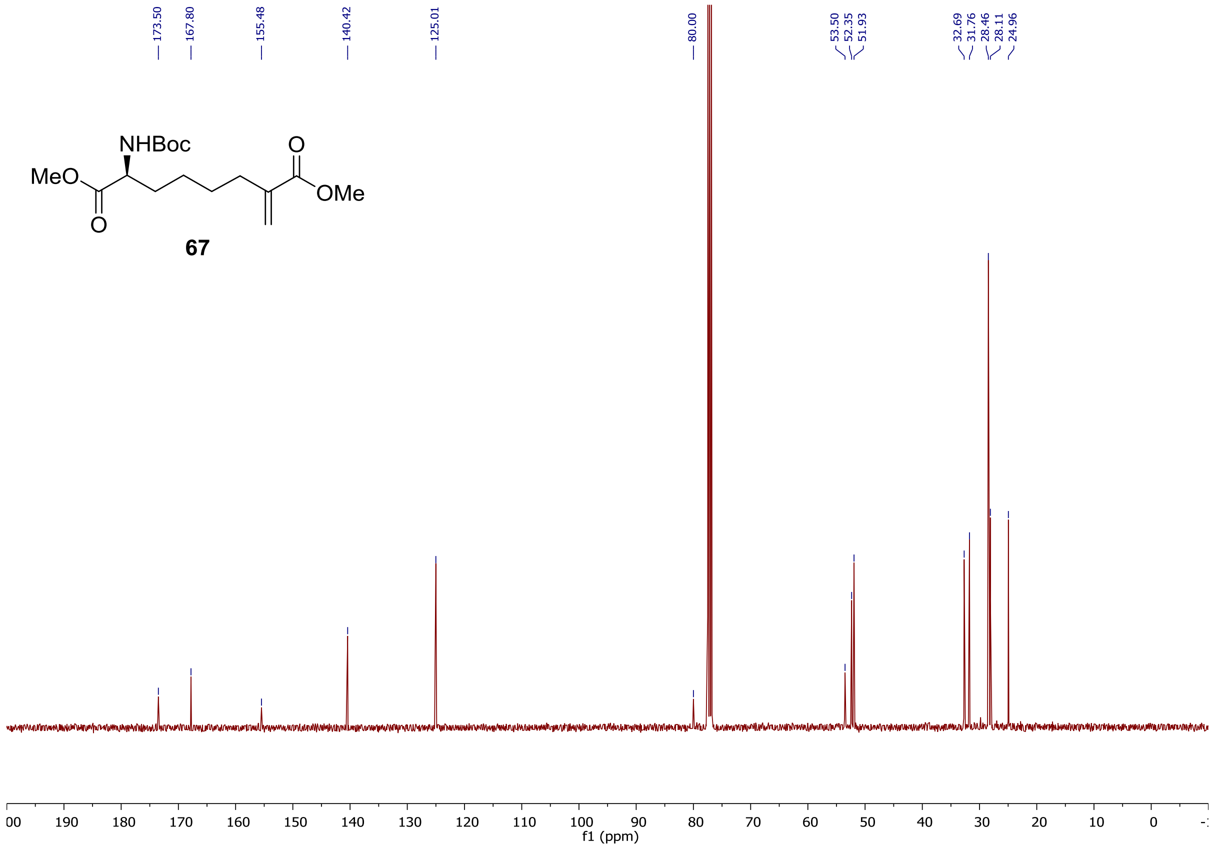
NHBoc
MeO
O
O
OMe
67
f1 (ppm)

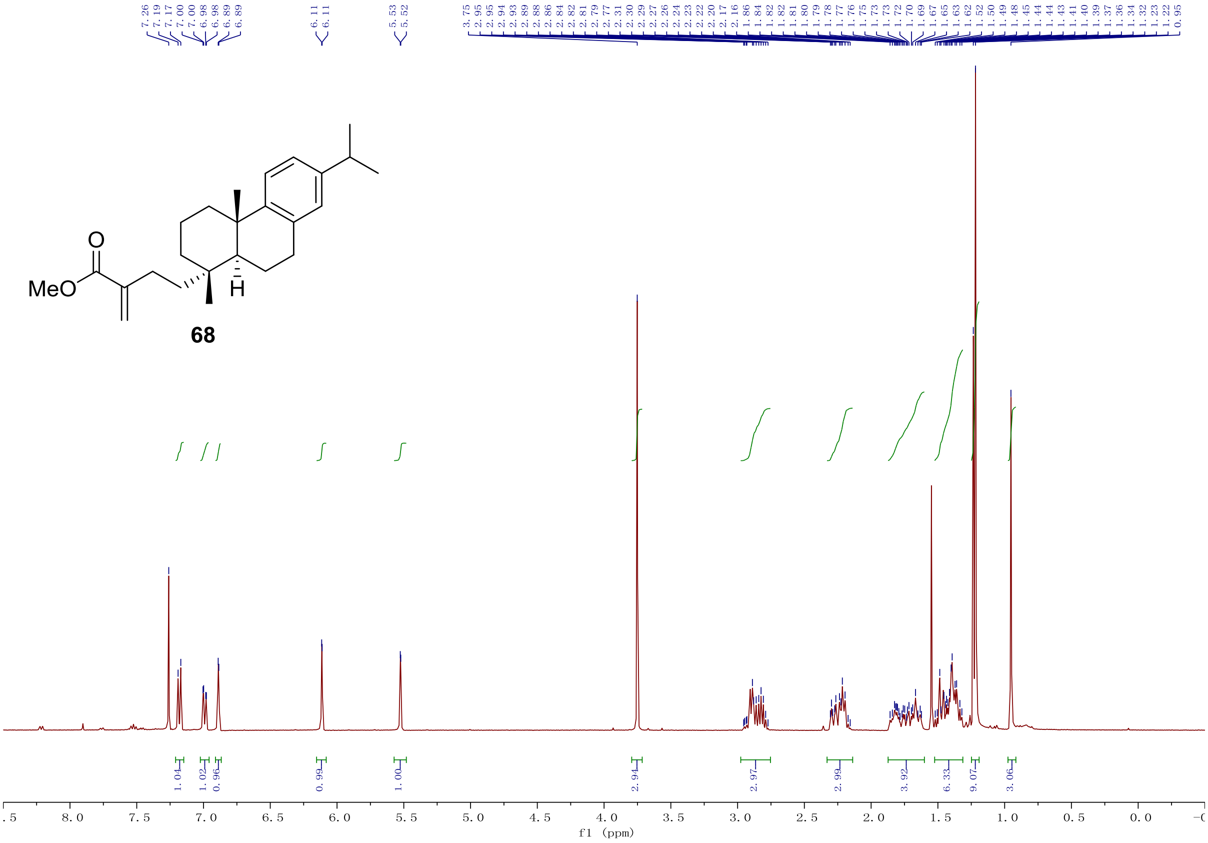
7.26 7.19 7.17 7.00 7.00 6.98 6.98 6.89 6.89
6.11 6.11
5.53 5.52
3.75
MeO
O
H
68
1.04 1.02 0.96 0.99 1.00 2.94 2.97 2.99 3.92 6.33 9.07 3.06
f1 (ppm)

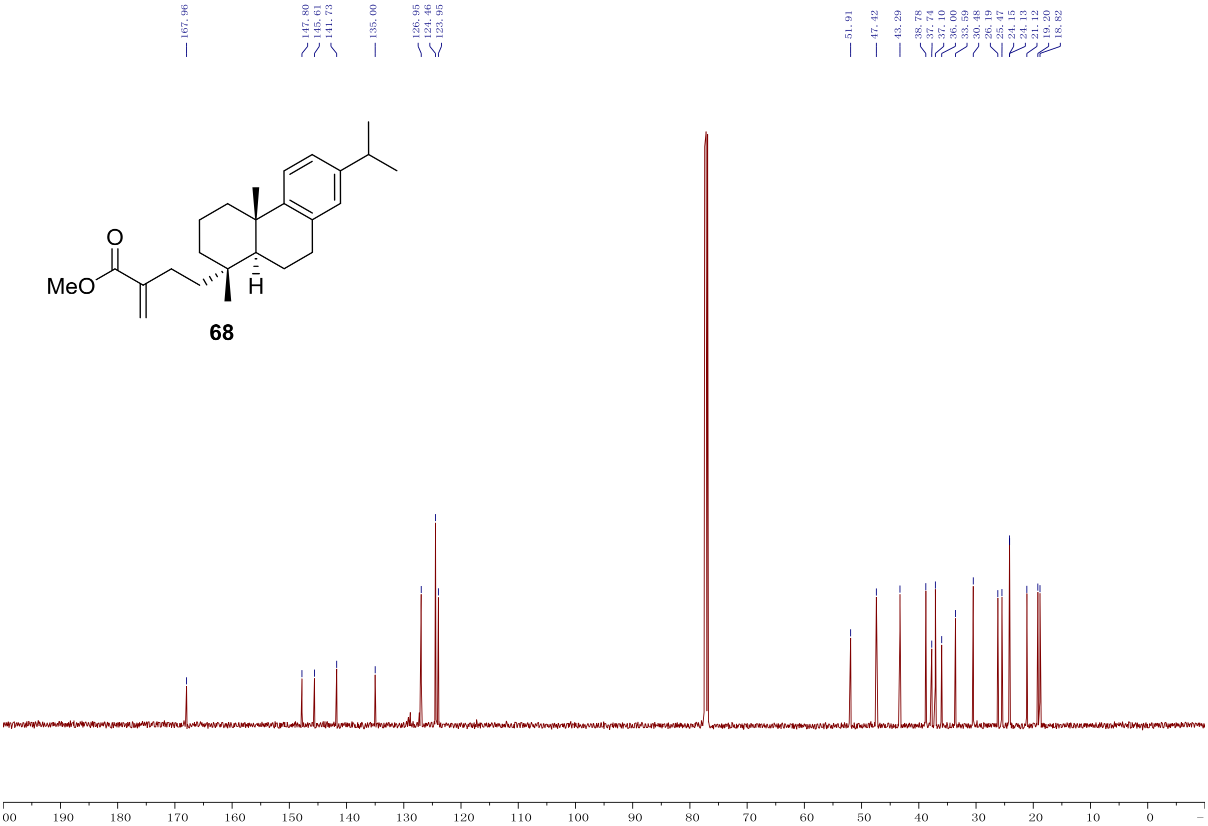
167.96
147.80 145.61 141.73
135.00
126.95 124.46 123.95
51.91 47.42 43.29 38.78 37.74 37.10 36.00 33.59 30.48 26.19 25.47 24.15 24.13 21.12 19.20 18.82
MeO
O
H
68
f1 (ppm)

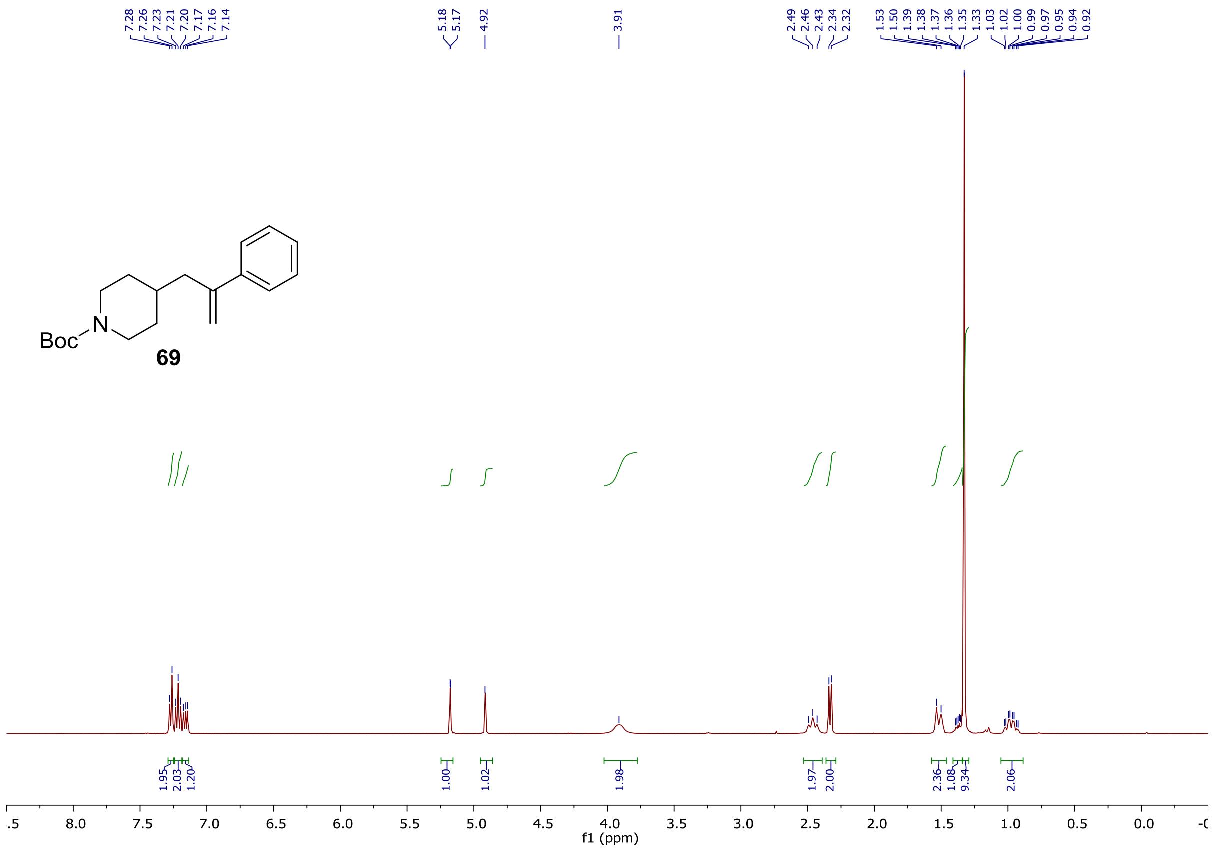

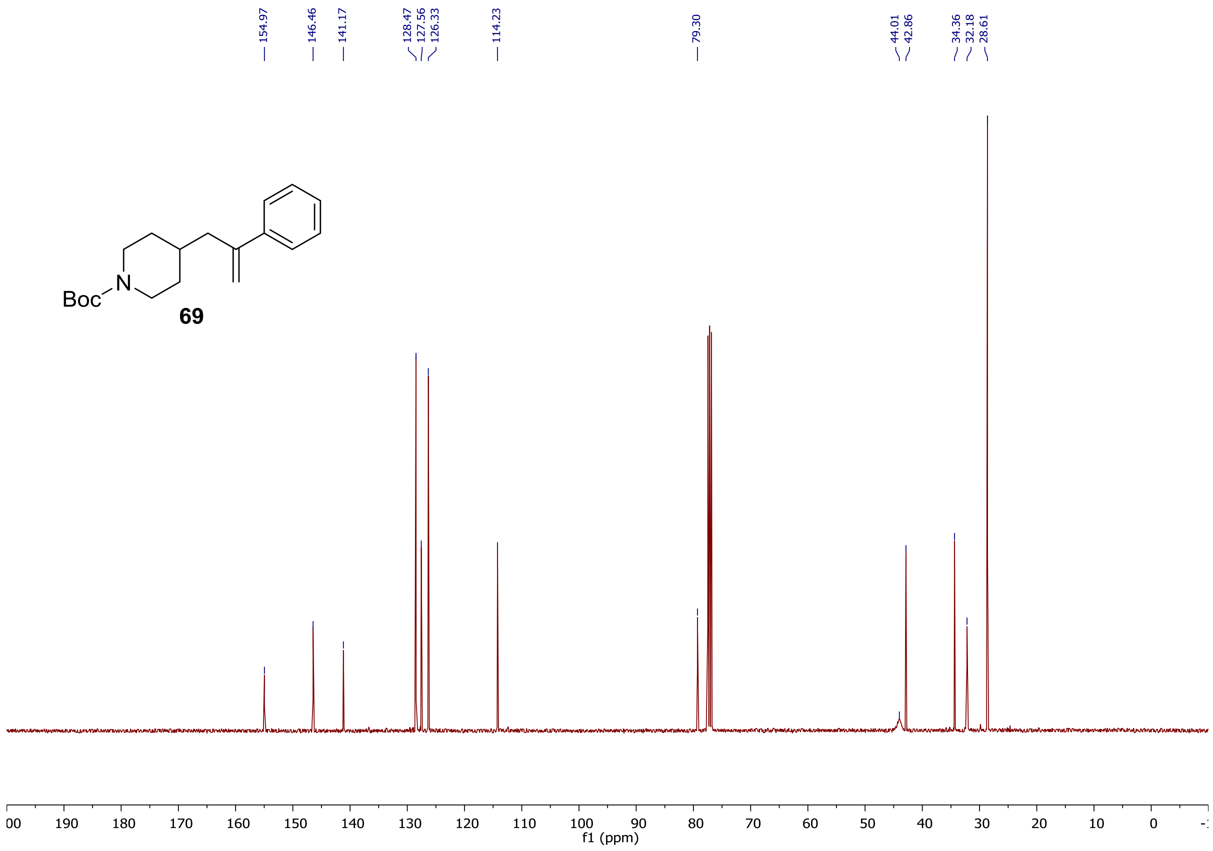

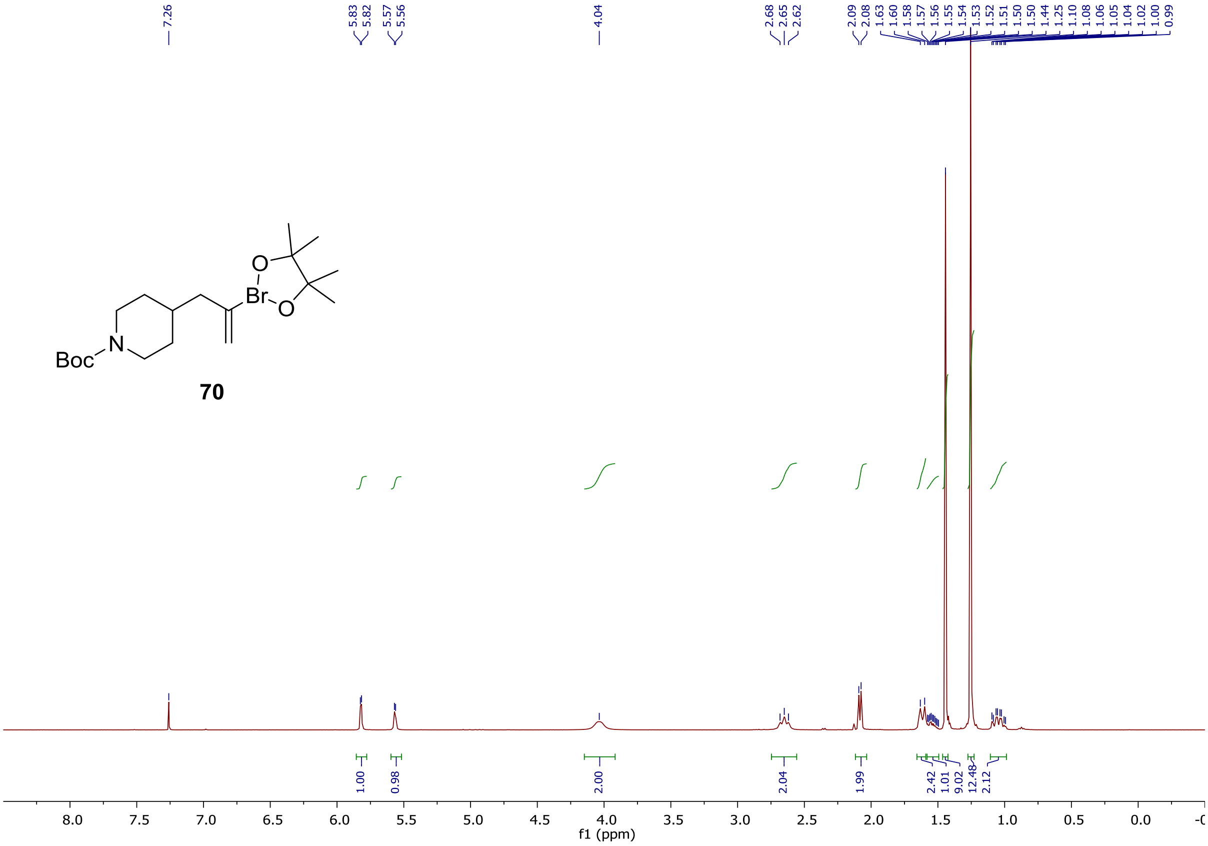
7.26
5.83
5.82
5.57
5.56
4.04
2.68
2.65
2.62
2.09
2.08
1.63
1.60
1.58
1.57
1.56
1.55
1.54
1.53
1.52
1.51
1.50
1.50
1.44
1.25
1.10
1.08
1.06
1.05
1.04
1.02
1.00
0.99
O
Br
O
Boc
N
70
1.00
0.98
2.00
2.04
1.99
2.42
1.01
9.02
12.48
2.12
8.0
7.5
7.0
6.5
6.0
5.5
5.0
4.5
4.0
3.5
3.0
2.5
2.0
1.5
1.0
0.5
0.0
f1 (ppm)

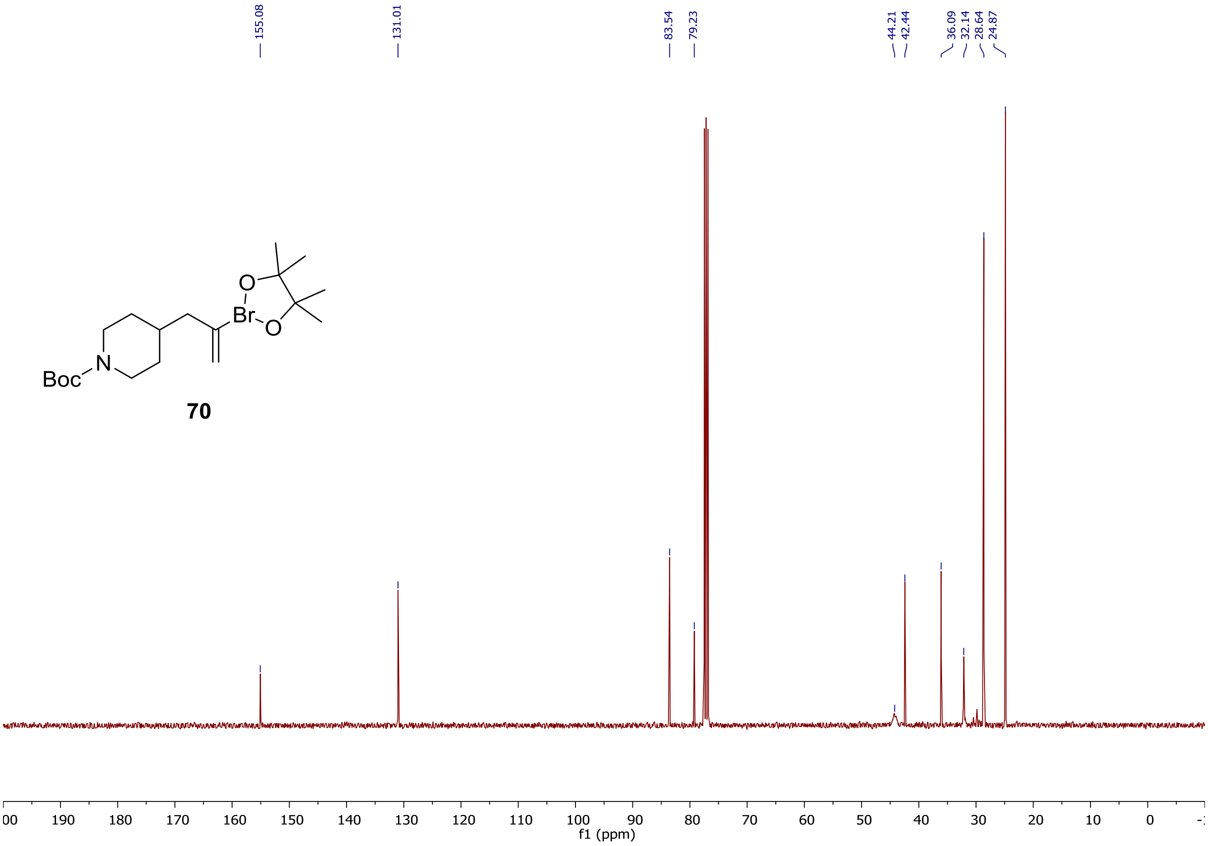
155.08
131.01
83.54
79.23
44.21
42.44
36.09
32.14
28.64
24.87
O
Br
O
Boc
N
70
190
180
170
160
150
140
130
120
110
100
90
80
70
60
50
40
30
20
10
0
f1 (ppm)

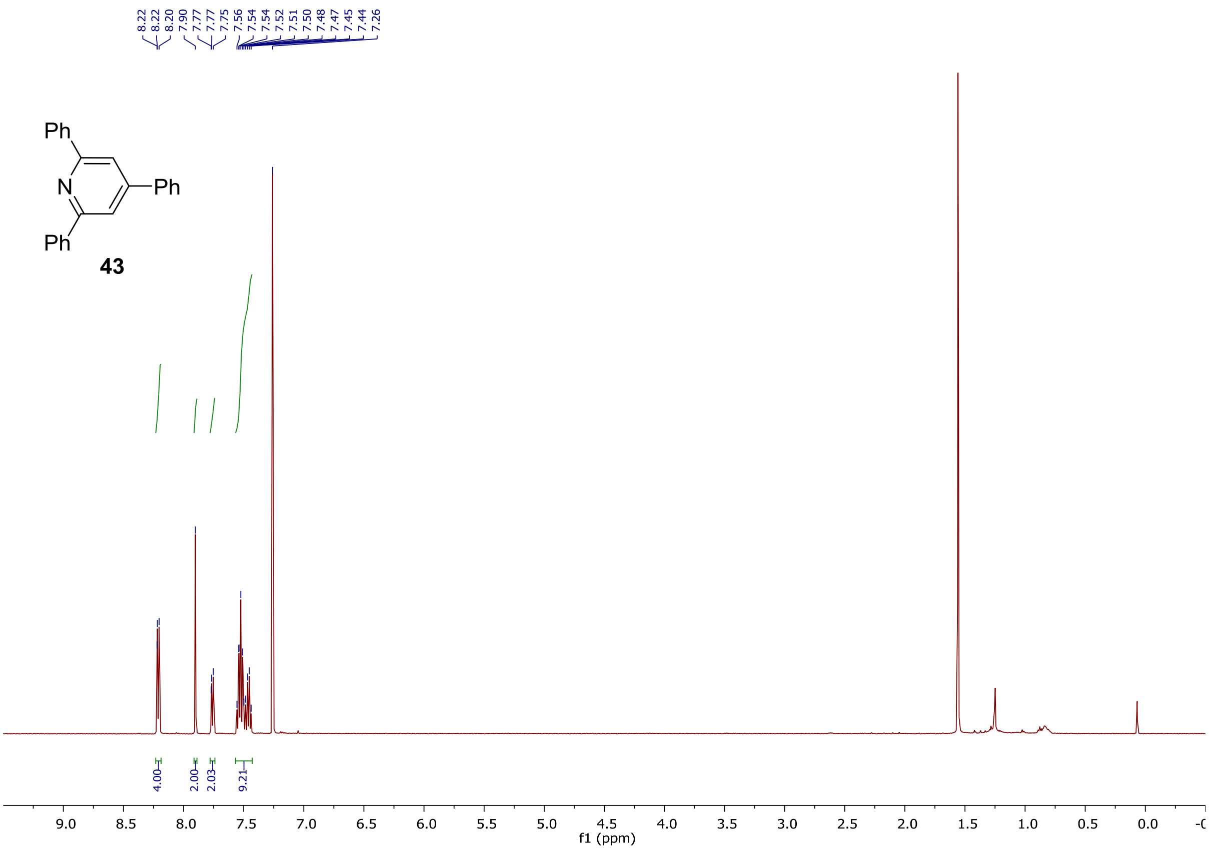

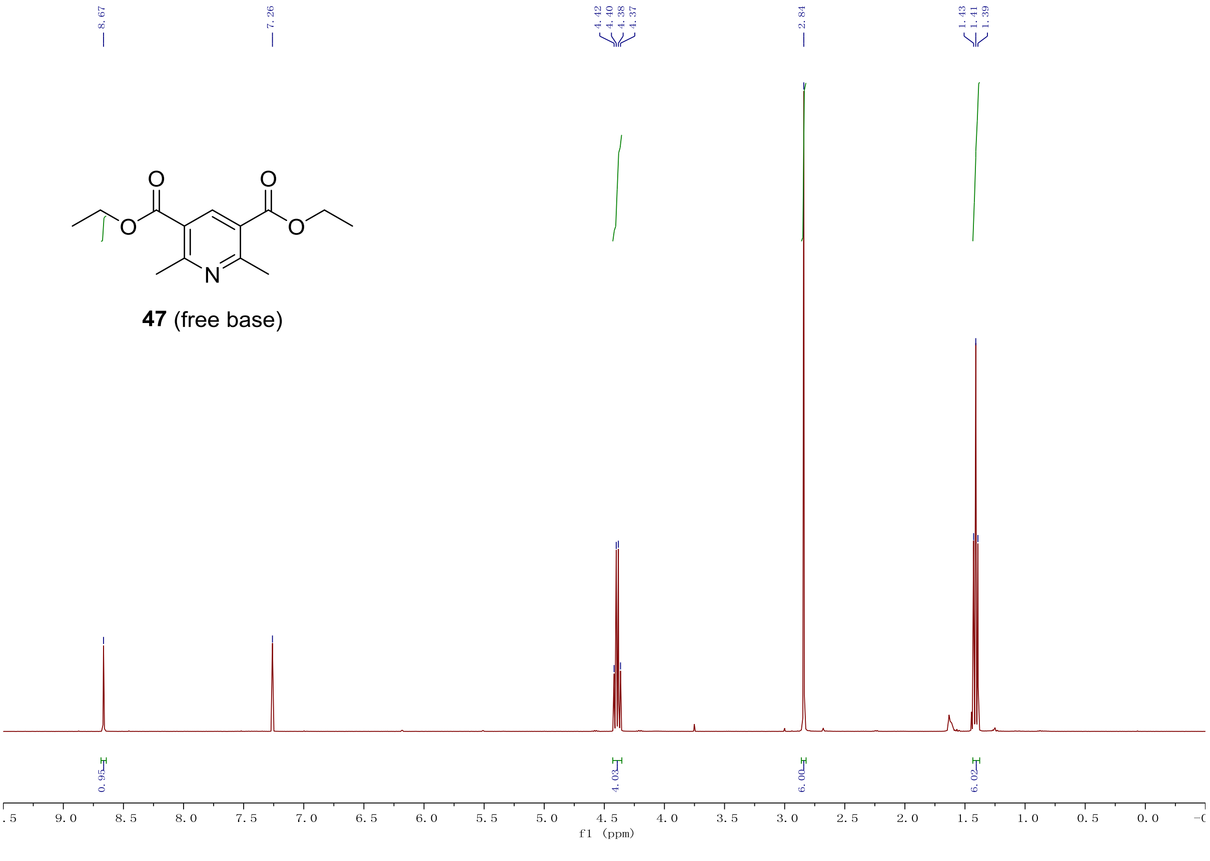

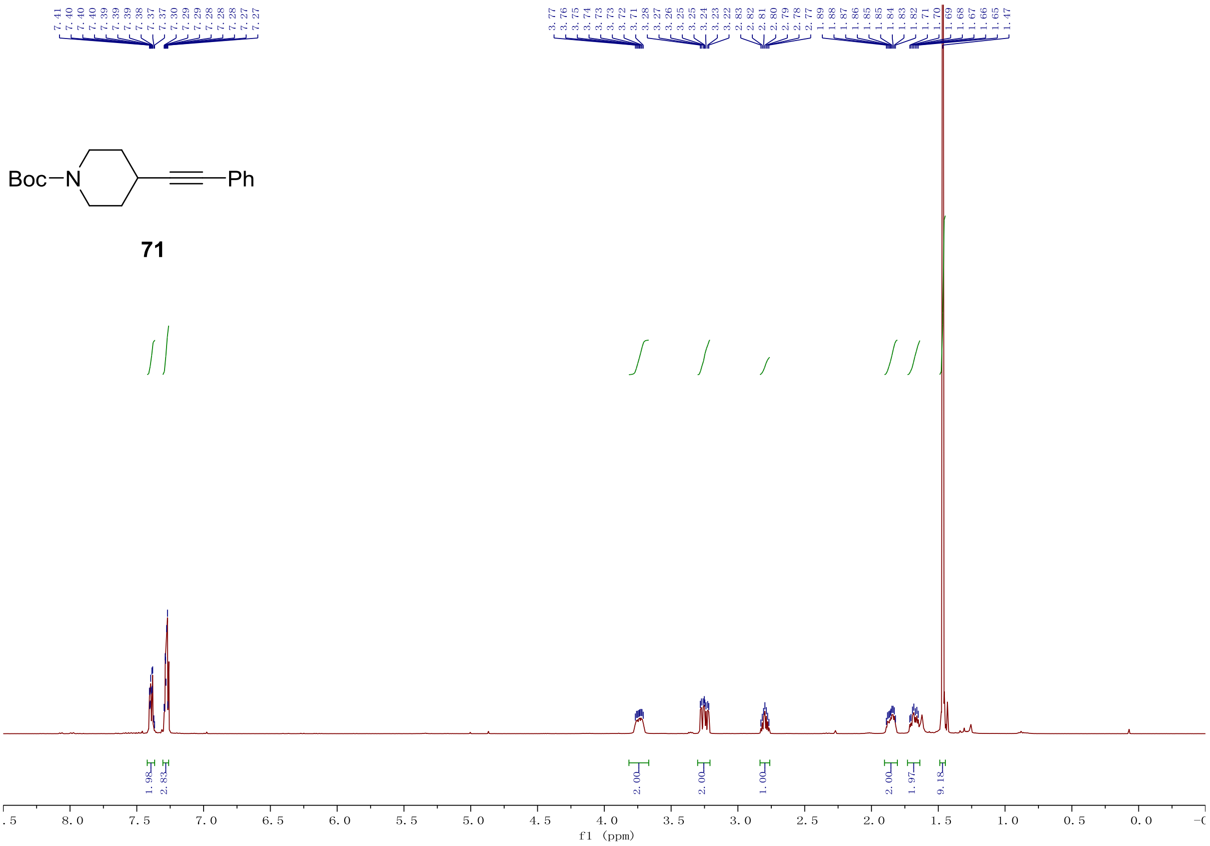
Boc
N
Ph
71
f1 (ppm)

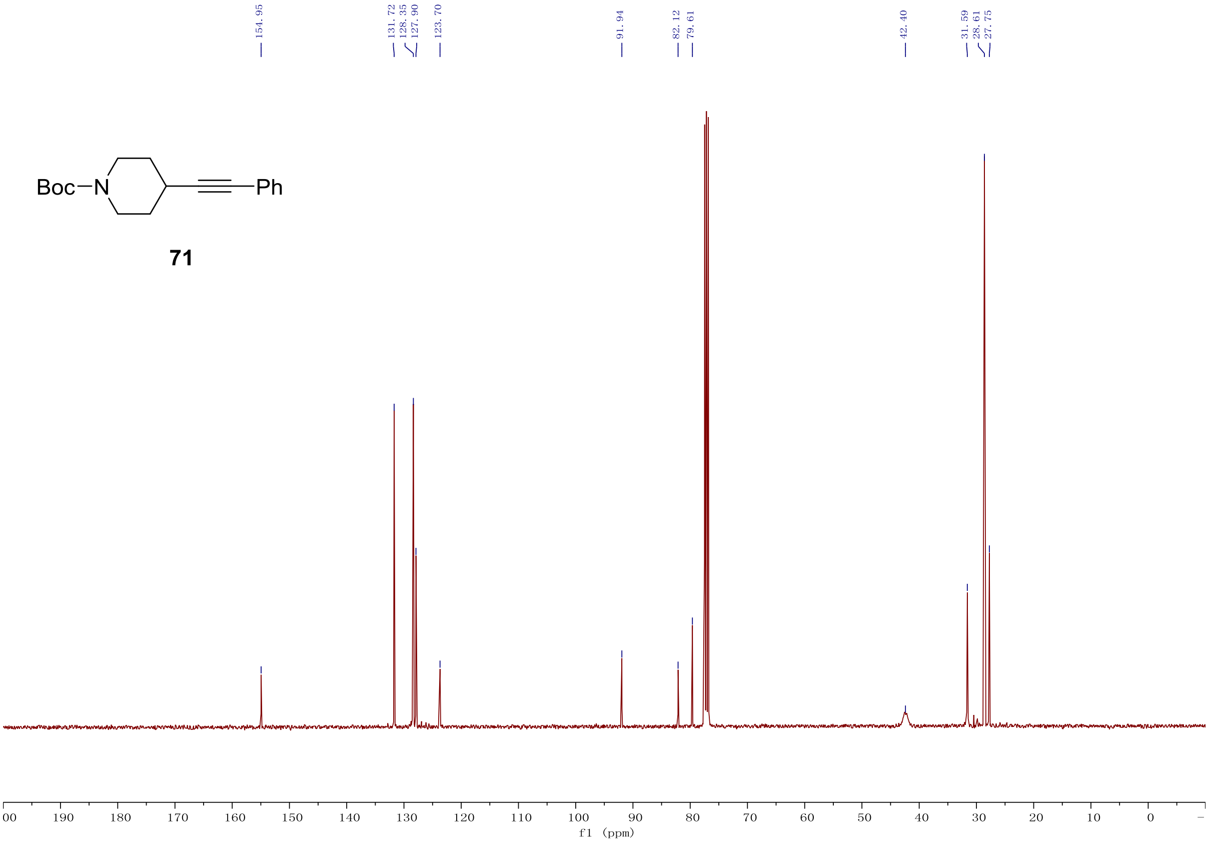
Boc
N
Ph
71
f1 (ppm)

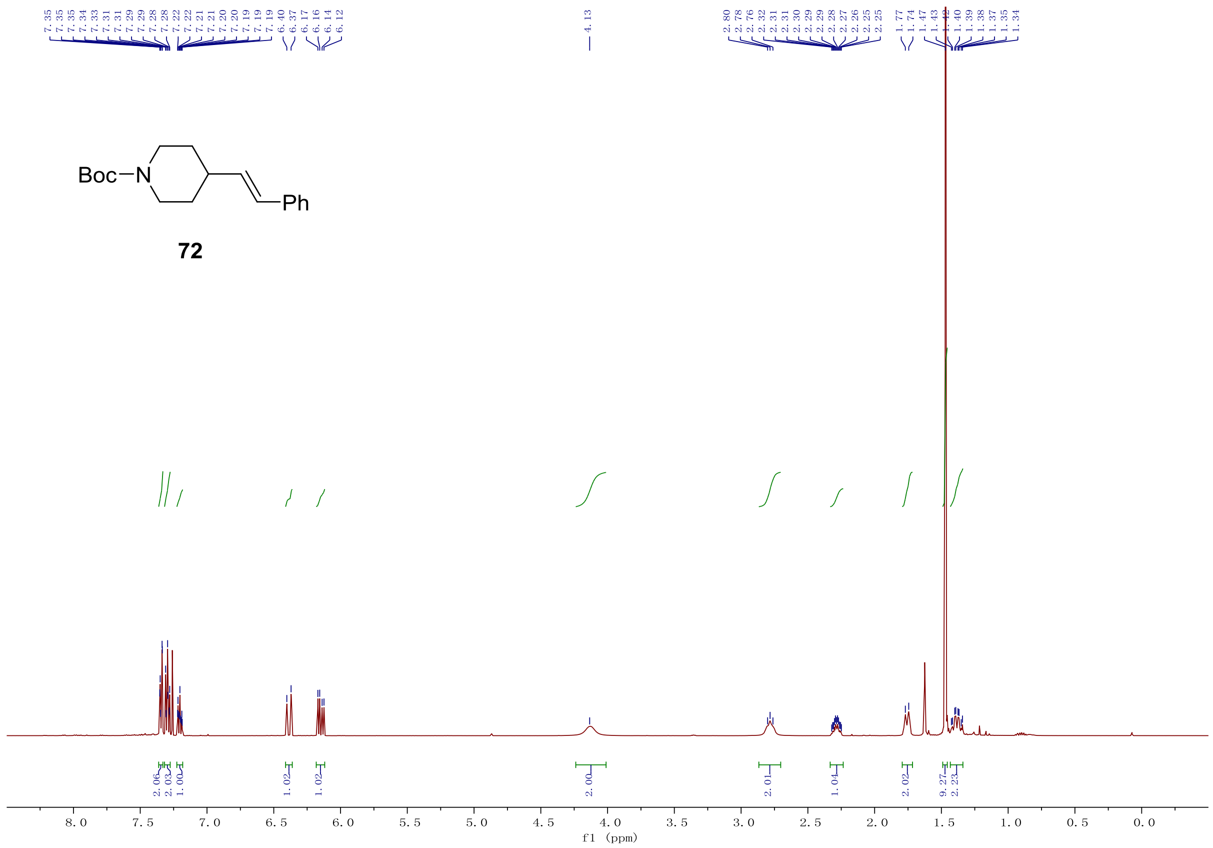
7.35 7.35 7.34 7.33 7.31 7.31 7.29 7.29 7.28 7.28 7.22 7.22 7.21 7.21 7.20 7.20 7.19 7.19 7.19 6.40 6.37 6.17 6.16 6.14 6.12
4.13
2.80 2.78 2.76 2.32 2.31 2.31 2.30 2.29 2.29 2.28 2.27 2.26 2.25 2.25
1.77 1.74 1.47 1.43 1.40 1.39 1.38 1.37 1.35 1.34
Boc–N
Ph
72
2.06 2.03 1.00 1.02 1.02 2.00 2.01 1.04 2.02 9.27 2.23
8.0 7.5 7.0 6.5 6.0 5.5 5.0 4.5 4.0 3.5 3.0 2.5 2.0 1.5 1.0 0.5 0.0
f1 (ppm)

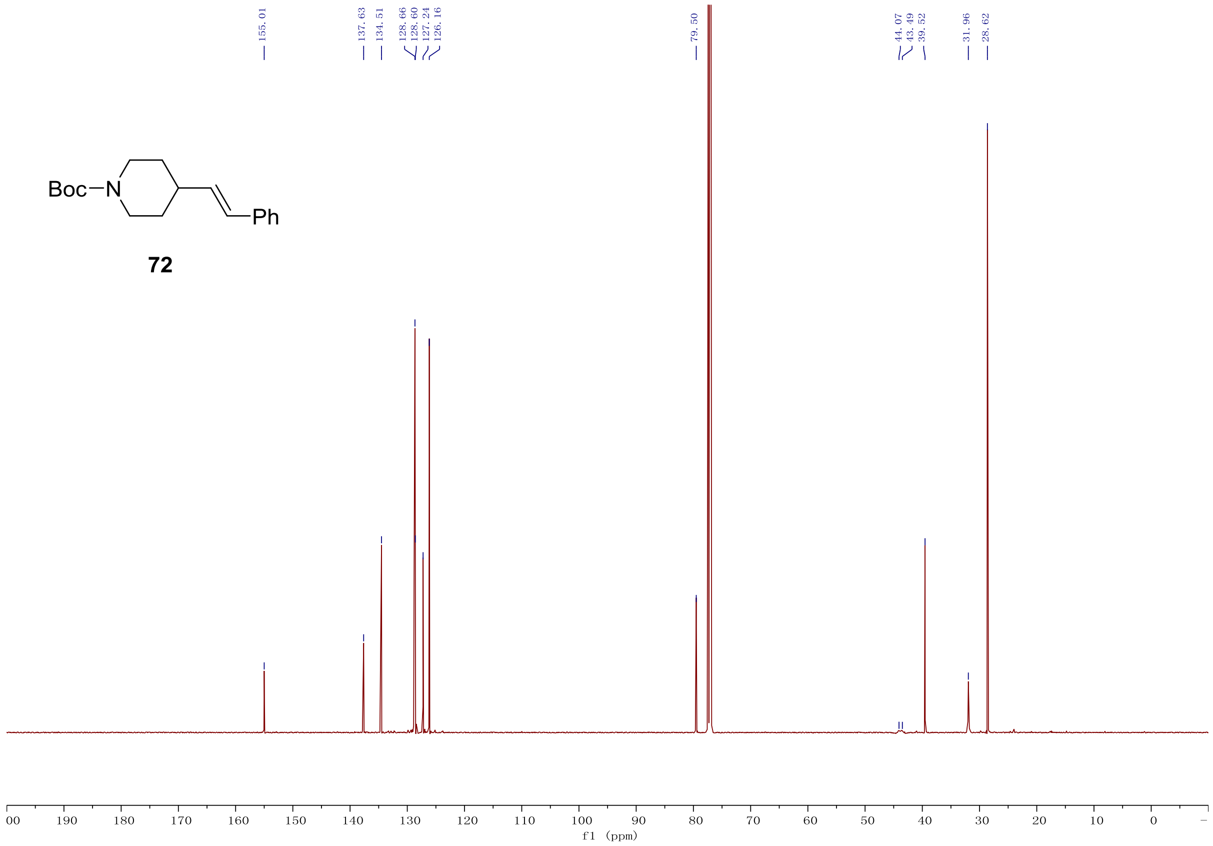
155.01
137.63 134.51 128.66 128.60 127.24 126.16
79.50
44.07 43.49 39.52 31.96 28.62
Boc–N
Ph
72
00 190 180 170 160 150 140 130 120 110 100 90 80 70 60 50 40 30 20 10 0
f1 (ppm)

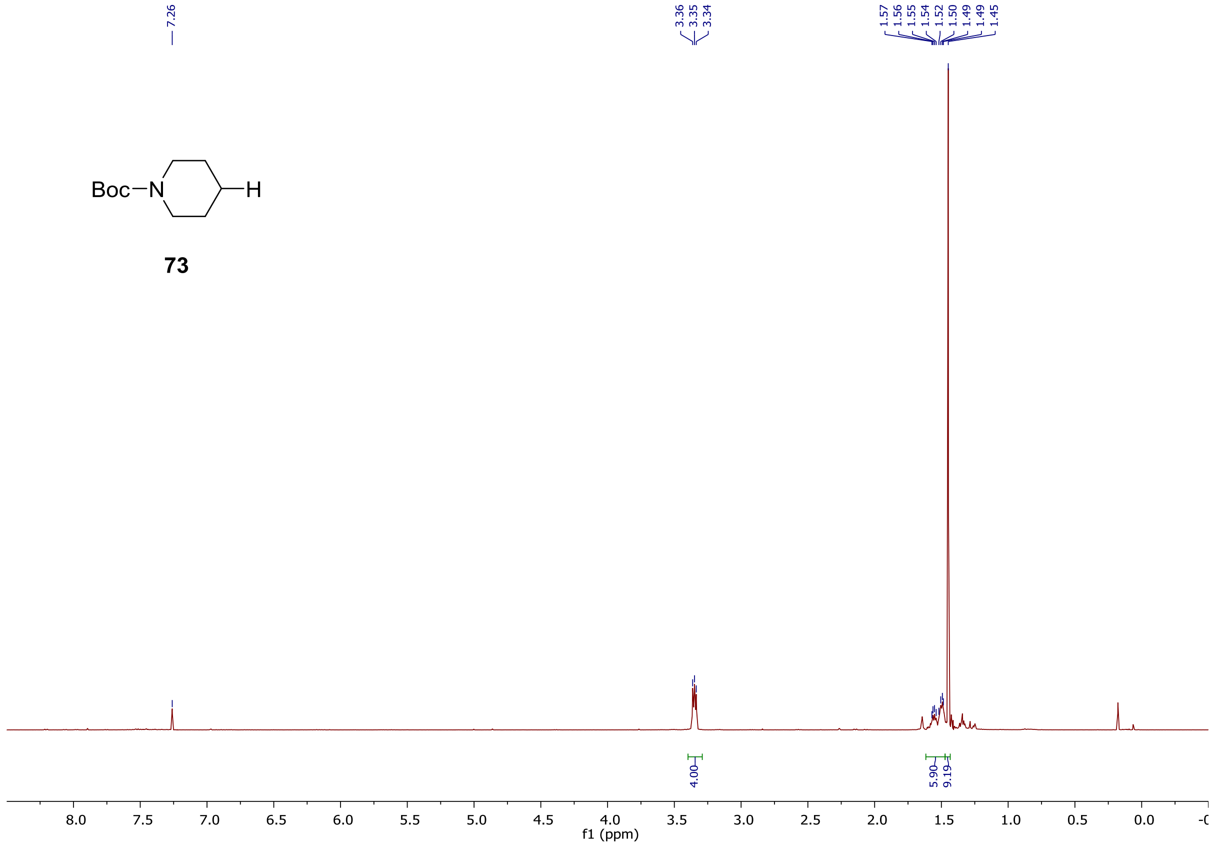

73

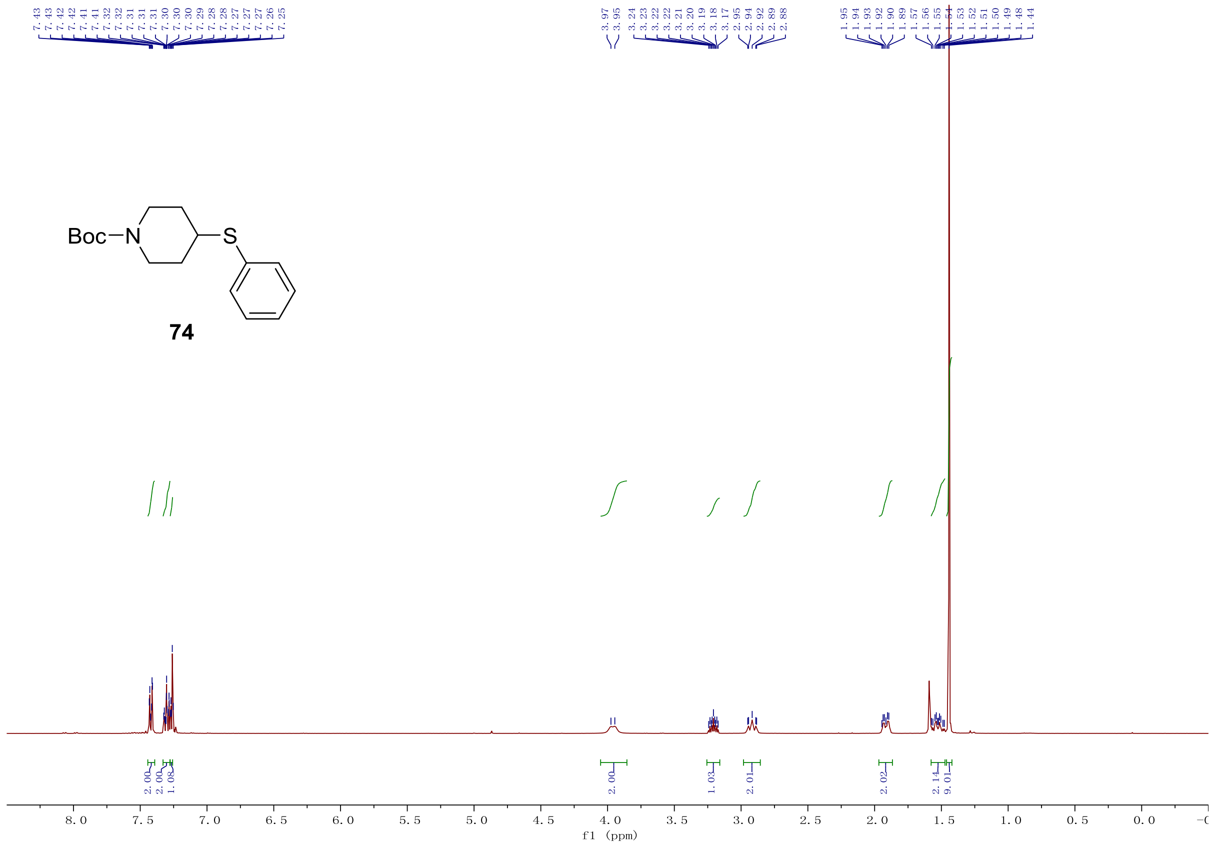

74

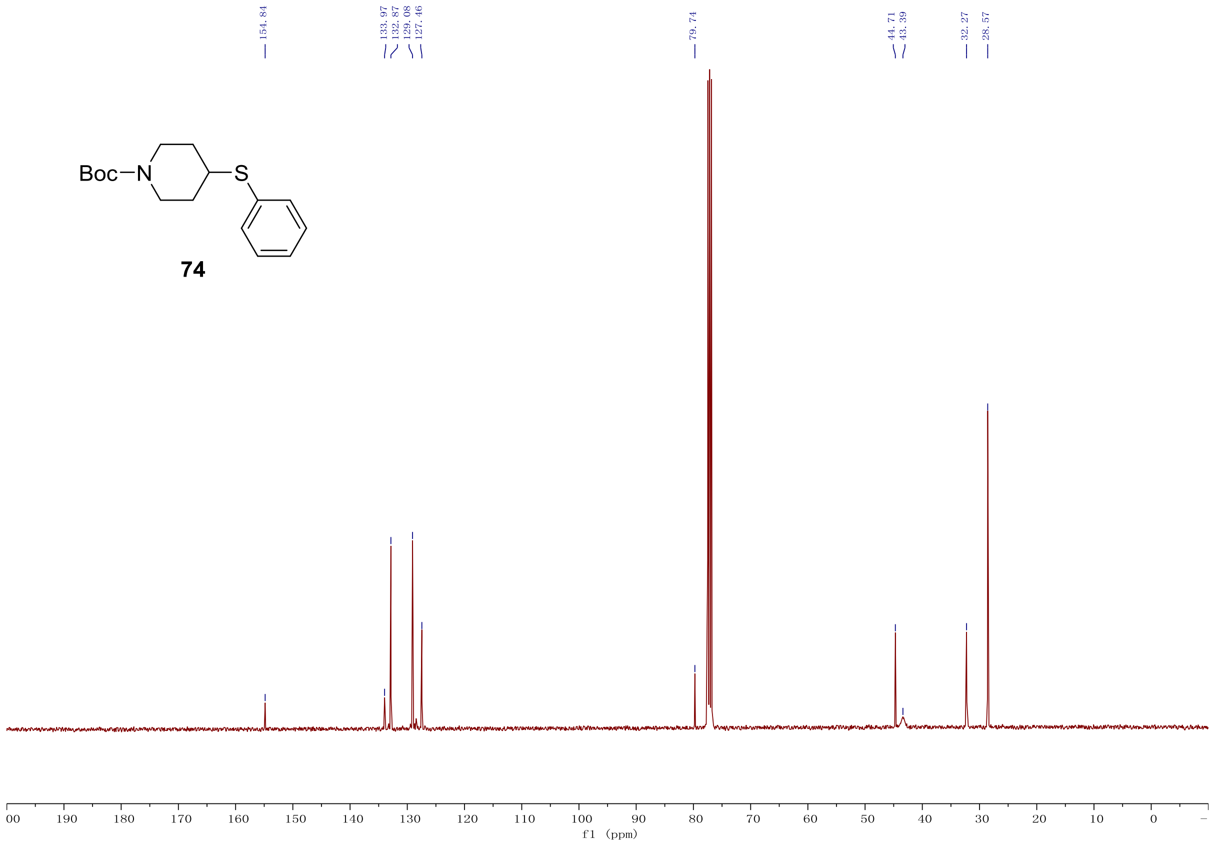

74

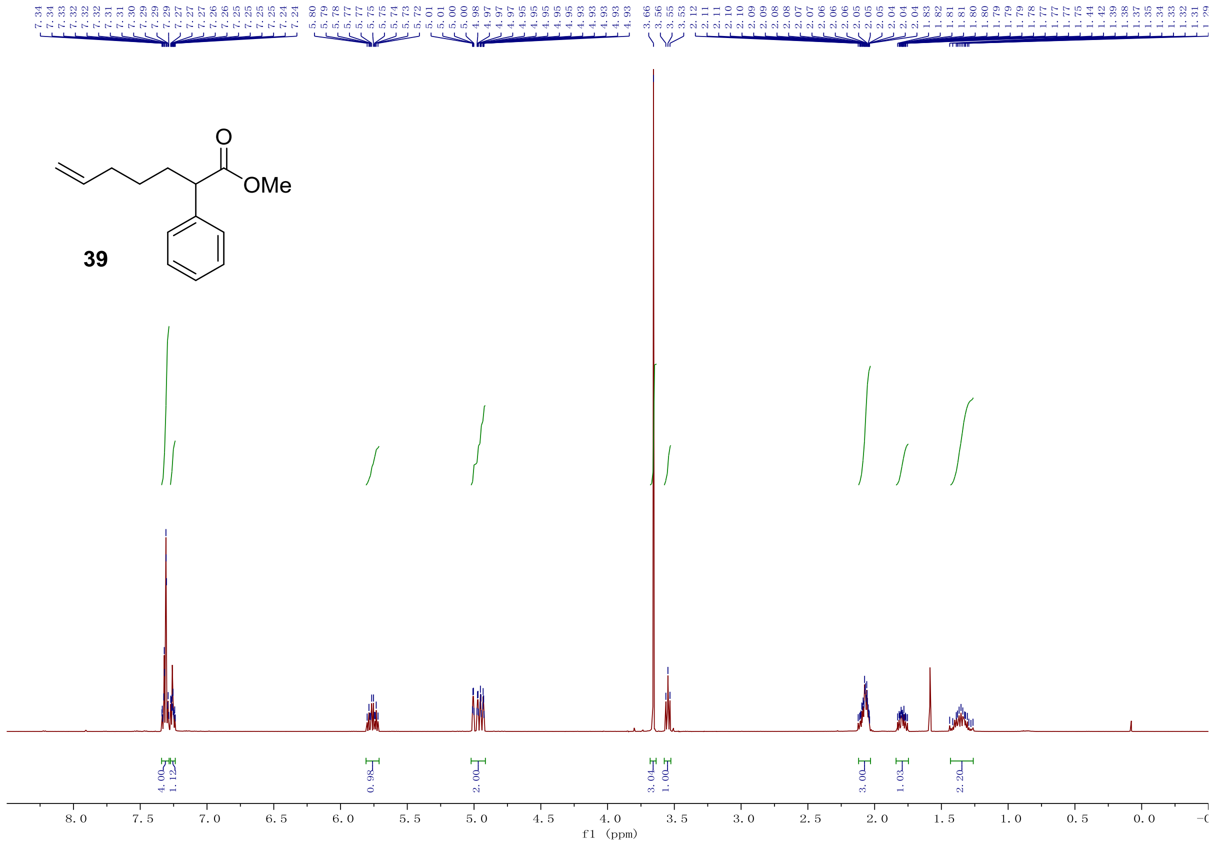

39

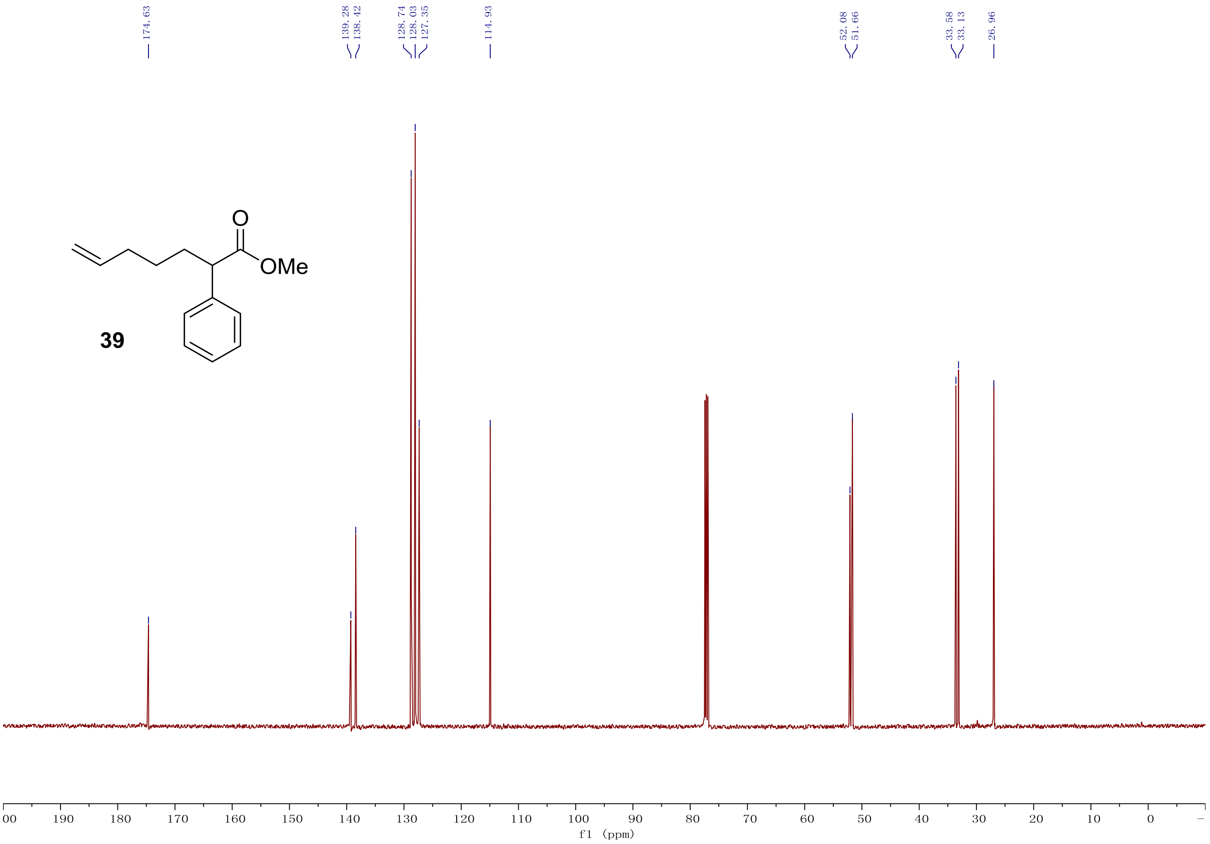
174.63
139.28
138.42
128.74
128.03
127.35
114.93
52.08
51.66
33.58
33.13
26.96
O
OMe
39
f1 (ppm)

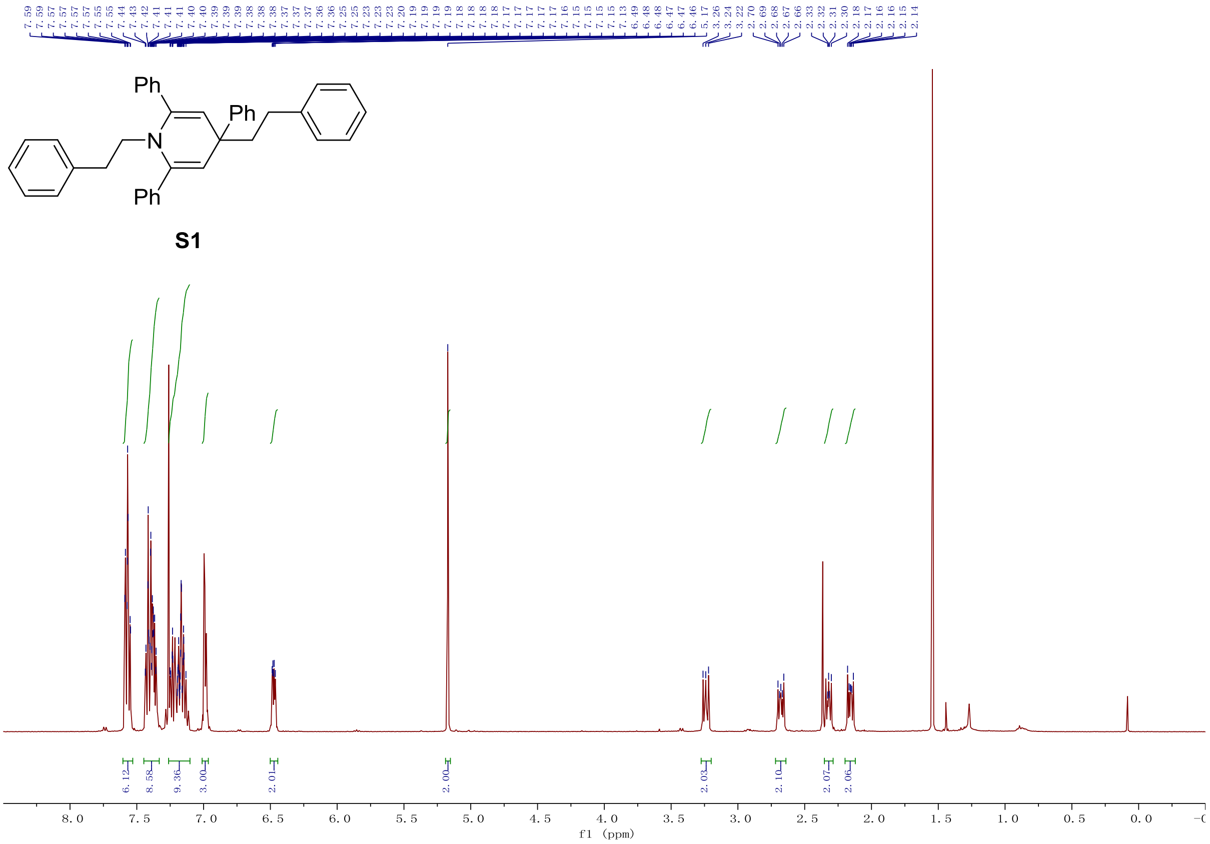
7.59 7.59 7.57 7.57 7.57 7.55 7.55 7.44 7.43 7.42 7.41 7.41 7.41 7.40 7.39 7.39 7.38 7.38 7.37 7.37 7.37 7.36 7.36 7.25 7.25 7.23 7.23 7.23 7.20 7.19 7.19 7.19 7.19 7.18 7.18 7.18 7.18 7.17 7.17 7.17 7.17 7.16 7.15 7.15 7.15 7.13 6.49 6.48 6.48 6.47 6.47 6.46 5.17 3.26 3.24 3.22 2.70 2.69 2.68 2.67 2.66 2.33 2.32 2.31 2.30 2.18 2.17 2.16 2.15 2.14
Ph
Ph
N
Ph
S1
6.12
8.58
9.36
3.00
2.01
2.00
2.03
2.10
2.07
2.06
f1 (ppm)

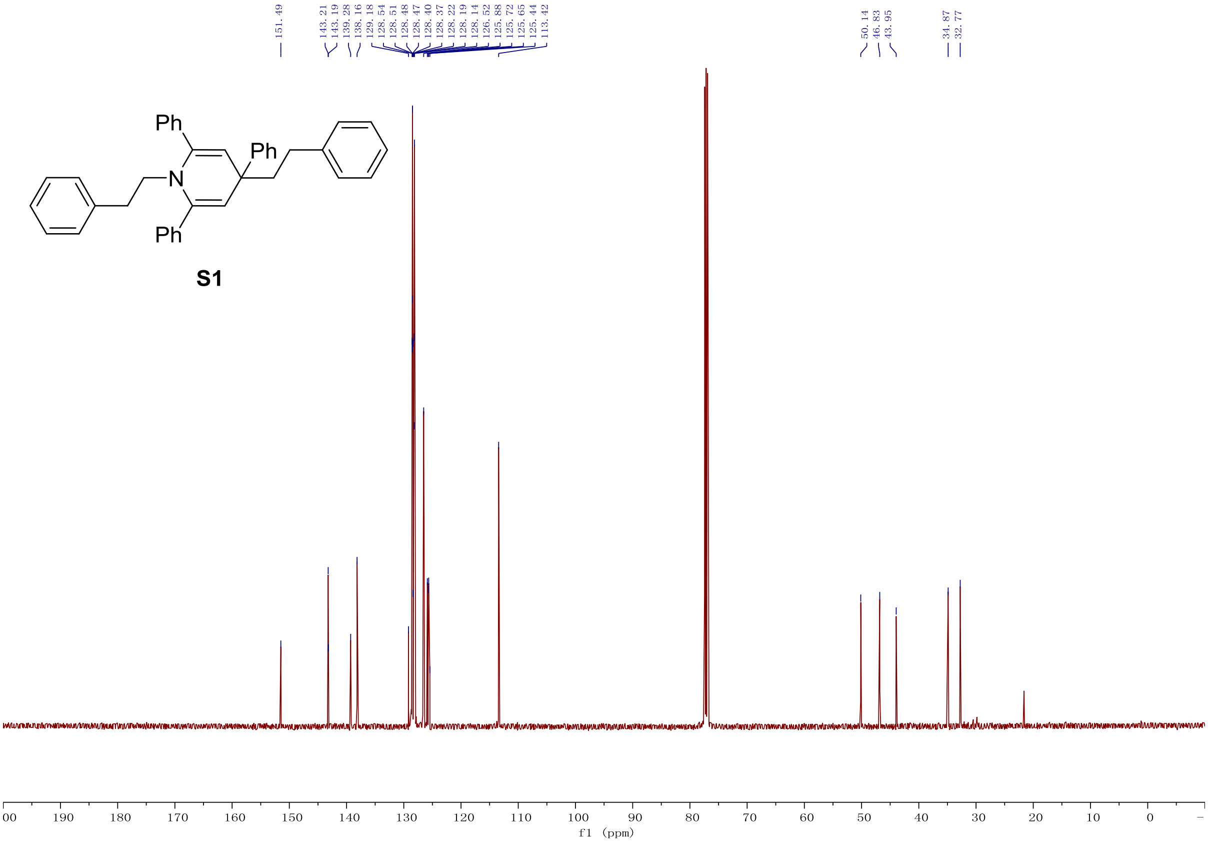
Ph
Ph
N
Ph
S1
f1 (ppm)

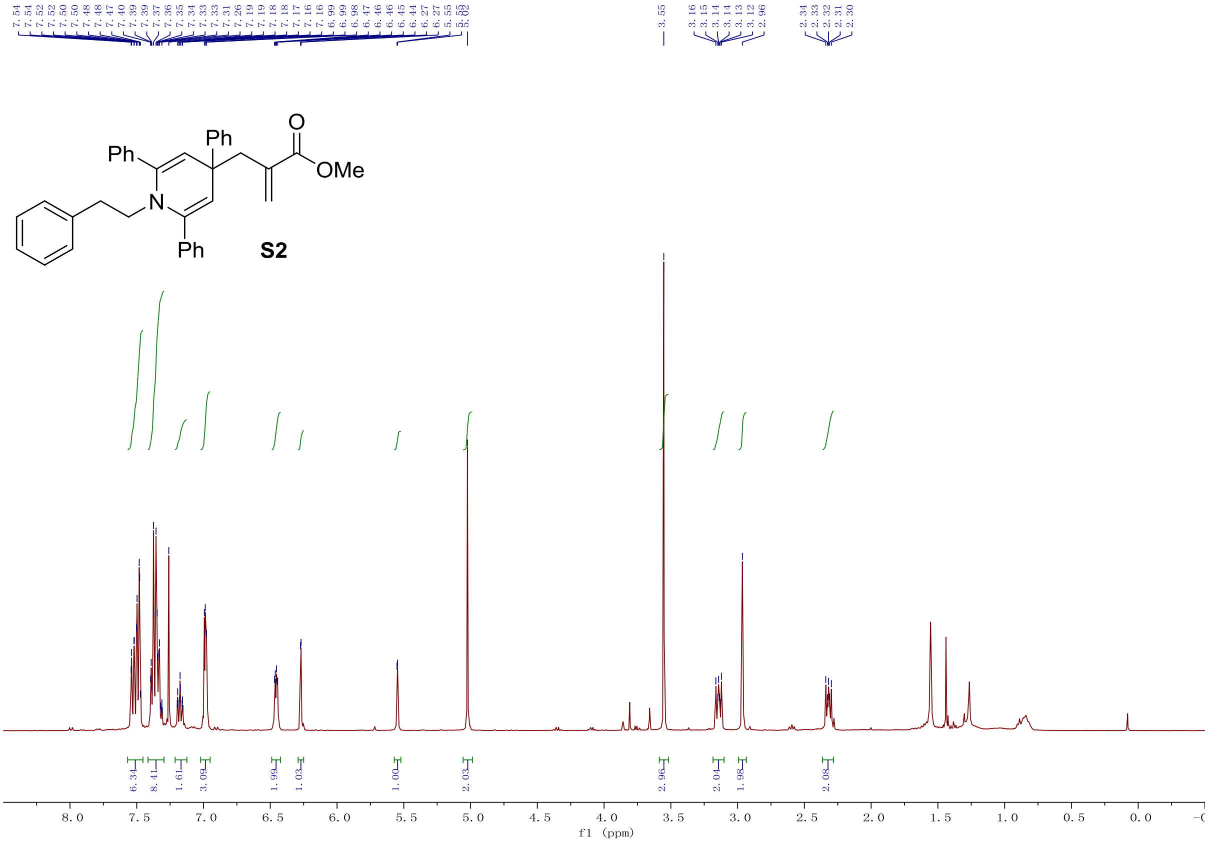
Ph
Ph
O
OMe
N
Ph
S2
f1 (ppm)

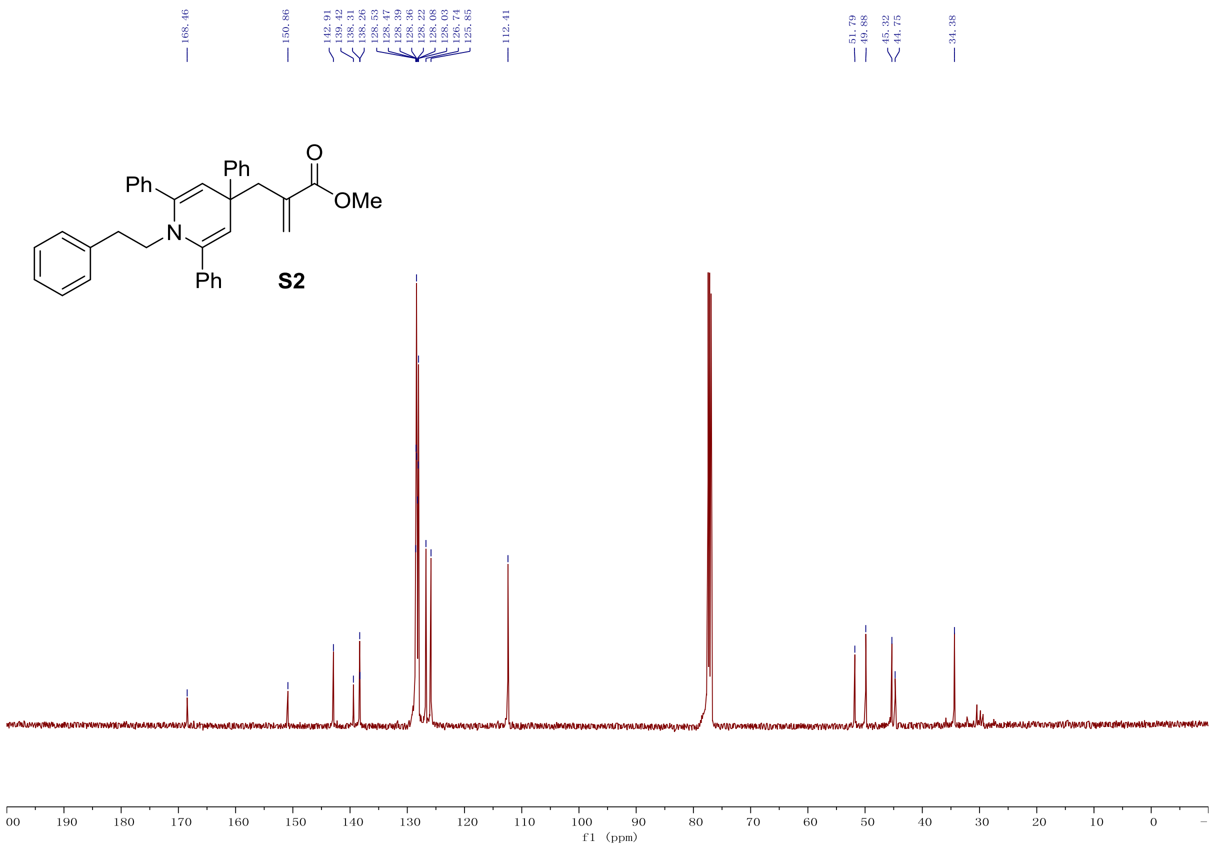

Ph
Ph
O
OMe
N
Ph
S2
f1 (ppm)

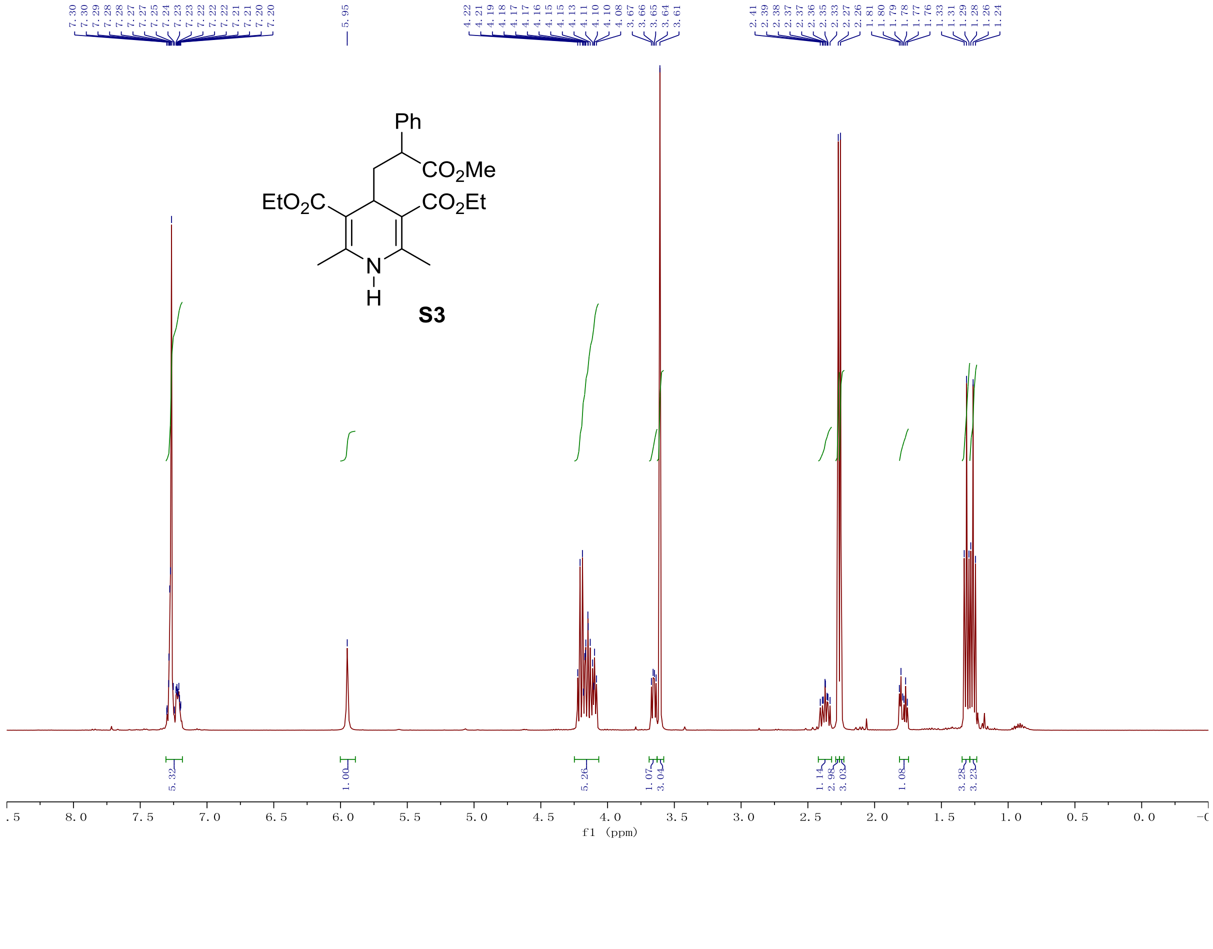

Ph
CO_2Me
EtO_2C
CO_2Et
N
H
S3
f1 (ppm)

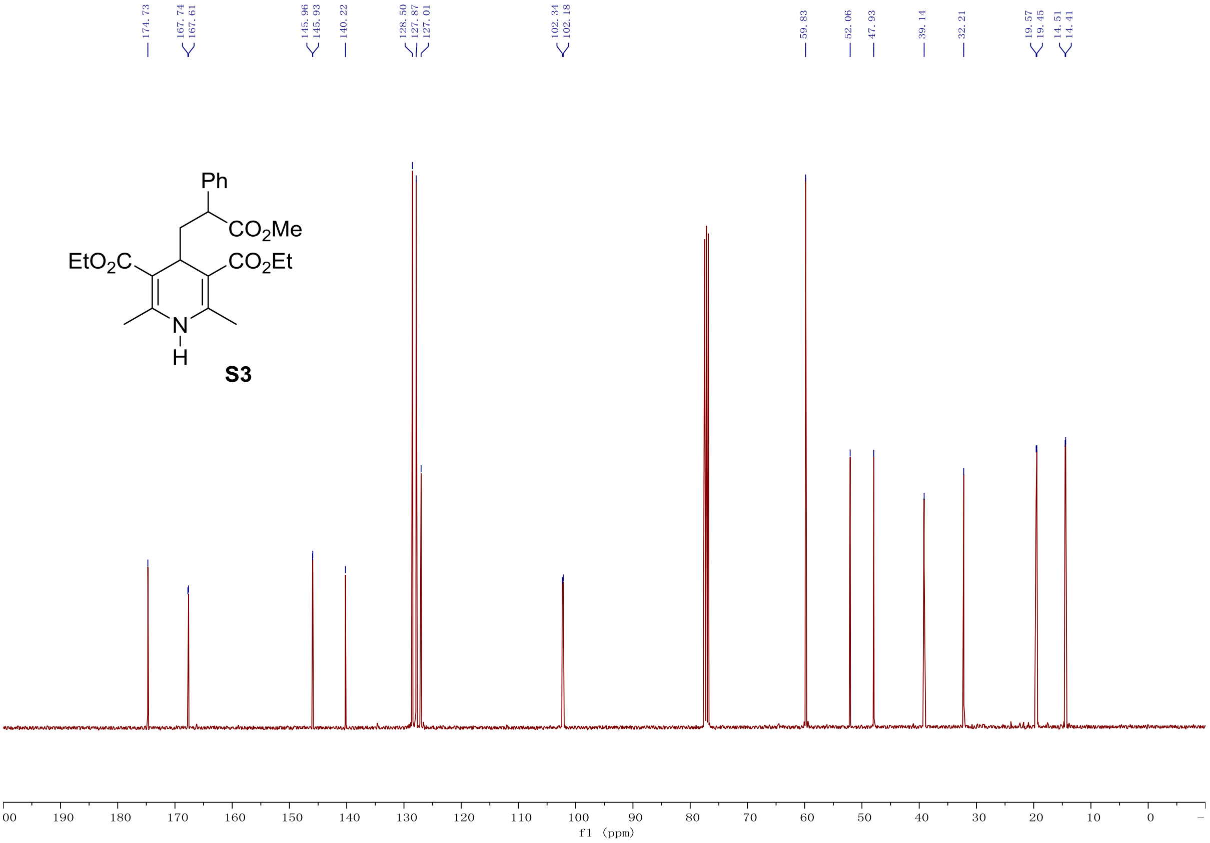
174.73
167.74
167.61
145.96
145.93
140.22
128.50
127.87
127.01
102.34
102.18
59.83
52.06
47.93
39.14
32.21
19.57
19.45
14.51
14.41
Ph
CO_2Me
EtO_2C
CO_2Et
N
H
S3
f1 (ppm)

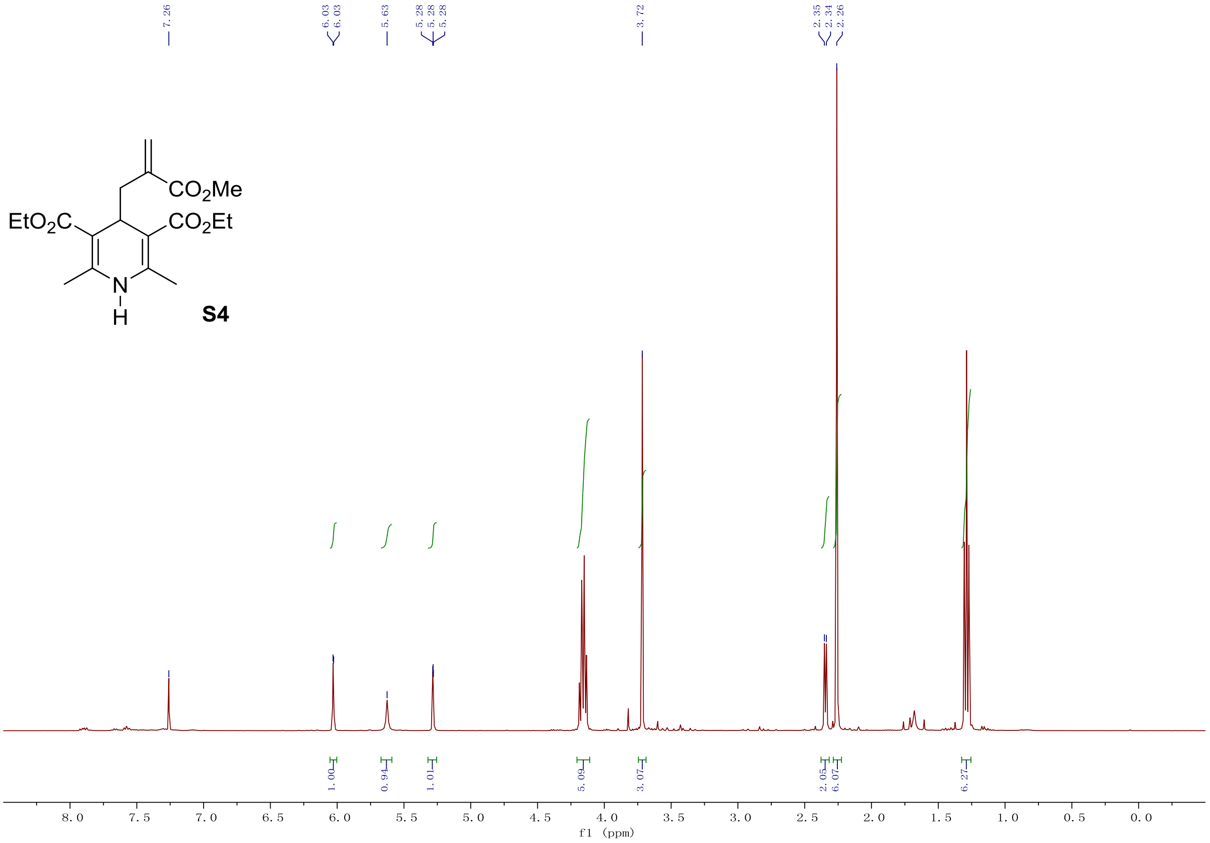
7.26
6.03
6.03
5.63
5.28
5.28
5.28
3.72
2.35
2.34
2.26
CO_2Me
EtO_2C
CO_2Et
N
H
S4
1.00
0.94
1.01
5.09
3.07
2.05
6.07
6.27
f1 (ppm)

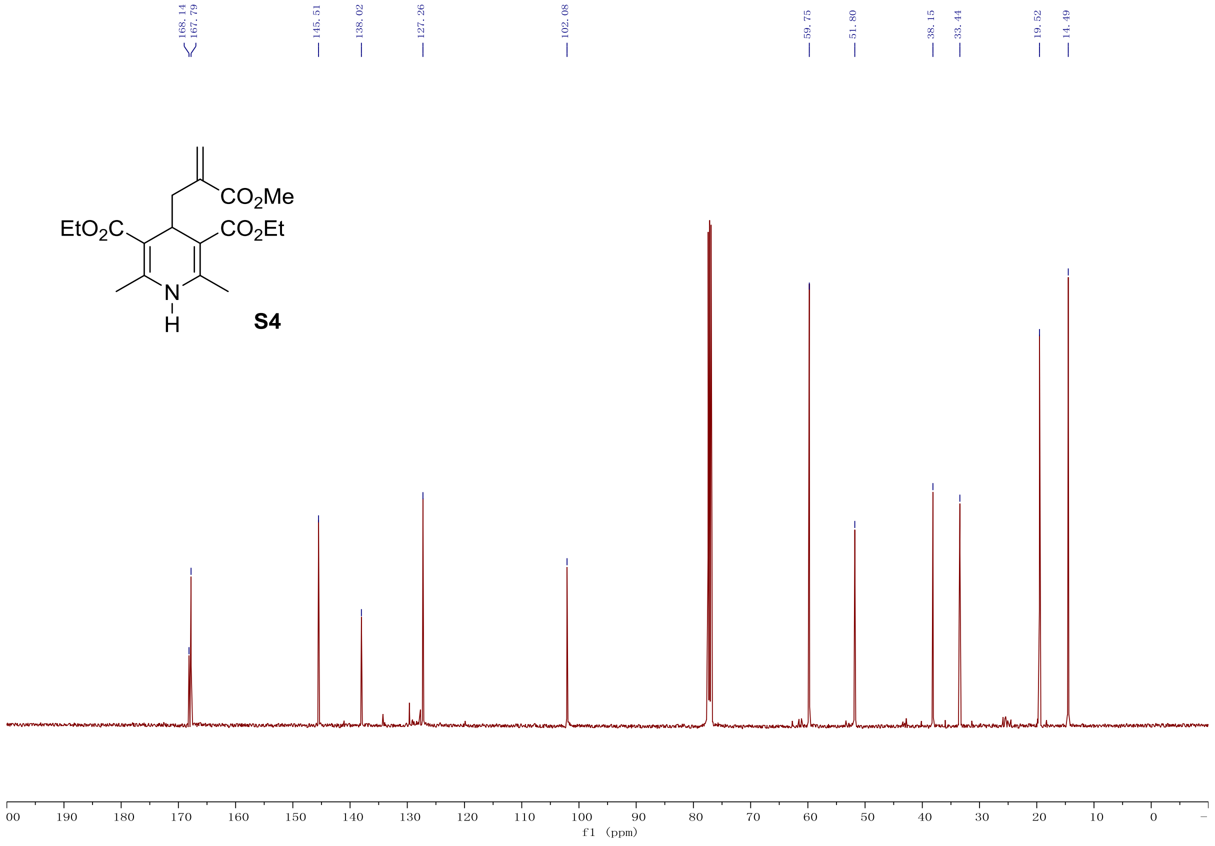
168.14
167.79
145.51
138.02
127.26
102.08
59.75
51.80
38.15
33.44
19.52
14.49
CO2Me
EtO2C
CO2Et
N
H
S4
f1 (ppm)
